# Dual functions of Macpiwi1 in transposon silencing and stem cell maintenance in the flatworm *Macrostomum lignano*

**DOI:** 10.1261/rna.052456.115

**Published:** 2015-11

**Authors:** Xin Zhou, Giorgia Battistoni, Osama El Demerdash, James Gurtowski, Julia Wunderer, Ilaria Falciatori, Peter Ladurner, Michael C. Schatz, Gregory J. Hannon, Kaja A. Wasik

**Affiliations:** 1 Cold Spring Harbor Laboratory and Watson School of Biological Sciences, Cold Spring Harbor, New York 11724, USA; 2 Molecular and Cellular Biology Graduate Program, Stony Brook University, Stony Brook, New York 11794, USA; 3 University of Innsbruck, Institute of Zoology and CMBI, A-6020 Innsbruck, Austria; 4 CRUK Cambridge Institute, Li Ka Shing Centre, University of Cambridge, Cambridge CB2 0RE, United Kingdom

**Keywords:** PIWI proteins, piRNAs, transposon silencing, stem cell maintenance, *Macrostomum*

## Abstract

PIWI proteins and piRNA pathways are essential for transposon silencing and some aspects of gene regulation during animal germline development. In contrast to most animal species, some flatworms also express PIWIs and piRNAs in somatic stem cells, where they are required for tissue renewal and regeneration. Here, we have identified and characterized piRNAs and PIWI proteins in the emerging model flatworm *Macrostomum lignano*. We found that *M. lignano* encodes at least three PIWI proteins. One of these, Macpiwi1, acts as a key component of the canonical piRNA pathway in the germline and in somatic stem cells. Knockdown of *Macpiwi1* dramatically reduces piRNA levels, derepresses transposons, and severely impacts stem cell maintenance. Knockdown of the piRNA biogenesis factor *Macvasa* caused an even greater reduction in piRNA levels with a corresponding increase in transposons. Yet, in *Macvasa* knockdown animals, we detected no major impact on stem cell self-renewal. These results may suggest stem cell maintenance functions of PIWI proteins in flatworms that are distinguishable from their impact on transposons and that might function independently of what are considered canonical piRNA populations.

## INTRODUCTION

Argonaute proteins have emerged as essential components of gene regulatory mechanisms. In association with their small RNA partners, the Argonaute family of proteins silences target genes at both transcriptional and post-transcriptional levels. In animals, there are two clades of Argonaute proteins—the Argonaute (AGO) clade and the PIWI clade (Carmell et al. 2002; Höck and Meister 2008). The AGO clade typically associates with either microRNAs (miRNAs) or endogenous small interfering RNAs (endo-siRNAs). miRNAs play well-established roles in the regulation of protein-coding mRNAs, while endo-siRNAs are generally involved in transposon silencing (Höck and Meister 2008). PIWI proteins associate with PIWI-interacting RNAs (piRNAs). These are generally 24–35 nucleotides (nt) in length and have been shown to protect genomic integrity by suppressing transposable elements (TEs) specifically during animal germline development (Ishizu et al. 2012). piRNAs primarily derive from genomic aggregates of transposon insertions, termed piRNA clusters, and act to guide PIWI proteins to transposon mRNAs by sequence complementarity (Brennecke et al. 2007). PIWI proteins can then silence transposons either post-transcriptionally—by cleaving transposon mRNAs, or transcriptionally—by changing the chromatin structure (Luteijn and Ketting 2013). These functions prevent transposon propagation and earned PIWIs their nickname—“guardians of the genome” (Senti and Brennecke 2010). Altogether, piRNAs and PIWIs are indispensable for germline formation and maintenance, and mutations in piRNA pathway components usually cause sterility (Klattenhoff et al. 2007; Soper et al. 2008; Thomson and Lin 2009).

Although most studies of piRNAs and PIWIs have been focused on transposon silencing in the germline, a number of studies have posited roles for piRNAs in somatic cells. In the California sea hare *Aplysia californica*, piRNAs are thought to participate in memory formation (Rajasethupathy et al. 2012). PIWI expression has also been observed in various types of human cancers, though neither their interactions with piRNAs nor concrete functional roles have been demonstrated in this setting (Suzuki et al. 2012). Notably, PIWIs have also been shown to play a critical role in the regenerative capabilities of flatworm somatic stem cells (neoblasts) (Reddien et al. 2005; Palakodeti et al. 2008; De Mulder et al. 2009). The potential involvement of PIWI proteins in flatworm stem cells is of particular interest since the founding member of the PIWI clade (Piwi in *Drosophila melanogaster*) was described as a factor involved in germline stem cell (GSC) maintenance (Lin and Spradling 1997). In *D. melanogaster* ovaries, the best-studied role of Piwi is transposon silencing, although in *Piwi* knockouts, germline stem cells (GSCs) cease to divide after a few initial divisions, and the germ cell-depleted gonads contain only a few egg chambers/sperm bundles (Cox et al. 1998, 2000). It has remained an open question whether the roles of Piwi in GSC regulation and transposon silencing are interrelated; however, some recent evidence suggests that these two roles are separable (Klenov et al. 2011). Truncation of the nuclear localization signal in Piwi does not affect GSC maintenance, although the same mutation causes derepression of transposable elements (TEs) and sterility, confirming that the Piwi TE silencing function is impaired. These observations suggest that PIWIs may have a conserved role in stem cell regulation, but it remains to be determined whether this role is linked to transposon silencing or is piRNA dependent.

Flatworms harbor an unusual population of adult stem cells known as neoblasts (Bode et al. 2006; Baguñà 2012). Neoblasts are responsible for the impressive regeneration abilities of these animals—some species can regenerate their entire body after lethal irradiation, with all tissues being derived from a single transplanted neoblast (Wagner et al. 2011). In the planarian *Schmidtea mediterranea*, at least three PIWI proteins (SMEDWI1-3) are expressed in neoblasts and the depletion of either SMEDWI2 or SMEDWI3 can impair stem cell functions (Reddien et al. 2005; Palakodeti et al. 2008). It is however still largely unclear whether the major role of flatworm PIWIs is to sustain stem cell maintenance, foster differentiation, or perhaps both, depending on the context. It is also unknown whether piRNAs are essential for the action of SMEDWIs as stem cell regulators.

Here, we have focused on the PIWI proteins of an emerging flatworm model—*Macrostomum lignano. M. lignano* has both neoblast populations and regenerative characteristics similar to those of *S. mediterranea* (Reddien and Sánchez Alvarado 2004). The genomic sequence and transcriptome of *M. lignano* have recently been assembled (KA Wasik, J Gurtowski, X Zhou, OM Ramos, MJ Delás, O El Demerdash, G Battistoni, I Falciatori, DB Vizoso, AD Smith, et al., in prep.), opening the way for its use as a new model for studies of not only piRNA pathway but also of stem cell maintenance and differentiation. The one previously described *M. lignano* PIWI protein, Macpiwi1, is present in the germline and in neoblasts. It is required for neoblast maintenance and stem cell-dependent functions, such as regeneration, tissue renewal, and post-embryonic development (De Mulder et al. 2009). Here we identify two additional *M. lignano* PIWI proteins, and describe the *M. lignano* piRNA population and its Macpiwi1-associated subpopulation. *M. lignano* piRNAs, like piRNAs in other species, target transposons and participate in the piRNA amplification loop (ping-pong). We find that silencing of *Macpiwi1* leads to reduction of piRNA populations, transposon derepression and loss of stem cell proliferation. The outcome differs if piRNAs are depleted via knockdown of *Macvasa*, which has no impact on stem cell renewal and further differentiation.

## RESULTS

### *Macrostomum lignano* has conserved small RNA pathway components

Metazoans have highly conserved miRNA and piRNA pathways. The existence of PIWI proteins in *M. lignano* (De Mulder et al. 2009) strongly suggested the presence of small RNA pathways in this organism. In order to address this hypothesis, we searched for well-known and conserved miRNA and piRNA biogenesis factors in the genome and de novo transcriptome drafts that we recently assembled (KA Wasik, J Gurtowski, X Zhou, OM Ramos, MJ Delás, O El Demerdash, G Battistoni, I Falciatori, DB Vizoso, AD Smith, et al., in prep.). Briefly, we sequenced the genome of *M. lignano* using a combination of ∼170× coverage of Illumina short read sequencing (100-bp paired-end and mate-pair reads) and ∼130× coverage of PacBio long read sequencing (6.5 kbp mean read length) to produce an assembly with a contig N50 of 64 kbp. We also sequenced the mRNA from whole worms and sorted stem cells and de novo assembled the *M. lignano* transcriptome into ∼150,000 transcripts. The average assembled transcript length was 516 bp and the N50 of the transcriptome was 649 bp. Nearly all of the transcripts (97%, with >90% identity) align to our draft genome assembly. We also found the majority of core eukaryotic genes (99%) based on CEGMA (Core Eukaryotic Genes Mapping Approach) analysis, suggesting a nearly complete representation of the *M. lignano* genes in our assembly. The genome and transcriptome can be accessed through NCBI: SRP 059553.

**FIGURE 1.**
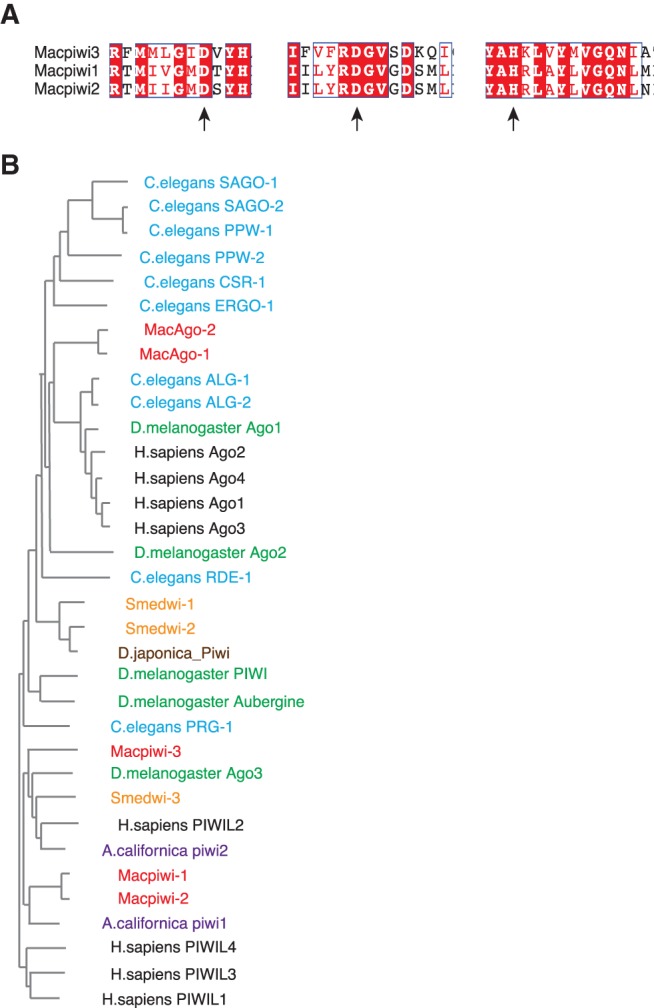
Ago/PIWI proteins are present in *M. lignano*. (*A*) Protein sequence alignment of Macpiwi1, Macpiwi2, and Macpiwi3 catalytic triads, DDH. Arrows point to the catalytic residues. Identical (white letters on a red background) or similar (red letters on a white background) residues are labeled. (*B*) A phylogenetic tree depicting the relationship between Ago/PIWI proteins in *M. lignano* (red), *C. elegans* (blue), *S. mediterranea* (orange), *D. melanogaster* (green), *A. californica* (purple), *Dugesia japonica* (brown), and *Homo sapiens* (black).

The canonical miRNA pathway, in addition to Argonaute proteins, includes RNase III proteins Drosha and Dicer (Ha and Kim 2014). We were able to identify all of these pathway components as well as other cofactors—DGCR8, TRBP, and Exportin-5 (Supplemental Table 1)—in the assembled genome and transcriptome. This has led us to the conclusion that a canonical miRNA pathway is likely to exist in *M. lignano*. Furthermore, Metazoans usually express multiple PIWI proteins with distinct functions in piRNA pathways. We searched for all potential PIWI proteins in the genome and the de novo transcriptome assemblies, and in addition to the previously identified *Macpiwi1* (De Mulder et al. 2009), we identified several candidate genomic loci coding for PIWI-like gene fragments. Only two others, *Macpiwi2* and *Macpiwi3*, however, were found as full-length transcripts. Macpiwi2 had a similar protein sequence (80% identity) to Mapiwi1, whereas Macpiwi3 was only ∼30% identical to Macpiwi1. All three Macpiwis had putative PAZ and PIWI domains. The PIWI domains included a catalytic triad (DDH) (Fig. 1A) that has been shown to be required for the cleavage of RNA targets (Song et al. 2004).

Phylogenetic analysis showed that Macpiwi proteins are most similar to PIWIs in the planarian *S. mediterranea*, and the mollusk *A. californica* (Fig. 1B). This is in agreement with the relatively close evolutionary relationship of *Macrostomum* sp. with planarians and mollusks (superphylum Lophotrochozoa) and much more distant relationship with Deuterostomes and Ecdysozoans (Egger et al. 2009, 2015). piRNA pathways are highly conserved across metazoan species and this conservation seems to extend to flatworms (Supplemental Fig. 1). Despite the relatively far phylogenetic distance, evidence for the majority of well-known fly and mammalian piRNA components (Ku and Lin 2014) could be found in the *M. lignano* genome and transcriptome (Supplemental Table 1). This suggests that a canonical piRNA pathway is likely to be operating in *M. lignano*.

### Abundant miRNA and piRNA populations are present in *M. lignano*

**FIGURE 2.**
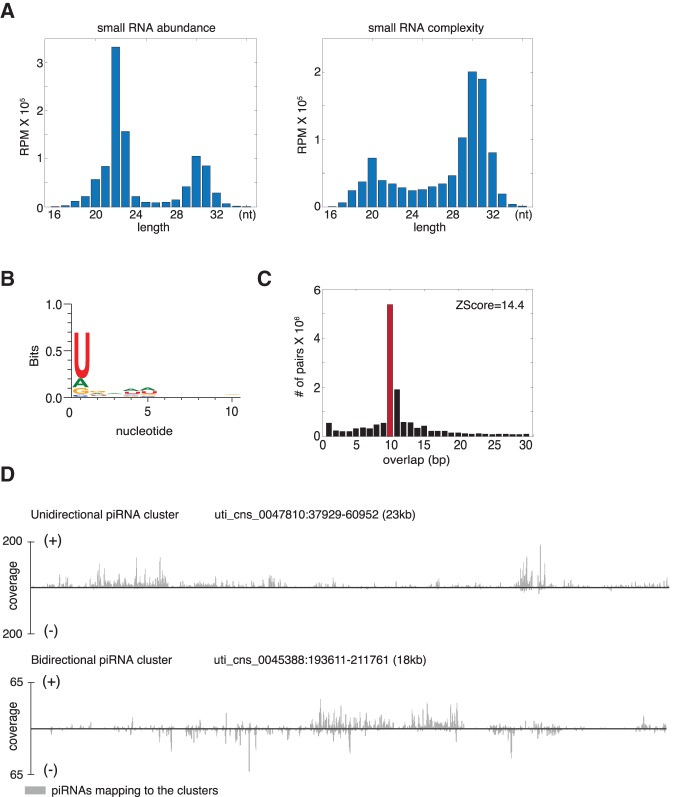
Characterization of *M. lignano* piRNAs. (*A*) Size distribution of putative piRNA population in *M. lignano.* Numbers of uncollapsed and collapsed reads per million total mapping reads (RPM) aligned to the genome are plotted on the *left* and *right*, respectively. (*B*) Sequence bias of the 28–32-nt long RNA population from whole worms. (*C*) Distribution of 5′ overlap between sense and antisense piRNA strands. *Z*-score represents the enrichment of 10-bp overlap. (*D*) Examples of unidirectional and bidirectional piRNA clusters from the genome draft. The piRNA coverage at each nucleotide position of the clusters is shown in gray.

To investigate the content of *M. lignano* small RNA populations, we cloned and sequenced small RNAs from adult worms. Small RNAs in *M. lignano* formed three characteristic populations varying in size, abundance (number of reads), and sequence complexity (number of unique sequences) (Fig. 2A). The population of ∼22-nt RNAs was very abundant with relatively low complexity while the ∼20-nt and ∼30-nt populations had higher sequence complexity but were less abundant.

Based on its size and low complexity, the 22-nt small RNA population likely represented canonical miRNAs. Indeed, 631 distinct miRNA sequences were found in *M. lignano* small RNAs with at least 1 read per million (rpm) total reads and 100% sequence identity to their counterparts in miRBase (Supplemental Table 2). Only those sequences within typical miRNA size range aligned with sequences in miRBase (Supplemental Fig. 2A).

As seen with previously characterized miRNAs, the *M. lignano* miRNA population showed a strong 5′ U bias (Supplemental Fig. 2C). Since miRNAs are known to derive from stem–loop structures, we used the UNAfold miRNA prediction tool (Markham and Zuker 2008) to identify potential precursor miRNA hairpin structures across the *M. lignano* genome. For 12 out of the 20 most abundant and highly conserved (100% identity to miRBase) miRNAs, we identified high-confidence hairpins that likely represent genes from which pri-miRNAs are expressed (Supplemental Fig. 2B). A number of conserved miRNAs were found to be differentially expressed in comparisons between sorted dividing cells (somatic and germline stem cells), and irradiated worms and during regeneration (Supplemental Fig. 2D; Supplemental Table 2).

In metazoans, piRNAs are highly complex 24–32-nt small RNAs with a strong U bias at their 5′ end. These primarily target transposable elements (TEs) (Brennecke et al. 2007; Senti and Brennecke 2010; Cora et al. 2014). In the planarian *S. mediterranea*, piRNA populations also share these signature features (Palakodeti et al. 2008). The ∼30-nt small RNA population in *M. lignano* was within the typical piRNA size range. It also exhibited a strong 5′U bias—another typical piRNA characteristic (Fig. 2B). Since in other species the principal targets of piRNAs are transposable elements, we mapped all 28–32-nt small RNAs to the *M. lignano* de novo transcriptome assembly. Among 10 transcripts that generated the greatest diversity of piRNAs, six were transposon-related (Supplemental Table 3); the remaining were unannotated or uncharacterized. The small RNAs that aligned to transposons showed key features of secondary piRNA biogenesis known as the ping-pong signature (Brennecke et al. 2007), namely, a 5′ 10-nt overlap with piRNAs from the opposite orientation (Fig. 2C).

piRNAs are usually generated from large genomic loci known as piRNA clusters (10–100 kbp) (Brennecke et al. 2007). To investigate whether putative *M. lignano* piRNAs were produced in a similar manner, we identified a set of 436 clusters in the *M. lignano* genome draft. These gave rise to 80.2% of all uniquely mapping piRNAs (Supplemental Table 4). These candidate piRNA clusters have a median length of 9.7 kbp, a maximum length of 93.7 kbp, and made up 0.63% of the genome. Among them, we found both unidirectional and bidirectional clusters (Fig. 2D). Thus, these piRNA clusters have characteristics similar to those of other species (Brennecke et al. 2007). The *M. lignano* genome is highly repetitive, potentially complicating the identification of piRNA-generating loci. We therefore took a second approach, identifying 3228 piRNA-generating transcripts with abundant piRNA production (Supplemental Table 5), reasoning that the extended nature of piRNA-generating transcripts might allow better genome mapping. Of all piRNA-producing transcripts, 1951 aligned within the 436 identified piRNA clusters, while the others were distributed throughout the remainder of the genome and could represent individual transposon copies or fragments. In summary, *M. lignano* piRNAs are in many respects similar to piRNAs that are well characterized in other species.

### The *M. lignano* piRNA pathway uses multiple PIWIs

**FIGURE 3.**
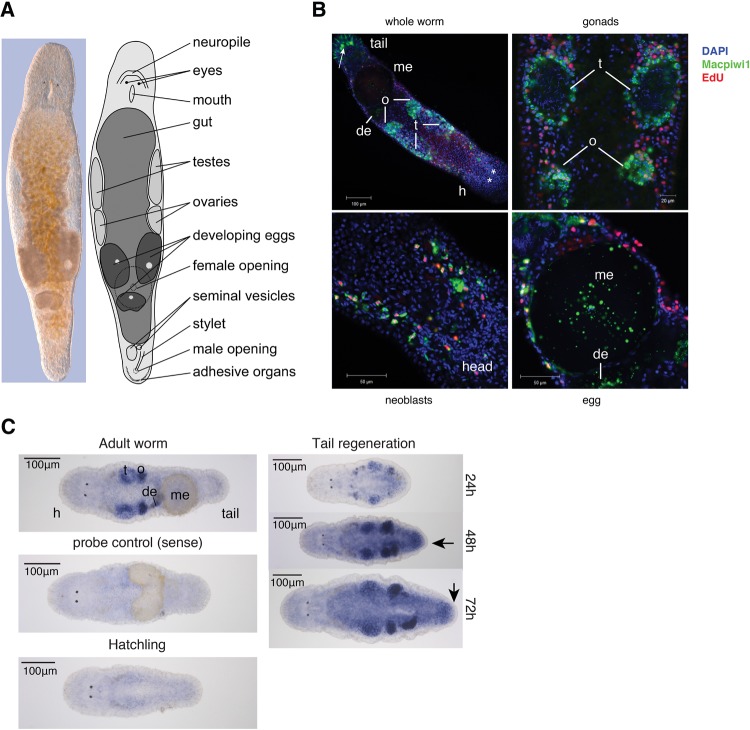
Expression patterns of *Macpiwi1* and *Macpiwi2* in *M. lignano.* (*A*) Interference contract image and diagrammatic representation of an adult worm. (*B*) Immunofluorescence labeling showing localization of Macpiwi1 (green) in adult worms. Dividing cells are labeled with EdU (red). (h) Head, (t) testis, (o) ovary, (de) developing egg, (me) mature egg. Stars denote eyes. Arrow points to nonspecific staining by the secondary antibody. (*C*) Localization of *Macpiwi2* mRNA by whole-mount in situ hybridization in adult worms, hatchlings, and during regeneration induced by posterior amputation. Sense riboprobe was used as negative control. Arrows point to blastemas during regeneration. (t) Testis, (o) ovary, (de) developing egg, (me) mature egg.

PIWI proteins are predominantly expressed in the germline (Ku and Lin 2014). In *M. lignano*, however, the expression of Macpiwi1 indicates that PIWI proteins are expressed in multiple cell types, including in the cytoplasm of gonadal cells, developing and mature eggs, as well as dividing somatic neoblasts (Fig. 3A,B; De Mulder et al. 2009). *Macpiwi2* in situ hybridization shows expression in gonads and in eggs at early stages of development (Fig. 3C). An additional parenchymal staining pattern suggests the presence of *Macpiwi2* also in the neoblasts. Following posterior amputation, *Macpiwi2* expression was increased in the blastema during regeneration.

Since Macpiwi1 and 2 were enriched in dividing neoblasts, we asked whether piRNAs were also present in these cell types. We isolated two cell populations from adult worms based on their DNA content (2*N* or 4*N*), reasoning that the only 4*N* cells in the organism would be those in G2/M phase (Supplemental Fig. 3A). This strategy was based upon the understanding that somatic and germline stem cells are the only abundant, dividing cell types in *M. lignano* (De Mulder et al. 2010). piRNAs were clearly enriched in the 4*N* dividing cell population (neoblast and germline stem cells); however, a subset of piRNAs present in the 4*N* cell population was also detectable in the 2*N* fraction (Supplemental Fig. 3B). This could have represented piRNAs in either germ cells or neoblasts that were in G1/S phase at the time of harvest, or nondividing cell types. To investigate these possibilities, we analyzed the piRNA composition of lethally irradiated worms. Even after a lethal dose of γ radiation and despite the absence of EdU-positive dividing cells and Macpiwi1-positive cells (Supplemental Fig. 3C), piRNAs remained detectable in whole worms (Supplemental Fig. 3D). Considered as a whole, our data indicate that Macpiwi proteins and piRNAs are likely to exist in cell types other than previously characterized germline and stem cell populations.

### Macpiwi1 participates in a heterotypic ping-pong cycle

**FIGURE 4.**
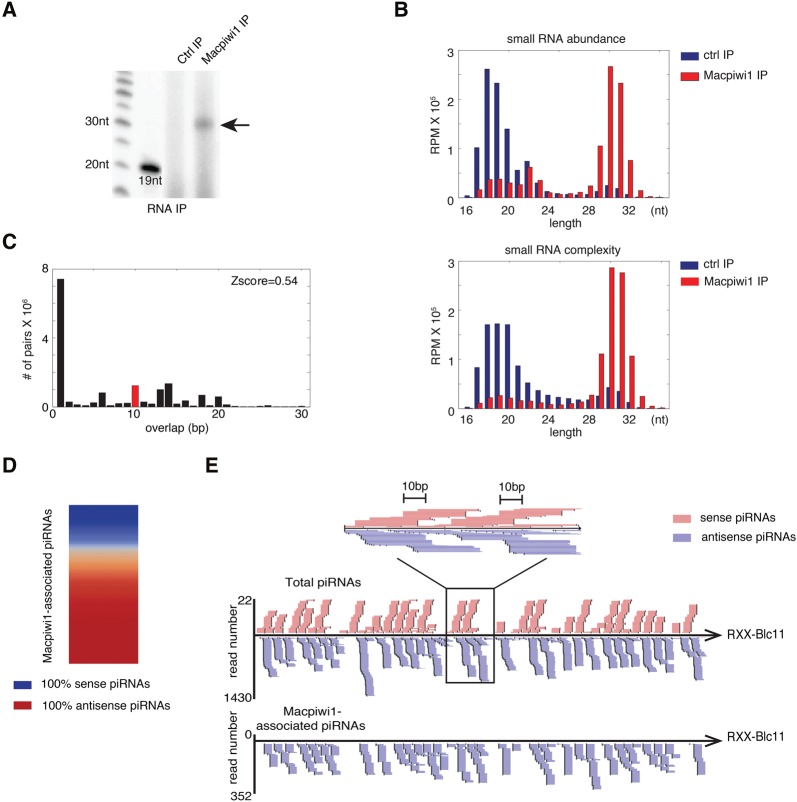
Characteristics of Macpiwi1-associated piRNAs. (*A*) Arrow points to 5′-end radiolabeling of Macpiwi1-associated small RNAs. (*B*) Length distribution of sequence abundance and complexity of Macpiwi1-associated small RNAs. Numbers of uncollapsed and collapsed reads per million total mapping reads (RPM) that aligned to the genome are plotted. (*C*) Distribution of 5′ overlaps between sense and antisense strands of Macpiwi1-associated piRNAs. (*D*) A heatmap depicting the strand bias of Macpiwi1-associated piRNAs from each transposon consensus sequences. (*E*) An example of piRNA coverage on a transposon consensus sequence, RXX-Blc11. piRNAs mapping in sense and antisense orientations are labeled in pink and purple, respectively. A close-up view shows ping-pong signature characterized by a 10-bp overlap between sense and antisense strands.

PIWI proteins act in concert with piRNAs. We therefore tried to determine whether specific populations of piRNAs associated with any of the three Macpiwi proteins that were identified. We generated antibodies directed against each protein, but could only detect small RNAs in Macpiwi1 immunoprecipitates (Fig. 4A,B). Failure to detect associated RNAs with either Macpiwi2 or Macpiwi3 may be simply attributable to the technical qualities of the antibodies.

Of the 3228 piRNA-producing transcripts identified, 3093 contributed to Macpiwi1-associated piRNAs. This suggests that Macpiwi1 interacts with the majority (judged by sequence diversity) of all piRNAs in *M. lignano*. In contrast to piRNAs cloned from the whole worms (total piRNAs) (Fig. 2C), *Macpiwi1*-associated piRNAs did not display ping-pong signatures (Fig. 4C) and were predominantly oriented antisense to transposons (Fig. 4D). In small RNAs from whole worms we did note the presence of sense-oriented piRNAs, which formed ping-pong pairs with antisense piRNAs (Fig. 4E). These sense-oriented piRNAs were absent from Macpiwi1-associated populations, suggesting their preferential association with another PIWI protein. Uniquely mapping piRNAs are enriched in Macpiwi1 complexes, with one-third of piRNAs associated with this protein assignable to discrete genomic locations, in contrast to one-fourth in the total piRNA population (Supplemental Table 6). Considered together, these data suggest that Macpiwi1 binds primary piRNAs to initiate a heterotypic ping-pong cycle with at least one other Macpiwi, which serves as the host for sense piRNAs.

### Macpiwi1 and the piRNA pathway mediate transposon silencing

**FIGURE 5.**
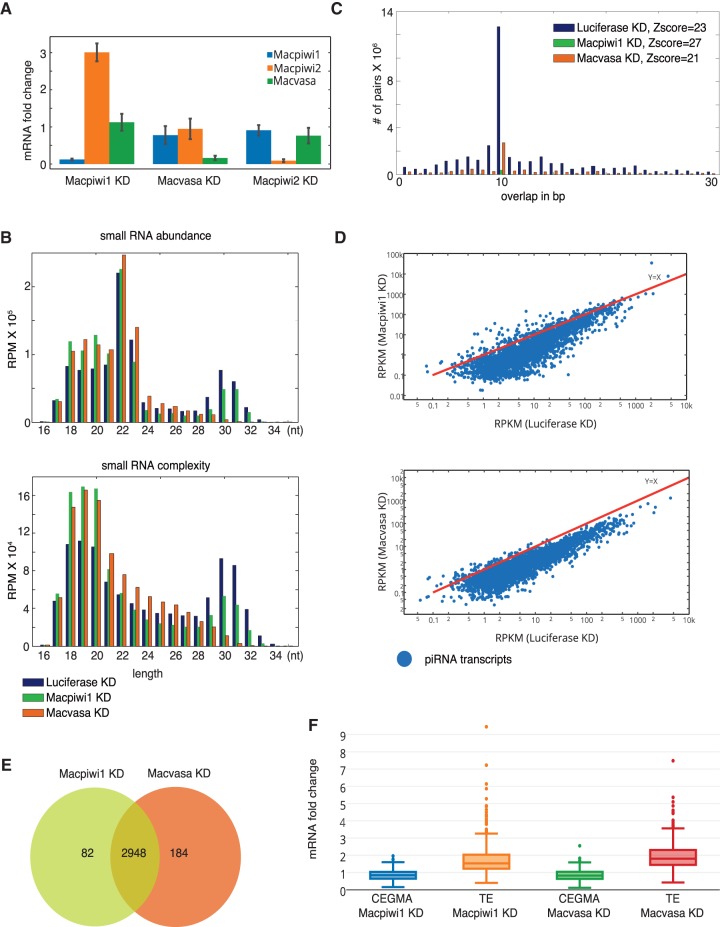
*Macpiwi1* and *Macvasa* silencing leads to piRNA downregulation and transposon derepression. (*A*) Quantitative PCR showing RNAi knockdown efficiency as compared to *luciferase* dsRNA. (*B*) Length distribution plotted against sequence abundance and complexity of small RNAs from *luciferase*, *Macpiwi1*, and *Macvasa* knockdown (KD) worms. Numbers of uncollapsed and collapsed reads per million total reads (RPM) aligned to the genome are plotted. (*C*) Distribution of 5′ overlaps between piRNAs from sense and antisense strands in *Macpiwi1*, *Macvasa*, and *luciferase* KD. (*D*) Read counts of piRNAs mapped to individual transposon-related transcripts are plotted for l*uciferase* KD and *Macpiwi1* or *Macvasa* KD worms. Each dot represents a transposon consensus sequence. Axes are presented on log_10_ scale. (RPKM) Reads per kilo base pair per million. (*E*) A Venn diagram showing numbers of transcripts with decreased piRNA production and the overlap of those between *Macpiwi1* KD and *Macvasa* KD. (*F*) mRNA fold change of CEGMA core gene set and transposon consensus sequences in *Macpiwi1* KD and *Macvasa* KD, compared with *luciferase* KD.

To determine the effects of depleting individual *M. lignano* piRNA pathway components on overall piRNA and mRNA levels, we knocked down *Macpiwi1*, *Macpiwi2*, and *Macvasa* using RNAi (Fig. 5A). Vasa is a DEAD-box RNA helicase required for germline piRNA biogenesis in flies and mammals (Kuramochi-Miyagawa et al. 2010; Zhang et al. 2012). In *D. melanogaster*, Vasa localizes at the nuclear envelope and is thought to deliver piRNA transcripts to cytoplasmic piRNA machinery (Liang et al. 1994; Zhang et al. 2012). In *M. lignano*, the *vasa* homologue, *Macvasa*, is expressed in two isoforms in the germline and in somatic stem cells (Pfister et al. 2008).

Depletion of *Macpiwi1* or *Macvasa* (both isoforms) affected both piRNA levels and mRNA expression. Knockdown of *Macpiwi1* caused a decline in piRNA abundance and the overall complexity of piRNA populations, as compared with a control knockdown (Fig. 5B). Effects of *Macvasa* knockdown on piRNA levels were even more dramatic—the piRNA population became essentially undetectable in treated worms (Fig. 5B). The number of ping-pong pairs was drastically decreased in both knockdowns (Fig. 5C), probably due to overall piRNA depletion. Any remaining ping-pong signature was likely a result of a small amount of residual protein expression. Both *Macpiwi1* and *Macvasa* knockdowns led to a global decrease in piRNA levels from piRNA-producing transcripts determined from analysis of wild-type animals (Fig. 5D,E).

An examination of the transcriptome profiles indicated transposon derepression in both *Macpiwi1* and *Macvasa* knockdowns (Fig. 5F), whereas the set of CEGMA protein-coding genes was not affected in either knockdown. This confirms that piRNAs from *M. lignano* preferably target transposable elements and mediate transposon silencing, as has been seen in other species where the piRNA pathway has been studied.

In contrast to *Macpiwi1*, *Macpiwi2* knockdown did not result in any detectable morphological change (Supplemental Fig. 5A), substantial change in piRNA abundance or complexity (Supplemental Fig. 4A), ping-pong signature (Supplemental Fig. 4B), or overall transposon expression (Supplemental Fig. 4C). This strongly suggested that Macpiwi2 was not the exclusive receiver protein in a Macpiwi1-driven heterotypic ping-pong cycle. These data provoke the notion that Macpiwi2 either binds the same set of piRNAs as Macpiwi1 and can be completely compensated by the presence of the latter protein, or that it shares piRNA preference with yet another PIWI protein and can be compensated by that protein. Alternatively, Macpiwi2 might bind a population of RNAs that are not obvious in our RNA-seq libraries.

### Macpiwi1 is essential for stem cell maintenance

**FIGURE 6.**
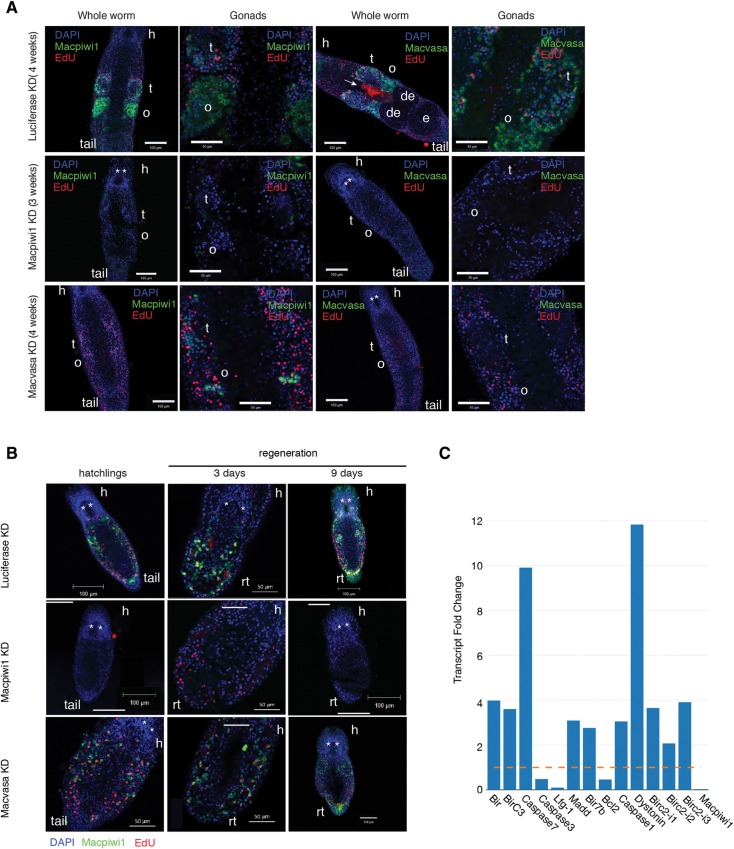
Depletion of Macpiwi1 but not Macvasa results in stem cell failure. (*A*) Macpiwi1 and Macvasa immunostaining after *luciferase* KD (4 wk), *Macpiwi1* KD (3 wk), and *Macvasa* KD (4 wk) in adult worms. EdU-positive cells are shown in red. (t) Testis, (o) ovary, (de) developing egg, (e) egg, (h) head. Stars denote eyes. The arrow denotes nonspecific staining of diatoms in the gut. (*B*) Macpiwi1 immunostaining in 1-wk-old hatchlings, regenerating worms 3 and 9 d post anterior amputation. EdU-positive cells are shown in red. (h) Head, (rt) regenerating tail. Stars denote eyes. (*C*) Fold change of differentially expressed apoptosis-related transcripts in *Macpiwi1* KD, normalized to *luciferase* KD. (Yellow dashed line) Fold change = 1. *Macpiwi1* is plotted as a control.

In all other species studied so far, loss of the piRNA pathway components leads to transposon derepression and sterility (Klattenhoff et al. 2007; Klattenhoff and Theurkauf 2008). Given their expression patterns, when silencing *Macpiwi1*, *Macvasa*, and *Macpiwi2*, we paid particular attention to effects on dividing cell populations. To track changes in the germline and neoblasts, we stained for Macpiwi1 and Macvasa proteins and labeled dividing cells by EdU incorporation. As reported previously (De Mulder et al. 2009), Macpiwi1 is present in both germline and neoblasts, where it is essential for stem cell maintenance and functions in adult homeostasis, regeneration, and postembryonic development. In accord with prior studies, silencing of *Macpiwi1* caused defects in adult worms (Fig. 6A), hatchlings, and regenerating worms (Fig. 6B). Macpiwi1 protein was undetectable in *Macpiwi1*-depleted adult worms within 2 wk of treatment with dsRNA (Supplemental Fig. 5B). By 3 wk following silencing of *Macpiwi1* in adults, EdU-positive cells were eliminated and gonads degenerated (Fig. 6A; Supplemental Fig. 5A). In hatchlings, *Macpiwi1* knockdown resulted in retarded development and in failure of gonad formation (Fig. 6B), and EdU-positive cells were dramatically decreased in 9-d-old hatchlings (Fig. 6B). *Macpiwi1* silencing also resulted in severe defects in regeneration. Three days after anterior amputation, EdU-positive cells accumulated at the blastema, however, stem cells failed to develop into new tissues (Fig. 6B). Nine days post-amputation, EdU-positive cells were undetectable and regeneration did not progress (Fig. 6B). Prolonged exposure to *Macpiwi1* RNAi eventually led to mortality in all of the above conditions, most likely due to the lack of stem cells whose activity is required for tissue maintenance.

*Macvasa*-knockdown worms suffered an even more dramatic piRNA loss and a greater increase in transposon expression than did *Macpiwi1*-knockdown animals (Fig. 5B), yet the phenotypes of these animals were dramatically different. Despite substantive piRNA loss, *Macvasa*-knockdown worms failed to show phenotypes expected for stem cell defects in adults, hatchlings, or regenerating worms within 3 wk of RNAi treatment (Fig. 6A,B). This was consistent with one previous study of *Macvasa* knockdown (Pfister et al. 2008). After 3 wk of RNAi treatment, we noticed a gradual decrease of Macpiwi1 staining in *Macvasa* knockdown animals (Fig. 6A), indicating either decreased expression, stability, or delocalization of the Macpiwi1 protein. Mortality and morphological changes (Supplemental Fig. 5A) occurred in some worms during the fourth week of *Macvasa* RNAi treatment. EdU-positive cells were, however, constantly present in *Macvasa* knockdown worms in all conditions, even after piRNAs underwent a depletion more severe than that in *Macpiwi1* knockdown worms (Fig. 6A). Differential expression analysis identified 16 apoptosis-related genes that were increased upon *Mapiwi1* but not *Macvasa* knockdown (Fig. 6C). The activation of apoptosis might be a strong contributor to stem cell failure in *Macpiwi1*-knockdown worms. Considered as a whole, our data could suggest that Macpiwi1 has functions in stem cell maintenance that are distinct from its roles in transposon repression. *Macvasa* knockdown had profound effects on piRNA populations but did not seem to disrupt the stem cell maintenance function of Macpiwi1. This raises several interesting possibilities. Macpiwi1 could maintain stem cells in a piRNA-independent fashion, and such piRNA-independent activities have been suggested for PIWI proteins in other organisms (Klenov et al. 2011). Alternatively, those small RNAs needed for stem cell maintenance functions may not depend upon Macvasa for their production and could form a piRNA population that is either not readily identified in our sequencing data or could be modified in a fashion that makes them less amenable to capture by our cloning protocol.

## DISCUSSION

PIWI proteins are well established as being essential for transposable element silencing in many animal species, including *D. melanogaster*, *Mus musculus*, and *H. sapiens* (Juliano et al. 2011)*.* Here, we show that one of *M. lignano* PIWI proteins, Macpiwi1, has essential roles in transposon repression, analogous to those documented in other animals.

PIWI proteins in *M. lignano* appear to diverge from those in other flatworm species (Fig. 1B), despite the relatively close phylogenetic relationship. In a recent study revising the phylogeny of flatworms (Laumer et al. 2015), *M. lignano* was shown to occupy a very basal position among Platyhelminthes, corroborating the commonly believed primacy of *M. lignano* among flatworms. Given the basal position of *M. lignano*, understanding the *M. lignano* piRNA pathway might shed some light on the divergence of piRNA pathways within Platyhelminthes and Metazoans.

Like other metazoans, *M. lignano* has multiple PIWI proteins, Macpiwi1, 2, and 3, and an abundant piRNA population that appears enriched for transposon sequences. Based on our attempts to clarify the hierarchy of Macpiwis, several observations imply that Macpiwi1 is the binding partner for what are likely primary piRNAs that subsequently enter the secondary amplification loop in a heterotypic ping-pong cycle. However, it remains unclear which additional Macpiwi protein(s) cooperate with Macpiwi1 in ping-pong amplification. *Macpiwi2* knockdown fails to abolish piRNA production, although there are some groups of differentially expressed piRNAs (Supplemental Fig. 4D), and slightly more repressed transposon expression (Supplemental Fig. 4C) suggests participation of other Macpiwis also. Nevertheless, the weakened ping-pong signature in *Macpiwi2* knockdown animals (Supplemental Fig. 4B) poses it as one possible ping-pong partner for Macpiwi1.

In some settings, PIWI proteins also play essential roles in stem cell maintenance. In *M. lignano*, this is also the case, both for maintenance of the germline and for sustaining populations of totipotent stem cells, the neoblasts. In *M. lignano*, stem cell maintenance functions for Macpiwi1 appear to be separable from its role in transposon repression, based on our analysis of *Macvasa* knockdown worms. Distinctions between transposon repression and stem cell maintenance functions have also been made in other organisms. In *D. melanogaster* (Klenov et al. 2011), *Piwi*-null mutants display activation of transposons and severe disruption of germline stem cell maintenance. A Piwi mutant lacking its nuclear localization signal is capable of sustaining stem cell self-renewal, even though it fails to repress transposons in the bulk of the germline. Additionally, mutation of the nuclease that processes primary piRNA precursors, Zucchini, prevents loading of primary piRNAs into Piwi, but does not interfere with germline stem cell maintenance (Klenov et al. 2011). Here we find that *Macvasa*-knockdown worms, which lack the vast majority of piRNAs, are fully capable of stem cell division, regeneration, and postembryonic development, despite transposon activation. Whether this indicates that Macpiwi1 functions in stem cell maintenance without the need to bind any small RNA partner, however, remains to be determined.

## MATERIALS AND METHODS

### Animal culture, regeneration, γ-irradiation, and RNA isolation

*Macrostomum lignano* was kept in petri dishes with nutrient-enriched f/2 medium and fed ad libitum with diatoms (*Nitzchia curvilineata*) (Andersen et al. 2005) Climate chamber conditions were set at 20°C, 60% humidity, and a 14/10 h day/night cycle. For regeneration, worms were cut at the post-pharyngeal level in order to completely remove gonads. The anterior part was kept under normal conditions with diatoms. γ-Irradiation was performed as previously described (De Mulder et al. 2010). Of note, 200–400 worms were resuspended in TRIzol reagent (Life Technologies) for RNA extraction according to the manufacturer's instruction.

### Cloning of *Macpiwi1* and *Macpiwi2*

The *Macpiwi1* full-length coding sequence was published previously (De Mulder et al. 2009). In order to obtain a full-length mRNA sequence, 5′ and 3′ RACE were performed using SMARTer RACE cDNA amplification kit (Clontech). Primer sequences used: 5′-CGACACGTCAACATGCAGCATCAGAG-3′ (5′RACE) and 5′-GAGGACGTGAATGACGCCAACATCAA-3′ (3′RACE). A partial *Macpiwi2* sequence was identified from the de novo transcriptome assembly (KA Wasik, J Gurtowski, X Zhou, OM Ramos, MJ Delás, O El Demerdash, G Battistoni, I Falciatori, DB Vizoso, AD Smith, et al., in prep.). 5′ and 3′ RACE were performed in order to obtain the full-length transcript sequence. Primers sequences are: 5′-CTCGGTCCTGCATCACGGGCAGCACGTA-3′ (5′RACE) and 5′-AAAGTCGCTCCGTGCAGGGTGTGGTGTT-3′ (3′RACE). The PCR fragments were cloned using Zero Blunt TOPO PCR cloning kit (Life Technologies) for sequencing.

### Reverse transcription and quantitative PCR

Reverse transcription was performed using 2 μg of total RNA, oligo(dT), and SuperScript III reverse transcriptase (Life Technologies) following the manufacturer's instruction. *Macpiwi1*, *Macpiwi2*, and *Macvasa* expression levels were measured using Sybr-green PCR on Eppendorf Realplex thermal cycler. β-Actin was used as a housekeeping gene for normalization. PCR primers used:

Actin forward: 5′-CGTGACCTCACCGACTACCT-3′,
Actin reverse: 5′-GGGCAGCTCGTAGCTCTTCT-3′,
Macpiwi1 forward: 5′-AGGCCATTGTGGTGAAGAAG-3′,
Macpiwi1 reverse: 5′-ACTGCGACACCAGGAAGAAG-3′,
Macpiwi2 forward: 5′-GCTGCACCTGATGAATGTTG-3′,
Macpiwi2 reverse: 5′-TTCGACGGATCCAGGTAAAG-3′,
Macvasa forward: 5′-GCTTCATGGACTCGGTGACT-3′,
Macvasa reverse: 5′-GGCCGAGAACATCACAATCT-3′.

### Whole-mount in situ hybridization of *Macpiwi2*

Whole-mount in situ hybridization was performed as previously described (Pfister et al. 2007) with modifications (Lengerer et al. 2014). The template DNA for DIG-labeled in situ probe synthesis was made using Q5 High-Fidelity DNA Polymerase (New England Biolabs). Color development was carried out at 37°C for 1 h and at 4°C for 80 min. Primer binding sites used for template DNA synthesis were 5′-AGCTTCTGGCTTCGGGTATC-3′; 5′-CAGATCGATGTCGTAAGTCTGC-3′.

### Immunofluorescence and labeling of S-phase cells

Polyclonal Macpiwi1 antibody was produced by PrimmBiotech by rabbit immunization with the peptide RPAPPPGLSAQAG (position 44–56 amino acids). The antibody was purified from serum using synthetic peptides and the sulfolink immobilization kit (Thermo Scientific) according to manufacturer's instructions. Macpiwi1 and Macvasa staining was performed as previously described (Pfister et al. 2008; De Mulder et al. 2009). For double staining of S-phase cells and Macpiwi1 or Macvasa, worms were soaked in 5 mM EdU for 30 min. EdU-positive cells were labeled using click-iT cell reaction buffer kit (Life Technologies) and Alexa Fluor 594 azide (Life Technologies), according to the manufacturer's instructions, after the secondary antibody reaction. Nuclei were stained with DAPI (5 μg/mL) (Sigma-Aldrich) at room temperature for 15 min. Specimens were mounted with prolong gold antifade reagent (Life Technologies) for imaging. Images were captured using Zeiss LSM 710 confocal microscope.

### RNA interference

RNAi knockdown of *Macpiwi1* and *Macvasa* was performed as previously described (Pfister et al. 2008; De Mulder et al. 2009). dsRNA was synthesized using the T7 Ribomax express RNAi system (Promega). Worms were soaked in f/2 medium containing 4 μg/mL of dsRNA probes and 35 μg/mL of antibiotics (Ampicillin and Kanamycin alternately every other day). Medium was changed twice a day. Before amputation, whole worms were presoaked for 2 wk. Eggs were soaked as soon as they were laid. Worms were fed on diatoms throughout the entire experiment. Three biological replicates were performed for each knockdown condition. Approximately 300 worms were used for each replicate. Each biological replicate was used for all downstream applications including immunostaining (20 worms each staining), small RNA cloning, mRNA sequencing, and RT-qPCR.

### Immunoprecipitation, RNA end labeling, and Western blot

Approximately 10,000 worms were collected and lysed in worm lysis buffer 2.0 (20 mM HEPES, 150 mM NaCl, 2 mM EDTA, 2 mM EGTA, 0.1 M PMSF, 0.5% NP-40, 0.5% Triton X-100, 10% glycerol, 1 mM DTT, cOmplete mini protease inhibitor cocktail [Roche], 100 units/mL RNasin RNase inhibitor [Promega]) using a dounce homogenizer. Crude lysate was centrifuged at >12,000*g* at 4°C for 15min. The supernatant was transferred to a new tube while the pellet was washed with worm lysis buffer 2.0 and centrifuged again. Supernatants from the two centrifugations were combined and the volume was brought up to 0.5 mL using worm lysis buffer 2.0. Polyclonal Macpiwi1 antibody or pre-immune rabbit serum (1:10) was added to cleared lysate and incubated at 4°C overnight under rotation. Fifty microliters of protein A agarose beads (Roche) blocked with 5% BSA was added and incubation was carried out at 4°C for 4 h. Beads were spun down and washed in NT2 buffer (50 mM Tris pH 7.4, 150 mM NaCl, 1 mM MgCl_2_, 0.05% NP40, 100 units/mL RNasin, 1 mM DTT) at 4°C with a series of increasing salt concentrations (150 mM NaCl, 2 × 10 min; 300 mM NaCl, 2 × 10 min; 500 mM NaCl, 2 × 10 min). After washing, beads were spun down and digested with 2 μg/μL of proteinase K in 200 µL of proteinase K buffer (200 mM Tris–HCl pH 7.5, 25 mM EDTA, 300 mM NaCl, 2% SDS) at 65°C for 1 h. RNA was extracted using equal volumes of acidic phenol:chloroform and chloroform sequentially, followed by ethanol precipitation at −20°C overnight.

For end labeling of RNA, one-tenth of the RNA isolated was treated with CIP (New England Biolabs) at 37°C for 30 min before labeled using [γ-^32^P] ATP (Perkin Elmer) and PNK (New England Biolabs) at 37°C for 1 h. Labeled RNA was visualized on a 12% polyacrylamide gel.

For Western blotting of Macpiwi1, protein samples were resuspended in Laemmli buffer, boiled for 5 min, and run on 10% SDS-PAGE. Proteins were transferred onto a PVDF membrane using a wet transfer method at 35 V overnight. Membranes were blocked in 5% fat-free milk at room temperature for 1 h and incubated with polyclonal Macpiwi1 antibody (1:200) at 4°C overnight. Membranes were washed in TBS-T (3 × 5 min) and incubated with HRP-conjugated anti-rabbit IgG secondary antibody (Cell Signaling) at room temperature for 1 h. After washes in TBS-T (5 × 5 min), Amersham ECL prime detection reagent was used for detection.

### S-phase cell sorting

At least 10,000 worms were collected and relaxed in F/2-7,14% MgCl_2_ (1:1) at room temperature for 10 min. Relaxed worms were washed in CMFM (88 mM NaCl, 1 mM KCl, 2.4 mM NaHCO_3,_ 7.5 mM Tris–HCl [pH 7.6]) on ice (3 × 5 min). Worms were trypsinized with 1% Trypsin in CMFM at 37°C for 20 min with agitation. An equal volume of maceration solution (glacial acetic acid:glycerol:H_2_O 1:1:13, 9% sucrose) was added and incubated at room temperature for 1 min. Cells were spun down at 5000*g*, 4°C for 10 min, resuspended in PBS with 5% RNase inhibitor and incubated on ice for 5 min. Centrifugation was repeated and cells were resuspended and incubated in 0.5 mg/mL lysolecithin (Sigma-Aldrich) in PBS with 5% RNase inhibitor at room temperature for 10 min. Cells were blocked with 2% BSA on ice for 5 min and let recover in 500 µL 2% FBS in PBS for 10 min at 4°C. Propidium iodide (20 μg/mL) (Sigma-Aldrich) was added to cell suspension and incubated on ice for 30 min. Cells were sorted using an Aria IIU cell sorter (BD biosciences) and directly mixed with TRIzol LS reagent (Life Technologies) for RNA extraction.

### Small RNA and mRNA sequencing

Small RNAs with 5′ phosphate and 3′ hydroxyl group were cloned using a previously published method (Lau et al. 2001). Of note, 1–10 μg of total RNA was used as input. RNA-seq libraries were generated using Encore Complete RNA-Seq DR Multiplex System (Nugen) according to manufacturer's instruction. Samples were sequenced on Illumina Hiseq 2000. All the raw sequencing data are available in the Sequencing Read Archive (SRA) under accession number SRP059454.

### Small RNA sequencing data analysis

Three biological replicates were generated for each experiment, except for irradiated worms for which two replicates were generated. All sequence alignment against the genome draft, de novo transcriptome, or transposon consensus was performed using Bowtie (Langmead 2010), allowing up to two mismatches. Samtools (Li et al. 2009) and BEDTools (Quinlan and Hall 2010) were used for downstream processing, including read count and strand bias calculations from each genomic location. BAM files generated by Bowtie were loaded into the Integrative genomics viewer (IGV) to display read alignments and coverage. To examine the ping-pong signature, we used the ping-pong tool from piPipes (Han et al. 2015) on piRNA sequences. Nucleotide bias of piRNAs and microRNAs was displayed using Weblogo 3.0 (Crooks et al. 2004) command line interface.

The identification of piRNA clusters (Brennecke et al. 2007) and piRNA-producing transcripts (Li et al. 2013) was done according to previously published methods with adjustments. All collapsed and uniquely mapped 28–32-nt small RNA reads were aligned to the genome draft or de novo transcriptome. For piRNA cluster identification, numbers of reads were counted in 1-kb sliding windows with a step size of 968 nt. Windows containing at least 10 reads were merged and defined as piRNA clusters. Transcripts with at least 100 RPKM or PPM of mapping reads were qualified as piRNA-producing transcripts.

To generate transposon consensus sequences, we used the TEdenovo pipeline from REPET package (Flutre et al. 2011). Due to the highly repetitive nature of the *M. lignano* genome, only RepeatMasker-annotated repeats (Smit et al. 2013–2015) and piRNA clusters were used as input. Low complexity repeats and microsatellites were excluded from the final consensus sequences.

### Differential expression analysis of RNA-seq data

For differential expression of genes, paired-end reads were processed using RSEM (Li and Dewey 2011) with de novo transcriptome assembly. Differentially expressed genes (FDR ≤0.05, fold change ≤0.5 or ≥2) were identified using EBSeq (Leng et al. 2013). Expression change of transposons was analyzed on transposon consensus sequences according to the strategy described previously (Le Thomas et al. 2013). Reads were aligned to transposon consensus with Bowtie allowing multiple mapping and up to three mismatches. Read count for each transposon was calculated with normalization for length of consensus sequence, number of aligned locations of each read, and total number of reads aligned to the whole genome. Three biological replicates of each condition were generated for statistical analysis.

### Phylogenetic tree and protein alignment

Phylogenetic tree of PIWI proteins was generated using Clustal Omega. The length of branch represents amount of genetic change. Protein sequence alignment was performed using ESPript 3.0.

## SUPPLEMENTAL MATERIAL

Supplemental material is available for this article.

## Supplementary Material

Supplemental Material
