# Supplementary material for: Dual functions of Macpiwi1 in transposon silencing and stem cell maintenance in the flatworm Macrostomum lignano
Source: RNA. 2015 Nov;21(11):1885–97. doi: 10.1261/rna.052456.115 (PMC4604429; doi:10.1261/rna.052456.115)
Supplement: Supplemental Material [file supp_21_11_1885__index.html]

Dual functions of Macpiwi1 in transposon silencing and stem cell maintenance in the flatworm Macrostomum lignano — Dual functions of Macpiwi1 in transposon silencing and stem cell maintenance in the flatworm Macrostomum lignano — Supplemental Material 

# Dual functions of Macpiwi1 in transposon silencing and stem cell maintenance in the flatworm *Macrostomum lignano*

## Supplemental Material

**Files in this Data Supplement:**

- Supp Figure 1.pdf
- Supp Figure 2.pdf
- Supp Figure 3.pdf
- Supp Figure 4.pdf
- Supp Figure 5.pdf
- Supp Legends & Tables.docx
