# Supplementary material for: Dual functions of Macpiwi1 in transposon silencing and stem cell maintenance in the flatworm Macrostomum lignano
Source: RNA. 2015 Nov;21(11):1885–97. doi: 10.1261/rna.052456.115 (PMC4604429; doi:10.1261/rna.052456.115)
Supplement: Supplemental Material [file supp_052456.115_Sup_Fig1.pdf]

```
                        1        10        20        30        40        50        60
Smedwi-1                NLQKNLGTVLKREPRERLNDIRDFCKWTGAGKS.KDYMKSWGMCIEEQPITIRGRELAPVDIFSNEVKI..
Smedwi-2                DLQKNLGCVLKREPRERLDDIPAYCNWIKNSDAATGMCNKWQLKIDNKPLEIEGRELPPCDVISGGSKI..
D.japonica_Piwi         .......................................................................
Macpiwi-3               RLMRDLAKFTKLSPDRRLMELRKFVQTINNRPECRQLLDAWGLRLLEEPEVVPARRFVNERVLFGGGGRGP
D.melanogaster_PIWI     QLMRAMSSYTRMNPKQRTDRLRAFNHRLQNTPESVKVLRDWNMELDKNVTEVQGRIIGQQNIVFHNGKV..
D.melanogaster_Aubergine RTLRAMSEHTRLNPDRRIERLRMFNKRLKSCKQSVETLKSWNIELDSALVEIPARVLPPEKILFGNQKI..
Macpiwi-1               NLMKDLATFTRIDPSTRCGKLVKFASTMAQNRECQRVLGEFGLAVAPNAVEATARQLAPETVQMTKPIR..
Macpiwi-2               NLMRDLATHTRVNPSARCDKMLTFARTMAGNPESRRVLGEFGLEVSPNAVEATARQLQPETVQMSRPIR..
H.sapiens_PIWIL4        QLMKAVAEKTRLSPSGRQQRLARLVDNIQRNTNARFELETWGLHFGSQ.ISLTGRIVPSEKILMQDHIC..
A.californica_piwi1     .......................................................................
H.sapiens_PIWIL3        SIMKELAKHTRLSPRRRHHTLKEFINTLQDNKKVRELLQLWDLKFDTNFLSVPGRVLKNANIVQGRRMV..
Smedwi-3                RLMRELHEHCRVTPKKRHEALLEFVDNIYSCEEAKKLLGYWGITIEKDTVNINACKMNPEMIYFGNEAS..
H.sapiens_PIWIL2        RAMKDLAQQINLSPKQHHSALECLLQRIAKNEAATNELMRWGLRLQKDVHKIEGRVLPMERINLKNTSF..
H.sapiens_PIWIL1        NVMKDLAVHTRLTPEQRQREVGRLIDYIHKNDNVQRELRDWGLSFDSNLLSFSGRILQTEKIHQGGKTF..
A.californica_piwi2     .......................................................................
```

```
                                70        80        90       100       110       120
Smedwi-1                ....NTKIPDDWKFGEIK...FDVPKGTAHRFGVLVVDRNPSHFNNFIEDVKREIGRLRINYTMDSI...S
Smedwi-2                ....NEKMGDDWKFGRVQ...FDIKRDRKHEIDVVIVDRNDFQYKNFMNDVEQELRNMRIDARVGKV...N
D.japonica_Piwi         .......................................................................
Macpiwi-3               FAPVPVGQTSEFKRALADNRVLID..GKLLKWLVVYPAREERTIASFCETMMKKCRAMGITVAQPSTVALP
D.melanogaster_PIWI     ....PAGENADWQRHFRDQRMLTTPSDGLDRWAVIAPQRNSHELRTLLDSLYRAASGMGLRIRSPQEFIIY
D.melanogaster_Aubergine ...FVCDARADWTNEFRTCSMFKN..VHINRWYVITPSRNLRETQEFVQMCIRTASSMKMNICNPIYEEIP
Macpiwi-1               ....VDPERADWDNALKGNGMFQP..VNCKNWIFVYSQRDEQAAADFCQKLSRASQSLGMSFGEPYVLAVA
Macpiwi-2               ....VDPERADWDNGLKGNGMFQP..IDCKNWIFVYFQRDEQAAADFCQKLHRVSQSLGMSFGEPYVLPVM
H.sapiens_PIWIL4        ....QPVSAADWSKDIRTCKILNA..QSLNTWLILCSDRTEYVAESFLNCLRRVAGSMGFNVDYPKIIKVQ
A.californica_piwi1     .......................................................................
H.sapiens_PIWIL3        ....KANSQGDWSREIRELPLLNA..MPLHSWLILYSRSSHREAMSLKGHLQSVTAPMGITMKPAEMIEVD
Smedwi-3                ...VSAGEQAEFKQALAHNKVIGG..IRIENWILISPKSLLTKANGLLQALMSKSPRVGVMFGKPKIVEMN
H.sapiens_PIWIL2        ...IT.SQELNWVKEVTRDPSILT..IPMHFWALFYPKRAMDQARELVNMLEKIAGPIGMRMSPPAWVELK
H.sapiens_PIWIL1        ...DYNPQFADWSKETRGAPLISV..KPLDNWLLIYTRRNYEAANSLIQNLFKVTPAMGMQMRKAIMIEV.
A.californica_piwi2     .......................................................................
```

```
                       130        140       150       160       170       180
Smedwi-1                SCRSNEVEDALTDFIRV....S..KVHMALV....FIPDDKVYAKVKNFT.MSTGLLTQCVTQRNGSNRD.
Smedwi-2                TCGPNDVERCLNDAARS....GSGCAKMALV....FVPDDRVYAKVKSFT.MSTGLLTQCVTTRNGTNRN.
D.japonica_Piwi         .............................LV....FVPDDRVYAKVKSFT.ISTGLLTQCVTTRNGTNRN.
Macpiwi-3               NDSSGQYQQAIKQHFTT.........DLNLVCAVFTSQREERYNMVKRLCCTELVVPSQCLWTKTI....S
D.melanogaster_PIWI     DDRTGTYVRAMDDCVRS.........DPKLILCLVPNDNAERYSSIKKRGYVDRAVPTQVVTLKTT.....
D.melanogaster_Aubergine DDRNGTYSQAIDNAAAN.........DPQIVMVVMRSPNEEKYSCIKKRTCVDRPVPSQVVTLKVIAPRQQ
Macpiwi-1               QDRDQLWVKTIEENIDQ.........GLDLVFCLLPSNKKQRYDSIKRLCYVNKPVPSQCVLTKTI....R
Macpiwi-2               QDRDQFWLKTIQDNINQ.........ELDLVFCLLPNNNAHRYNIIKRLCYVDKPVPSQCVLTKTI....R
H.sapiens_PIWIL4        EN.PAAFVRAIQQYVDP.........DVQLVMCILPSNQKTYYDSIKKYLSSDCPVPSQCVLARTL....N
A.californica_piwi1     .............................MIVTVVPNNKKDRYDAIKKSCCIDNPVPSQVVLQKTL....S
H.sapiens_PIWIL3        GD.ANSYIDTLRKYTRPTLQMGMSCLLVFKVICILPNDDKRRYDSIKRYLCTKCPIPSQCVVKKTL....E
Smedwi-3                NDRTEEYLKELKRNVAP.........GVQLVVTILSAVREDRYNAIKKFCYVDCPVPSQVVLAQTL....K
H.sapiens_PIWIL2        DDRIETYVRTIQSTLGAEG.......KIQMVVCIIMGPRDDLYGAIKKLCCVQSPVPSQVVNVRTI....G
H.sapiens_PIWIL1        DDRTEAYLRVLQQKVTA.........DTQIVVCLLSSNRKDKYDAIKKYLCTDCPTPSQCVVARTL....G
A.californica_piwi2     .............................LVVVINPTSRDDRYSAIKKVCCVEAPVPSQVIVARTI....S
```

```
                        190       200       210       220       230       240       250
Smedwi-1                .DRRRKTVADKSVMQMFSKLGYDPWGINLKMAPTMIVGLDTFHSK.TGKRSVQASVFSISAKF.SQYISFV
Smedwi-2                .DKRRKVVSSKTVMQIFAKFGYDPWTVEIKLRPTMIVGMDTYHNK.SSKKSIQASVFSINSTF.TQYMSFV
D.japonica_Piwi         .DKRRQVVSSKTVMQIFSKFGYDPWTIDIKMRPTMIVGMDTFHNK.GSKKSIHASVFSINSTF.SQYMSFA
Macpiwi-3               DPKKMGSVAVNVAAQINVKLGGALWGIEIPYDRFMMLGIDVYHADKARTTSYTGIVASVDQAC.TRWYSRV
D.melanogaster_PIWI     KNRSLMSIATKIAIQLNCKLGYTPWMIELPLSGLMTIGFDIAKSTRDRKRAYGALIASMDLQQNSTYFSTV
D.melanogaster_Aubergine KPTGLMSIATKVVIQMNAKLMGAPWQVVIPLHGLMTVGFDVCHSPKNKNKSYGAFVATMDQKESFRYFSTV
Macpiwi-1               NPAKVMSVATKVALQISCKLGGVAWAVSIPMKRTMIVGMDTYHDK.RQSVSVQGIVFSLNETF.TQYYSYS
Macpiwi-2               NPAKVMSVATKVAIQISCKLGGVAWALSIPIKRTMIIGMDSYHDK.RQSRSVQGVVFSLNETF.TQYYSYS
H.sapiens_PIWIL4        KQGMMMSIATKIAMQMTCKLGGELWAVEIPLKSLMVVGIDVCKDALSKDVMVVGCVASVNPRI.TRWFSRC
A.californica_piwi1     KQQGLMSVATKIAIQLNCKMGGEVWGAVIPVKGMMIIGLDTYHDSANKNQSVGAMVASLNKEC.TRYYCKT
H.sapiens_PIWIL3        KVQ.ARTIVTKIAQQMNCKMGGALWKVETDVQRTMFVGIDCFHDIVNRQKSIAGFVASTNAEL.TKWYSQC
Smedwi-3                EGPKLNSVAVNIALQINAKLGGELWAVKIPIKKFMVVGLDVWHDTKGRSRSVGAVVGSTNALC.TRWFSKS
H.sapiens_PIWIL2        QPTRLRSVAQKILLQINCKLGGELWGVDIPLKQLMVIGMDVYHDPSRGMRSVVGFVASINLTL.TKWYSRV
H.sapiens_PIWIL1        KQQTVMAIATKIALQMNCKMGGELWRVDIPLKLVMIVGIDCYHDMTAGRRSIAGFVASINEGM.TRWFSRC
A.californica_piwi2     KPEKMRSVVQKIALQINCKLGGELWAVKIPLKSLMVVGIDSFHDNARGKQSVGGFVCSLNATC.TRWFSNV
```

```
                                  260       270       280       290       300       310       320
Smedwi-1                     .NSSKGKNEFHENLGKNFLTALTTFQNKFNTMPLRLIIYRDGVGDSQLAFTKKFETDAVMKMIEKIYENQ.
Smedwi-2                     VNSPKGRQEFHETLGKNFNLALEDFKKRYDILPQRILVFRDGVGDNQLQFTKNFEVDAMKPLIENIYKGNG
D.japonica_Piwi              .NSPKGKQEFHDTLSGNFKAALTEFKRIYKILPVRIMVYRDGVGDSQLQFTKQFEVDAMKPLVENIYKENG
Macpiwi-3                    ATQRH.GQEILDSMRVIVQSQIAKYMQTVGAPPNVIFVFRDGVSDKQIGEVQQQEVKAIYAAIRG.YDPSY
D.melanogaster_PIWI          TECSA.FDVLANTLWPMIAKALRQYQHEHRKLPSRIVFYRDGVSSGSLKQLFEFEVKDIIEKLKTEYARVQ
D.melanogaster_Aubergine     NEHIK.GQELSEQMSVNMACALRSYQEQHRSLPERILFFRDGVGDGQLYQVVNSEVNTLKDRLDEIYKSAG
Macpiwi-1                    PIVKGGKAELHNRLEVGFNLALQKFREKNGDLPTRIILYRDGVGDSMLEEVKNSELLQLKQSLSKIYGERM
Macpiwi-2                    PIVKGGKAELHNRLDAGFTMALQKFREKNGELPARIILYRDGVGDSMLEQVKNSELVQLKQSLSRIYGDRI
H.sapiens_PIWIL4             ILQRT.MTDVADCLKVFMTGALNKWYKYNHDLPARIIVYRAGVGDGQLKTLIEYEVPQLLSSVAE.SSSNT
A.californica_piwi1          EYHDK.KAEIMQSLGVLVTGALRKFHETNQANPTRVIVYRDGVGDGQLDAVFQSEKEQIEQAFRMAGGESF
H.sapiens_PIWIL3             VIQKT.GEELVKELEICLKAALDVWCKNESSMPHSVIVYRDGVGDGQLQALLDHEAKKMSTYLKT.ISPN.
Smedwi-3                     HLQEQ.DKEIMYVLQSCMLSLLKAYFEENNFLPETIFMYRDGVSDGQLGYVQKTEIEQFFKVFES.FSADY
H.sapiens_PIWIL2             VFQMP.HQEIVDSLKLCLVGSLKKFYEVNHCLPEKIVVYRDGVSDGQLKTVANYEIPQLQKCFEA.F.ENY
H.sapiens_PIWIL1             IFQDR.GQELVDGLKVCLQAALRAWNSCNEYMPSRIIVYRDGVGDGQLKTLVNYEVPQFLDCLKS.IGRGY
A.californica_piwi2          CIQRQ.GEELVHGLQLCLTKGLRKFHADNHFLPEKIVIFRDGVGDGQLNTLADHEVKQLHRCFLS.FGEGY
```

```
                                    330       340                350       360       370       380
Smedwi-1                     .TL.PQIIYVVVKKRISVKFFKDG.......ANPNPGTVVDEKIVKPNFYEFYLVSQKTTKGTASPTNYNV
Smedwi-2                     FPV.PQIIYVIVKKRVGTKLFNRG.......NNPNPGTVVDKEIVKPNFYEFFLVSQRTTKGTATPTNYNV
D.japonica_Piwi              CQV.PQIIYVIVKKRIGTKLFNRG.......DNANPGTIVDKEIVKPNFFEFFLVSQRVTRGTASPTNYNV
Macpiwi-3                    ..R.PKLVFMVVQKRINQRLFLLQG...NNFSNPPPGTVLDHSITRPEMMDFFLVPQHVTQGTVTPTHYIV
D.melanogaster_PIWI          .LSPPQLAYIVVTRSMNTRFFLNG.......QNPPPGTIVDDVITLPERYDFYLVSQQVRQGTVSPTSYNV
D.melanogaster_Aubergine     KQEGCRMTFIIVSKRINSRYFTGH.......RNPVPGTVVDDVITLPERYDFFLVSQAVRIGTVSPTSYNV
Macpiwi-1                    ..SSLGFKAIVVKKLVSSRMFRKQG...SQLRNPAPGTVLDDVITMPNFVDFFLVSQYVNQGTVTPTHYNM
Macpiwi-2                    ..SSLGFKVIIVKKLVSSRLFRRGG...NQLRNPAPGTVLDDVITMPNYVDFFLVSQYVNQGTVTPTHYNI
H.sapiens_PIWIL4             ..S.SRLSVIVVRKKCMPRFFTEMN...RTVQNPPLGTVVDSEATRNEWYDFYLISQVACRGTVSPTYYNV
A.californica_piwi1          ..K.PALTMVIVKKRINTRIFKRAE....KVMNPQPGSVVDTFITKQEWYDFFLVSQSVRQGTVSPTSYNV
H.sapiens_PIWIL3             ..N.FTLAFIVVKKRINTRFFLKHG...SNFQNPPPGTVIDVELTRNEWYDFFIVSQSVQDGTVTPTHYNV
Smedwi-3                     ..K.PNMVYNVVQKRINTRLYVSDPKNKGQINNPNPGTIVDHTVTRANLYDFFLVSQSVRQGTVTPTHYVV
H.sapiens_PIWIL2             ..Q.PKMVVFVVQKKISTNLYLAAP...QNFVTPTPGTVVDHTITSCEWVDFYLLAHHVRQGCGIPTHYVC
H.sapiens_PIWIL1             ..N.PRLTVIVVKKRVNTRFFAQSG...GRLQNPLPGTVIDVEVTRPEWYDFFIVSQAVRSGSVSPTHYNV
A.californica_piwi2          ..Q.PLCSTVVVQKRINTRIFSKGD...GGVDNPGPGTVVDHTVTRRDRYDFYIVSQHVRQGTVSPTHYIV
```

```
                               390       400       410       420       430       440
Smedwi-1                     LMDTKFTNKKTNEVSVMSPSVLQQITYSLTHLYFNWMGTIRVPVPTHYAHRLAELVGK...
Smedwi-2                     LEDTRLLTKK.GTMDPMAPNELQKITYALTHLYFNWMGTIRVPVPVHYAHRLAELVGK...
D.japonica_Piwi              LEDTRYQNKA.GIIEAITPNELQRITYTLTHLYFNWMGTIRVPVPVHYAHKLAELIGK...
Macpiwi-3                    VEC..A........GDIPVDEIQQLTFRSCHLYYNWAGSIRVPAPCQYAHKLVYMVGQ...
D.melanogaster_PIWI          LYS..S........MGLSPEKMQKLTYKMCHLYYNWSGTTRVPAVCQYAKKLATLVGT...
D.melanogaster_Aubergine     ISD..N........MGLNADKLQMLSYKMTHMYYNYSGTIRVPAVCHYAHKLAFLVAE...
Macpiwi-1                    IEDVND........ANIKPDQVQQLTYKLTHLYFNWPGTIRVPAPCHYAHRLAYLVGQ...
Macpiwi-2                    IEDVND........ANIRPDQVQQLTYKLCHLYFNWPGTIRVPAPCQYAHRLAYLVGQ...
H.sapiens_PIWIL4             IYD..D........NGLKPDHMQRLTFKLCHLYYNWPGIVSVPAPCQYAHKLTFLVAQ...
A.californica_piwi1          IFD..E........SGLKPDHLQQLTYKMTHLYFNWQGTIRVPAPCQYAHKLAFLQGQSLH
H.sapiens_PIWIL3             IYD..T........IGLSPDTVQRLTYCLCHMYYNLPGIIRVPAPCHYAHKLAYLVGQ...
Smedwi-3                     LCD..N........SKYTPHQVQLMAYKTCHIYYNWPGTVRVPAPCMYAHKLAYMVGQ...
H.sapiens_PIWIL2             VLN..T........ANLSPDHMQRLTFKLCHMYWNWPGTIRVPAPCKYAHKLAFLSGH...
H.sapiens_PIWIL1             IYD..N........SGLKPDHIQRLTYKLCHIYYNWPGVIRVPAPCQYAHKLAFLVGQ...
A.californica_piwi2          VDD..G........LQLKPDHMQALTYKMTHLYYNWPGTVRVPAPCQYAHKLAYLVGENIH
```
