# Supplementary figures and images for: Dual functions of Macpiwi1 in transposon silencing and stem cell maintenance in the flatworm Macrostomum lignano
Source: RNA. 2015 Nov;21(11):1885–97. doi: 10.1261/rna.052456.115 (PMC4604429; doi:10.1261/rna.052456.115)

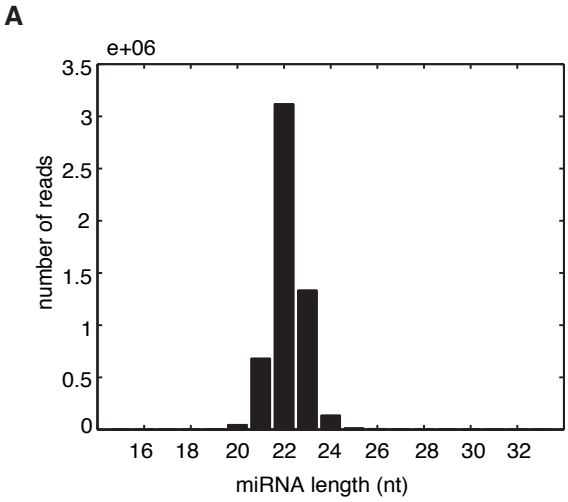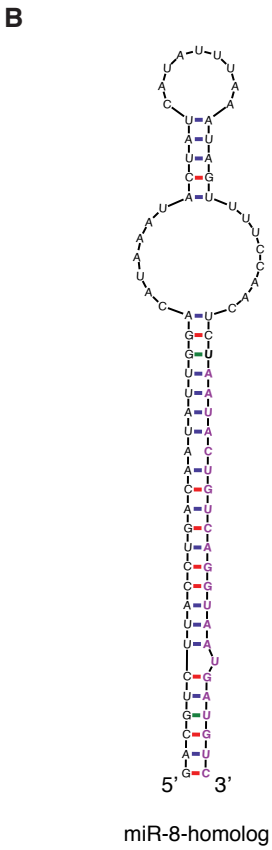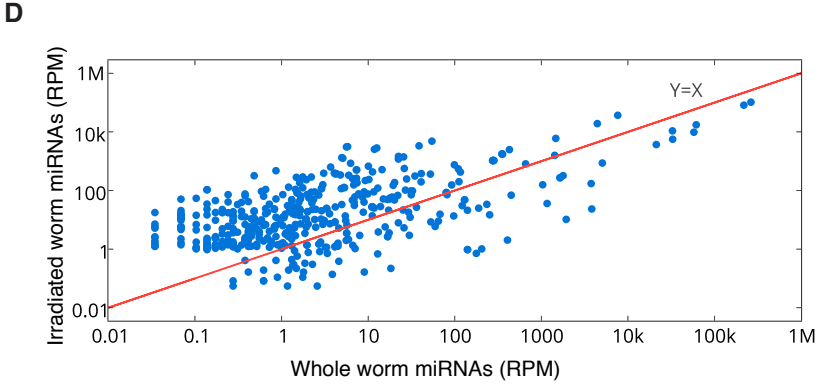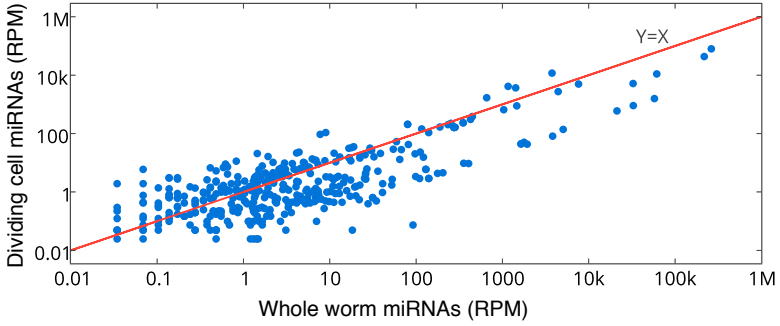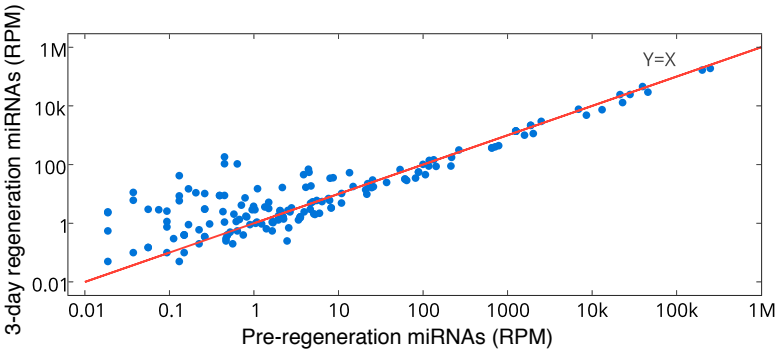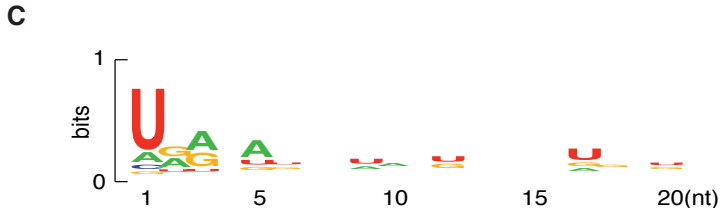

Supplement: Supplemental Material [file supp_052456.115_Sup_Fig2.pdf]

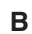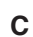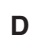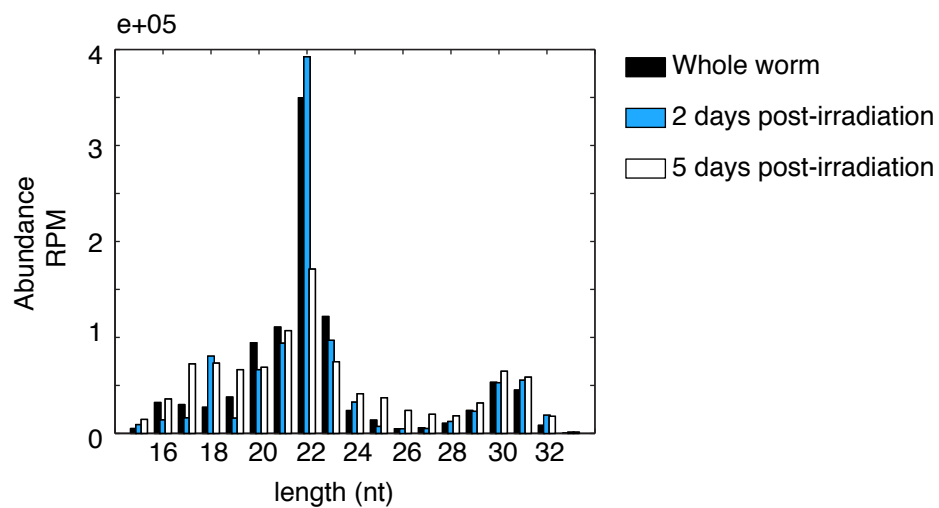

Supplement: Supplemental Material [file supp_052456.115_Sup_Fig3.pdf]

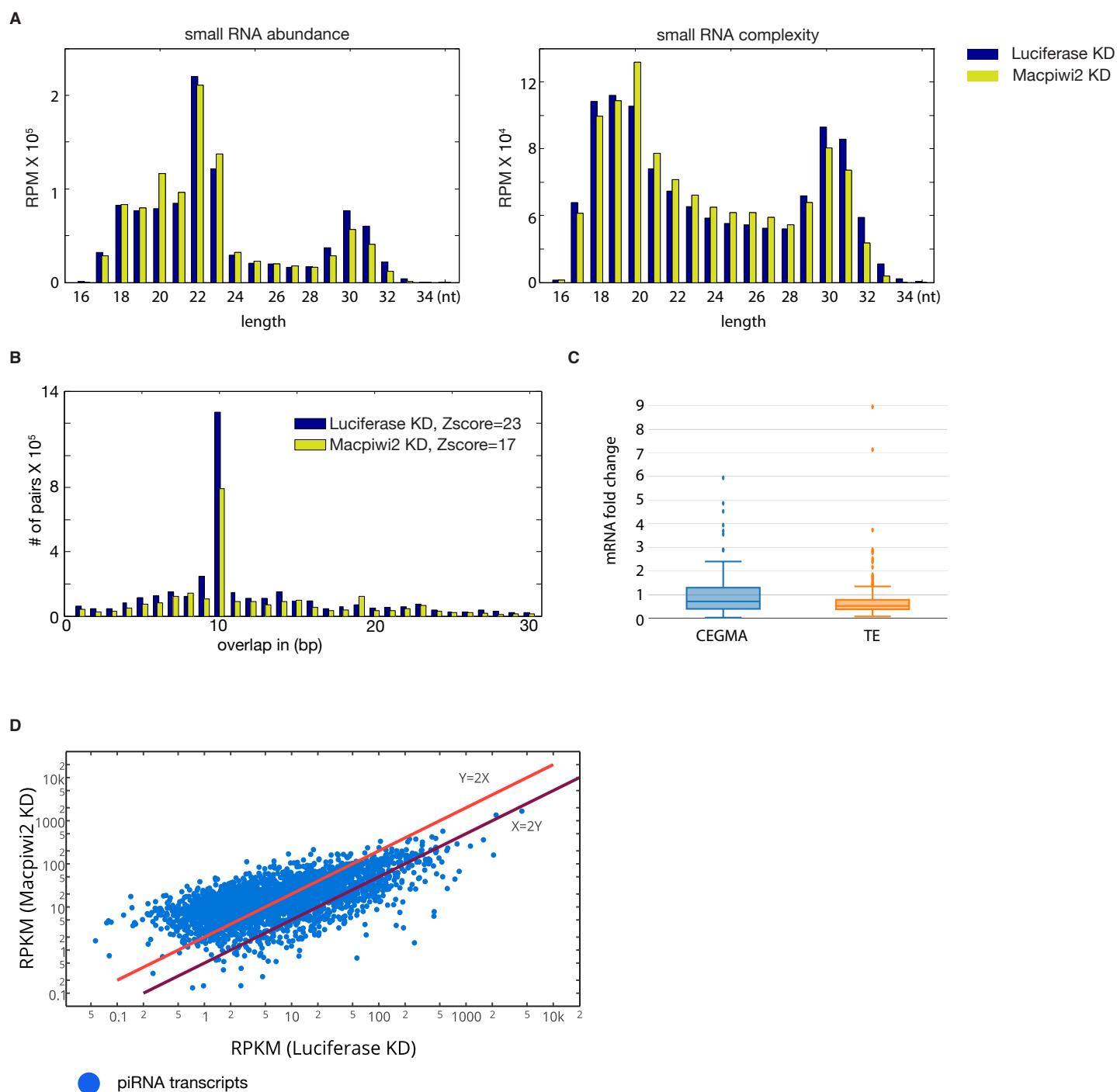

Supplement: Supplemental Material [file supp_052456.115_Sup_Fig4.pdf]

**A**

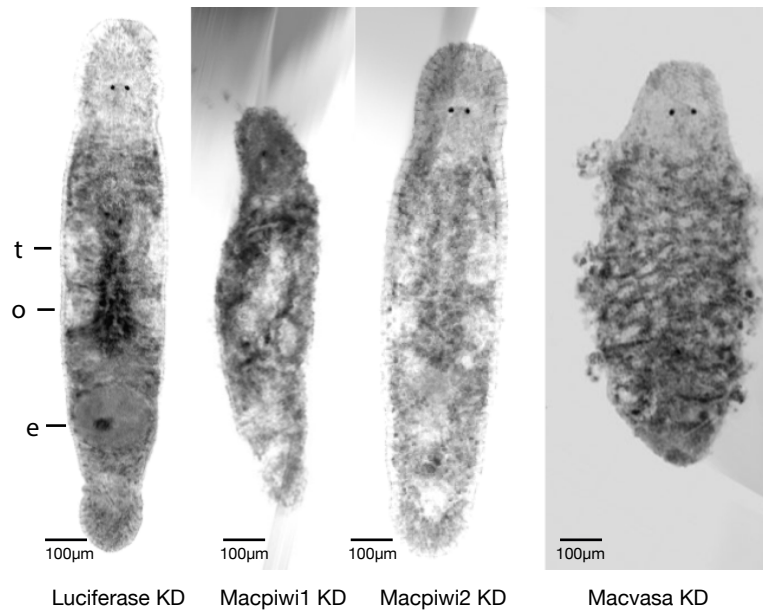

B

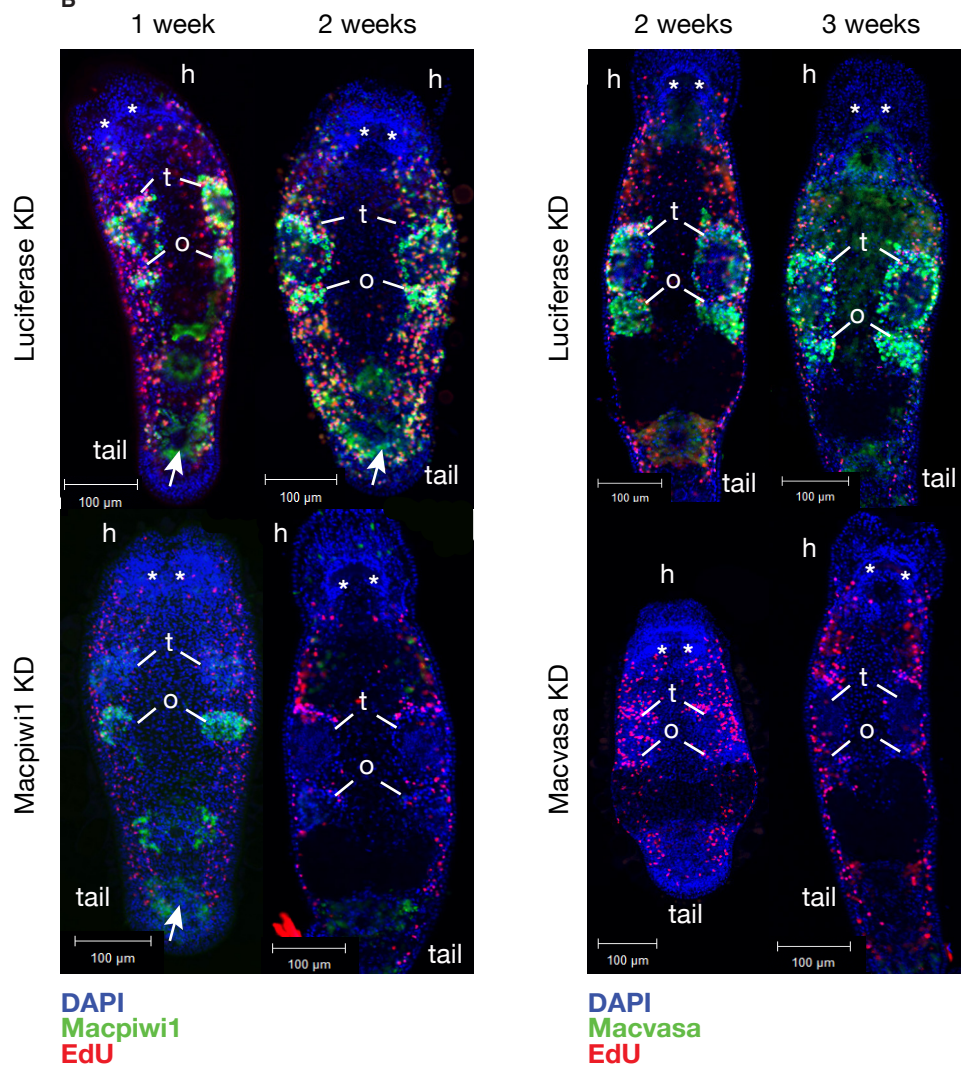

Supplement: Supplemental Material [file supp_052456.115_Sup_Fig5.pdf]
