# Supplementary material for: Dual functions of Macpiwi1 in transposon silencing and stem cell maintenance in the flatworm Macrostomum lignano
Source: RNA. 2015 Nov;21(11):1885–97. doi: 10.1261/rna.052456.115 (PMC4604429; doi:10.1261/rna.052456.115)
Supplement: Supplemental Material [file supp_052456.115_SuppLegendsTables.docx]

**SUPPLEMENTARY FIGURES**

**Supplementary Figure 1.** Sequence alignment of PIWI domains from PIWI proteins in various metazoan species. Identical (white letters on a red background) or similar (red letters on a white background) residues are labeled.

**Supplementary Figure 2**. Characteristics of *M. lignano* miRNAs. (A) Length distribution of small RNAs that match to miRBase mature miRNAs. (B) Stem-loop structure of the miR-8 homologue precursor from the genome draft. The mature miRNA sequence is highlighted in purple. (C) Sequence bias of all small RNAs mapped to miRBase mature miRNAs. (D) Abundance of miRNA in whole worms, irradiated worms, sorted dividing cells, and regenerating worms (0 day or 3 days post anterior amputation). Each dot represents a miRNA.

**Supplementary Figure 3**. piRNAs in sorted dividing cells and irradiated worms. (A) 2N and 4N cell populations from whole worm cells labeled with propidium iodide. Blue quadrangle: total cells; red quadrangle: 4N cells. (B) Length distribution of small RNAs mapping to the genome from 2N and 4N cells. (C) Immunostaining for Macpiwi1 and EdU labeling in untreated and irradiated worms. t: testis; o: ovary; e: egg. Stars denote eyes. Arrows point to nonspecific staining by the secondary antibody. (D) Length distribution of small RNAs from untreated and irradiated worms 2 days and 5 days post-irradiation.

**Supplementary Figure 4**. Impact of *Macpiwi2* knockdown on piRNA production and transposon silencing. (A) Length distribution of sequence abundance and complexity of small RNAs mapped to genome from luciferase and *Macpiwi2* KD. (B) Distribution of 5’ overlap between piRNAs from sense and antisense strands in *luciferase* and *Macpiwi2* KD. (C) mRNA fold change of CEGMA core gene set and transposon consensus sequences in *Macpiwi2* KD, compared with *luciferase* KD. (D) Read counts of piRNAs mapped to individual transposon-related transcripts are plotted for *luciferase* KD and *Macpiwi2* KD worms. Each dot represents a transposon consensus sequence. Axes are presented on log10 scale.

**Supplementary Figure 5**. Morphology of adult worms and knockdown efficiency following RNAi. (A) Differential interference contrast (DIC) images of adult worms treated with *luciferase* (4 weeks), *Macpiwi1* (3 weeks), *Macpiwi2* (5 weeks) and *Macvasa* (4 weeks) RNAi. t: testis; o: ovary: e: egg. (B) Macpiwi1 and Macvasa immunostaining following *Macpiwi1* and *Macvasa* RNAi respectively. *Luciferase* RNAi serves as a negative control. t: testis; o: ovary: e: egg. Stars denote eyes. Arrows point to nonspecific staining from the secondary antibody.

**SUPPLEMENTARY TABLE LEGENDS**

**Supplementary Table 1.** List of piRNA and miRNA pathway-related genes identified in *M. lignano.* Homologues were identified based on the mRNA sequences from the *de novo* transcriptome assembly.

**Supplementary Table 2.** List of conserved miRNAs and their expression levels in different conditions. RPM: reads per million total reads.

**Supplementary Table 3.** Top 10 piRNA-producing transcripts ranked by sequence complexity.

**Supplementary Table 4.** List of piRNA clusters across the *M. lignano* genome assembly.

**Supplementary Table 5.** List of piRNA-producing transcripts from the *de novo* transcriptome and their piRNA levels. PPM: parts per million total reads; RPKM: reads per kilo base pairs per million total reads.

**Supplementary Table 6.** Mapping statistics of small RNAs from whole worm and Macpiwi1 immunoprecipitation.

**Supplementary table 1. Homologues of piRNA and microRNA pathway components in *M. lignano***

| piRNA pathway | miRNA pathway |
| --- | --- |
| PIWI proteins | **AGO proteins** |
| Macpiwi1 | Macago1 |
| Macpiwi2 | Macago2 |
| Macpiwi3 |  |
| Other factors | **Other factors** |
| Tdrkh | DROSHA |
| Tdrd6 | DICER1 |
| Tdrd9 | DGCR8 |
| Tdrd12 | TARBP2 |
| Macvasa | Exportin-5 |
| MOV10L1 |  |
| Maelstrom |  |
| HEN1 |  |
| Hsp90 |  |
| Fkbp6 |  |
| PRMT5 |  |

*Homologues were identified based on the mRNA sequences from the *de novo* transcriptome assembly. Accession numbers: AM942740 (Macpiwi1), KR132203 (Macpiwi2), KR132204 (Macpiwi3), KR132205 (Tdrkh), KR132206 (Tdrd6), KR132207 (Tdrd9), KR132208 (Tdrd12), AM411989 (Macvasa), AM411990 (Macvasa), KR132209 (MOV10L1), KR132210 (Maelstrom), KR132211 (HEN1), KR132212 (Hsp90), KR132213 (Fkbp6), KR132214 (PRMT5), KR132217 (Macago1), KR132215 (Macago2), KR132216 (Macago2), KR132218 (Drosha), KR132219 (Dicer), KR132220 (DGCR8), KR132221 (TARBP2), KR132222 (Exportin-5).

**Supplementary Table 2.** **List of conserved miRNAs and their expression levels in different conditions**

|  | RPM | RPM | RPM | RPM | RPM |
| --- | --- | --- | --- | --- | --- |
| miRNA ID | Whole worm | Dividing cell | Irradiated worm | Regeneration day 0 | Regeneration day 3 |
| bantam | 13.0868 | 15.8414932 | 3.8352324 | 0.4480238 | 183.042504 |
| bantam-3p | 3.20282 | 3.972711 | 1.1505696 | 0.1306737 | 41.895286 |
| let-7 | 22.350818 | 14.508987 | 1184.704762 | 4.405562 | 69.5262 |
| let-7-5p | 5.9579228 | 4.293472 | 331.6657836 | 1.101391 | 15.062337 |
| let-7a | 25.6569528 | 18.728412 | 1401.5055 | 4.480234 | 54.613407 |
| let-7a-5p | 22.385261 | 16.532328 | 1458.40355 | 3.864202 | 45.38648 |
| let-7b | 6.267866 | 13.571356 | 850.1628 | 7.840421 | 6.034905 |
| let-7b-5p | 3.684952 | 9.376576 | 630.2393 | 5.954986 | 2.194511 |
| let-7c | 5.613533 | 6.958409 | 3069.883664 | 0.616033 | 0 |
| let-7c-5p | 5.785728 | 7.10646 | 3148.944 | 0.672036 | 0 |
| let-7d | 1.2053587 | 0.0740256 | 129.9599326 | 0 | 0 |
| let-7d-5p | 1.6186237 | 0 | 183.351999 | 0 | 0 |
| let-7e | 0.137755 | 0.6415552 | 34.4349464 | 0.056003 | 0 |
| let-7e-5p | 0 | 0.5922048 | 28.3807655 | 0 | 0 |
| let-7f | 5.096948 | 9.82073 | 1264.8094 | 0.616033 | 0 |
| let-7f-5p | 4.959192 | 10.363584 | 1322.886789 | 0.672036 | 0 |
| let-7g | 7.369908 | 2.3194716 | 502.361167 | 0 | 0 |
| let-7g-5p | 10.056136 | 3.2571304 | 675.2481945 | 0 | 0 |
| let-7i | 2.892863 | 1.751936 | 113.824456 | 0 | 0 |
| let-7i-5p | 2.892865 | 1.8012856 | 120.28958 | 0 | 0 |
| let-7j-5p | 1.51531 | 1.11038 | 82.5945891 | 0.261347 | 3.04239 |
| miR-1 | 7624.9609 | 4966.0296 | 36891.62 | 39574.169 | 45690.752 |
| miR-10 | 0.4821438 | 2.3688192 | 7.2321581 | 0.896048 | 0.897756 |
| miR-100 | 0.86097 | 3.3311512 | 473.3236048 | 0.9333825 | 0 |
| miR-100-5p | 0.4821432 | 2.196092 | 319.1469636 | 0.5226942 | 0 |
| miR-100a-5p | 0.1033164 | 0.419478 | 54.0495 | 0.1120059 | 0 |
| miR-100b | 0.0344388 | 0.123376 | 17.6969 | 0.0373353 | 0 |
| miR-101 | 0 | 1.0116832 | 5.4241195 | 0 | 0 |
| miR-101-3p | 0 | 0.3948032 | 1.753251 | 0 | 0 |
| miR-101a | 0 | 0.4441536 | 2.958612 | 0 | 0 |
| miR-101a-3p | 0 | 0.2714272 | 1.2053595 | 0 | 0 |
| miR-101b-3p | 0.0688777 | 0.1480512 | 5.341941 | 0 | 0 |
| miR-103 | 11.2270625 | 21.269948 | 430.8886 | 4.256228 | 0 |
| miR-103-3p | 6.6122577 | 12.411582 | 252.9885 | 2.464132 | 0 |
| miR-103a | 0.654338 | 1.15973 | 24.0524 | 0.224012 | 0 |
| miR-103a-3p | 1.963014 | 3.47919 | 72.1572 | 0.672036 | 0 |
| miR-103b | 1.308676 | 2.31946 | 48.1048 | 0.448024 | 0 |
| miR-103b-3p | 1.308676 | 2.31946 | 48.1048 | 0.448024 | 0 |
| miR-10-3p | 0.86097 | 0 | 0.410918 | 0 | 1.24688 |
| miR-10-5p | 0.4132665 | 1.4558368 | 4.081786 | 0.672036 | 1.346634 |
| miR-106a | 1.20536 | 0.0246752 | 2.68467 | 0 | 0 |
| miR-106b | 2.479596 | 0.5922048 | 5.588484 | 0 | 0 |
| miR-106b-3p | 0.2066328 | 0.7155808 | 1.4245182 | 0 | 0 |
| miR-106b-5p | 1.274237 | 0.2961024 | 2.821637 | 0 | 0 |
| miR-107 | 1.9285753 | 16.384342 | 233.0721 | 4.704252 | 0 |
| miR-107-3p | 0.5854604 | 6.859712 | 93.5795 | 2.016108 | 0 |
| miR-107a | 0.7232157 | 1.924662 | 34.0788 | 0.448024 | 0 |
| miR-107a-3p | 0.0688777 | 0.764932 | 10.7934 | 0.224012 | 0 |
| miR-107b | 0.0688777 | 1.924667 | 12.026154 | 0.448024 | 0 |
| miR-10a | 1.4808666 | 4.071408 | 19.2309866 | 2.464132 | 0.249376 |
| miR-10a-5p | 1.5153056 | 5.724646 | 25.915255 | 3.36018 | 0 |
| miR-10b | 2.410716 | 3.2077764 | 89.19669 | 0 | 0 |
| miR-10b-5p | 2.066328 | 2.689597 | 74.37623 | 0 | 0 |
| miR-10c-5p | 0.172194 | 0.296103 | 9.67027 | 0 | 0 |
| miR-11 | 1.377558 | 0.789607 | 0.3013395 | 0.0373354 | 6.084784 |
| miR-11-3p | 0.89541 | 0.6909068 | 0.109578 | 0.0186677 | 2.294264 |
| miR-1175-3p | 3756.685878 | 11758.22678 | 172.0652 | 265.6407 | 313.9148753 |
| miR-12 | 26.07027 | 4.4908954 | 0 | 0 | 3.74064 |
| miR-122 | 0 | 70.69446 | 0 | 0 | 0 |
| miR-122-5p | 0 | 56.7283 | 0 | 0 | 0 |
| miR-122a-5p | 0 | 4.73764 | 0 | 0 | 0 |
| miR-12-3p | 1.61863 | 0.296103 | 0 | 0 | 0.249376 |
| miR-124 | 57696.64 | 1577.9554 | 9742.3672 | 22955.4436 | 13009.183 |
| miR-124-3p | 32827.72943 | 907.5543504 | 5603.87778 | 13125.64634 | 7424.49013 |
| miR-124-4-3p | 130.317 | 5.94673 | 47.9404 | 61.1179 | 32.1696 |
| miR-1249 | 0 | 0 | 1.205358 | 0 | 0 |
| miR-124a | 21249.683 | 597.23853 | 3692.8624 | 8566.0299 | 4832.5216 |
| miR-124a-1-3p | 1643.21 | 44.2673 | 272.548 | 650.848 | 370.025 |
| miR-124a-3p | 5060.707502 | 138.8226556 | 866.022758 | 2014.949986 | 1143.542056 |
| miR-124b | 1782.003992 | 50.3127804 | 321.2555285 | 713.646339 | 403.0426773 |
| miR-124b-3p | 129.421072 | 5.9714052 | 47.748678 | 61.0805853 | 32.069826 |
| miR-124c-3p | 1643.21 | 44.2673 | 272.548 | 650.848 | 370.025 |
| miR-125 | 6.8188937 | 0.6909056 | 155.107891 | 0 | 0.6982542 |
| miR-125-5p | 4.8903177 | 0.493504 | 115.76933 | 0 | 0.5486283 |
| miR-125a | 1.1709211 | 0.0987008 | 30.2983611 | 0 | 0.0997506 |
| miR-125a-5p | 2.0663313 | 0.1480512 | 55.77527 | 0 | 0.1496259 |
| miR-125b | 10.1939017 | 1.0363584 | 237.20933 | 0 | 1.0972566 |
| miR-125b-3p | 0.482144 | 0.0493504 | 11.0673945 | 0 | 0.0498753 |
| miR-125b-5p | 5.3380228 | 0.5428544 | 124.48079 | 0 | 0.5985036 |
| miR-125c | 0.482144 | 0.0493504 | 11.04 | 0 | 0.0498753 |
| miR-12-5p | 5.7513 | 0.9129842 | 0 | 0 | 0.748128 |
| miR-126 | 0.3443885 | 0.937658 | 39.092028 | 0 | 0 |
| miR-126-3p | 0.6198993 | 1.2337604 | 63.719726 | 0 | 0 |
| miR-126-5p | 0.0688777 | 5.9467282 | 50.570287 | 0 | 0 |
| miR-126a | 0 | 0 | 2.16417 | 0 | 0 |
| miR-126a-3p | 0.1377554 | 0.1974016 | 14.65609 | 0 | 0 |
| miR-126a-5p | 0 | 1.332462 | 10.19076 | 0 | 0 |
| miR-126b | 0 | 0.444154 | 3.39692 | 0 | 0 |
| miR-126b-3p | 0 | 0.444154 | 3.39692 | 0 | 0 |
| miR-126b-5p | 0 | 0.444154 | 3.506498 | 0 | 0 |
| miR-127 | 0.4132656 | 6.711652 | 3.451707 | 0 | 0 |
| miR-127-3p | 0.172194 | 3.084399 | 1.4245141 | 0 | 0 |
| miR-128 | 2.4107126 | 0.4194784 | 10.24556 | 0 | 0 |
| miR-128-3p | 1.65306 | 0.2961024 | 7.23216 | 0 | 0 |
| miR-128a-3p | 0.27551 | 0.0493504 | 1.20536 | 0 | 0 |
| miR-128b-3p | 0.27551 | 0.0493504 | 1.20536 | 0 | 0 |
| miR-129 | 0 | 0 | 4.4653136 | 0 | 0 |
| miR-129-2-3p | 0 | 0 | 2.9312156 | 0 | 0 |
| miR-129-3p | 0 | 0 | 3.56129 | 0 | 0 |
| miR-129-5p | 0 | 0 | 5.698069 | 0 | 0 |
| miR-129a | 0 | 0 | 1.150571 | 0 | 0 |
| miR-129a-3p | 0 | 0 | 1.424516 | 0 | 0 |
| miR-129a-5p | 0 | 0 | 1.753252 | 0 | 0 |
| miR-129b | 0 | 0 | 1.588884 | 0 | 0 |
| miR-129b-3p | 0 | 0 | 1.6162776 | 0 | 0 |
| miR-130 | 0.275511 | 0.370128 | 9.06759 | 0.0186677 | 0.0498753 |
| miR-1306 | 0 | 0 | 1.643674 | 0 | 0 |
| miR-1306-5p | 0 | 0 | 4.38313 | 0 | 0 |
| miR-130a | 2.6862321 | 4.367511 | 69.938232 | 0.2240124 | 0.5985036 |
| miR-130a-3p | 1.5841881 | 2.936349 | 40.023408 | 0.1493416 | 0.3990024 |
| miR-130b | 0.2755104 | 0.1974016 | 9.3141196 | 0 | 0 |
| miR-130b-3p | 0.8265325 | 1.2584352 | 23.641462 | 0.0560031 | 0.1496259 |
| miR-130c-3p | 0.826533 | 1.110384 | 22.68267 | 0.0560031 | 0.1496259 |
| miR-133 | 448.3933706 | 383.5508772 | 69.1164326 | 213.744886 | 89.2766706 |
| miR-133-3p | 140.062756 | 107.879892 | 21.1212072 | 64.048772 | 28.8279012 |
| miR-133-5p | 16.7717 | 14.7064 | 2.65727 | 8.19511 | 3.34164 |
| miR-133a | 234.73504 | 201.8923616 | 33.667909 | 106.62977 | 45.23685 |
| miR-133a-3p | 188.8971 | 167.8654 | 24.98385 | 81.55908 | 34.71318 |
| miR-133b | 97.324193 | 20.233626 | 13.5329491 | 37.073926 | 24.6384306 |
| miR-133b-3p | 64.74504 | 13.02848 | 8.98544 | 24.64128 | 16.40899 |
| miR-133c | 59.3037 | 53.0024 | 5.91724 | 21.63584 | 9.77556 |
| miR-133c-3p | 29.8929 | 26.5012 | 2.95862 | 10.84592 | 4.88778 |
| miR-133d | 0.413266 | 1.381812 | 0.1643672 | 0.224012 | 0.1995012 |
| miR-133d-3p | 16.7717 | 14.7064 | 2.65727 | 8.19511 | 3.34164 |
| miR-13-3p | 0.619899 | 0 | 0.0821836 | 0.0746707 | 2.89277 |
| miR-138 | 0.2410716 | 0 | 17.422916 | 0 | 0 |
| miR-138-5p | 0.2755104 | 0 | 18.847432 | 0 | 0 |
| miR-138a | 0.1033164 | 0 | 6.90342 | 0 | 0 |
| miR-138b | 0.0344388 | 0 | 2.520296 | 0 | 0 |
| miR-13a | 1.96301 | 3.01038 | 1.36973 | 2.20278 | 1.64588 |
| miR-13a-3p | 1.96301 | 3.01038 | 1.36973 | 2.20278 | 1.64588 |
| miR-13b | 2.272963 | 0 | 0.3013395 | 0.2053447 | 10.972566 |
| miR-13b-3p | 0.619899 | 0 | 0.0821835 | 0.0560031 | 2.992518 |
| miR-1-3p | 4427.350839 | 2732.578775 | 19201.51205 | 21413.83769 | 24277.04575 |
| miR-14 | 3.857158 | 6.045423 | 0.1369725 | 0 | 3.840399 |
| miR-140 | 0.5510215 | 0.1480512 | 3.396922 | 0 | 0 |
| miR-140-3p | 1.0331639 | 0.9129824 | 12.0535956 | 0 | 0 |
| miR-140-5p | 1.3086763 | 0 | 0.438312 | 0 | 0 |
| miR-141 | 0 | 0.5922048 | 1.123177 | 0.1493416 | 0.3990024 |
| miR-142 | 0.8954096 | 0.2714272 | 1.3971229 | 0 | 0 |
| miR-142-3p | 2.0318901 | 0.246752 | 0.767047 | 0 | 0 |
| miR-142-5p | 0.206633 | 0.4194784 | 1.1779655 | 0 | 0 |
| miR-143 | 3.0650554 | 6.045422 | 389.1393 | 0.3173509 | 0 |
| miR-143-3p | 2.204084 | 4.564911 | 288.3273945 | 0.2240124 | 0 |
| miR-14-3p | 1.170922 | 1.529862 | 0.054789 | 0 | 0.997506 |
| miR-144 | 1.446438 | 0 | 0 | 0 | 0 |
| miR-144-3p | 1.136487 | 0 | 0 | 0 | 0 |
| miR-145 | 1.03317 | 0.493504 | 12.163147 | 0 | 0 |
| miR-145-3p | 0 | 0 | 3.068188 | 0 | 0 |
| miR-145-5p | 1.0676084 | 0.4441536 | 11.889214 | 0 | 0 |
| miR-145a-5p | 0.103317 | 0.0493504 | 1.15057 | 0 | 0 |
| miR-146a | 0 | 0.246752 | 1.369728 | 0 | 0 |
| miR-146a-5p | 0 | 0.3948032 | 1.972408 | 0 | 0 |
| miR-146b-5p | 0 | 0.3948032 | 1.780644 | 0 | 0 |
| miR-148-3p | 0 | 6.51426 | 0 | 0 | 0 |
| miR-148a | 0 | 15.570068 | 0 | 0 | 0 |
| miR-148a-3p | 0 | 17.963565 | 0 | 0 | 0 |
| miR-15 | 3.0995 | 0.0493504 | 9.47851 | 0 | 0.0498753 |
| miR-151 | 0.654338 | 0.0987008 | 16.107973 | 0 | 0 |
| miR-151-5p | 5.234704 | 0.3948032 | 127.768 | 0 | 0 |
| miR-151a-5p | 1.308676 | 0.0987008 | 31.942 | 0 | 0 |
| miR-152 | 0 | 0.4441536 | 1.0683872 | 0 | 0 |
| miR-155-5p | 0 | 0.123376 | 1.040993 | 0 | 0 |
| miR156b | 0 | 0 | 1.479306 | 0.2240118 | 0 |
| miR156c | 0 | 0 | 1.972408 | 0.2986824 | 0 |
| miR156e | 0.0344388 | 0 | 1.232755 | 0.1866765 | 0 |
| miR156f | 0 | 0 | 1.479306 | 0.2240118 | 0 |
| miR156g | 0 | 0 | 1.232755 | 0.1866765 | 0 |
| miR156h | 0 | 0 | 1.232755 | 0.1866765 | 0 |
| miR156i | 0 | 0 | 1.479306 | 0.2240118 | 0 |
| miR159 | 0.0344388 | 1.924667 | 1.890221 | 0.1680093 | 0 |
| miR159a | 0.172194 | 2.7636232 | 2.657269 | 0.2613478 | 0 |
| miR159b | 0.1377552 | 1.2831098 | 1.123177 | 0.1493415 | 0 |
| miR-15a | 19.45789 | 1.480512 | 149.7112745 | 0 | 0 |
| miR-15a-5p | 11.6058588 | 0.8883072 | 89.7993945 | 0 | 0 |
| miR-15b | 31.4082664 | 0.6415552 | 97.606742 | 0 | 0.6483789 |
| miR-15b-3p | 0.2066328 | 0 | 1.80804 | 0 | 0 |
| miR-15b-5p | 21.9720108 | 0.5428544 | 68.048033 | 0 | 0.5486283 |
| miR-15c | 0.1377554 | 0.0987008 | 1.095782 | 0 | 0.0997506 |
| miR-15c-5p | 0.2066331 | 0.1480512 | 1.643673 | 0 | 0.1496259 |
| miR-1-5p | 276.096 | 163.078 | 1052.06 | 1253.27 | 1383.74 |
| miR-16 | 99.011631 | 3.3805006 | 753.158 | 0.896048 | 0 |
| miR-16-5p | 52.312598 | 1.7272628 | 361.63539 | 0.448024 | 0 |
| miR168 | 0 | 0.7649312 | 1.5614905 | 0.1493416 | 0.3990024 |
| miR168a | 0 | 0.6662304 | 1.1779665 | 0.1120062 | 0.2992518 |
| miR-16a | 1.377552 | 0 | 16.43672 | 0.224012 | 0 |
| miR-16a-5p | 9.883948 | 0.4688286 | 76.3486 | 0.280015 | 0 |
| miR-16b | 2.341843 | 0.4688284 | 49.6115 | 0.336018 | 0 |
| miR-16b-5p | 1.653064 | 0.0987008 | 21.01161 | 0.280015 | 0 |
| miR-16c-5p | 8.60971 | 0.271427 | 58.7613 | 0.056003 | 0 |
| miR-17 | 1.411994 | 0.0246752 | 5.36933 | 0 | 0 |
| miR-17-3p | 0.0344388 | 0 | 1.58888 | 0 | 0 |
| miR-17-5p | 31.442677 | 0.6415552 | 71.14375 | 0 | 0 |
| miR-17a-5p | 1.308677 | 0.0246752 | 4.027 | 0 | 0 |
| miR-18 | 11.1237348 | 0 | 27.750663 | 0 | 0 |
| miR-181a | 0 | 3.035052 | 34.024053 | 0.1680093 | 0.8977554 |
| miR-181a-3p | 0 | 0 | 1.917622 | 0 | 0 |
| miR-181a-5p | 0 | 8.438923 | 110.482286 | 0.4293571 | 2.493765 |
| miR-181b | 0 | 1.1350592 | 22.490905 | 0 | 0 |
| miR-181b-5p | 0 | 0.9376576 | 21.477306 | 0 | 0 |
| miR-181c | 0.5510216 | 0.1974016 | 3.314741 | 0 | 0 |
| miR-181c-5p | 0.447705 | 0.2220768 | 3.095586 | 0 | 0 |
| miR-181d | 0 | 0 | 1.232757 | 0 | 0 |
| miR-181e | 0 | 0.0740256 | 1.9998 | 0 | 0 |
| miR-183 | 0 | 2.5168704 | 2.1915601 | 0 | 0 |
| miR-183-5p | 0 | 1.480512 | 1.150569 | 0 | 0 |
| miR-1839 | 0 | 0 | 2.05459 | 0 | 0 |
| miR-1839-5p | 0 | 0 | 2.684664 | 0 | 0 |
| miR-184 | 17.5638127 | 11.6220226 | 50.789542 | 0.6347018 | 105.685771 |
| miR-184-3p | 11.70921 | 10.758396 | 25.148212 | 0.4480248 | 106.88278 |
| miR-184a | 0.964288 | 1.08571 | 1.479306 | 0.0373354 | 11.27182 |
| miR-185 | 0.2410716 | 0.1727264 | 5.999406 | 0.392021 | 0 |
| miR-185-5p | 0.2066328 | 0.1480512 | 4.931016 | 0.336018 | 0 |
| miR-186 | 0.5510216 | 0.9870072 | 2.41072 | 0 | 0 |
| miR-186-5p | 0.6198993 | 0.9870076 | 1.890225 | 0 | 0 |
| miR-188 | 0 | 0 | 4.60228 | 0 | 0 |
| miR-188-5p | 0 | 0 | 1.561488 | 0 | 0 |
| miR-18a | 11.1581737 | 0 | 27.887635 | 0 | 0 |
| miR-18a-5p | 18.080361 | 0 | 56.21358 | 0 | 0 |
| miR-18b | 0.344388 | 0 | 1.36973 | 0 | 0 |
| miR-18b-5p | 1.1709198 | 0 | 2.9312171 | 0 | 0 |
| miR-19 | 1.34311 | 0.197402 | 0.273945 | 0 | 0 |
| miR-190a | 0 | 0.0740256 | 1.8080386 | 0 | 0 |
| miR-190a-5p | 0 | 0.0987008 | 1.917617 | 0 | 0 |
| miR-190b | 0.1377552 | 0 | 1.3971204 | 0 | 0 |
| miR-191 | 12.914561 | 0.888308 | 120.78248 | 0.2986824 | 0 |
| miR-191-5p | 20.18115 | 1.554539 | 187.0772 | 0.4106883 | 0 |
| miR-191a | 1.99745 | 0.222077 | 18.9296 | 0.0373353 | 0 |
| miR-191a-5p | 1.99745 | 0.222077 | 18.9296 | 0.0373353 | 0 |
| miR-192 | 0.6198993 | 7.6493152 | 0.1917621 | 0 | 0 |
| miR-192-5p | 0.4821439 | 5.2804928 | 0 | 0 | 0 |
| miR-193a-5p | 1.205358 | 0 | 0.1917615 | 0 | 0 |
| miR-193b-3p | 0.4132662 | 0.0493504 | 2.027199 | 0 | 0 |
| miR-194 | 1.1020432 | 1.23376 | 1.3149376 | 0 | 0 |
| miR-195 | 2.479593 | 0 | 22.92927 | 0 | 0 |
| miR-195-5p | 0.964286 | 0 | 9.67029 | 0 | 0 |
| miR-195a-5p | 0.137755 | 0 | 2.0272 | 0 | 0 |
| miR-196 | 0 | 0 | 2.739453 | 0 | 0.3491271 |
| miR-196a | 0 | 0 | 5.670667 | 0 | 0.6483789 |
| miR-196a-5p | 0 | 0.0246752 | 5.2049605 | 0 | 0.498753 |
| miR-199 | 0.2755116 | 0.444154 | 14.19038 | 0 | 0 |
| miR-199-3p | 0.1377552 | 0.814283 | 22.901844 | 0 | 0 |
| miR-199-5p | 0.4477064 | 0 | 4.109181 | 0 | 0 |
| miR-199a | 0.8609748 | 0.197402 | 12.30015 | 0 | 0 |
| miR-199a-3p | 0.6543372 | 4.0960882 | 108.455028 | 0 | 0 |
| miR-199a-5p | 1.6186318 | 0 | 13.889029 | 0 | 0 |
| miR-199b | 0.0688776 | 0.444154 | 11.697473 | 0 | 0 |
| miR-199b-3p | 0.2410716 | 1.5051892 | 40.571328 | 0 | 0 |
| miR-199c-3p | 0.0688776 | 0.419479 | 11.94402 | 0 | 0 |
| miR-19a | 5.5791 | 0.4441536 | 1.972404 | 0 | 0 |
| miR-19a-3p | 3.306134 | 0.2961024 | 1.150569 | 0 | 0 |
| miR-19b | 25.9323557 | 3.9480392 | 5.314533 | 0 | 0 |
| miR-19b-3p | 17.46043 | 2.566226 | 3.561285 | 0 | 0 |
| miR-1a | 427.7992 | 305.8002 | 2478.356 | 2504.788 | 2974.562 |
| miR-1a-3p | 1468.12734 | 889.88549 | 5984.36516 | 6912.1952 | 7719.75015 |
| miR-1b | 351.7925488 | 234.3400504 | 1765.331679 | 1879.121677 | 2176.957 |
| miR-1b-3p | 276.37151 | 163.1273504 | 1052.662679 | 1253.456677 | 1383.74 |
| miR-1b-5p | 350.7596 | 234.094 | 1763.549 | 1878.713 | 2176.906 |
| miR-1c | 273.238 | 161.919 | 1047.9 | 1248.68 | 1381.25 |
| miR-1c-3p | 282.192 | 164.238 | 1056.28 | 1257.6 | 1385.14 |
| miR-1c-5p | 282.192 | 164.238 | 1056.28 | 1257.6 | 1385.14 |
| miR-2 | 0.688775 | 0 | 0 | 0 | 1.1471309 |
| miR-20 | 7.438794 | 0.8142822 | 182.721558 | 0 | 0 |
| miR-200 | 4.47705 | 0.56753 | 1.588885 | 3.52819 | 1.64588 |
| miR-200-3p | 4.13266 | 0.518179 | 1.20536 | 3.52819 | 1.54613 |
| miR-200a | 0 | 0.9129824 | 34.571903 | 0 | 0 |
| miR-200a-3p | 0 | 0.9376576 | 35.366345 | 0 | 0 |
| miR-200b | 4.5803664 | 0.7155812 | 5.204968 | 3.52819 | 1.64588 |
| miR-200b-3p | 0.0344388 | 0.2961024 | 7.149976 | 0 | 0 |
| miR-200b-5p | 4.47705 | 0.56753 | 1.26015 | 3.52819 | 1.64588 |
| miR-201-5p | 0.0688777 | 0 | 4.65707 | 0 | 0 |
| miR-202 | 0.0688776 | 0.0493504 | 9.64288 | 0 | 0 |
| miR-202-3p | 0 | 0 | 28.43554 | 0 | 0 |
| miR-202-5p | 0.2066328 | 0.1480512 | 22.490922 | 0 | 0 |
| miR-203 | 0.1377552 | 0.7649312 | 1.5614885 | 0.1493412 | 0 |
| miR-204 | 0 | 0.370128 | 14.436924 | 0 | 0 |
| miR-204-5p | 0 | 0.246752 | 9.58809 | 0 | 0 |
| miR-204a | 0 | 0.0493504 | 1.917618 | 0 | 0 |
| miR-205 | 29.238526 | 31.707597 | 18.13518 | 10.883256 | 10.374062 |
| miR-205-5p | 5.64796 | 6.02074 | 3.5065 | 2.09078 | 1.995012 |
| miR-205a | 5.64796 | 6.11944 | 3.5065 | 2.09078 | 1.995012 |
| miR-205a-5p | 2.82398 | 3.30648 | 1.75325 | 1.04539 | 0.997506 |
| miR-205b-5p | 1.41199 | 1.52986 | 0.876625 | 0.522695 | 0.498753 |
| miR-206 | 4.13266 | 0.4194784 | 8.21836 | 7.616407 | 6.98254 |
| miR-206-3p | 1.446431 | 0.1480512 | 2.876426 | 2.669476 | 2.443889 |
| miR-20a | 9.2640548 | 0.9870088 | 218.38921 | 0 | 0 |
| miR-20a-5p | 26.139102 | 2.7389496 | 565.203937 | 0 | 0 |
| miR-21 | 7.7487396 | 93.93856 | 76.869052 | 1.642751 | 1.0972566 |
| miR-210 | 0.1377554 | 0.1480512 | 0.9588105 | 0.9520517 | 2.892765 |
| miR-210-3p | 0 | 0.1480512 | 0.9588105 | 1.0827257 | 1.097256 |
| miR-21-3p | 0.1033164 | 0.3454524 | 1.068386 | 0 | 0 |
| miR-21-5p | 8.988538 | 108.39824 | 90.23758 | 1.941433 | 1.2967578 |
| miR-218 | 1.0331655 | 0 | 6.273348 | 0 | 0 |
| miR-218-5p | 0.8265324 | 0 | 5.25975 | 0 | 0 |
| miR-218a | 0.5510216 | 0 | 3.944812 | 0 | 0 |
| miR-218b | 0.1377554 | 0 | 1.205359 | 0 | 0 |
| miR-219 | 3819.8615 | 81.62552 | 23.394939 | 53.501595 | 67.88035 |
| miR-219-5p | 1936.602055 | 43.37901 | 10.327742 | 25.31337 | 29.77557 |
| miR-219a | 204.7047 | 4.54024 | 1.013598 | 2.781479 | 3.341645 |
| miR-219a-5p | 406.1721 | 9.42593 | 2.027196 | 4.946937 | 5.386535 |
| miR-219b | 1.480873 | 0.0246752 | 0.219156 | 0.6160333 | 1.147131 |
| miR-219b-3p | 177.4288 | 4.34284 | 0.712258 | 1.941438 | 1.79551 |
| miR-219b-5p | 140.442 | 2.91167 | 0.958809 | 2.03478 | 2.69327 |
| miR-219c-5p | 140.442 | 2.91167 | 0.958809 | 2.03478 | 2.69327 |
| miR-21a-5p | 0.654338 | 7.22984 | 6.90342 | 0.149341 | 0.0997506 |
| miR-22 | 54.482251 | 29.116762 | 4831.32907 | 6.3843377 | 5.486278 |
| miR-221 | 4.132665 | 21.6648756 | 1.2053584 | 1.68009 | 0 |
| miR-221-3p | 4.408176 | 23.14539 | 1.3697255 | 1.792096 | 0 |
| miR-221-5p | 0.275511 | 1.35714 | 0.0547891 | 0.112006 | 0 |
| miR-223 | 0 | 1.3324608 | 3.040791 | 0 | 0 |
| miR-223-3p | 0 | 0.4441536 | 1.068387 | 0 | 0 |
| miR-22-3p | 38.674821 | 20.9492632 | 3350.653927 | 4.6669138 | 4.788026 |
| miR-22-5p | 0.8265354 | 0 | 5.94462 | 0 | 0 |
| miR-22a | 4.1671 | 2.3194708 | 374.866924 | 0.485359 | 0.399002 |
| miR-22a-3p | 0 | 0.0493504 | 1.698462 | 0 | 0 |
| miR-22b | 4.3048552 | 2.22077 | 374.757344 | 0.485359 | 0.399002 |
| miR-22b-3p | 0.0688776 | 0 | 1.643672 | 0 | 0 |
| miR-23-3p | 0.103317 | 0 | 1.0136 | 0 | 0 |
| miR-236 | 1036.677733 | 656.4102 | 156.97104 | 142.8076 | 86.882716 |
| miR-236-3p | 252.781 | 224.3472 | 14.79304 | 4.6296 | 2.992516 |
| miR-23a | 11.0204344 | 2.6649202 | 27.449351 | 5.525631 | 5.985024 |
| miR-23a-3p | 5.44134 | 1.2337594 | 13.532919 | 2.520136 | 2.743136 |
| miR-23b | 1.6186326 | 0 | 23.559302 | 0.0186677 | 0 |
| miR-23b-3p | 1.343121 | 0 | 16.1354 | 0 | 0 |
| miR-24 | 1.239795 | 1.110384 | 20.95686 | 0 | 0 |
| miR-24-3p | 2.2385188 | 2.0233664 | 37.366218 | 0 | 0 |
| miR-2478 | 0.378827 | 2.61557 | 0.410918 | 0 | 0.149626 |
| miR-24a | 0.137755 | 0.123376 | 2.32854 | 0 | 0 |
| miR-24a-3p | 0.1721938 | 0.1727264 | 2.438118 | 0 | 0 |
| miR-25 | 8.781911 | 11.7700772 | 18.16263 | 0 | 0 |
| miR-25-3p | 4.132664 | 5.527248 | 8.54712 | 0 | 0 |
| miR-26 | 1.446431 | 2.048041 | 109.3589 | 0.0373354 | 0 |
| miR-263a | 1.3086758 | 0.4441536 | 2.191562 | 1.4934138 | 3.1920194 |
| miR-263a-5p | 0.7576537 | 0.2220768 | 1.424518 | 0.9893864 | 3.7905236 |
| miR-26-5p | 1.7563817 | 2.344144 | 123.30282 | 0.0560031 | 0 |
| miR-26a | 7.3010397 | 8.858397 | 509.12772 | 0.2613478 | 0 |
| miR-26a-5p | 4.787002 | 5.823347 | 338.0213 | 0.1680093 | 0 |
| miR-26b | 0.1377554 | 3.035049 | 106.51016 | 0.2613471 | 0.3491271 |
| miR-26b-5p | 0.1377554 | 3.035049 | 105.71566 | 0.2613471 | 0.3491271 |
| miR-26c | 1.033166 | 1.23376 | 72.4586 | 0.0373354 | 0 |
| miR-27-3p | 0.137755 | 0.641555 | 5.47891 | 0.0373353 | 0 |
| miR-275 | 1.859706 | 0.8883072 | 3.451716 | 0 | 2.693268 |
| miR-276 | 2.58291 | 5.996075 | 0.0547891 | 0.130674 | 5.8354046 |
| miR-276-3p | 1.7908177 | 4.4662118 | 0.164367 | 0.0933383 | 2.593514 |
| miR-276-5p | 0.5510208 | 0.2220768 | 0 | 0.448025 | 8.8777953 |
| miR-276a | 2.755104 | 6.711656 | 0 | 0 | 3.192016 |
| miR-276a-3p | 1.377552 | 3.355828 | 0 | 0 | 1.596008 |
| miR-276a-5p | 0.516582 | 0.2220768 | 0 | 0.392022 | 8.82792 |
| miR-276b-5p | 0.516582 | 0.2220768 | 0 | 0.392022 | 8.82792 |
| miR-277 | 1.8252666 | 0.9376576 | 0 | 0.7093707 | 0 |
| miR-277a | 67.569 | 28.2531 | 15.5053 | 22.1399 | 22.394 |
| miR-277c-3p | 30.5128 | 26.4518 | 12.218 | 14.8968 | 17.9052 |
| miR-278 | 1.343121 | 0.6415552 | 0 | 0 | 0 |
| miR-279 | 1.343121 | 3.035045 | 0 | 0 | 2.2942653 |
| miR-279a | 0.103317 | 1.3818118 | 0 | 0 | 0.6483793 |
| miR-27a | 0.5854597 | 1.6779136 | 9.9442186 | 0 | 0 |
| miR-27a-3p | 0.551021 | 1.3818112 | 8.656675 | 0 | 0 |
| miR-27b | 1.65306 | 7.797363 | 65.77431 | 0.4480236 | 0 |
| miR-27b-3p | 2.20408 | 10.338908 | 87.66256 | 0.5973648 | 0 |
| miR-27d | 0 | 0.1480512 | 3.94482 | 0 | 0 |
| miR-28 | 0 | 0.0246752 | 12.16318 | 0 | 0 |
| miR-281 | 116.1624058 | 144.3008544 | 424.8893036 | 116.9713836 | 89.027426 |
| miR-281-2-5p | 1.03317 | 0.493504 | 0 | 0 | 0.997506 |
| miR-281-3p | 6.6467 | 0.4688288 | 1.3423316 | 0.8213776 | 1.645886 |
| miR-281a | 0.723216 | 0.394803 | 4.05439 | 1.40007 | 0.648379 |
| miR-282 | 3.78827 | 0 | 0 | 0 | 0 |
| miR-28-5p | 0 | 0 | 8.43752 | 0 | 0 |
| miR-28a-5p | 0 | 0 | 1.20536 | 0 | 0 |
| miR-29 | 0 | 0 | 4.54749 | 0 | 0 |
| miR-296 | 0.378827 | 0 | 2.4381141 | 0 | 0 |
| miR-296-3p | 1.894135 | 0 | 10.601685 | 0 | 0 |
| miR-296-5p | 2.755105 | 0 | 0.821835 | 0 | 0 |
| miR-298-5p | 0.3788268 | 0 | 1.780644 | 0 | 0 |
| miR-29a | 14.464327 | 2.097394 | 514.223069 | 1.6427527 | 0 |
| miR-29a-3p | 7.335481 | 1.08571 | 262.87788 | 0.7093702 | 0 |
| miR-29b | 12.5701926 | 3.429854 | 2833.49861 | 2.090776 | 0 |
| miR-29b-3p | 11.8125376 | 3.084402 | 2547.774344 | 2.202782 | 0 |
| miR-29c | 2.996182 | 0.172726 | 158.47728 | 1.008054 | 0 |
| miR-29c-3p | 1.3775556 | 0 | 26.983632 | 0.448024 | 0 |
| miR-29c-5p | 0.1377552 | 0 | 1.09578 | 0 | 0 |
| miR-2a | 4.5803538 | 6.3908856 | 0.383524 | 0.709372 | 4.1396463 |
| miR-2a-3p | 1.7563776 | 0.641555 | 0.1095781 | 0.298683 | 0.9476299 |
| miR-2b | 9.0229686 | 3.3064728 | 0.1643672 | 2.16544 | 16.5586196 |
| miR-2b-3p | 1165.237665 | 4136.35037 | 35.9964672 | 217.49744 | 176.109279 |
| miR-2c | 0.413265 | 0 | 0 | 0.0933383 | 1.1471313 |
| miR-2c-3p | 32.510355 | 15.94018 | 96.8672 | 99.8346 | 101.9450516 |
| miR-301-3p | 0.413265 | 0.1480512 | 1.232754 | 0 | 0 |
| miR-301a | 0.8609688 | 0.2961024 | 2.6846645 | 0 | 0 |
| miR-301a-3p | 1.10204 | 0.3948032 | 3.287344 | 0 | 0 |
| miR-304 | 6.508944 | 0 | 0 | 0 | 1.346634 |
| miR-305 | 10.882676 | 1.5792128 | 0 | 0 | 20.74816 |
| miR-305-5p | 3.099496 | 0.5428544 | 0 | 0 | 6.284296 |
| miR-3068-5p | 0 | 0 | 1.80804 | 0 | 0 |
| miR-308 | 0.344388 | 0 | 0 | 0 | 1.49626 |
| miR-30a | 0.482144 | 0.0493504 | 6.60208 | 0 | 0 |
| miR-30a-3p | 1.653071 | 0.0740256 | 51.419561 | 0 | 0 |
| miR-30a-5p | 6.5778205 | 1.1350586 | 46.7899207 | 0 | 0 |
| miR-30b | 13.8788516 | 0.4441536 | 28.3259273 | 0 | 0 |
| miR-30b-3p | 0.0688777 | 0 | 2.246351 | 0 | 0 |
| miR-30b-5p | 20.0778417 | 0.740256 | 47.3651191 | 0 | 0 |
| miR-30c | 0.9987265 | 0 | 1.0683868 | 0 | 0 |
| miR-30c-3p | 0.3099501 | 0 | 3.753053 | 0 | 0 |
| miR-30c-5p | 1.136482 | 0.0987008 | 3.6434768 | 0 | 0 |
| miR-30d | 4.9591968 | 0.493504 | 33.1748256 | 0 | 0 |
| miR-30d-3p | 0.103317 | 0 | 4.492705 | 0 | 0 |
| miR-30d-5p | 3.30613 | 0.4194784 | 24.84687 | 0 | 0 |
| miR-30e | 2.2729638 | 0.6909056 | 22.65529 | 0 | 0 |
| miR-30e-3p | 0.4132662 | 0.5922048 | 41.6397231 | 0 | 0 |
| miR-30e-5p | 3.340568 | 1.1597344 | 23.0936041 | 0 | 0 |
| miR-31 | 3.6160748 | 0.4441536 | 7.3143561 | 1.6427561 | 0.5486283 |
| miR-312-3p | 0.0344388 | 0.0246752 | 0 | 0 | 1.2967606 |
| miR-313-5p | 0 | 0 | 0 | 0 | 1.09726 |
| miR-314 | 3.78827 | 0 | 0 | 0 | 0 |
| miR-31-5p | 5.510208 | 0.61688 | 9.3689522 | 2.5761394 | 0.6982542 |
| miR-317 | 7.0599658 | 0.5428544 | 1.0409929 | 1.064057 | 0 |
| miR-317-3p | 1.859698 | 0.1480512 | 0.3287346 | 0.336018 | 0 |
| miR-318 | 5.303584 | 1.0857088 | 0 | 0 | 6.034908 |
| miR-3184-3p | 0.0344388 | 0.0493504 | 1.28754 | 0.0186677 | 0 |
| miR-31a | 2.686227 | 0.0987008 | 1.47931 | 0.448024 | 1.0972566 |
| miR-320 | 0.619899 | 8.88309 | 6.57469 | 1.68009 | 1.147131 |
| miR-320-3p | 0.619899 | 8.88309 | 6.41031 | 1.68009 | 1.047381 |
| miR-320a | 1.446431 | 20.72721 | 14.95739 | 3.92021 | 2.443889 |
| miR-320b | 0.826532 | 11.3506 | 4.82144 | 2.24012 | 1.396508 |
| miR-322-5p | 0.0688776 | 0.0493504 | 16.65587 | 0 | 0 |
| miR-324 | 0.3443898 | 0 | 19.696664 | 0 | 0 |
| miR-324-5p | 0.8609748 | 0 | 51.474314 | 0 | 0 |
| miR-328 | 1.10204 | 0 | 15.56008 | 0 | 0 |
| miR-328-3p | 0.413265 | 0 | 5.83503 | 0 | 0 |
| miR-328a-3p | 0.137755 | 0 | 1.94501 | 0 | 0 |
| miR-33 | 15.704116 | 1.1103836 | 5.7254556 | 1.2320652 | 0.9476308 |
| miR-335 | 0 | 0 | 2.465505 | 0 | 0 |
| miR-335-5p | 0 | 0 | 1.314936 | 0 | 0 |
| miR-33-5p | 22.316351 | 1.727262 | 7.8896226 | 2.0907767 | 1.8453868 |
| miR-338 | 0 | 0.6909056 | 3.068184 | 0 | 0 |
| miR-338-3p | 0 | 0.3948032 | 2.081982 | 0 | 0 |
| miR-339 | 0.5165828 | 0 | 4.931021 | 0 | 0 |
| miR-339-5p | 1.446432 | 0 | 15.45055 | 0 | 0 |
| miR-339a | 0.241072 | 0 | 2.0272 | 0 | 0 |
| miR-339b | 0.172194 | 0 | 1.15057 | 0 | 0 |
| miR-33a | 5.923485 | 0.4194784 | 2.136773 | 0.4666914 | 0.2992518 |
| miR-33a-5p | 7.98981 | 0.5675292 | 2.931214 | 0.6347002 | 0.5486284 |
| miR-33b | 3.47832 | 0 | 0 | 0 | 0.0498753 |
| miR-33b-5p | 1.51531 | 0.0987008 | 0.575285 | 0.0933383 | 0.0997506 |
| miR-34 | 25.278139 | 2.9363424 | 21.5595106 | 8.6431244 | 35.660872 |
| miR-340 | 0.2755108 | 0.5181792 | 12.1357945 | 0 | 0 |
| miR-340-5p | 0.4821439 | 0.863632 | 20.13501 | 0 | 0 |
| miR-342 | 0.206633 | 0 | 17.422931 | 0 | 0 |
| miR-342-3p | 0.3099493 | 0 | 33.202178 | 0 | 0 |
| miR-34-5p | 12.880141 | 1.5545344 | 0.6848634 | 4.7229167 | 18.703246 |
| miR-3473b | 0.172194 | 0 | 17.7243 | 0.056003 | 0 |
| miR-34a | 4.063783 | 1.5051872 | 394.865 | 0 | 0 |
| miR-34a-5p | 41.016636 | 1.9246654 | 378.0447836 | 0.5786973 | 2.04489 |
| miR-34b | 40.8444374 | 0.962333 | 196.720359 | 0.0933383 | 0 |
| miR-34b-3p | 5.682411 | 0 | 6.519913 | 0 | 0 |
| miR-34b-5p | 92.1239028 | 0.0740256 | 154.58732 | 0 | 0 |
| miR-34c | 109.9287 | 2.886999 | 549.3432 | 0.2800149 | 0 |
| miR-34c-3p | 0.723216 | 0 | 1.150572 | 0 | 0 |
| miR-34c-5p | 356.54503 | 9.62333 | 1742.3492 | 0.933383 | 0 |
| miR-351-5p | 0 | 0 | 3.45172 | 0 | 0 |
| miR-3529-3p | 0.172194 | 0.61688 | 2.32854 | 0 | 0.548628 |
| miR-3571 | 111.754 | 23.2194 | 199.734 | 118.484 | 139.002 |
| miR-3586-3p | 0.688777 | 0.0740256 | 18.3817 | 0 | 0 |
| miR-3587 | 0.0688777 | 0.0246752 | 20.6829 | 0.261347 | 0 |
| miR-3588 | 0.482144 | 0.0493504 | 11.04 | 0 | 0.0498753 |
| miR-3591-3p | 0 | 4.61426 | 0 | 0 | 0 |
| miR-3596 | 0.3788268 | 0.9129824 | 65.63737 | 0.56003 | 0.199501 |
| miR-3596a | 1.51531 | 1.11038 | 82.5398 | 0.261347 | 3.04239 |
| miR-3596c | 0 | 0.0246752 | 2.79424 | 0 | 0 |
| miR-3596d | 0.0344388 | 0.0493504 | 5.17757 | 0 | 0 |
| miR-3600 | 4.1671 | 2.22077 | 371.47 | 0.485359 | 0.399002 |
| miR-3604 | 0.0344388 | 0.222077 | 5.78025 | 0 | 0 |
| miR-362-5p | 0.2755104 | 0 | 1.753248 | 0 | 0 |
| miR-369-3p | 0 | 1.628561 | 0 | 0 | 0 |
| miR-374b-5p | 0 | 0.2961024 | 1.123178 | 0 | 0 |
| miR-375 | 1425.906204 | 3679.764975 | 1582.527676 | 1586.770116 | 1007.331193 |
| miR-375-3p | 657.989093 | 1688.056226 | 811.480764 | 784.881543 | 441.546114 |
| miR-375a-3p | 80.0359 | 207.173 | 85.3066 | 88.4474 | 55.6608 |
| miR-375b-3p | 79.657 | 207.05 | 85.1696 | 88.3914 | 55.6608 |
| miR-378 | 0.172194 | 0.8883088 | 1.287542 | 0 | 0 |
| miR-379 | 0 | 0 | 1.534096 | 0 | 0 |
| miR-379-5p | 0 | 0 | 1.150572 | 0 | 0 |
| miR-380-3p | 0 | 1.406487 | 0 | 0 | 0 |
| miR395 | 0 | 0 | 1.890222 | 0 | 0 |
| miR395a | 0 | 0 | 8.190962 | 0 | 0 |
| miR395b | 0 | 0 | 7.560888 | 0 | 0 |
| miR395c | 0 | 0 | 6.930814 | 0 | 0 |
| miR395d | 0 | 0 | 6.3555291 | 0 | 0 |
| miR395d-3p | 0 | 0 | 1.260148 | 0 | 0 |
| miR395e | 0 | 0 | 5.0953811 | 0 | 0 |
| miR395f | 0 | 0 | 3.8352331 | 0 | 0 |
| miR395g | 0 | 0 | 3.2051591 | 0 | 0 |
| miR395h | 0 | 0 | 3.2051591 | 0 | 0 |
| miR395i | 0 | 0 | 3.15037 | 0 | 0 |
| miR395j | 0 | 0 | 2.520296 | 0 | 0 |
| miR395k | 0 | 0 | 1.890222 | 0 | 0 |
| miR395l | 0 | 0 | 1.260148 | 0 | 0 |
| miR395m | 0 | 0 | 1.260148 | 0 | 0 |
| miR396 | 0 | 1.7766156 | 0.410919 | 0 | 0 |
| miR396a | 0 | 2.0233672 | 0.273946 | 0 | 0 |
| miR396a-5p | 0 | 1.2831112 | 0.273946 | 0 | 0 |
| miR396b | 0 | 3.5779072 | 1.095784 | 0 | 0 |
| miR396b-5p | 0 | 1.3077868 | 0.410919 | 0 | 0 |
| miR396c | 0 | 3.133754 | 1.232757 | 0 | 0 |
| miR396d | 0 | 1.4558384 | 0.547892 | 0 | 0 |
| miR396e | 0 | 1.727266 | 0.684865 | 0 | 0 |
| miR396e-5p | 0 | 1.08571 | 0.410919 | 0 | 0 |
| miR403a | 0 | 1.5792092 | 0.7944415 | 0.3360177 | 0 |
| miR403b | 0 | 1.2337572 | 0.6300743 | 0.2613471 | 0 |
| miR-423 | 0.1033165 | 0.0740256 | 1.725852 | 0.0373354 | 0 |
| miR-423-3p | 0.7576547 | 0.2714272 | 1.808037 | 0 | 0 |
| miR-423-5p | 0.344388 | 0.493504 | 12.8754 | 0.186677 | 0 |
| miR-423a | 0.0344388 | 0.0493504 | 1.28754 | 0.0186677 | 0 |
| miR-424 | 0 | 0 | 3.287345 | 0 | 0 |
| miR-424-5p | 0 | 0 | 3.944814 | 0 | 0 |
| miR-425 | 3.58164 | 0.1974016 | 15.9984 | 0 | 0 |
| miR-425-3p | 0 | 0 | 1.09578 | 0 | 0 |
| miR-425-5p | 8.9541 | 0.493504 | 40.0781836 | 0 | 0 |
| miR-429 | 0 | 0 | 16.436765 | 0 | 0 |
| miR-429-3p | 0 | 0 | 7.807464 | 0 | 0 |
| miR-429a | 0 | 0 | 2.109384 | 0 | 0 |
| miR-4334-3p | 0.172194 | 0 | 1.15057 | 0 | 0 |
| miR-449 | 7.0255188 | 0.0987008 | 60.8981 | 0 | 0 |
| miR-449a | 34.645471 | 0.493504 | 311.09252 | 0 | 0 |
| miR-449a-5p | 27.792143 | 0.3948032 | 247.4275845 | 0 | 0 |
| miR-450a | 0 | 0 | 2.547691 | 0 | 0 |
| miR-450a-5p | 0 | 0 | 1.6984611 | 0 | 0 |
| miR-451 | 1.928573 | 1.3818096 | 10.574286 | 0 | 0.1995012 |
| miR-451-?? | 0.241072 | 0.172726 | 1.39712 | 0 | 0.0498753 |
| miR-451-5p | 0.27551 | 0.1974016 | 1.424516 | 0 | 0 |
| miR-451a | 0.27551 | 0.1974016 | 1.424516 | 0 | 0 |
| miR-465a-3p | 0.378827 | 0 | 2.16417 | 0 | 0 |
| miR-465a-5p | 0.206633 | 0 | 3.28735 | 0 | 0 |
| miR-465b-3p | 0.378827 | 0 | 2.16417 | 0 | 0 |
| miR-465b-5p | 0.0688777 | 0.0740256 | 1.39712 | 0 | 0 |
| miR-465c-3p | 0.378827 | 0 | 2.16417 | 0 | 0 |
| miR-465c-5p | 0.378827 | 0.0987008 | 12.5193 | 0 | 0 |
| miR-466i-5p | 1.30868 | 0.222077 | 1.28754 | 0.80271 | 1.74564 |
| miR-467e-5p | 0.241072 | 0 | 3.04079 | 0 | 0 |
| miR-470-5p | 0.103317 | 0.123376 | 14.2726 | 0 | 0 |
| miR-471-5p | 0.27551 | 0 | 3.61608 | 0 | 0 |
| miR-497 | 1.446435 | 0 | 6.930825 | 0 | 0 |
| miR-497-5p | 1.239801 | 0 | 5.341941 | 0 | 0 |
| miR-497a-5p | 0.30995 | 0 | 1.0136 | 0 | 0 |
| miR-499 | 0 | 0.2961024 | 1.80804 | 0 | 0 |
| miR-499-5p | 0 | 0.5922048 | 3.61608 | 0 | 0 |
| miR-500-3p | 0 | 0 | 2.35594 | 0 | 0 |
| miR-501-3p | 0 | 0 | 1.643672 | 0 | 0 |
| miR-503 | 0.0688776 | 0 | 14.546507 | 0 | 0 |
| miR-503-5p | 0.0688776 | 0 | 14.546507 | 0 | 0 |
| miR-511-3p | 0 | 0 | 1.040993 | 0 | 0 |
| miR-532 | 0.0344388 | 0 | 2.629876 | 0 | 0.0498753 |
| miR-532-3p | 0.2755104 | 0 | 1.479307 | 0 | 0.3990024 |
| miR-532-5p | 0 | 0 | 6.903423 | 0 | 0 |
| miR-547-3p | 0 | 0 | 2.27375 | 0 | 0 |
| miR5658 | 3.37501 | 3.60258 | 0.712258 | 5.09627 | 2.09476 |
| miR-652 | 0 | 0.1974016 | 16.21756 | 0.7467064 | 0.3990024 |
| miR-652-3p | 0 | 0.123376 | 10.10858 | 0.4666915 | 0.2493765 |
| miR-669c-5p | 0 | 0.0246752 | 1.06839 | 0 | 0 |
| miR-672 | 0.0688776 | 0 | 5.86244 | 0 | 0 |
| miR-672-5p | 0.0688776 | 0 | 5.86244 | 0 | 0 |
| miR-674-5p | 0 | 0 | 2.41072 | 0 | 0 |
| miR-7 | 6.922202 | 12.2142196 | 31.03804 | 0 | 4.3391496 |
| miR-708 | 0 | 0 | 1.506698 | 0 | 0 |
| miR-708-5p | 0 | 0 | 1.917615 | 0 | 0 |
| miR-71 | 263342.3511 | 79755.92267 | 103297.8581 | 250059.6763 | 190127.9273 |
| miR-71-5p | 217919.7162 | 43457.2034 | 81683.868 | 201079.558 | 166888.039 |
| miR-71a-5p | 52.932517 | 2.7142704 | 8.464918 | 20.963824 | 13.865301 |
| miR-71c-5p | 32675.8 | 5179.45 | 10848.9 | 28103.6 | 24600.3 |
| miR-72 | 11.158187 | 0.4441528 | 13.69727 | 3.360184 | 1.2967586 |
| miR-72-5p | 17.219424 | 0.7155796 | 21.28556 | 5.264288 | 1.9950132 |
| miR-72a | 0 | 0.0493504 | 1.12318 | 0.130674 | 0.0498753 |
| miR-741-3p | 0.482144 | 0.0246752 | 18.9844 | 0 | 0 |
| miR-743a-3p | 0.447705 | 0 | 10.8208 | 0 | 0 |
| miR-743b-3p | 0.137755 | 0 | 1.06839 | 0 | 0 |
| miR-744 | 0 | 0 | 5.88985 | 0 | 0 |
| miR-744-5p | 0 | 0 | 2.986014 | 0 | 0 |
| miR-7-5p | 2.892859 | 6.637627 | 20.354169 | 0 | 3.8403963 |
| miR-79 | 0.7576547 | 0 | 0 | 0.1680089 | 14.9126806 |
| miR-79-3p | 0.2755108 | 0 | 0.0821836 | 0.1306736 | 8.57854 |
| miR-7a | 0.86097 | 2.048041 | 6.46512 | 0 | 1.2468819 |
| miR-7a-5p | 0.688776 | 1.085708 | 2.41072 | 0 | 0.1995012 |
| miR-7b | 0.447705 | 0.6415552 | 2.766853 | 0.0186677 | 0.548628 |
| miR-7c | 0.172194 | 0.61688 | 2.32854 | 0 | 0.548628 |
| miR-8 | 61436.45193 | 11024.57 | 17462.96099 | 45754.42536 | 29461.97205 |
| miR-8-3p | 121.74113 | 15.520734 | 30.325785 | 133.75386 | 145.68555 |
| miR-8-5p | 0.4821439 | 0.1727264 | 0 | 0.784042 | 7.33166 |
| miR-871-5p | 0.275511 | 0 | 4.95841 | 0 | 0 |
| miR-872-5p | 0.516582 | 0 | 1.479306 | 0 | 0 |
| miR-878-3p | 0.172194 | 0 | 1.06839 | 0 | 0 |
| miR-878-5p | 0 | 0 | 1.34233 | 0 | 0 |
| miR-880-3p | 0.378827 | 0 | 6.93082 | 0 | 0 |
| miR-881-3p | 0.447705 | 0.222077 | 1.26015 | 0 | 0 |
| miR-9 | 2.0318892 | 0.8142816 | 7.122577 | 1.026722 | 2.8927646 |
| miR-92 | 18.94134 | 35.3596056 | 59.96661 | 0 | 0.0498753 |
| miR-92-3p | 1.928573 | 3.6766092 | 5.6158771 | 0 | 0.0997506 |
| miR-92a | 16.668379 | 31.2881908 | 49.6114804 | 0 | 0.6483789 |
| miR-92a-3p | 18.045931 | 33.2375352 | 54.8986233 | 0 | 0.0997506 |
| miR-92b | 2.9961764 | 9.7713894 | 6.9856022 | 0 | 1.4463837 |
| miR-92b-3p | 1.0331649 | 2.3688206 | 1.6436701 | 0 | 0.7481295 |
| miR-93 | 4.1670944 | 0.789607 | 27.394538 | 0 | 0 |
| miR-93-3p | 0 | 0 | 1.369726 | 0 | 0 |
| miR-93-5p | 6.8877576 | 1.332462 | 40.215182 | 0 | 0 |
| miR-9-5p | 2.8584204 | 1.2090848 | 10.601682 | 1.4934138 | 5.1870266 |
| miR-970 | 0.2066331 | 0.1480512 | 0 | 0 | 1.645884 |
| miR-970-3p | 0.3443885 | 0.246752 | 0 | 0 | 2.74314 |
| miR-98 | 0 | 0.3207776 | 69.80129 | 0 | 0 |
| miR-98-5p | 0 | 0.1480512 | 35.22938 | 0 | 0 |
| miR-989 | 0.9642877 | 0.66623 | 0 | 0 | 15.46135 |
| miR-989-3p | 0.344388 | 0.542854 | 0 | 0 | 10.67332 |
| miR-989a | 0.0688777 | 0.123376 | 0 | 0 | 3.29177 |
| miR-99 | 0.3788268 | 0.0740256 | 76.1568 | 0 | 0 |
| miR-993 | 26.931102 | 6.1688 | 103.113106 | 7.9524262 | 34.314156 |
| miR-993-3p | 0.516585 | 0 | 1.232755 | 0.0933385 | 0.74813 |
| miR-993a-3p | 14.980917 | 3.15843 | 51.474351 | 4.1068877 | 16.857826 |
| miR-993b-3p | 18.287 | 0.0493504 | 0.2191561 | 0.0373353 | 0.0997506 |
| miR-994-5p | 0.2066327 | 0 | 0 | 0 | 6.433912 |
| miR-995-3p | 0.1033164 | 0.0987008 | 0 | 0 | 7.730658 |
| miR-99-5p | 0.2410716 | 0.0740256 | 72.3489 | 0 | 0 |
| miR-996-3p | 0.8265318 | 0 | 0 | 0.0186677 | 2.443892 |
| miR-99a | 1.7563788 | 0.2714272 | 290.76574 | 0 | 0 |
| miR-99a-3p | 0 | 0 | 1.040993 | 0 | 0.2992518 |
| miR-99a-5p | 1.4808684 | 0.2714272 | 281.7802 | 0 | 0 |
| miR-99b | 0.172194 | 0.3207776 | 45.28318 | 0 | 0 |
| miR-99b-5p | 0 | 0.2961024 | 19.83366 | 0 | 0 |
| miR-9a | 3.099492 | 1.3324608 | 9.368928 | 1.3440726 | 3.591018 |
| miR-9a-5p | 81.4134236 | 42.3180304 | 71.965471 | 25.8546746 | 17.6059606 |
| miR-9b | 36.1263338 | 5.157114 | 26.7097 | 13.4967177 | 52.9675263 |
| miR-9b-5p | 0.7576538 | 0.6168802 | 1.8080395 | 0.2613473 | 10.3241773 |
| miR-9c | 0.344388 | 0.987008 | 0 | 0 | 8.97755 |
| miR-9c-5p | 0.1033164 | 0.2961024 | 0 | 0 | 2.693265 |
| miR-iab-4-3p | 1.653072 | 0 | 0 | 0 | 0 |

**Supplementary table 3. Top 10 piRNA-producing transcripts ranked by sequence complexity**

| Transcript ID | Annotation |
| --- | --- |
| c100550_g1 | n/a |
| c100543_g1 | Retrovirus-related Pol polyprotein from transposon 412 |
| c100501_g1 | Uncharacterized protein |
| c100559_g1 | n/a |
| c99380_g1 | Lian-Aa1 retrotransposon protein |
| c98987_g3 | Transposase domain-containing protein |
| c97394_g4 | n/a |
| c100394_g1 | ATP-dependent DNA helicase PIF1 |
| c97352_g5 | n/a |
| c100403_g2 | n/a |

Accession numbers: c100550_g1 (KR132223); c100543_g1 (KR132224, KR132225, KR132226); c100501_g1 (KR132227); c100559_g1 (KR132228, KR132229, KR132230, KR132231); c99380_g1 (KR132232), c98987_g3 (KR132233, KR132234, KR132235); c97394_g4 (KR132236); c100394_g1 (KR132237, KR132238, KR132239); c97352_g5 (KR132240); c100403_g2 (KR132241).

**Supplementary Table 4. Impact of *Macpiwi2* knockdown on piRNA production and transposon silencing**

| Contig ID | Start | End | Length |
| --- | --- | --- | --- |
| unitig_26198 | 567 | 8586 | 8019 |
| unitig_26950 | 2173 | 8693 | 6520 |
| unitig_29363 | 5564 | 12590 | 7026 |
| unitig_37027 | 2358 | 9065 | 6707 |
| uti_cns_0000018 | 10 | 22253 | 22243 |
| uti_cns_0000046 | 189620 | 195217 | 5597 |
| uti_cns_0000063 | 45527 | 80217 | 34690 |
| uti_cns_0000063 | 87038 | 96526 | 9488 |
| uti_cns_0000106 | 99886 | 106341 | 6455 |
| uti_cns_0000109 | 235064 | 246616 | 11552 |
| uti_cns_0000109 | 252387 | 279494 | 27107 |
| uti_cns_0000109 | 293043 | 340413 | 47370 |
| uti_cns_0000136 | 2286 | 13271 | 10985 |
| uti_cns_0000149 | 264143 | 286066 | 21923 |
| uti_cns_0000149 | 292034 | 299797 | 7763 |
| uti_cns_0000149 | 307289 | 324944 | 17655 |
| uti_cns_0000184 | 59008 | 105411 | 46403 |
| uti_cns_0000184 | 117018 | 125733 | 8715 |
| uti_cns_0000184 | 133517 | 140248 | 6731 |
| uti_cns_0000190 | 24179 | 31944 | 7765 |
| uti_cns_0000197 | 29045 | 43383 | 14338 |
| uti_cns_0000197 | 58193 | 66033 | 7840 |
| uti_cns_0000205 | 305584 | 316238 | 10654 |
| uti_cns_0000206 | 39045 | 56095 | 17050 |
| uti_cns_0000206 | 62880 | 78353 | 15473 |
| uti_cns_0000206 | 84142 | 90813 | 6671 |
| uti_cns_0000217 | 195393 | 222398 | 27005 |
| uti_cns_0000233 | 46448 | 72415 | 25967 |
| uti_cns_0000233 | 78329 | 116027 | 37698 |
| uti_cns_0000240 | 29543 | 34800 | 5257 |
| uti_cns_0000242 | 129611 | 135360 | 5749 |
| uti_cns_0000242 | 163464 | 173062 | 9598 |
| uti_cns_0000244 | 100700 | 116763 | 16063 |
| uti_cns_0000244 | 127819 | 156640 | 28821 |
| uti_cns_0000276 | 158589 | 193344 | 34755 |
| uti_cns_0000276 | 205363 | 213621 | 8258 |
| uti_cns_0000276 | 219509 | 227207 | 7698 |
| uti_cns_0000276 | 239838 | 304381 | 64543 |
| uti_cns_0000297 | 151852 | 164093 | 12241 |
| uti_cns_0000297 | 188618 | 223399 | 34781 |
| uti_cns_0000308 | 71787 | 91662 | 19875 |
| uti_cns_0000308 | 170226 | 178912 | 8686 |
| uti_cns_0000308 | 196373 | 213716 | 17343 |
| uti_cns_0000314 | 52330 | 59016 | 6686 |
| uti_cns_0000360 | 44918 | 53977 | 9059 |
| uti_cns_0000371 | 92835 | 118007 | 25172 |
| uti_cns_0000371 | 126732 | 134444 | 7712 |
| uti_cns_0000395 | 149355 | 165321 | 15966 |
| uti_cns_0000395 | 201641 | 215616 | 13975 |
| uti_cns_0000419 | 52262 | 63802 | 11540 |
| uti_cns_0000419 | 79299 | 86786 | 7487 |
| uti_cns_0000419 | 96700 | 110119 | 13419 |
| uti_cns_0000419 | 120882 | 129608 | 8726 |
| uti_cns_0000419 | 137260 | 147453 | 10193 |
| uti_cns_0000419 | 161702 | 175782 | 14080 |
| uti_cns_0000502 | 177930 | 234986 | 57056 |
| uti_cns_0000502 | 241754 | 254348 | 12594 |
| uti_cns_0000503 | 1981 | 17098 | 15117 |
| uti_cns_0000517 | 212828 | 219529 | 6701 |
| uti_cns_0000518 | 68936 | 79314 | 10378 |
| uti_cns_0000518 | 88090 | 117942 | 29852 |
| uti_cns_0000518 | 136592 | 165386 | 28794 |
| uti_cns_0000527 | 93070 | 98607 | 5537 |
| uti_cns_0000527 | 126775 | 220508 | 93733 |
| uti_cns_0000588 | 194461 | 229012 | 34551 |
| uti_cns_0000593 | 151853 | 161375 | 9522 |
| uti_cns_0000597 | 43637 | 49291 | 5654 |
| uti_cns_0000614 | 176971 | 206000 | 29029 |
| uti_cns_0000633 | 44646 | 50206 | 5560 |
| uti_cns_0000633 | 74469 | 99389 | 24920 |
| uti_cns_0000636 | 6783 | 13497 | 6714 |
| uti_cns_0000655 | 33922 | 55788 | 21866 |
| uti_cns_0000655 | 71116 | 92727 | 21611 |
| uti_cns_0000660 | 1 | 5818 | 5817 |
| uti_cns_0000661 | 60939 | 92845 | 31906 |
| uti_cns_0000662 | 183762 | 228073 | 44311 |
| uti_cns_0000662 | 237170 | 245620 | 8450 |
| uti_cns_0000694 | 27525 | 34634 | 7109 |
| uti_cns_0000694 | 44482 | 60408 | 15926 |
| uti_cns_0000722 | 34876 | 40626 | 5750 |
| uti_cns_0000722 | 65814 | 88812 | 22998 |
| uti_cns_0000722 | 94786 | 115893 | 21107 |
| uti_cns_0000722 | 123000 | 129594 | 6594 |
| uti_cns_0000750 | 24248 | 30924 | 6676 |
| uti_cns_0000762 | 1179 | 25168 | 23989 |
| uti_cns_0000768 | 73856 | 79276 | 5420 |
| uti_cns_0000768 | 87050 | 139278 | 52228 |
| uti_cns_0000788 | 49339 | 59690 | 10351 |
| uti_cns_0000788 | 68794 | 80868 | 12074 |
| uti_cns_0000788 | 87235 | 100593 | 13358 |
| uti_cns_0000814 | 114132 | 154735 | 40603 |
| uti_cns_0000814 | 161492 | 185479 | 23987 |
| uti_cns_0000852 | 19425 | 25049 | 5624 |
| uti_cns_0000867 | 47415 | 83162 | 35747 |
| uti_cns_0000867 | 90939 | 96622 | 5683 |
| uti_cns_0000867 | 111302 | 117944 | 6642 |
| uti_cns_0000887 | 52373 | 79327 | 26954 |
| uti_cns_0000905 | 25520 | 32882 | 7362 |
| uti_cns_0000908 | 77945 | 99585 | 21640 |
| uti_cns_0000908 | 106676 | 139267 | 32591 |
| uti_cns_0000941 | 88147 | 104274 | 16127 |
| uti_cns_0000941 | 120901 | 129419 | 8518 |
| uti_cns_0000941 | 135418 | 149772 | 14354 |
| uti_cns_0000941 | 155702 | 177870 | 22168 |
| uti_cns_0000959 | 4911 | 10477 | 5566 |
| uti_cns_0000984 | 33847 | 43547 | 9700 |
| uti_cns_0000984 | 88064 | 121869 | 33805 |
| uti_cns_0000984 | 148939 | 156526 | 7587 |
| uti_cns_0001056 | 12081 | 26647 | 14566 |
| uti_cns_0001056 | 33080 | 50267 | 17187 |
| uti_cns_0001088 | 65020 | 77374 | 12354 |
| uti_cns_0001095 | 48445 | 55151 | 6706 |
| uti_cns_0001157 | 77546 | 85112 | 7566 |
| uti_cns_0001160 | 39761 | 59019 | 19258 |
| uti_cns_0001160 | 101648 | 129245 | 27597 |
| uti_cns_0001163 | 89931 | 98606 | 8675 |
| uti_cns_0001163 | 115078 | 157633 | 42555 |
| uti_cns_0001186 | 13539 | 57064 | 43525 |
| uti_cns_0001186 | 73515 | 84119 | 10604 |
| uti_cns_0001217 | 23211 | 29019 | 5808 |
| uti_cns_0001217 | 42817 | 51209 | 8392 |
| uti_cns_0001217 | 78381 | 85113 | 6732 |
| uti_cns_0001217 | 91910 | 104464 | 12554 |
| uti_cns_0001217 | 128660 | 139280 | 10620 |
| uti_cns_0001217 | 146987 | 154732 | 7745 |
| uti_cns_0001247 | 74785 | 81244 | 6459 |
| uti_cns_0001264 | 9603 | 20086 | 10483 |
| uti_cns_0001292 | 60941 | 66756 | 5815 |
| uti_cns_0001298 | 10006 | 16402 | 6396 |
| uti_cns_0001332 | 29235 | 35291 | 6056 |
| uti_cns_0001340 | 40738 | 48367 | 7629 |
| uti_cns_0001345 | 32070 | 38705 | 6635 |
| uti_cns_0001389 | 70591 | 76398 | 5807 |
| uti_cns_0001389 | 103526 | 111167 | 7641 |
| uti_cns_0001393 | 34812 | 41594 | 6782 |
| uti_cns_0001393 | 49333 | 58022 | 8689 |
| uti_cns_0001397 | 19351 | 42555 | 23204 |
| uti_cns_0001397 | 81266 | 90898 | 9632 |
| uti_cns_0001397 | 105433 | 118891 | 13458 |
| uti_cns_0001418 | 85132 | 109967 | 24835 |
| uti_cns_0001437 | 25166 | 36707 | 11541 |
| uti_cns_0001465 | 9809 | 22208 | 12399 |
| uti_cns_0001465 | 59988 | 78267 | 18279 |
| uti_cns_0001465 | 89050 | 105356 | 16306 |
| uti_cns_0001465 | 119059 | 136379 | 17320 |
| uti_cns_0001492 | 76402 | 97679 | 21277 |
| uti_cns_0001503 | 35025 | 45289 | 10264 |
| uti_cns_0001544 | 35814 | 61913 | 26099 |
| uti_cns_0001544 | 78381 | 129537 | 51156 |
| uti_cns_0001551 | 60613 | 130267 | 69654 |
| uti_cns_0001554 | 54598 | 59957 | 5359 |
| uti_cns_0001554 | 106373 | 115996 | 9623 |
| uti_cns_0001554 | 121862 | 155561 | 33699 |
| uti_cns_0001558 | 25197 | 40468 | 15271 |
| uti_cns_0001558 | 89174 | 102524 | 13350 |
| uti_cns_0001558 | 114433 | 124774 | 10341 |
| uti_cns_0001558 | 131636 | 144099 | 12463 |
| uti_cns_0001570 | 17604 | 31939 | 14335 |
| uti_cns_0001593 | 17406 | 22916 | 5510 |
| uti_cns_0001598 | 70636 | 78352 | 7716 |
| uti_cns_0001611 | 8129 | 29993 | 21864 |
| uti_cns_0001611 | 37120 | 42501 | 5381 |
| uti_cns_0001611 | 51252 | 78357 | 27105 |
| uti_cns_0001623 | 15893 | 21266 | 5373 |
| uti_cns_0001623 | 27078 | 53201 | 26123 |
| uti_cns_0001632 | 7845 | 13013 | 5168 |
| uti_cns_0001738 | 28099 | 72473 | 44374 |
| uti_cns_0001739 | 68971 | 75439 | 6468 |
| uti_cns_0001768 | 16575 | 22273 | 5698 |
| uti_cns_0001802 | 78334 | 88029 | 9695 |
| uti_cns_0001811 | 7166 | 16293 | 9127 |
| uti_cns_0001837 | 7767 | 57057 | 49290 |
| uti_cns_0001852 | 133473 | 146021 | 12548 |
| uti_cns_0001879 | 29065 | 48255 | 19190 |
| uti_cns_0001879 | 56528 | 137083 | 80555 |
| uti_cns_0001902 | 55234 | 63509 | 8275 |
| uti_cns_0001948 | 10204 | 41557 | 31353 |
| uti_cns_0001948 | 52420 | 67677 | 15257 |
| uti_cns_0001982 | 30958 | 37350 | 6392 |
| uti_cns_0002022 | 24186 | 45469 | 21283 |
| uti_cns_0002037 | 41630 | 47321 | 5691 |
| uti_cns_0002038 | 27135 | 39671 | 12536 |
| uti_cns_0002125 | 13555 | 20334 | 6779 |
| uti_cns_0002138 | 18501 | 48339 | 29838 |
| uti_cns_0002228 | 10792 | 16435 | 5643 |
| uti_cns_0002230 | 13538 | 54732 | 41194 |
| uti_cns_0002230 | 76396 | 95623 | 19227 |
| uti_cns_0002305 | 98649 | 112167 | 13518 |
| uti_cns_0002305 | 144347 | 150792 | 6445 |
| uti_cns_0002307 | 21352 | 28001 | 6649 |
| uti_cns_0002323 | 75471 | 82154 | 6683 |
| uti_cns_0002391 | 31912 | 40550 | 8638 |
| uti_cns_0002410 | 82287 | 104456 | 22169 |
| uti_cns_0002412 | 18537 | 24645 | 6108 |
| uti_cns_0002505 | 13734 | 19359 | 5625 |
| uti_cns_0002521 | 22241 | 34823 | 12582 |
| uti_cns_0002538 | 39170 | 47277 | 8107 |
| uti_cns_0002585 | 32905 | 39677 | 6772 |
| uti_cns_0002598 | 24414 | 41542 | 17128 |
| uti_cns_0002598 | 82295 | 101543 | 19248 |
| uti_cns_0002598 | 111246 | 117040 | 5794 |
| uti_cns_0002604 | 95770 | 104445 | 8675 |
| uti_cns_0002604 | 115151 | 127650 | 12499 |
| uti_cns_0002613 | 90916 | 96098 | 5182 |
| uti_cns_0002622 | 24218 | 32882 | 8664 |
| uti_cns_0002629 | 43532 | 49301 | 5769 |
| uti_cns_0002641 | 15481 | 21296 | 5815 |
| uti_cns_0002665 | 89005 | 103370 | 14365 |
| uti_cns_0002740 | 31063 | 36756 | 5693 |
| uti_cns_0002749 | 35114 | 74377 | 39263 |
| uti_cns_0002749 | 80264 | 103449 | 23185 |
| uti_cns_0002749 | 114542 | 122746 | 8204 |
| uti_cns_0002749 | 143134 | 148897 | 5763 |
| uti_cns_0002752 | 95745 | 102510 | 6765 |
| uti_cns_0002757 | 22277 | 31934 | 9657 |
| uti_cns_0002846 | 18393 | 30977 | 12584 |
| uti_cns_0002857 | 71567 | 78296 | 6729 |
| uti_cns_0002864 | 108494 | 117994 | 9500 |
| uti_cns_0002876 | 19522 | 58053 | 38531 |
| uti_cns_0002876 | 64446 | 76079 | 11633 |
| uti_cns_0002943 | 25155 | 31942 | 6787 |
| uti_cns_0002960 | 30980 | 42353 | 11373 |
| uti_cns_0002964 | 68853 | 122742 | 53889 |
| uti_cns_0002992 | 85200 | 92575 | 7375 |
| uti_cns_0003072 | 93820 | 105330 | 11510 |
| uti_cns_0003076 | 24269 | 32825 | 8556 |
| uti_cns_0003076 | 41614 | 49224 | 7610 |
| uti_cns_0003076 | 55354 | 89923 | 34569 |
| uti_cns_0003100 | 19348 | 33811 | 14463 |
| uti_cns_0003124 | 19369 | 33878 | 14509 |
| uti_cns_0003124 | 57057 | 69446 | 12389 |
| uti_cns_0003124 | 82398 | 87883 | 5485 |
| uti_cns_0003152 | 80292 | 91865 | 11573 |
| uti_cns_0003233 | 37716 | 57084 | 19368 |
| uti_cns_0003233 | 64067 | 75452 | 11385 |
| uti_cns_0003233 | 93922 | 110078 | 16156 |
| uti_cns_0003243 | 82230 | 88985 | 6755 |
| uti_cns_0003254 | 33864 | 60942 | 27078 |
| uti_cns_0003254 | 70604 | 113170 | 42566 |
| uti_cns_0003257 | 27124 | 40593 | 13469 |
| uti_cns_0003269 | 6776 | 14380 | 7604 |
| uti_cns_0003387 | 71642 | 84111 | 12469 |
| uti_cns_0003393 | 5867 | 76231 | 70364 |
| uti_cns_0003414 | 20308 | 32326 | 12018 |
| uti_cns_0003572 | 22255 | 32872 | 10617 |
| uti_cns_0003584 | 119088 | 126589 | 7501 |
| uti_cns_0003625 | 20336 | 29736 | 9400 |
| uti_cns_0003645 | 92420 | 103498 | 11078 |
| uti_cns_0003645 | 109329 | 117941 | 8612 |
| uti_cns_0003729 | 47433 | 93790 | 46357 |
| uti_cns_0003729 | 103471 | 110271 | 6800 |
| uti_cns_0003852 | 34892 | 43437 | 8545 |
| uti_cns_0003919 | 59043 | 69577 | 10534 |
| uti_cns_0003931 | 13538 | 22271 | 8733 |
| uti_cns_0003944 | 13605 | 40531 | 26926 |
| uti_cns_0003944 | 50343 | 59018 | 8675 |
| uti_cns_0003944 | 73497 | 91587 | 18090 |
| uti_cns_0003944 | 107351 | 116053 | 8702 |
| uti_cns_0003966 | 17412 | 28055 | 10643 |
| uti_cns_0003966 | 35780 | 68489 | 32709 |
| uti_cns_0003973 | 85541 | 90926 | 5385 |
| uti_cns_0003974 | 16447 | 24884 | 8437 |
| uti_cns_0004010 | 80389 | 87032 | 6643 |
| uti_cns_0004094 | 28416 | 76379 | 47963 |
| uti_cns_0004101 | 73626 | 82130 | 8504 |
| uti_cns_0004150 | 58205 | 66723 | 8518 |
| uti_cns_0004208 | 96785 | 104186 | 7401 |
| uti_cns_0004296 | 12188 | 53640 | 41452 |
| uti_cns_0004323 | 58991 | 70618 | 11627 |
| uti_cns_0004356 | 33013 | 42578 | 9565 |
| uti_cns_0004471 | 89246 | 97371 | 8125 |
| uti_cns_0004531 | 39818 | 53214 | 13396 |
| uti_cns_0004671 | 15532 | 23241 | 7709 |
| uti_cns_0004685 | 23225 | 36208 | 12983 |
| uti_cns_0004691 | 11620 | 42400 | 30780 |
| uti_cns_0004691 | 50640 | 66713 | 16073 |
| uti_cns_0004711 | 31937 | 38712 | 6775 |
| uti_cns_0004711 | 46464 | 59785 | 13321 |
| uti_cns_0004817 | 22275 | 39667 | 17392 |
| uti_cns_0004890 | 37345 | 47896 | 10551 |
| uti_cns_0004891 | 42613 | 51131 | 8518 |
| uti_cns_0005008 | 12683 | 26142 | 13459 |
| uti_cns_0005147 | 64031 | 74402 | 10371 |
| uti_cns_0005215 | 22344 | 31924 | 9580 |
| uti_cns_0005223 | 67982 | 77101 | 9119 |
| uti_cns_0005358 | 23220 | 29027 | 5807 |
| uti_cns_0005367 | 15783 | 25097 | 9314 |
| uti_cns_0005371 | 38748 | 48231 | 9483 |
| uti_cns_0005376 | 50390 | 61608 | 11218 |
| uti_cns_0005442 | 32881 | 38633 | 5752 |
| uti_cns_0005656 | 52305 | 60820 | 8515 |
| uti_cns_0005911 | 9734 | 15483 | 5749 |
| uti_cns_0005936 | 12610 | 18221 | 5611 |
| uti_cns_0005956 | 61107 | 66725 | 5618 |
| uti_cns_0005987 | 46595 | 64611 | 18016 |
| uti_cns_0006064 | 29085 | 37640 | 8555 |
| uti_cns_0006513 | 36779 | 44447 | 7668 |
| uti_cns_0006921 | 5839 | 11637 | 5798 |
| uti_cns_0007054 | 23224 | 30775 | 7551 |
| uti_cns_0007227 | 7874 | 13552 | 5678 |
| uti_cns_0007235 | 44742 | 52247 | 7505 |
| uti_cns_0007245 | 38019 | 49288 | 11269 |
| uti_cns_0007386 | 11669 | 18228 | 6559 |
| uti_cns_0007416 | 38705 | 45475 | 6770 |
| uti_cns_0007471 | 37717 | 43465 | 5748 |
| uti_cns_0007471 | 55361 | 63526 | 8165 |
| uti_cns_0007710 | 32933 | 43546 | 10613 |
| uti_cns_0007796 | 27123 | 41582 | 14459 |
| uti_cns_0007853 | 21398 | 28741 | 7343 |
| uti_cns_0007895 | 30119 | 37158 | 7039 |
| uti_cns_0007934 | 23215 | 33584 | 10369 |
| uti_cns_0007972 | 42553 | 52220 | 9667 |
| uti_cns_0008181 | 22270 | 27905 | 5635 |
| uti_cns_0008428 | 5837 | 37504 | 31667 |
| uti_cns_0008577 | 21311 | 28046 | 6735 |
| uti_cns_0008638 | 17868 | 24052 | 6184 |
| uti_cns_0009017 | 36757 | 45457 | 8700 |
| uti_cns_0009434 | 16479 | 29987 | 13508 |
| uti_cns_0009825 | 13130 | 24095 | 10965 |
| uti_cns_0010104 | 15557 | 23946 | 8389 |
| uti_cns_0010480 | 37913 | 43339 | 5426 |
| uti_cns_0010502 | 39714 | 45431 | 5717 |
| uti_cns_0010506 | 46418 | 52903 | 6485 |
| uti_cns_0010743 | 32899 | 45280 | 12381 |
| uti_cns_0010761 | 26254 | 37692 | 11438 |
| uti_cns_0010855 | 14648 | 20280 | 5632 |
| uti_cns_0010871 | 16488 | 25955 | 9467 |
| uti_cns_0011446 | 11980 | 20292 | 8312 |
| uti_cns_0011476 | 18380 | 25058 | 6678 |
| uti_cns_0011749 | 5064 | 13480 | 8416 |
| uti_cns_0011932 | 15492 | 23226 | 7734 |
| uti_cns_0012120 | 13699 | 19235 | 5536 |
| uti_cns_0012401 | 17538 | 22998 | 5460 |
| uti_cns_0012479 | 9671 | 21880 | 12209 |
| uti_cns_0012554 | 2368 | 8580 | 6212 |
| uti_cns_0012609 | 14624 | 43406 | 28782 |
| uti_cns_0012777 | 2034 | 11596 | 9562 |
| uti_cns_0013208 | 16632 | 23223 | 6591 |
| uti_cns_0013275 | 5808 | 13567 | 7759 |
| uti_cns_0013750 | 14544 | 20945 | 6401 |
| uti_cns_0013827 | 43622 | 51840 | 8218 |
| uti_cns_0013846 | 13093 | 22246 | 9153 |
| uti_cns_0014133 | 6434 | 11607 | 5173 |
| uti_cns_0014782 | 13547 | 20322 | 6775 |
| uti_cns_0014889 | 7771 | 15392 | 7621 |
| uti_cns_0015551 | 3033 | 8708 | 5675 |
| uti_cns_0045366 | 103491 | 110133 | 6642 |
| uti_cns_0045366 | 125717 | 135995 | 10278 |
| uti_cns_0045367 | 238859 | 245623 | 6764 |
| uti_cns_0045374 | 21305 | 27987 | 6682 |
| uti_cns_0045387 | 60929 | 85123 | 24194 |
| uti_cns_0045388 | 93875 | 117015 | 23140 |
| uti_cns_0045388 | 170335 | 187535 | 17200 |
| uti_cns_0045388 | 193611 | 211761 | 18150 |
| uti_cns_0045388 | 217575 | 227238 | 9663 |
| uti_cns_0045388 | 243222 | 258988 | 15766 |
| uti_cns_0045388 | 297849 | 307339 | 9490 |
| uti_cns_0045390 | 14785 | 29022 | 14237 |
| uti_cns_0045390 | 61889 | 122696 | 60807 |
| uti_cns_0045390 | 260127 | 277543 | 17416 |
| uti_cns_0045390 | 283334 | 291093 | 7759 |
| uti_cns_0045390 | 305607 | 313316 | 7709 |
| uti_cns_0045403 | 36834 | 42515 | 5681 |
| uti_cns_0045431 | 360714 | 370046 | 9332 |
| uti_cns_0045480 | 19353 | 29970 | 10617 |
| uti_cns_0045485 | 271906 | 281340 | 9434 |
| uti_cns_0045486 | 114109 | 135402 | 21293 |
| uti_cns_0045489 | 130594 | 140241 | 9647 |
| uti_cns_0045494 | 146034 | 207769 | 61735 |
| uti_cns_0045611 | 288232 | 293824 | 5592 |
| uti_cns_0045637 | 12637 | 54880 | 42243 |
| uti_cns_0045638 | 21527 | 29967 | 8440 |
| uti_cns_0045654 | 42615 | 52047 | 9432 |
| uti_cns_0045672 | 25295 | 39679 | 14384 |
| uti_cns_0045714 | 47689 | 88982 | 41293 |
| uti_cns_0045714 | 101611 | 118697 | 17086 |
| uti_cns_0045747 | 94784 | 100422 | 5638 |
| uti_cns_0045800 | 125183 | 131279 | 6096 |
| uti_cns_0045843 | 40665 | 53023 | 12358 |
| uti_cns_0045849 | 120876 | 173923 | 53047 |
| uti_cns_0045855 | 270821 | 278431 | 7610 |
| uti_cns_0045977 | 62867 | 68682 | 5815 |
| uti_cns_0045977 | 77364 | 86307 | 8943 |
| uti_cns_0045977 | 109885 | 118973 | 9088 |
| uti_cns_0045981 | 150991 | 156508 | 5517 |
| uti_cns_0045981 | 162494 | 173004 | 10510 |
| uti_cns_0045983 | 71570 | 95350 | 23780 |
| uti_cns_0045983 | 107406 | 114138 | 6732 |
| uti_cns_0045983 | 126678 | 139064 | 12386 |
| uti_cns_0045997 | 133544 | 147754 | 14210 |
| uti_cns_0045997 | 174060 | 207864 | 33804 |
| uti_cns_0046010 | 226471 | 233999 | 7528 |
| uti_cns_0046030 | 101593 | 127644 | 26051 |
| uti_cns_0046030 | 157623 | 163439 | 5816 |
| uti_cns_0046031 | 18480 | 82768 | 64288 |
| uti_cns_0046031 | 91914 | 102286 | 10372 |
| uti_cns_0046031 | 109687 | 118959 | 9272 |
| uti_cns_0046031 | 128472 | 134407 | 5935 |
| uti_cns_0046087 | 86069 | 98646 | 12577 |
| uti_cns_0046118 | 67386 | 78322 | 10936 |
| uti_cns_0046130 | 142149 | 200202 | 58053 |
| uti_cns_0046134 | 24196 | 33850 | 9654 |
| uti_cns_0046145 | 72546 | 79305 | 6759 |
| uti_cns_0046185 | 18477 | 27109 | 8632 |
| uti_cns_0046185 | 63005 | 91795 | 28790 |
| uti_cns_0046236 | 76450 | 87892 | 11442 |
| uti_cns_0046251 | 74500 | 84020 | 9520 |
| uti_cns_0046258 | 97683 | 104394 | 6711 |
| uti_cns_0046287 | 75474 | 82136 | 6662 |
| uti_cns_0046368 | 83166 | 90845 | 7679 |
| uti_cns_0046369 | 22285 | 32900 | 10615 |
| uti_cns_0046481 | 59132 | 72495 | 13363 |
| uti_cns_0046520 | 65862 | 71453 | 5591 |
| uti_cns_0046572 | 17451 | 56115 | 38664 |
| uti_cns_0046572 | 62868 | 75447 | 12579 |
| uti_cns_0046643 | 65837 | 72414 | 6577 |
| uti_cns_0046650 | 99905 | 106376 | 6471 |
| uti_cns_0046814 | 21325 | 43530 | 22205 |
| uti_cns_0046814 | 51285 | 60922 | 9637 |
| uti_cns_0046835 | 64878 | 72541 | 7663 |
| uti_cns_0047112 | 61164 | 74442 | 13278 |
| uti_cns_0047128 | 29083 | 38682 | 9599 |
| uti_cns_0047510 | 42067 | 53100 | 11033 |
| uti_cns_0047575 | 19343 | 25148 | 5805 |
| uti_cns_0047761 | 40617 | 60867 | 20250 |
| uti_cns_0047778 | 18387 | 31937 | 13550 |
| uti_cns_0047809 | 9670 | 14742 | 5072 |
| uti_cns_0047810 | 37929 | 60952 | 23023 |
| uti_cns_0047867 | 28349 | 38699 | 10350 |
| uti_cns_0047952 | 61997 | 67465 | 5468 |
| uti_cns_0047986 | 69628 | 75885 | 6257 |
| uti_cns_0048012 | 967 | 11635 | 10668 |
| uti_cns_0048155 | 44716 | 60568 | 15852 |
| uti_cns_0048232 | 4973 | 12479 | 7506 |
| uti_cns_0048261 | 16489 | 37687 | 21198 |
| uti_cns_0048529 | 36079 | 42562 | 6483 |

**Supplementary Table 5. List of piRNA-producing transcripts from the *de novo* transcriptome and their piRNA levels**

| Transcript ID | Length | PPM | RPKM |
| --- | --- | --- | --- |
| c11_g1_i1 | 251 | 26.555 | 105.797 |
| c44_g1_i1 | 204 | 38.1728 | 187.122 |
| c67_g1_i1 | 219 | 179.246 | 818.476 |
| c74_g1_i2 | 455 | 115.348 | 253.513 |
| c90_g1_i1 | 565 | 161.82 | 286.406 |
| c101_g1_i1 | 303 | 45.6414 | 150.632 |
| c155_g1_i1 | 510 | 148.542 | 291.259 |
| c187_g1_i1 | 623 | 89.6231 | 143.857 |
| c187_g2_i1 | 241 | 25.7252 | 106.743 |
| c200_g1_i1 | 267 | 63.898 | 239.318 |
| c205_g1_i1 | 346 | 68.0472 | 196.668 |
| c257_g1_i1 | 395 | 44.8116 | 113.447 |
| c317_g1_i1 | 209 | 63.898 | 305.732 |
| c365_g1_i1 | 218 | 118.668 | 544.347 |
| c403_g1_i1 | 223 | 41.4922 | 186.064 |
| c529_g1_i1 | 245 | 94.6022 | 386.131 |
| c557_g1_i1 | 213 | 82.1545 | 385.702 |
| c765_g1_i1 | 205 | 29.0445 | 141.681 |
| c982_g1_i1 | 287 | 94.6022 | 329.624 |
| c1014_g1_i1 | 215 | 34.0236 | 158.249 |
| c1077_g1_i1 | 220 | 43.1519 | 196.145 |
| c1294_g1_i1 | 296 | 54.7697 | 185.033 |
| c1307_g1_i1 | 214 | 33.1938 | 155.111 |
| c1345_g1_i1 | 317 | 66.3875 | 209.424 |
| c1345_g2_i1 | 246 | 98.7514 | 401.429 |
| c1603_g1_i1 | 213 | 23.2356 | 109.087 |
| c1642_g1_i1 | 219 | 40.6623 | 185.673 |
| c1768_g1_i1 | 401 | 108.71 | 271.096 |
| c1832_g1_i1 | 245 | 76.3456 | 311.615 |
| c1853_g1_i1 | 318 | 80.4949 | 253.128 |
| c1885_g1_i1 | 334 | 40.6623 | 121.744 |
| c1931_g1_i1 | 380 | 84.6441 | 222.748 |
| c2050_g1_i1 | 225 | 43.1519 | 191.786 |
| c2089_g1_i1 | 261 | 59.7488 | 228.922 |
| c2167_g1_i1 | 205 | 55.5995 | 271.217 |
| c2300_g1_i1 | 331 | 141.903 | 428.711 |
| c2321_g1_i1 | 267 | 76.3456 | 285.939 |
| c2346_g1_i1 | 347 | 44.8116 | 129.14 |
| c2387_g1_i2 | 310 | 39.0027 | 125.815 |
| c2390_g1_i1 | 283 | 53.9398 | 190.6 |
| c2392_g1_i1 | 235 | 77.1755 | 328.406 |
| c2750_g1_i1 | 222 | 82.9844 | 373.804 |
| c2790_g1_i1 | 285 | 753.498 | 2643.85 |
| c2855_g1_i1 | 248 | 34.8534 | 140.538 |
| c3031_g1_i1 | 682 | 670.514 | 983.158 |
| c3108_g1_i1 | 242 | 92.9425 | 384.06 |
| c3133_g1_i1 | 372 | 120.327 | 323.461 |
| c3290_g1_i1 | 314 | 48.9608 | 155.926 |
| c3441_g1_i1 | 537 | 64.7278 | 120.536 |
| c3510_g1_i1 | 258 | 43.1519 | 167.255 |
| c3966_g1_i1 | 366 | 96.2619 | 263.011 |
| c3989_g1_i1 | 229 | 103.73 | 452.972 |
| c4239_g1_i1 | 615 | 209.121 | 340.034 |
| c4289_g1_i1 | 298 | 53.11 | 178.221 |
| c4357_g1_i1 | 215 | 31.5341 | 146.67 |
| c4420_g1_i1 | 242 | 75.5158 | 312.049 |
| c4443_g1_i1 | 201 | 41.4922 | 206.429 |
| c4481_g1_i1 | 294 | 82.1545 | 279.437 |
| c4590_g1_i1 | 429 | 58.9189 | 137.34 |
| c4692_g1_i1 | 264 | 110.369 | 418.065 |
| c4716_g1_i1 | 267 | 171.778 | 643.362 |
| c4800_g1_i1 | 946 | 125.306 | 132.459 |
| c5064_g1_i1 | 248 | 27.3848 | 110.423 |
| c5085_g1_i1 | 342 | 133.605 | 390.657 |
| c5386_g1_i1 | 364 | 58.9189 | 161.865 |
| c5392_g2_i1 | 251 | 58.9189 | 234.737 |
| c5550_g1_i1 | 673 | 85.4739 | 127.004 |
| c5596_g1_i1 | 429 | 181.736 | 423.627 |
| c5888_g1_i1 | 229 | 172.608 | 753.745 |
| c5969_g1_i1 | 336 | 73.8561 | 219.81 |
| c6021_g1_i1 | 316 | 49.7906 | 157.565 |
| c6091_g1_i1 | 296 | 33.1938 | 112.141 |
| c6098_g1_i1 | 236 | 29.8744 | 126.586 |
| c6154_g1_i1 | 208 | 24.0655 | 115.699 |
| c6191_g1_i1 | 257 | 47.3011 | 184.051 |
| c6343_g1_i1 | 243 | 24.8953 | 102.45 |
| c6345_g1_i1 | 341 | 117.838 | 345.565 |
| c6387_g1_i1 | 213 | 108.71 | 510.373 |
| c6441_g1_i1 | 237 | 44.8116 | 189.078 |
| c6465_g1_i1 | 224 | 139.414 | 622.383 |
| c6619_g1_i1 | 307 | 73.8561 | 240.574 |
| c6682_g1_i1 | 242 | 49.7906 | 205.746 |
| c6723_g1_i1 | 277 | 34.8534 | 125.825 |
| c6736_g1_i1 | 311 | 377.579 | 1214.08 |
| c6745_g1_i1 | 225 | 23.2356 | 103.269 |
| c6789_g1_i1 | 229 | 95.432 | 416.734 |
| c6891_g1_i1 | 346 | 90.453 | 261.425 |
| c6945_g1_i1 | 206 | 27.3848 | 132.936 |
| c6979_g1_i1 | 283 | 31.5341 | 111.428 |
| c6988_g1_i1 | 573 | 82.9844 | 144.824 |
| c7020_g1_i1 | 405 | 156.84 | 387.26 |
| c7079_g1_i1 | 225 | 61.4084 | 272.926 |
| c7091_g1_i1 | 245 | 102.071 | 416.615 |
| c7161_g1_i1 | 281 | 89.6231 | 318.944 |
| c7180_g1_i1 | 269 | 37.343 | 138.821 |
| c7288_g1_i1 | 344 | 46.4713 | 135.091 |
| c7316_g1_i1 | 204 | 28.2147 | 138.307 |
| c7323_g1_i1 | 280 | 42.322 | 151.15 |
| c7448_g1_i1 | 260 | 181.736 | 698.984 |
| c7457_g1_i1 | 311 | 66.3875 | 213.465 |
| c7617_g1_i1 | 399 | 61.4084 | 153.906 |
| c7623_g1_i1 | 240 | 92.1127 | 383.803 |
| c7642_g1_i1 | 225 | 73.8561 | 328.249 |
| c7796_g1_i1 | 396 | 40.6623 | 102.683 |
| c7816_g1_i1 | 260 | 131.115 | 504.29 |
| c7836_g1_i1 | 228 | 41.4922 | 181.983 |
| c7861_g1_i1 | 346 | 196.673 | 568.419 |
| c7881_g1_i1 | 227 | 56.4294 | 248.588 |
| c7940_g1_i1 | 203 | 43.1519 | 212.571 |
| c7956_g1_i1 | 272 | 36.5131 | 134.239 |
| c7959_g1_i1 | 550 | 440.647 | 801.177 |
| c7981_g1_i1 | 215 | 44.8116 | 208.426 |
| c7981_g2_i1 | 309 | 78.8352 | 255.13 |
| c8033_g1_i1 | 294 | 92.9425 | 316.131 |
| c8081_g1_i1 | 225 | 407.453 | 1810.9 |
| c8103_g1_i1 | 272 | 54.7697 | 201.359 |
| c8162_g1_i1 | 298 | 39.8325 | 133.666 |
| c8300_g1_i1 | 305 | 39.8325 | 130.598 |
| c8360_g1_i1 | 239 | 43.9817 | 184.024 |
| c8363_g1_i1 | 257 | 37.343 | 145.303 |
| c8488_g1_i1 | 340 | 37.343 | 109.832 |
| c8515_g1_i1 | 461 | 48.1309 | 104.406 |
| c8559_g1_i1 | 580 | 712.006 | 1227.6 |
| c8629_g1_i1 | 216 | 38.1728 | 176.726 |
| c8629_g2_i1 | 345 | 77.1755 | 223.697 |
| c8688_g1_i1 | 211 | 22.4058 | 106.189 |
| c8708_g1_i1 | 1079 | 437.328 | 405.308 |
| c8714_g1_i1 | 211 | 77.1755 | 365.761 |
| c8719_g1_i1 | 239 | 34.0236 | 142.358 |
| c8772_g1_i1 | 558 | 59.7488 | 107.077 |
| c8807_g1_i1 | 225 | 270.529 | 1202.35 |
| c8859_g1_i1 | 288 | 52.2802 | 181.528 |
| c8995_g1_i1 | 319 | 251.443 | 788.222 |
| c9076_g1_i1 | 227 | 61.4084 | 270.522 |
| c9183_g1_i1 | 319 | 64.7278 | 202.909 |
| c9226_g1_i1 | 308 | 79.665 | 258.653 |
| c9243_g1_i1 | 208 | 111.199 | 534.611 |
| c9280_g1_i1 | 303 | 34.8534 | 115.028 |
| c9387_g1_i1 | 201 | 36.5131 | 181.657 |
| c9398_g1_i1 | 211 | 50.6205 | 239.907 |
| c9573_g1_i1 | 221 | 61.4084 | 277.866 |
| c9687_g1_i1 | 560 | 71.3666 | 127.44 |
| c9702_g1_i1 | 484 | 79.665 | 164.597 |
| c9777_g1_i1 | 209 | 48.1309 | 230.292 |
| c10043_g1_i1 | 252 | 332.767 | 1320.51 |
| c10121_g1_i1 | 208 | 54.7697 | 263.316 |
| c10197_g1_i1 | 219 | 111.199 | 507.758 |
| c10252_g1_i1 | 298 | 63.0681 | 211.638 |
| c10269_g1_i1 | 216 | 34.8534 | 161.359 |
| c10310_g1_i1 | 259 | 37.343 | 144.181 |
| c10432_g1_i1 | 283 | 61.4084 | 216.991 |
| c10532_g1_i1 | 407 | 42.322 | 103.985 |
| c10638_g1_i1 | 207 | 48.9608 | 236.526 |
| c10799_g1_i1 | 377 | 90.453 | 239.928 |
| c10846_g1_i1 | 281 | 48.9608 | 174.238 |
| c10914_g1_i1 | 201 | 344.385 | 1713.36 |
| c10924_g2_i1 | 352 | 80.4949 | 228.679 |
| c11108_g1_i1 | 272 | 100.411 | 369.158 |
| c11174_g1_i1 | 218 | 33.1938 | 152.265 |
| c11293_g1_i1 | 391 | 131.115 | 335.333 |
| c11293_g2_i1 | 790 | 141.073 | 178.574 |
| c11413_g2_i1 | 280 | 46.4713 | 165.969 |
| c11442_g1_i2 | 319 | 34.8534 | 109.258 |
| c11450_g1_i1 | 270 | 44.8116 | 165.969 |
| c11453_g1_i1 | 240 | 44.8116 | 186.715 |
| c11496_g1_i1 | 261 | 60.5786 | 232.102 |
| c11585_g1_i1 | 386 | 79.665 | 206.386 |
| c11676_g1_i1 | 248 | 77.1755 | 311.191 |
| c11716_g1_i1 | 311 | 50.6205 | 162.767 |
| c11718_g1_i1 | 341 | 113.689 | 333.398 |
| c11757_g1_i1 | 467 | 66.3875 | 142.157 |
| c11877_g1_i2 | 364 | 138.584 | 380.725 |
| c11918_g1_i1 | 298 | 164.309 | 551.373 |
| c11944_g1_i1 | 223 | 61.4084 | 275.374 |
| c11960_g1_i1 | 243 | 39.8325 | 163.92 |
| c12028_g1_i1 | 204 | 25.7252 | 126.104 |
| c12049_g1_i1 | 225 | 79.665 | 354.067 |
| c12070_g1_i1 | 242 | 61.4084 | 253.754 |
| c12086_g1_i1 | 643 | 196.673 | 305.868 |
| c12086_g2_i1 | 236 | 121.987 | 516.894 |
| c12177_g1_i1 | 215 | 27.3848 | 127.371 |
| c12260_g1_i1 | 490 | 49.7906 | 101.614 |
| c12263_g1_i1 | 281 | 74.6859 | 265.786 |
| c12301_g1_i1 | 316 | 52.2802 | 165.444 |
| c12322_g1_i1 | 265 | 26.555 | 100.208 |
| c12340_g1_i1 | 454 | 273.019 | 601.363 |
| c12389_g1_i1 | 216 | 41.4922 | 192.093 |
| c12452_g1_i1 | 345 | 107.05 | 310.289 |
| c12533_g1_i1 | 315 | 43.1519 | 136.99 |
| c12616_g1_i1 | 309 | 34.8534 | 112.794 |
| c12759_g1_i1 | 409 | 60.5786 | 148.114 |
| c12786_g1_i1 | 272 | 33.1938 | 122.036 |
| c12878_g1_i1 | 307 | 60.5786 | 197.324 |
| c12907_g1_i1 | 320 | 97.9216 | 306.005 |
| c12975_g1_i1 | 394 | 40.6623 | 103.204 |
| c13023_g1_i1 | 240 | 138.584 | 577.433 |
| c13066_g1_i1 | 321 | 105.39 | 328.318 |
| c13119_g1_i1 | 206 | 112.029 | 543.83 |
| c13122_g1_i1 | 201 | 22.4058 | 111.472 |
| c13136_g1_i1 | 234 | 48.9608 | 209.234 |
| c13221_g1_i1 | 380 | 60.5786 | 159.417 |
| c13351_g1_i1 | 293 | 41.4922 | 141.612 |
| c13464_g1_i1 | 328 | 62.2383 | 189.751 |
| c13521_g1_i1 | 329 | 117.008 | 355.647 |
| c13574_g1_i1 | 315 | 35.6833 | 113.28 |
| c13695_g1_i1 | 304 | 323.639 | 1064.6 |
| c13709_g1_i1 | 239 | 68.0472 | 284.716 |
| c13773_g1_i1 | 324 | 76.3456 | 235.635 |
| c13778_g2_i1 | 388 | 58.9189 | 151.853 |
| c13781_g1_i1 | 311 | 36.5131 | 117.406 |
| c13892_g1_i1 | 279 | 34.8534 | 124.923 |
| c13934_g1_i1 | 426 | 78.8352 | 185.059 |
| c13966_g1_i1 | 230 | 110.369 | 479.866 |
| c13973_g1_i1 | 270 | 82.9844 | 307.35 |
| c14046_g1_i1 | 201 | 74.6859 | 371.572 |
| c14155_g1_i1 | 227 | 30.7042 | 135.261 |
| c14166_g1_i1 | 414 | 43.1519 | 104.232 |
| c14296_g1_i1 | 307 | 47.3011 | 154.075 |
| c14296_g2_i1 | 329 | 69.7069 | 211.875 |
| c14496_g1_i1 | 519 | 653.917 | 1259.96 |
| c14506_g1_i1 | 243 | 24.8953 | 102.45 |
| c14539_g1_i1 | 249 | 44.8116 | 179.966 |
| c14621_g1_i1 | 259 | 41.4922 | 160.202 |
| c14661_g1_i1 | 471 | 110.369 | 234.33 |
| c14682_g1_i1 | 283 | 34.0236 | 120.225 |
| c14724_g1_i1 | 467 | 64.7278 | 138.603 |
| c14733_g1_i1 | 1106 | 330.278 | 298.624 |
| c14740_g1_i1 | 536 | 117.008 | 218.298 |
| c14851_g1_i1 | 367 | 145.223 | 395.702 |
| c14894_g1_i1 | 243 | 62.2383 | 256.125 |
| c14897_g1_i1 | 326 | 79.665 | 244.371 |
| c14995_g1_i1 | 349 | 48.1309 | 137.911 |
| c15102_g1_i1 | 237 | 26.555 | 112.046 |
| c15122_g1_i1 | 552 | 703.708 | 1274.83 |
| c15147_g1_i1 | 328 | 119.498 | 364.322 |
| c15198_g1_i1 | 684 | 271.359 | 396.724 |
| c15236_g1_i1 | 401 | 82.1545 | 204.874 |
| c15362_g1_i1 | 252 | 39.0027 | 154.772 |
| c15458_g1_i1 | 235 | 80.4949 | 342.531 |
| c15607_g1_i1 | 231 | 94.6022 | 409.533 |
| c15610_g1_i1 | 206 | 41.4922 | 201.418 |
| c15614_g1_i1 | 382 | 180.906 | 473.576 |
| c15643_g1_i1 | 227 | 28.2147 | 124.294 |
| c15840_g1_i1 | 215 | 24.8953 | 115.792 |
| c15885_g1_i1 | 258 | 26.555 | 102.926 |
| c15887_g1_i1 | 201 | 89.6231 | 445.886 |
| c15970_g1_i1 | 357 | 560.145 | 1569.03 |
| c15988_g1_i1 | 505 | 126.966 | 251.418 |
| c16060_g1_i1 | 252 | 42.322 | 167.945 |
| c16116_g1_i1 | 212 | 34.8534 | 164.403 |
| c16245_g1_i1 | 208 | 120.327 | 578.497 |
| c16322_g1_i1 | 232 | 35.6833 | 153.807 |
| c16667_g1_i1 | 220 | 24.0655 | 109.389 |
| c16684_g1_i1 | 213 | 33.1938 | 155.839 |
| c16767_g1_i1 | 265 | 31.5341 | 118.996 |
| c16911_g1_i1 | 207 | 59.7488 | 288.641 |
| c16924_g1_i1 | 277 | 67.2174 | 242.662 |
| c17083_g1_i1 | 224 | 29.0445 | 129.663 |
| c17237_g1_i1 | 725 | 87.9634 | 121.329 |
| c17302_g1_i1 | 206 | 32.3639 | 157.106 |
| c17393_g1_i1 | 344 | 41.4922 | 120.617 |
| c17399_g1_i1 | 213 | 114.518 | 537.645 |
| c17467_g1_i1 | 277 | 33.1938 | 119.833 |
| c17479_g1_i1 | 241 | 25.7252 | 106.743 |
| c17484_g1_i1 | 216 | 27.3848 | 126.782 |
| c17553_g1_i1 | 210 | 29.0445 | 138.307 |
| c17562_g1_i1 | 247 | 125.306 | 507.313 |
| c17874_g1_i1 | 285 | 65.5577 | 230.027 |
| c17939_g1_i1 | 236 | 55.5995 | 235.591 |
| c17956_g1_i1 | 308 | 73.8561 | 239.793 |
| c18154_g1_i1 | 542 | 89.6231 | 165.356 |
| c18261_g1_i2 | 455 | 227.377 | 499.73 |
| c18273_g1_i1 | 250 | 62.2383 | 248.953 |
| c18392_g1_i1 | 219 | 49.7906 | 227.354 |
| c18394_g1_i1 | 217 | 189.204 | 871.91 |
| c18408_g1_i1 | 270 | 53.9398 | 199.777 |
| c18425_g1_i1 | 378 | 141.073 | 373.21 |
| c18472_g1_i1 | 230 | 34.0236 | 147.929 |
| c18491_g1_i1 | 250 | 28.2147 | 112.859 |
| c18580_g1_i1 | 221 | 823.205 | 3724.91 |
| c18598_g1_i1 | 269 | 66.3875 | 246.794 |
| c18846_g1_i1 | 224 | 23.2356 | 103.73 |
| c18853_g1_i1 | 752 | 492.097 | 654.385 |
| c19016_g1_i1 | 332 | 118.668 | 357.433 |
| c19120_g1_i1 | 210 | 29.0445 | 138.307 |
| c19168_g1_i1 | 285 | 37.343 | 131.028 |
| c19178_g1_i1 | 313 | 101.241 | 323.454 |
| c19200_g1_i1 | 225 | 25.7252 | 114.334 |
| c19270_g1_i1 | 278 | 107.88 | 388.056 |
| c19356_g1_i1 | 228 | 45.6414 | 200.182 |
| c19408_g1_i1 | 248 | 38.1728 | 153.923 |
| c19408_g2_i1 | 451 | 78.0053 | 172.961 |
| c19421_g1_i1 | 217 | 45.6414 | 210.329 |
| c19577_g1_i1 | 347 | 40.6623 | 117.183 |
| c19636_g1_i1 | 223 | 185.055 | 829.844 |
| c19729_g1_i1 | 377 | 39.8325 | 105.657 |
| c19779_g1_i1 | 330 | 58.0891 | 176.027 |
| c19804_g1_i1 | 630 | 307.042 | 487.369 |
| c19843_g1_i1 | 201 | 92.1127 | 458.272 |
| c19883_g1_i1 | 522 | 52.2802 | 100.154 |
| c20018_g1_i1 | 214 | 26.555 | 124.089 |
| c20112_g1_i1 | 201 | 42.322 | 210.557 |
| c20181_g1_i1 | 555 | 78.8352 | 142.045 |
| c20200_g1_i1 | 386 | 91.2828 | 236.484 |
| c20201_g1_i1 | 305 | 42.322 | 138.761 |
| c20234_g1_i1 | 263 | 44.8116 | 170.386 |
| c20412_g2_i1 | 514 | 137.754 | 268.004 |
| c20415_g1_i1 | 206 | 48.9608 | 237.674 |
| c20438_g1_i1 | 238 | 40.6623 | 170.85 |
| c20483_g1_i1 | 307 | 152.691 | 497.366 |
| c20483_g1_i2 | 226 | 40.6623 | 179.922 |
| c20491_g1_i1 | 233 | 74.6859 | 320.541 |
| c20510_g1_i1 | 478 | 94.6022 | 197.913 |
| c20535_g2_i1 | 227 | 34.0236 | 149.884 |
| c20578_g1_i1 | 473 | 77.1755 | 163.162 |
| c20611_g1_i1 | 665 | 259.741 | 390.588 |
| c20652_g1_i1 | 404 | 86.3038 | 213.623 |
| c20659_g1_i1 | 203 | 37.343 | 183.956 |
| c20669_g1_i1 | 216 | 48.9608 | 226.67 |
| c20699_g1_i1 | 239 | 26.555 | 111.109 |
| c20794_g1_i1 | 542 | 203.312 | 375.114 |
| c20917_g1_i1 | 616 | 167.628 | 272.124 |
| c20922_g1_i1 | 203 | 39.8325 | 196.219 |
| c21044_g1_i1 | 302 | 62.2383 | 206.087 |
| c21155_g1_i1 | 201 | 46.4713 | 231.2 |
| c21167_g1_i1 | 422 | 95.432 | 226.142 |
| c21297_g1_i1 | 318 | 79.665 | 250.519 |
| c21322_g1_i1 | 247 | 37.343 | 151.186 |
| c21598_g1_i1 | 231 | 35.6833 | 154.473 |
| c21699_g1_i1 | 270 | 32.3639 | 119.866 |
| c21762_g1_i1 | 277 | 34.0236 | 122.829 |
| c22047_g1_i1 | 502 | 82.9844 | 165.308 |
| c22073_g1_i1 | 337 | 34.0236 | 100.96 |
| c22157_g1_i1 | 217 | 53.11 | 244.747 |
| c22238_g1_i1 | 388 | 103.73 | 267.347 |
| c22276_g1_i1 | 256 | 26.555 | 103.73 |
| c22296_g1_i1 | 284 | 181.736 | 639.915 |
| c22327_g1_i1 | 204 | 24.8953 | 122.036 |
| c22405_g1_i1 | 219 | 69.7069 | 318.296 |
| c22433_g1_i1 | 228 | 34.0236 | 149.226 |
| c22489_g1_i1 | 299 | 133.605 | 446.839 |
| c22612_g1_i1 | 251 | 85.4739 | 340.534 |
| c22646_g1_i1 | 212 | 30.7042 | 144.831 |
| c22711_g1_i1 | 207 | 34.0236 | 164.365 |
| c22715_g1_i1 | 233 | 46.4713 | 199.447 |
| c22921_g1_i1 | 212 | 53.11 | 250.519 |
| c22941_g2_i1 | 344 | 65.5577 | 190.575 |
| c22943_g1_i1 | 240 | 31.5341 | 131.392 |
| c23034_g1_i1 | 331 | 56.4294 | 170.482 |
| c23054_g1_i1 | 267 | 46.4713 | 174.05 |
| c23071_g1_i1 | 237 | 282.977 | 1193.99 |
| c23292_g1_i1 | 371 | 92.9425 | 250.519 |
| c23328_g1_i1 | 312 | 43.1519 | 138.307 |
| c23366_g1_i1 | 268 | 34.0236 | 126.954 |
| c23456_g1_i1 | 347 | 117.838 | 339.59 |
| c23497_g1_i1 | 232 | 29.0445 | 125.192 |
| c23512_g1_i1 | 239 | 43.1519 | 180.552 |
| c23522_g1_i1 | 610 | 493.757 | 809.438 |
| c23530_g1_i1 | 213 | 68.877 | 323.366 |
| c23572_g1_i1 | 216 | 32.3639 | 149.833 |
| c23741_g1_i1 | 423 | 101.241 | 239.34 |
| c23807_g1_i1 | 233 | 26.555 | 113.97 |
| c23850_g1_i1 | 334 | 35.6833 | 106.836 |
| c23874_g1_i1 | 302 | 34.0236 | 112.661 |
| c23900_g1_i1 | 230 | 187.545 | 815.412 |
| c24040_g1_i1 | 228 | 80.4949 | 353.048 |
| c24052_g1_i1 | 314 | 41.4922 | 132.141 |
| c24202_g1_i1 | 230 | 30.7042 | 133.497 |
| c24329_g1_i1 | 377 | 50.6205 | 134.272 |
| c24335_g1_i1 | 255 | 44.8116 | 175.732 |
| c24366_g1_i1 | 337 | 52.2802 | 155.134 |
| c24424_g1_i1 | 547 | 279.657 | 511.257 |
| c24497_g1_i1 | 282 | 126.966 | 450.234 |
| c24548_g1_i1 | 327 | 48.1309 | 147.189 |
| c24606_g1_i1 | 356 | 39.8325 | 111.889 |
| c24625_g1_i1 | 320 | 48.1309 | 150.409 |
| c24696_g1_i1 | 245 | 34.8534 | 142.259 |
| c24723_g1_i1 | 226 | 144.393 | 638.906 |
| c24748_g1_i1 | 418 | 161.82 | 387.128 |
| c24751_g1_i1 | 220 | 29.0445 | 132.021 |
| c24812_g1_i1 | 469 | 103.73 | 221.174 |
| c24914_g1_i1 | 261 | 118.668 | 454.665 |
| c25062_g1_i1 | 223 | 149.372 | 669.829 |
| c25064_g1_i1 | 261 | 34.0236 | 130.359 |
| c25073_g1_i1 | 441 | 84.6441 | 191.937 |
| c25073_g2_i1 | 229 | 45.6414 | 199.307 |
| c25132_g1_i1 | 244 | 51.4503 | 210.862 |
| c25168_g1_i1 | 301 | 39.8325 | 132.334 |
| c25204_g1_i1 | 241 | 32.3639 | 134.29 |
| c25218_g1_i1 | 307 | 39.0027 | 127.044 |
| c25340_g1_i1 | 240 | 36.5131 | 152.138 |
| c25392_g1_i1 | 341 | 50.6205 | 148.447 |
| c25521_g1_i1 | 376 | 68.877 | 183.184 |
| c25524_g1_i1 | 203 | 34.0236 | 167.604 |
| c25580_g1_i1 | 201 | 26.555 | 132.114 |
| c25600_g1_i1 | 298 | 165.139 | 554.157 |
| c25633_g1_i1 | 248 | 56.4294 | 227.538 |
| c25747_g1_i1 | 268 | 82.1545 | 306.547 |
| c25792_g1_i1 | 668 | 139.414 | 208.703 |
| c25966_g1_i1 | 212 | 39.8325 | 187.889 |
| c25970_g1_i1 | 300 | 65.5577 | 218.526 |
| c26001_g1_i1 | 260 | 28.2147 | 108.518 |
| c26010_g1_i1 | 211 | 62.2383 | 294.968 |
| c26025_g1_i1 | 466 | 108.71 | 233.282 |
| c26047_g1_i1 | 343 | 422.391 | 1231.46 |
| c26048_g1_i1 | 221 | 33.1938 | 150.198 |
| c26107_g2_i1 | 310 | 55.5995 | 179.353 |
| c26134_g1_i1 | 381 | 45.6414 | 119.794 |
| c26175_g1_i1 | 372 | 53.9398 | 145 |
| c26255_g1_i1 | 246 | 39.0027 | 158.547 |
| c26261_g1_i1 | 216 | 40.6623 | 188.252 |
| c26291_g1_i1 | 212 | 49.7906 | 234.861 |
| c26312_g1_i1 | 255 | 48.1309 | 188.749 |
| c26329_g1_i1 | 392 | 149.372 | 381.051 |
| c26337_g1_i1 | 202 | 300.403 | 1487.15 |
| c26357_g1_i1 | 232 | 93.7724 | 404.191 |
| c26422_g1_i1 | 317 | 69.7069 | 219.896 |
| c26432_g1_i1 | 294 | 167.628 | 570.165 |
| c26514_g1_i1 | 320 | 144.393 | 451.228 |
| c26531_g1_i1 | 282 | 49.7906 | 176.563 |
| c26629_g1_i1 | 610 | 119.498 | 195.898 |
| c26629_g2_i1 | 250 | 127.796 | 511.184 |
| c26636_g1_i1 | 264 | 273.848 | 1037.3 |
| c26650_g1_i1 | 351 | 40.6623 | 115.847 |
| c26734_g1_i1 | 367 | 105.39 | 287.167 |
| c26753_g1_i1 | 262 | 35.6833 | 136.196 |
| c26759_g1_i1 | 402 | 68.0472 | 169.272 |
| c26765_g1_i1 | 253 | 58.0891 | 229.601 |
| c26790_g1_i1 | 307 | 31.5341 | 102.717 |
| c26814_g1_i1 | 549 | 55.5995 | 101.274 |
| c26897_g1_i1 | 206 | 29.8744 | 145.021 |
| c26912_g2_i1 | 508 | 89.6231 | 176.423 |
| c26940_g1_i1 | 368 | 126.966 | 345.017 |
| c26958_g1_i1 | 230 | 47.3011 | 205.657 |
| c27020_g1_i1 | 253 | 73.0263 | 288.641 |
| c27048_g1_i1 | 378 | 131.115 | 346.866 |
| c27061_g1_i1 | 289 | 233.186 | 806.872 |
| c27105_g1_i1 | 205 | 81.3247 | 396.706 |
| c27108_g1_i1 | 282 | 34.0236 | 120.651 |
| c27147_g1_i1 | 213 | 126.136 | 592.189 |
| c27150_g1_i1 | 288 | 87.1336 | 302.547 |
| c27321_g1_i1 | 229 | 50.6205 | 221.05 |
| c27476_g1_i1 | 207 | 29.8744 | 144.321 |
| c27574_g1_i1 | 256 | 75.5158 | 294.984 |
| c27622_g1_i1 | 460 | 68.877 | 149.733 |
| c27643_g1_i1 | 259 | 247.293 | 954.801 |
| c27668_g1_i1 | 263 | 51.4503 | 195.629 |
| c27670_g1_i1 | 207 | 68.0472 | 328.73 |
| c27716_g1_i1 | 215 | 28.2147 | 131.231 |
| c27758_g1_i1 | 386 | 59.7488 | 154.79 |
| c27762_g1_i1 | 298 | 58.0891 | 194.93 |
| c27775_g1_i1 | 286 | 33.1938 | 116.062 |
| c27864_g1_i1 | 245 | 96.2619 | 392.906 |
| c27894_g1_i1 | 230 | 34.8534 | 151.537 |
| c27945_g1_i1 | 226 | 36.5131 | 161.563 |
| c27945_g2_i1 | 203 | 51.4503 | 253.45 |
| c27948_g1_i1 | 203 | 26.555 | 130.813 |
| c27977_g1_i1 | 256 | 209.121 | 816.878 |
| c27980_g1_i1 | 223 | 41.4922 | 186.064 |
| c27988_g1_i1 | 268 | 253.932 | 947.508 |
| c28038_g1_i1 | 232 | 146.053 | 629.537 |
| c28080_g1_i2 | 354 | 83.8142 | 236.763 |
| c28094_g1_i1 | 256 | 70.5367 | 275.534 |
| c28227_g1_i1 | 351 | 46.4713 | 132.397 |
| c28621_g1_i1 | 215 | 34.8534 | 162.109 |
| c28633_g1_i1 | 246 | 95.432 | 387.935 |
| c28643_g1_i1 | 253 | 100.411 | 396.882 |
| c28685_g1_i1 | 409 | 160.99 | 393.618 |
| c28791_g1_i1 | 214 | 51.4503 | 240.422 |
| c28803_g1_i1 | 203 | 29.0445 | 143.077 |
| c29012_g1_i1 | 262 | 67.2174 | 256.555 |
| c29114_g1_i1 | 256 | 41.4922 | 162.079 |
| c29122_g1_i1 | 250 | 43.1519 | 172.608 |
| c29222_g1_i1 | 458 | 102.901 | 224.674 |
| c29343_g1_i1 | 231 | 96.2619 | 416.718 |
| c29346_g1_i1 | 221 | 66.3875 | 300.396 |
| c29353_g1_i1 | 305 | 47.3011 | 155.086 |
| c29421_g1_i1 | 256 | 125.306 | 489.478 |
| c29448_g1_i1 | 230 | 29.0445 | 126.281 |
| c29523_g1_i1 | 274 | 36.5131 | 133.26 |
| c29559_g1_i1 | 218 | 34.0236 | 156.072 |
| c29609_g1_i1 | 328 | 68.0472 | 207.461 |
| c29696_g1_i1 | 311 | 36.5131 | 117.406 |
| c29696_g2_i1 | 236 | 24.0655 | 101.972 |
| c29711_g1_i1 | 278 | 99.5813 | 358.206 |
| c29850_g1_i1 | 352 | 133.605 | 379.559 |
| c29980_g1_i1 | 589 | 195.013 | 331.092 |
| c30009_g1_i1 | 266 | 53.9398 | 202.781 |
| c30009_g2_i1 | 305 | 53.11 | 174.131 |
| c30143_g1_i1 | 500 | 60.5786 | 121.157 |
| c30175_g1_i1 | 240 | 42.322 | 176.342 |
| c30177_g1_i1 | 209 | 53.9398 | 258.085 |
| c30177_g2_i1 | 242 | 25.7252 | 106.302 |
| c30209_g1_i1 | 205 | 46.4713 | 226.689 |
| c30279_g1_i1 | 263 | 80.4949 | 306.064 |
| c30511_g1_i2 | 242 | 31.5341 | 130.306 |
| c30534_g1_i1 | 207 | 34.0236 | 164.365 |
| c30546_g1_i1 | 228 | 45.6414 | 200.182 |
| c30610_g1_i1 | 300 | 117.838 | 392.793 |
| c30681_g1_i1 | 307 | 634.831 | 2067.85 |
| c30801_g1_i1 | 209 | 45.6414 | 218.38 |
| c30915_g1_i1 | 201 | 57.2592 | 284.872 |
| c30963_g1_i1 | 385 | 72.1964 | 187.523 |
| c31028_g1_i1 | 212 | 66.3875 | 313.149 |
| c31104_g1_i1 | 211 | 34.0236 | 161.249 |
| c31153_g1_i1 | 322 | 64.7278 | 201.018 |
| c31153_g2_i1 | 340 | 86.3038 | 253.835 |
| c31171_g1_i1 | 217 | 22.4058 | 103.252 |
| c31244_g1_i1 | 233 | 57.2592 | 245.748 |
| c31287_g1_i1 | 202 | 48.1309 | 238.272 |
| c31439_g1_i1 | 331 | 82.1545 | 248.201 |
| c31482_g1_i1 | 264 | 26.555 | 100.587 |
| c31597_g1_i1 | 394 | 131.115 | 332.78 |
| c31628_g1_i1 | 255 | 56.4294 | 221.292 |
| c31760_g1_i1 | 274 | 30.7042 | 112.059 |
| c31856_g1_i1 | 203 | 28.2147 | 138.989 |
| c31869_g1_i1 | 225 | 97.0917 | 431.519 |
| c32027_g1_i1 | 322 | 57.2592 | 177.824 |
| c32224_g1_i1 | 216 | 27.3848 | 126.782 |
| c32310_g1_i1 | 274 | 35.6833 | 130.231 |
| c32332_g1_i1 | 480 | 178.416 | 371.701 |
| c32507_g1_i1 | 411 | 50.6205 | 123.164 |
| c32825_g1_i1 | 209 | 147.712 | 706.757 |
| c32843_g1_i1 | 309 | 62.2383 | 201.418 |
| c32891_g1_i1 | 305 | 41.4922 | 136.04 |
| c32932_g1_i1 | 230 | 200.822 | 873.14 |
| c33044_g1_i1 | 224 | 53.9398 | 240.803 |
| c33148_g1_i1 | 334 | 34.8534 | 104.352 |
| c33153_g1_i1 | 234 | 63.0681 | 269.522 |
| c33204_g1_i1 | 259 | 29.8744 | 115.345 |
| c33505_g1_i1 | 223 | 94.6022 | 424.225 |
| c33630_g1_i1 | 237 | 27.3848 | 115.548 |
| c33700_g1_i1 | 281 | 63.0681 | 224.442 |
| c33888_g1_i1 | 522 | 174.267 | 333.845 |
| c33954_g1_i1 | 324 | 51.4503 | 158.797 |
| c34025_g1_i2 | 360 | 73.0263 | 202.851 |
| c34035_g1_i1 | 377 | 58.0891 | 154.082 |
| c34064_g1_i1 | 244 | 65.5577 | 268.679 |
| c34108_g1_i1 | 358 | 48.9608 | 136.762 |
| c34141_g1_i1 | 389 | 52.2802 | 134.396 |
| c34142_g1_i1 | 266 | 77.1755 | 290.133 |
| c34153_g1_i1 | 344 | 39.8325 | 115.792 |
| c34162_g1_i1 | 673 | 304.553 | 452.53 |
| c34162_g2_i1 | 329 | 83.8142 | 254.754 |
| c34275_g1_i1 | 229 | 48.1309 | 210.179 |
| c34322_g1_i1 | 211 | 31.5341 | 149.451 |
| c34444_g1_i1 | 220 | 42.322 | 192.373 |
| c34469_g1_i1 | 272 | 73.8561 | 271.53 |
| c34471_g1_i1 | 375 | 44.8116 | 119.498 |
| c34655_g1_i1 | 344 | 126.966 | 369.088 |
| c34695_g1_i1 | 207 | 46.4713 | 224.499 |
| c34779_g1_i1 | 589 | 69.7069 | 118.348 |
| c34875_g1_i1 | 342 | 39.0027 | 114.043 |
| c34889_g1_i1 | 206 | 164.309 | 797.617 |
| c34889_g2_i1 | 398 | 168.458 | 423.262 |
| c34893_g1_i1 | 231 | 70.5367 | 305.354 |
| c34965_g1_i1 | 329 | 63.898 | 194.219 |
| c34965_g2_i1 | 269 | 33.1938 | 123.397 |
| c35056_g1_i1 | 379 | 195.013 | 514.547 |
| c35075_g1_i1 | 230 | 74.6859 | 324.722 |
| c35082_g1_i1 | 443 | 186.715 | 421.478 |
| c35122_g1_i1 | 397 | 106.22 | 267.557 |
| c35134_g2_i1 | 203 | 24.8953 | 122.637 |
| c35237_g1_i1 | 459 | 54.7697 | 119.324 |
| c35345_g1_i1 | 415 | 53.9398 | 129.976 |
| c35357_g1_i1 | 202 | 62.2383 | 308.11 |
| c35363_g1_i1 | 210 | 71.3666 | 339.841 |
| c35385_g1_i1 | 292 | 31.5341 | 107.993 |
| c35385_g2_i1 | 266 | 73.8561 | 277.655 |
| c35404_g1_i1 | 288 | 51.4503 | 178.647 |
| c35445_g1_i1 | 202 | 99.5813 | 492.977 |
| c35512_g1_i1 | 211 | 42.322 | 200.578 |
| c35518_g1_i1 | 202 | 20.7461 | 102.703 |
| c35751_g1_i1 | 208 | 26.555 | 127.668 |
| c35788_g1_i1 | 525 | 54.7697 | 104.323 |
| c35829_g1_i1 | 465 | 51.4503 | 110.646 |
| c35983_g1_i1 | 238 | 25.7252 | 108.089 |
| c36131_g1_i1 | 257 | 79.665 | 309.981 |
| c36156_g1_i1 | 474 | 86.3038 | 182.075 |
| c36239_g1_i1 | 294 | 82.9844 | 282.26 |
| c36282_g1_i1 | 244 | 39.0027 | 159.847 |
| c36317_g1_i1 | 261 | 74.6859 | 286.153 |
| c36372_g1_i1 | 587 | 67.2174 | 114.51 |
| c36554_g1_i1 | 209 | 22.4058 | 107.205 |
| c36662_g1_i1 | 293 | 208.291 | 710.89 |
| c36662_g2_i1 | 365 | 55.5995 | 152.327 |
| c36677_g1_i2 | 316 | 39.0027 | 123.426 |
| c36679_g2_i1 | 245 | 28.2147 | 115.162 |
| c36681_g1_i1 | 204 | 102.071 | 500.347 |
| c36688_g1_i1 | 443 | 175.927 | 397.126 |
| c36813_g1_i1 | 223 | 39.8325 | 178.621 |
| c36839_g1_i1 | 338 | 52.2802 | 154.675 |
| c36853_g1_i1 | 388 | 47.3011 | 121.91 |
| c36956_g1_i1 | 223 | 176.757 | 792.631 |
| c36956_g2_i1 | 420 | 255.592 | 608.552 |
| c36966_g1_i1 | 582 | 192.524 | 330.797 |
| c37030_g1_i1 | 206 | 20.7461 | 100.709 |
| c37066_g1_i1 | 284 | 33.1938 | 116.879 |
| c37184_g1_i1 | 367 | 77.1755 | 210.287 |
| c37222_g1_i1 | 203 | 49.7906 | 245.274 |
| c37260_g1_i1 | 235 | 45.6414 | 194.219 |
| c37385_g1_i1 | 336 | 95.432 | 284.024 |
| c37424_g1_i1 | 520 | 59.7488 | 114.901 |
| c37527_g1_i1 | 404 | 209.95 | 519.679 |
| c37582_g1_i1 | 218 | 26.555 | 121.812 |
| c37638_g1_i1 | 740 | 530.27 | 716.581 |
| c37645_g1_i1 | 246 | 43.9817 | 178.787 |
| c37664_g1_i1 | 309 | 50.6205 | 163.82 |
| c37678_g1_i1 | 226 | 77.1755 | 341.484 |
| c37680_g1_i1 | 215 | 48.1309 | 223.865 |
| c37686_g1_i1 | 214 | 68.0472 | 317.978 |
| c37762_g1_i1 | 219 | 40.6623 | 185.673 |
| c37812_g1_i1 | 202 | 30.7042 | 152.001 |
| c37836_g1_i1 | 222 | 72.1964 | 325.209 |
| c37887_g2_i1 | 296 | 31.5341 | 106.534 |
| c37891_g1_i1 | 291 | 38.1728 | 131.178 |
| c38121_g1_i1 | 376 | 100.411 | 267.051 |
| c38334_g1_i1 | 234 | 82.1545 | 351.088 |
| c38434_g1_i1 | 254 | 29.8744 | 117.616 |
| c38466_g1_i1 | 272 | 138.584 | 509.5 |
| c38508_g1_i1 | 344 | 219.079 | 636.857 |
| c38508_g2_i1 | 238 | 113.689 | 477.683 |
| c38572_g1_i1 | 333 | 158.5 | 475.976 |
| c38594_g1_i1 | 391 | 462.223 | 1182.16 |
| c38695_g1_i1 | 275 | 52.2802 | 190.11 |
| c38742_g1_i1 | 249 | 47.3011 | 189.964 |
| c38772_g1_i1 | 236 | 36.5131 | 154.717 |
| c38813_g1_i1 | 381 | 65.5577 | 172.067 |
| c38929_g1_i1 | 298 | 36.5131 | 122.527 |
| c39175_g1_i2 | 239 | 33.1938 | 138.886 |
| c39186_g1_i1 | 201 | 34.8534 | 173.4 |
| c39291_g1_i1 | 318 | 60.5786 | 190.499 |
| c39492_g1_i1 | 269 | 225.718 | 839.099 |
| c39704_g1_i1 | 212 | 63.0681 | 297.491 |
| c39845_g1_i1 | 838 | 96.2619 | 114.871 |
| c39933_g1_i1 | 414 | 153.521 | 370.824 |
| c39968_g1_i1 | 308 | 44.8116 | 145.492 |
| c39978_g1_i1 | 297 | 54.7697 | 184.41 |
| c40105_g1_i1 | 325 | 137.754 | 423.859 |
| c40118_g1_i1 | 476 | 166.799 | 350.417 |
| c40157_g2_i1 | 246 | 34.0236 | 138.307 |
| c40251_g1_i1 | 238 | 24.0655 | 101.115 |
| c40255_g1_i1 | 235 | 90.453 | 384.906 |
| c40400_g1_i1 | 339 | 68.0472 | 200.729 |
| c40400_g2_i1 | 415 | 112.859 | 271.949 |
| c40426_g1_i1 | 351 | 44.8116 | 127.668 |
| c40480_g1_i1 | 420 | 42.322 | 100.767 |
| c40493_g1_i1 | 212 | 27.3848 | 129.174 |
| c40652_g1_i1 | 232 | 76.3456 | 329.076 |
| c40655_g1_i1 | 212 | 97.9216 | 461.894 |
| c40661_g1_i1 | 281 | 73.8561 | 262.833 |
| c40689_g1_i1 | 230 | 172.608 | 750.467 |
| c40693_g1_i1 | 469 | 237.335 | 506.045 |
| c40693_g2_i1 | 378 | 166.799 | 441.266 |
| c40918_g1_i1 | 208 | 137.754 | 662.279 |
| c40981_g1_i2 | 530 | 95.432 | 180.06 |
| c40997_g1_i2 | 406 | 51.4503 | 126.725 |
| c41038_g1_i1 | 517 | 165.139 | 319.418 |
| c41045_g1_i1 | 228 | 34.0236 | 149.226 |
| c41045_g1_i2 | 228 | 29.0445 | 127.388 |
| c41128_g1_i1 | 268 | 28.2147 | 105.279 |
| c41200_g1_i1 | 587 | 63.0681 | 107.441 |
| c41236_g1_i1 | 449 | 50.6205 | 112.74 |
| c41426_g1_i1 | 247 | 27.3848 | 110.87 |
| c41428_g1_i1 | 204 | 34.8534 | 170.85 |
| c41432_g2_i1 | 236 | 24.0655 | 101.972 |
| c41625_g1_i1 | 259 | 84.6441 | 326.811 |
| c41761_g1_i1 | 203 | 23.2356 | 114.461 |
| c41810_g1_i1 | 334 | 86.3038 | 258.394 |
| c41816_g1_i1 | 211 | 23.2356 | 110.121 |
| c41843_g1_i1 | 244 | 33.1938 | 136.04 |
| c41912_g1_i1 | 305 | 91.2828 | 299.288 |
| c41982_g1_i1 | 272 | 78.8352 | 289.835 |
| c41997_g1_i1 | 224 | 24.0655 | 107.435 |
| c42091_g1_i1 | 403 | 43.9817 | 109.136 |
| c42160_g1_i1 | 331 | 184.225 | 556.572 |
| c42225_g1_i1 | 326 | 58.9189 | 180.733 |
| c42252_g1_i1 | 212 | 24.0655 | 113.516 |
| c42297_g1_i1 | 278 | 32.3639 | 116.417 |
| c42711_g1_i1 | 263 | 159.33 | 605.818 |
| c42769_g1_i1 | 234 | 92.1127 | 393.644 |
| c42833_g1_i1 | 324 | 72.1964 | 222.828 |
| c43060_g1_i1 | 232 | 32.3639 | 139.5 |
| c43064_g2_i1 | 420 | 50.6205 | 120.525 |
| c43068_g1_i1 | 202 | 74.6859 | 369.732 |
| c43163_g1_i1 | 330 | 160.16 | 485.333 |
| c43193_g1_i2 | 344 | 35.6833 | 103.73 |
| c43197_g1_i1 | 464 | 158.5 | 341.595 |
| c43366_g1_i1 | 523 | 263.06 | 502.984 |
| c43580_g1_i1 | 559 | 76.3456 | 136.575 |
| c43621_g1_i1 | 242 | 37.343 | 154.31 |
| c43680_g1_i1 | 253 | 151.861 | 600.243 |
| c43889_g1_i1 | 266 | 120.327 | 452.358 |
| c43920_g1_i1 | 377 | 147.712 | 391.81 |
| c43976_g1_i1 | 214 | 63.898 | 298.589 |
| c44014_g1_i1 | 201 | 33.1938 | 165.143 |
| c44703_g1_i1 | 228 | 141.903 | 622.383 |
| c44737_g1_i1 | 226 | 85.4739 | 378.203 |
| c44754_g1_i1 | 222 | 68.0472 | 306.519 |
| c44870_g1_i1 | 288 | 73.8561 | 256.445 |
| c44876_g1_i1 | 224 | 51.4503 | 229.689 |
| c44913_g1_i1 | 768 | 112.029 | 145.871 |
| c44969_g1_i1 | 306 | 63.0681 | 206.105 |
| c44982_g1_i1 | 384 | 164.309 | 427.888 |
| c45014_g1_i1 | 362 | 248.123 | 685.424 |
| c45120_g1_i1 | 211 | 52.2802 | 247.773 |
| c45132_g1_i1 | 356 | 87.1336 | 244.757 |
| c45132_g2_i1 | 210 | 98.7514 | 470.245 |
| c45174_g1_i1 | 517 | 68.877 | 133.224 |
| c45224_g2_i1 | 264 | 58.0891 | 220.034 |
| c45243_g1_i1 | 282 | 110.369 | 391.38 |
| c45305_g1_i1 | 305 | 64.7278 | 212.222 |
| c45375_g1_i1 | 206 | 28.2147 | 136.965 |
| c45386_g1_i1 | 302 | 33.1938 | 109.913 |
| c45391_g1_i1 | 252 | 31.5341 | 125.135 |
| c45391_g2_i1 | 223 | 67.2174 | 301.423 |
| c45400_g1_i1 | 210 | 54.7697 | 260.808 |
| c45414_g1_i1 | 394 | 73.8561 | 187.452 |
| c45424_g1_i1 | 345 | 37.343 | 108.241 |
| c45473_g1_i1 | 478 | 607.446 | 1270.81 |
| c45483_g1_i1 | 221 | 110.369 | 499.408 |
| c45490_g1_i1 | 276 | 87.9634 | 318.708 |
| c45554_g1_i1 | 205 | 34.0236 | 165.969 |
| c45554_g2_i1 | 484 | 54.7697 | 113.161 |
| c45570_g1_i1 | 357 | 40.6623 | 113.9 |
| c45605_g1_i1 | 248 | 71.3666 | 287.768 |
| c45620_g1_i1 | 325 | 53.11 | 163.415 |
| c45633_g1_i1 | 394 | 355.173 | 901.455 |
| c45651_g1_i1 | 1025 | 1967.56 | 1919.57 |
| c45707_g1_i1 | 227 | 32.3639 | 142.572 |
| c45784_g1_i1 | 246 | 78.8352 | 320.468 |
| c45866_g1_i1 | 644 | 64.7278 | 100.509 |
| c45868_g1_i1 | 640 | 126.966 | 198.385 |
| c45868_g2_i1 | 434 | 141.073 | 325.054 |
| c45868_g3_i1 | 263 | 27.3848 | 104.125 |
| c45882_g1_i1 | 354 | 102.071 | 288.336 |
| c45932_g1_i1 | 343 | 66.3875 | 193.55 |
| c45959_g1_i1 | 241 | 63.898 | 265.137 |
| c46005_g1_i1 | 461 | 59.7488 | 129.607 |
| c46110_g1_i1 | 255 | 78.8352 | 309.158 |
| c46121_g1_i1 | 230 | 66.3875 | 288.641 |
| c46211_g1_i1 | 221 | 350.194 | 1584.59 |
| c46215_g1_i1 | 202 | 31.5341 | 156.109 |
| c46229_g1_i1 | 205 | 23.2356 | 113.345 |
| c46234_g1_i1 | 232 | 163.479 | 704.652 |
| c46301_g1_i1 | 280 | 42.322 | 151.15 |
| c46372_g2_i1 | 448 | 55.5995 | 124.106 |
| c46453_g1_i1 | 300 | 43.1519 | 143.84 |
| c46510_g1_i1 | 210 | 102.071 | 486.051 |
| c46581_g1_i1 | 241 | 29.0445 | 120.517 |
| c46581_g2_i1 | 287 | 86.3038 | 300.71 |
| c46581_g3_i1 | 272 | 77.1755 | 283.733 |
| c46648_g1_i1 | 407 | 404.134 | 992.958 |
| c46709_g1_i1 | 335 | 283.807 | 847.184 |
| c46756_g1_i1 | 401 | 87.1336 | 217.291 |
| c46803_g1_i1 | 205 | 131.945 | 643.635 |
| c46854_g2_i1 | 275 | 43.1519 | 156.916 |
| c46878_g1_i1 | 368 | 48.1309 | 130.791 |
| c46943_g1_i1 | 370 | 407.453 | 1101.23 |
| c47063_g1_i1 | 222 | 28.2147 | 127.093 |
| c47153_g1_i1 | 394 | 39.8325 | 101.098 |
| c47222_g1_i1 | 380 | 78.0053 | 205.277 |
| c47306_g1_i1 | 553 | 87.9634 | 159.066 |
| c47315_g1_i1 | 321 | 33.1938 | 103.407 |
| c47417_g1_i1 | 248 | 41.4922 | 167.307 |
| c47420_g1_i1 | 245 | 51.4503 | 210.001 |
| c47535_g1_i1 | 219 | 27.3848 | 125.045 |
| c47625_g1_i1 | 237 | 29.8744 | 126.052 |
| c47687_g2_i1 | 274 | 52.2802 | 190.804 |
| c47746_g1_i1 | 297 | 43.9817 | 148.087 |
| c47748_g1_i1 | 602 | 102.901 | 170.931 |
| c47877_g1_i1 | 272 | 28.2147 | 103.73 |
| c47929_g1_i1 | 233 | 39.8325 | 170.955 |
| c47972_g1_i1 | 260 | 79.665 | 306.404 |
| c47972_g2_i1 | 349 | 87.9634 | 252.044 |
| c48028_g1_i1 | 300 | 35.6833 | 118.944 |
| c48074_g1_i1 | 225 | 51.4503 | 228.668 |
| c48090_g1_i1 | 466 | 184.225 | 395.333 |
| c48098_g1_i1 | 392 | 55.5995 | 141.836 |
| c48177_g1_i1 | 346 | 35.6833 | 103.131 |
| c48184_g1_i1 | 275 | 34.0236 | 123.722 |
| c48362_g1_i1 | 268 | 95.432 | 356.09 |
| c48683_g1_i1 | 214 | 60.5786 | 283.078 |
| c48731_g1_i1 | 247 | 40.6623 | 164.625 |
| c48785_g1_i1 | 219 | 33.1938 | 151.57 |
| c48864_g1_i1 | 251 | 27.3848 | 109.103 |
| c48880_g1_i1 | 268 | 207.461 | 774.108 |
| c48886_g1_i1 | 683 | 192.524 | 281.88 |
| c48901_g1_i2 | 259 | 34.8534 | 134.569 |
| c48907_g1_i1 | 211 | 56.4294 | 267.438 |
| c48936_g2_i1 | 326 | 48.9608 | 150.186 |
| c48953_g1_i1 | 293 | 45.6414 | 155.773 |
| c48964_g1_i1 | 405 | 59.7488 | 147.528 |
| c49013_g1_i1 | 225 | 82.1545 | 365.131 |
| c49044_g1_i1 | 427 | 193.354 | 452.819 |
| c49090_g1_i1 | 558 | 97.0917 | 174 |
| c49095_g1_i1 | 298 | 41.4922 | 139.236 |
| c49172_g1_i1 | 493 | 86.3038 | 175.058 |
| c49181_g1_i1 | 648 | 130.285 | 201.058 |
| c49248_g1_i1 | 244 | 32.3639 | 132.639 |
| c49254_g1_i1 | 243 | 241.485 | 993.764 |
| c49296_g1_i1 | 375 | 111.199 | 296.531 |
| c49409_g1_i1 | 222 | 75.5158 | 340.161 |
| c49655_g1_i2 | 344 | 190.864 | 554.837 |
| c49672_g1_i1 | 271 | 29.8744 | 110.238 |
| c49736_g1_i1 | 219 | 126.966 | 579.754 |
| c49786_g1_i1 | 272 | 51.4503 | 189.156 |
| c49801_g1_i1 | 232 | 26.555 | 114.461 |
| c49801_g2_i1 | 362 | 113.689 | 314.057 |
| c49848_g2_i1 | 352 | 42.322 | 120.233 |
| c50130_g1_i1 | 362 | 97.0917 | 268.209 |
| c50153_g1_i1 | 246 | 34.0236 | 138.307 |
| c50172_g1_i1 | 442 | 166.799 | 377.372 |
| c50185_g1_i1 | 228 | 27.3848 | 120.109 |
| c50284_g1_i1 | 262 | 65.5577 | 250.22 |
| c50287_g1_i1 | 260 | 649.768 | 2499.11 |
| c50287_g2_i1 | 225 | 170.948 | 759.768 |
| c50297_g1_i1 | 235 | 29.8744 | 127.125 |
| c50324_g1_i1 | 411 | 250.613 | 609.764 |
| c50324_g2_i1 | 412 | 93.7724 | 227.603 |
| c50344_g1_i1 | 726 | 158.5 | 218.32 |
| c50344_g2_i1 | 455 | 84.6441 | 186.031 |
| c50344_g3_i1 | 586 | 107.05 | 182.679 |
| c50403_g1_i1 | 386 | 52.2802 | 135.441 |
| c50451_g1_i1 | 296 | 118.668 | 400.904 |
| c50497_g1_i1 | 265 | 146.053 | 551.142 |
| c50501_g1_i1 | 755 | 219.909 | 291.27 |
| c50617_g1_i1 | 460 | 114.518 | 248.953 |
| c50674_g1_i1 | 267 | 41.4922 | 155.401 |
| c50689_g1_i1 | 350 | 64.7278 | 184.937 |
| c50705_g1_i1 | 272 | 206.631 | 759.673 |
| c50829_g1_i1 | 208 | 53.11 | 255.337 |
| c50913_g1_i1 | 292 | 29.8744 | 102.31 |
| c51060_g1_i1 | 393 | 97.0917 | 247.053 |
| c51087_g1_i1 | 318 | 163.479 | 514.086 |
| c51124_g1_i1 | 343 | 43.9817 | 128.227 |
| c51134_g1_i1 | 234 | 73.0263 | 312.078 |
| c51207_g1_i1 | 536 | 58.9189 | 109.923 |
| c51293_g1_i1 | 439 | 47.3011 | 107.747 |
| c51298_g1_i1 | 283 | 171.778 | 606.988 |
| c51317_g1_i1 | 764 | 198.333 | 259.598 |
| c51325_g2_i1 | 231 | 26.555 | 114.957 |
| c51396_g1_i1 | 267 | 28.2147 | 105.673 |
| c51396_g2_i1 | 363 | 61.4084 | 169.169 |
| c51406_g1_i1 | 215 | 112.029 | 521.065 |
| c51407_g1_i1 | 205 | 148.542 | 724.595 |
| c51446_g1_i1 | 325 | 37.343 | 114.901 |
| c51521_g1_i1 | 228 | 26.555 | 116.469 |
| c51557_g1_i1 | 358 | 275.508 | 769.576 |
| c51584_g1_i1 | 306 | 153.521 | 501.703 |
| c51632_g1_i1 | 698 | 100.411 | 143.855 |
| c51664_g2_i1 | 526 | 65.5577 | 124.634 |
| c51720_g1_i1 | 494 | 102.901 | 208.301 |
| c51727_g1_i1 | 224 | 81.3247 | 363.057 |
| c51851_g1_i1 | 201 | 27.3848 | 136.243 |
| c51879_g1_i1 | 209 | 29.8744 | 142.94 |
| c51894_g1_i1 | 444 | 45.6414 | 102.796 |
| c51900_g1_i1 | 212 | 43.9817 | 207.461 |
| c52032_g1_i1 | 230 | 49.7906 | 216.481 |
| c52066_g1_i1 | 240 | 102.071 | 425.295 |
| c52169_g1_i1 | 346 | 900.381 | 2602.26 |
| c52261_g1_i1 | 242 | 76.3456 | 315.478 |
| c52272_g1_i1 | 408 | 51.4503 | 126.104 |
| c52273_g1_i1 | 376 | 157.67 | 419.336 |
| c52454_g1_i1 | 239 | 83.8142 | 350.687 |
| c52466_g2_i1 | 312 | 91.2828 | 292.573 |
| c52475_g1_i1 | 329 | 41.4922 | 126.116 |
| c52475_g2_i1 | 306 | 36.5131 | 119.324 |
| c52599_g1_i1 | 231 | 73.8561 | 319.723 |
| c52599_g2_i1 | 245 | 88.7933 | 362.422 |
| c52652_g1_i1 | 209 | 63.0681 | 301.761 |
| c52668_g1_i1 | 263 | 532.76 | 2025.7 |
| c52785_g1_i1 | 312 | 94.6022 | 303.212 |
| c52794_g1_i1 | 203 | 24.8953 | 122.637 |
| c52795_g1_i1 | 365 | 195.843 | 536.557 |
| c52898_g1_i1 | 263 | 49.7906 | 189.318 |
| c52918_g1_i1 | 202 | 20.7461 | 102.703 |
| c52937_g1_i1 | 251 | 90.453 | 360.37 |
| c52942_g1_i1 | 224 | 35.6833 | 159.3 |
| c53029_g1_i1 | 229 | 57.2592 | 250.04 |
| c53166_g1_i1 | 291 | 218.249 | 749.996 |
| c53212_g1_i1 | 260 | 143.563 | 552.165 |
| c53214_g1_i1 | 277 | 63.0681 | 227.683 |
| c53480_g1_i1 | 302 | 92.1127 | 305.009 |
| c53553_g1_i2 | 635 | 77.1755 | 121.536 |
| c53873_g1_i1 | 257 | 585.04 | 2276.42 |
| c53920_g1_i1 | 293 | 36.5131 | 124.618 |
| c54079_g1_i1 | 210 | 114.518 | 545.326 |
| c54088_g1_i1 | 437 | 107.88 | 246.864 |
| c54445_g1_i1 | 259 | 35.6833 | 137.773 |
| c54501_g1_i1 | 215 | 75.5158 | 351.236 |
| c54504_g1_i1 | 294 | 31.5341 | 107.259 |
| c54535_g1_i1 | 983 | 252.273 | 256.635 |
| c54542_g1_i1 | 341 | 36.5131 | 107.077 |
| c54717_g1_i1 | 383 | 175.097 | 457.172 |
| c54717_g2_i1 | 838 | 424.05 | 506.026 |
| c54729_g1_i1 | 209 | 97.9216 | 468.524 |
| c54757_g1_i1 | 218 | 50.6205 | 232.204 |
| c54827_g1_i1 | 246 | 34.8534 | 141.681 |
| c54923_g2_i1 | 329 | 39.0027 | 118.549 |
| c54933_g1_i1 | 229 | 74.6859 | 326.14 |
| c55079_g1_i1 | 235 | 200.822 | 854.563 |
| c55102_g1_i1 | 333 | 720.304 | 2163.08 |
| c55241_g1_i1 | 202 | 68.877 | 340.975 |
| c55345_g1_i1 | 1176 | 171.778 | 146.069 |
| c55345_g2_i1 | 556 | 142.733 | 256.714 |
| c55376_g1_i1 | 369 | 40.6623 | 110.196 |
| c55460_g1_i1 | 261 | 51.4503 | 197.128 |
| c55568_g1_i1 | 517 | 190.034 | 367.571 |
| c55571_g1_i1 | 304 | 43.9817 | 144.677 |
| c55762_g1_i1 | 346 | 66.3875 | 191.871 |
| c55898_g1_i1 | 342 | 77.1755 | 225.659 |
| c55964_g2_i1 | 243 | 29.8744 | 122.94 |
| c56064_g1_i1 | 264 | 78.8352 | 298.618 |
| c56065_g1_i1 | 310 | 57.2592 | 184.707 |
| c56239_g1_i1 | 280 | 41.4922 | 148.186 |
| c56402_g1_i1 | 378 | 45.6414 | 120.744 |
| c56459_g1_i1 | 221 | 25.7252 | 116.403 |
| c56524_g1_i1 | 280 | 28.2147 | 100.767 |
| c56580_g1_i1 | 255 | 42.322 | 165.969 |
| c56580_g2_i1 | 203 | 26.555 | 130.813 |
| c56648_g1_i1 | 657 | 193.354 | 294.298 |
| c56792_g1_i1 | 201 | 89.6231 | 445.886 |
| c56792_g2_i1 | 873 | 194.183 | 222.432 |
| c56829_g1_i1 | 213 | 136.094 | 638.941 |
| c56831_g1_i1 | 309 | 84.6441 | 273.929 |
| c56831_g2_i1 | 248 | 108.71 | 438.345 |
| c57017_g1_i1 | 274 | 42.322 | 154.46 |
| c57118_g1_i1 | 354 | 464.713 | 1312.75 |
| c57133_g1_i1 | 364 | 92.1127 | 253.057 |
| c57165_g1_i1 | 229 | 63.0681 | 275.407 |
| c57165_g2_i1 | 249 | 26.555 | 106.647 |
| c57231_g1_i1 | 423 | 58.9189 | 139.288 |
| c57233_g1_i1 | 303 | 89.6231 | 295.786 |
| c57251_g1_i1 | 240 | 74.6859 | 311.191 |
| c57251_g2_i1 | 243 | 209.121 | 860.579 |
| c57479_g1_i1 | 359 | 48.9608 | 136.381 |
| c57517_g1_i1 | 316 | 190.034 | 601.374 |
| c57521_g1_i1 | 329 | 532.76 | 1619.33 |
| c57539_g1_i1 | 251 | 113.689 | 452.943 |
| c57618_g1_i1 | 335 | 45.6414 | 136.243 |
| c57620_g2_i1 | 219 | 23.2356 | 106.099 |
| c57620_g3_i1 | 264 | 35.6833 | 135.164 |
| c57648_g1_i1 | 556 | 58.0891 | 104.477 |
| c57680_g1_i1 | 319 | 385.048 | 1207.05 |
| c57684_g1_i1 | 356 | 38.1728 | 107.227 |
| c57700_g1_i1 | 218 | 33.1938 | 152.265 |
| c57753_g2_i1 | 261 | 34.0236 | 130.359 |
| c57810_g1_i1 | 207 | 23.2356 | 112.249 |
| c57841_g1_i1 | 279 | 32.3639 | 116 |
| c57925_g1_i1 | 800 | 116.178 | 145.223 |
| c57982_g1_i1 | 297 | 37.343 | 125.734 |
| c57983_g1_i1 | 272 | 46.4713 | 170.85 |
| c58029_g1_i1 | 207 | 29.0445 | 140.312 |
| c58057_g1_i1 | 650 | 82.9844 | 127.668 |
| c58122_g1_i1 | 214 | 33.1938 | 155.111 |
| c58127_g1_i1 | 250 | 39.0027 | 156.011 |
| c58166_g1_i1 | 326 | 131.115 | 402.194 |
| c58218_g1_i1 | 275 | 28.2147 | 102.599 |
| c58225_g1_i1 | 346 | 46.4713 | 134.31 |
| c58229_g1_i1 | 306 | 42.322 | 138.307 |
| c58261_g1_i1 | 342 | 304.553 | 890.505 |
| c58276_g1_i1 | 432 | 106.22 | 245.88 |
| c58290_g1_i1 | 291 | 48.1309 | 165.398 |
| c58290_g2_i1 | 549 | 108.71 | 198.014 |
| c58290_g3_i1 | 299 | 58.9189 | 197.053 |
| c58295_g1_i1 | 458 | 67.2174 | 146.763 |
| c58308_g1_i2 | 467 | 73.0263 | 156.373 |
| c58323_g1_i1 | 724 | 222.398 | 307.18 |
| c58409_g1_i1 | 225 | 33.1938 | 147.528 |
| c58421_g1_i1 | 704 | 87.9634 | 124.948 |
| c58493_g2_i1 | 332 | 36.5131 | 109.979 |
| c58504_g1_i1 | 363 | 611.595 | 1684.83 |
| c58563_g1_i1 | 340 | 231.526 | 680.96 |
| c58563_g2_i1 | 358 | 321.15 | 897.066 |
| c58629_g1_i1 | 272 | 50.6205 | 186.105 |
| c58755_g3_i1 | 285 | 33.1938 | 116.469 |
| c58764_g2_i1 | 261 | 51.4503 | 197.128 |
| c58836_g1_i1 | 566 | 74.6859 | 131.954 |
| c58860_g1_i1 | 207 | 385.048 | 1860.13 |
| c58875_g1_i1 | 228 | 219.909 | 964.511 |
| c58947_g1_i1 | 252 | 46.4713 | 184.41 |
| c58952_g1_i1 | 363 | 68.877 | 189.744 |
| c58995_g2_i1 | 398 | 54.7697 | 137.612 |
| c59029_g1_i1 | 309 | 151.861 | 491.461 |
| c59050_g1_i1 | 283 | 450.605 | 1592.24 |
| c59098_g1_i1 | 738 | 168.458 | 228.263 |
| c59098_g2_i1 | 354 | 45.6414 | 128.931 |
| c59098_g3_i1 | 312 | 111.199 | 356.407 |
| c59212_g1_i1 | 454 | 68.0472 | 149.884 |
| c59304_g1_i1 | 356 | 73.8561 | 207.461 |
| c59304_g2_i1 | 210 | 26.555 | 126.452 |
| c59371_g1_i1 | 436 | 95.432 | 218.881 |
| c59455_g1_i1 | 327 | 33.1938 | 101.51 |
| c59471_g1_i1 | 233 | 58.0891 | 249.309 |
| c59475_g1_i1 | 349 | 48.1309 | 137.911 |
| c59536_g1_i1 | 298 | 84.6441 | 284.041 |
| c59599_g1_i2 | 328 | 151.861 | 462.992 |
| c59680_g1_i1 | 332 | 67.2174 | 202.462 |
| c59757_g1_i1 | 840 | 336.917 | 401.091 |
| c59848_g1_i1 | 283 | 46.4713 | 164.209 |
| c59998_g1_i1 | 261 | 30.7042 | 117.641 |
| c60079_g2_i1 | 291 | 52.2802 | 179.657 |
| c60132_g1_i1 | 218 | 45.6414 | 209.364 |
| c60214_g1_i1 | 310 | 46.4713 | 149.907 |
| c60225_g1_i1 | 303 | 62.2383 | 205.407 |
| c60227_g1_i1 | 209 | 33.1938 | 158.822 |
| c60267_g1_i1 | 666 | 185.885 | 279.107 |
| c60289_g1_i1 | 464 | 84.6441 | 182.423 |
| c60328_g1_i1 | 237 | 111.199 | 469.194 |
| c60328_g2_i1 | 370 | 277.998 | 751.345 |
| c60554_g2_i2 | 874 | 283.807 | 324.722 |
| c60564_g1_i1 | 324 | 94.6022 | 291.982 |
| c60564_g2_i1 | 292 | 49.7906 | 170.516 |
| c60580_g1_i1 | 302 | 64.7278 | 214.331 |
| c60580_g1_i2 | 328 | 37.343 | 113.851 |
| c60591_g2_i1 | 202 | 21.5759 | 106.812 |
| c60909_g2_i1 | 219 | 32.3639 | 147.78 |
| c60931_g1_i1 | 235 | 117.008 | 497.906 |
| c61022_g1_i1 | 264 | 108.71 | 411.779 |
| c61075_g1_i1 | 215 | 29.0445 | 135.091 |
| c61128_g1_i1 | 365 | 98.7514 | 270.552 |
| c61128_g2_i1 | 246 | 34.0236 | 138.307 |
| c61201_g1_i1 | 224 | 389.197 | 1737.49 |
| c61226_g1_i1 | 210 | 45.6414 | 217.34 |
| c61242_g1_i1 | 245 | 30.7042 | 125.323 |
| c61247_g1_i1 | 321 | 47.3011 | 147.355 |
| c61329_g1_i1 | 245 | 63.0681 | 257.421 |
| c61343_g1_i1 | 280 | 70.5367 | 251.917 |
| c61413_g1_i1 | 382 | 42.322 | 110.791 |
| c61479_g1_i1 | 548 | 74.6859 | 136.288 |
| c61506_g1_i1 | 389 | 43.9817 | 113.064 |
| c61510_g1_i1 | 257 | 40.6623 | 158.219 |
| c61513_g1_i1 | 264 | 48.9608 | 185.458 |
| c61542_g1_i1 | 302 | 127.796 | 423.165 |
| c61586_g1_i1 | 250 | 68.0472 | 272.189 |
| c61597_g1_i1 | 394 | 70.5367 | 179.027 |
| c61748_g1_i1 | 755 | 143.563 | 190.15 |
| c61760_g1_i1 | 273 | 45.6414 | 167.185 |
| c61767_g1_i1 | 323 | 43.9817 | 136.166 |
| c61774_g1_i1 | 260 | 30.7042 | 118.093 |
| c61801_g1_i1 | 334 | 43.9817 | 131.682 |
| c61856_g1_i1 | 421 | 52.2802 | 124.181 |
| c61872_g1_i2 | 289 | 123.647 | 427.843 |
| c61873_g1_i1 | 268 | 302.063 | 1127.1 |
| c61966_g2_i1 | 286 | 33.1938 | 116.062 |
| c61997_g1_i1 | 226 | 51.4503 | 227.656 |
| c62067_g1_i1 | 312 | 132.775 | 425.561 |
| c62070_g1_i1 | 464 | 409.113 | 881.709 |
| c62070_g2_i1 | 480 | 282.147 | 587.806 |
| c62098_g1_i1 | 463 | 54.7697 | 118.293 |
| c62099_g1_i1 | 311 | 80.4949 | 258.826 |
| c62099_g2_i1 | 801 | 138.584 | 173.014 |
| c62099_g3_i1 | 202 | 54.7697 | 271.137 |
| c62123_g1_i1 | 291 | 31.5341 | 108.364 |
| c62123_g3_i1 | 249 | 58.9189 | 236.622 |
| c62123_g4_i1 | 577 | 92.1127 | 159.641 |
| c62180_g1_i1 | 418 | 164.309 | 393.084 |
| c62436_g1_i1 | 227 | 58.9189 | 259.555 |
| c62438_g1_i1 | 316 | 93.7724 | 296.748 |
| c62523_g1_i1 | 534 | 97.9216 | 183.374 |
| c62586_g1_i1 | 474 | 98.7514 | 208.336 |
| c62602_g1_i1 | 446 | 45.6414 | 102.335 |
| c62619_g1_i1 | 529 | 67.2174 | 127.065 |
| c62619_g2_i1 | 386 | 52.2802 | 135.441 |
| c62687_g1_i1 | 506 | 64.7278 | 127.921 |
| c62799_g1_i1 | 215 | 55.5995 | 258.602 |
| c62829_g1_i1 | 215 | 32.3639 | 150.53 |
| c62919_g1_i1 | 326 | 41.4922 | 127.277 |
| c62942_g1_i1 | 234 | 144.393 | 617.063 |
| c62942_g2_i1 | 671 | 184.225 | 274.553 |
| c63022_g1_i1 | 207 | 33.1938 | 160.356 |
| c63054_g1_i1 | 463 | 69.7069 | 150.555 |
| c63054_g2_i1 | 360 | 81.3247 | 225.902 |
| c63082_g1_i1 | 265 | 33.1938 | 125.259 |
| c63123_g1_i1 | 414 | 114.518 | 276.615 |
| c63160_g1_i1 | 539 | 102.071 | 189.371 |
| c63190_g1_i1 | 367 | 134.435 | 366.307 |
| c63194_g1_i1 | 343 | 67.2174 | 195.969 |
| c63287_g1_i1 | 245 | 127.796 | 521.616 |
| c63378_g1_i1 | 329 | 35.6833 | 108.46 |
| c63527_g1_i1 | 280 | 30.7042 | 109.658 |
| c63527_g2_i1 | 278 | 74.6859 | 268.654 |
| c63537_g1_i1 | 538 | 57.2592 | 106.43 |
| c63676_g1_i1 | 249 | 48.1309 | 193.297 |
| c63740_g1_i1 | 710 | 114.518 | 161.294 |
| c63740_g2_i1 | 211 | 27.3848 | 129.786 |
| c63801_g2_i1 | 255 | 26.555 | 104.137 |
| c63889_g1_i2 | 1193 | 127.796 | 107.122 |
| c63958_g1_i1 | 606 | 269.699 | 445.048 |
| c63958_g2_i1 | 230 | 65.5577 | 285.033 |
| c64072_g1_i1 | 237 | 30.7042 | 129.554 |
| c64097_g1_i1 | 232 | 233.186 | 1005.11 |
| c64122_g2_i1 | 211 | 53.11 | 251.706 |
| c64156_g1_i1 | 278 | 254.762 | 916.41 |
| c64184_g1_i1 | 358 | 39.0027 | 108.946 |
| c64189_g1_i1 | 325 | 39.8325 | 122.562 |
| c64448_g1_i1 | 598 | 77.1755 | 129.056 |
| c64520_g1_i1 | 299 | 146.882 | 491.245 |
| c64549_g1_i1 | 264 | 67.2174 | 254.611 |
| c64593_g1_i1 | 249 | 35.6833 | 143.306 |
| c64635_g1_i1 | 377 | 44.8116 | 118.864 |
| c64709_g1_i1 | 225 | 43.9817 | 195.474 |
| c64752_g1_i1 | 287 | 57.2592 | 199.509 |
| c64818_g1_i1 | 372 | 394.176 | 1059.61 |
| c64818_g2_i2 | 1091 | 154.351 | 141.477 |
| c64838_g1_i1 | 206 | 87.9634 | 427.007 |
| c64848_g2_i1 | 300 | 47.3011 | 157.67 |
| c64857_g1_i1 | 300 | 60.5786 | 201.929 |
| c64899_g1_i1 | 264 | 100.411 | 380.345 |
| c64925_g1_i1 | 238 | 55.5995 | 233.612 |
| c64936_g1_i1 | 212 | 111.199 | 524.524 |
| c64956_g1_i1 | 514 | 114.518 | 222.799 |
| c65028_g1_i1 | 304 | 129.456 | 425.841 |
| c65287_g1_i1 | 496 | 54.7697 | 110.423 |
| c65331_g1_i1 | 573 | 181.736 | 317.165 |
| c65447_g1_i1 | 448 | 336.917 | 752.046 |
| c65447_g2_i1 | 259 | 197.503 | 762.559 |
| c65468_g2_i1 | 258 | 29.8744 | 115.792 |
| c65482_g1_i1 | 221 | 43.1519 | 195.257 |
| c65578_g1_i1 | 401 | 58.0891 | 144.861 |
| c65692_g1_i1 | 201 | 50.6205 | 251.843 |
| c65707_g1_i1 | 390 | 53.9398 | 138.307 |
| c65834_g1_i1 | 323 | 99.5813 | 308.301 |
| c65840_g1_i1 | 345 | 36.5131 | 105.835 |
| c65848_g3_i1 | 247 | 76.3456 | 309.092 |
| c65876_g1_i1 | 273 | 83.8142 | 307.012 |
| c65882_g1_i1 | 214 | 36.5131 | 170.622 |
| c65882_g2_i1 | 454 | 82.1545 | 180.957 |
| c65883_g2_i1 | 320 | 63.0681 | 197.088 |
| c65905_g1_i1 | 243 | 48.1309 | 198.07 |
| c65975_g1_i1 | 404 | 83.8142 | 207.461 |
| c66032_g1_i1 | 265 | 66.3875 | 250.519 |
| c66123_g2_i1 | 470 | 62.2383 | 132.422 |
| c66135_g1_i1 | 295 | 36.5131 | 123.773 |
| c66144_g1_i1 | 336 | 52.2802 | 155.596 |
| c66307_g1_i1 | 382 | 43.1519 | 112.963 |
| c66352_g1_i1 | 327 | 68.877 | 210.633 |
| c66352_g2_i1 | 407 | 60.5786 | 148.842 |
| c66368_g1_i1 | 230 | 36.5131 | 158.753 |
| c66525_g1_i1 | 285 | 297.914 | 1045.31 |
| c66573_g1_i1 | 394 | 276.338 | 701.365 |
| c66679_g1_i1 | 223 | 136.924 | 614.01 |
| c66679_g2_i1 | 256 | 104.56 | 408.439 |
| c66684_g1_i1 | 353 | 40.6623 | 115.191 |
| c66684_g2_i1 | 215 | 29.8744 | 138.951 |
| c66711_g1_i1 | 844 | 222.398 | 263.505 |
| c66786_g1_i1 | 288 | 325.299 | 1129.51 |
| c66801_g1_i1 | 481 | 63.0681 | 131.119 |
| c66833_g1_i1 | 218 | 24.0655 | 110.392 |
| c66960_g1_i1 | 220 | 22.4058 | 101.844 |
| c66986_g1_i1 | 373 | 171.778 | 460.53 |
| c67058_g1_i1 | 283 | 32.3639 | 114.36 |
| c67061_g1_i1 | 205 | 100.411 | 489.81 |
| c67061_g2_i1 | 227 | 43.9817 | 193.752 |
| c67082_g1_i2 | 1122 | 100.411 | 89.493 |
| c67119_g1_i1 | 421 | 85.4739 | 203.026 |
| c67162_g1_i3 | 342 | 61.4084 | 179.557 |
| c67238_g1_i1 | 551 | 158.5 | 287.659 |
| c67596_g1_i1 | 262 | 37.343 | 142.53 |
| c67610_g1_i1 | 352 | 87.9634 | 249.896 |
| c67651_g1_i1 | 238 | 40.6623 | 170.85 |
| c67656_g1_i1 | 348 | 42.322 | 121.615 |
| c67676_g1_i1 | 209 | 39.8325 | 190.586 |
| c67765_g1_i1 | 218 | 26.555 | 121.812 |
| c67772_g1_i1 | 941 | 1124.44 | 1194.94 |
| c67779_g1_i1 | 425 | 45.6414 | 107.392 |
| c67799_g1_i1 | 403 | 41.4922 | 102.958 |
| c67801_g1_i1 | 488 | 69.7069 | 142.842 |
| c67880_g1_i1 | 221 | 58.9189 | 266.601 |
| c67880_g2_i1 | 373 | 65.5577 | 175.758 |
| c67887_g1_i1 | 353 | 89.6231 | 253.89 |
| c67920_g1_i1 | 502 | 218.249 | 434.759 |
| c67964_g1_i2 | 265 | 38.1728 | 144.048 |
| c67998_g1_i1 | 287 | 58.0891 | 202.401 |
| c68040_g1_i1 | 348 | 38.1728 | 109.692 |
| c68040_g2_i1 | 655 | 68.0472 | 103.889 |
| c68146_g1_i1 | 266 | 86.3038 | 324.45 |
| c68149_g1_i1 | 463 | 70.5367 | 152.347 |
| c68218_g1_i1 | 204 | 28.2147 | 138.307 |
| c68218_g1_i2 | 211 | 21.5759 | 102.256 |
| c68324_g2_i1 | 657 | 318.66 | 485.023 |
| c68324_g3_i1 | 448 | 191.694 | 427.888 |
| c68394_g1_i1 | 549 | 83.8142 | 152.667 |
| c68402_g1_i1 | 273 | 32.3639 | 118.549 |
| c68481_g1_i1 | 370 | 75.5158 | 204.097 |
| c68531_g1_i1 | 527 | 73.0263 | 138.57 |
| c68598_g1_i1 | 676 | 95.432 | 141.172 |
| c68666_g1_i1 | 222 | 45.6414 | 205.592 |
| c68708_g1_i1 | 210 | 56.4294 | 268.711 |
| c68711_g1_i2 | 225 | 26.555 | 118.022 |
| c68809_g1_i1 | 269 | 42.322 | 157.331 |
| c68814_g1_i1 | 527 | 55.5995 | 105.502 |
| c68817_g1_i1 | 897 | 107.88 | 120.267 |
| c68823_g1_i1 | 592 | 87.1336 | 147.185 |
| c68824_g2_i1 | 2072 | 162.649 | 78.4987 |
| c68882_g1_i1 | 276 | 54.7697 | 198.441 |
| c68882_g1_i2 | 264 | 29.0445 | 110.017 |
| c68899_g1_i1 | 466 | 96.2619 | 206.571 |
| c68899_g1_i2 | 371 | 97.0917 | 261.703 |
| c68993_g1_i1 | 299 | 43.1519 | 144.321 |
| c69047_g1_i1 | 1113 | 416.582 | 374.287 |
| c69092_g1_i1 | 374 | 80.4949 | 215.227 |
| c69092_g2_i1 | 295 | 71.3666 | 241.921 |
| c69113_g1_i1 | 297 | 32.3639 | 108.969 |
| c69131_g1_i1 | 319 | 38.1728 | 119.664 |
| c69181_g1_i1 | 323 | 134.435 | 416.207 |
| c69250_g1_i1 | 454 | 129.456 | 285.145 |
| c69311_g1_i1 | 236 | 295.424 | 1251.8 |
| c69347_g1_i1 | 291 | 39.8325 | 136.881 |
| c69370_g1_i1 | 351 | 136.094 | 387.733 |
| c69370_g2_i1 | 421 | 358.493 | 851.526 |
| c69397_g2_i1 | 461 | 95.432 | 207.011 |
| c69397_g3_i1 | 392 | 114.518 | 292.139 |
| c69429_g1_i1 | 403 | 286.296 | 710.412 |
| c69429_g2_i1 | 248 | 278.828 | 1124.3 |
| c69442_g1_i1 | 489 | 53.9398 | 110.306 |
| c69452_g1_i1 | 567 | 121.987 | 215.145 |
| c69490_g1_i1 | 390 | 62.2383 | 159.585 |
| c69532_g1_i1 | 467 | 101.241 | 216.79 |
| c69701_g1_i1 | 228 | 29.0445 | 127.388 |
| c69792_g3_i1 | 203 | 46.4713 | 228.922 |
| c69809_g1_i1 | 457 | 91.2828 | 199.744 |
| c69819_g1_i1 | 267 | 46.4713 | 174.05 |
| c69829_g1_i1 | 313 | 131.945 | 421.55 |
| c69829_g2_i1 | 480 | 120.327 | 250.682 |
| c69830_g1_i1 | 397 | 68.877 | 173.494 |
| c69921_g1_i2 | 818 | 149.372 | 182.606 |
| c70022_g1_i1 | 873 | 110.369 | 126.425 |
| c70029_g1_i1 | 285 | 39.8325 | 139.763 |
| c70101_g1_i1 | 227 | 41.4922 | 182.785 |
| c70105_g1_i1 | 269 | 44.8116 | 166.586 |
| c70105_g2_i1 | 855 | 118.668 | 138.793 |
| c70149_g1_i1 | 820 | 144.393 | 176.089 |
| c70175_g1_i1 | 213 | 52.2802 | 245.447 |
| c70187_g1_i1 | 514 | 53.9398 | 104.941 |
| c70188_g1_i1 | 274 | 62.2383 | 227.147 |
| c70213_g1_i1 | 361 | 82.1545 | 227.575 |
| c70215_g1_i1 | 217 | 133.605 | 615.691 |
| c70236_g1_i1 | 623 | 87.9634 | 141.193 |
| c70250_g1_i1 | 1087 | 141.903 | 130.546 |
| c70325_g1_i1 | 349 | 204.971 | 587.311 |
| c70325_g1_i2 | 218 | 78.0053 | 357.823 |
| c70356_g1_i1 | 342 | 199.163 | 582.347 |
| c70458_g1_i1 | 335 | 81.3247 | 242.76 |
| c70533_g1_i1 | 530 | 74.6859 | 140.917 |
| c70554_g1_i1 | 234 | 34.8534 | 148.946 |
| c70559_g2_i1 | 284 | 63.0681 | 222.071 |
| c70715_g1_i1 | 363 | 232.356 | 640.1 |
| c70748_g2_i1 | 330 | 48.9608 | 148.366 |
| c70761_g1_i1 | 418 | 98.7514 | 236.247 |
| c70764_g2_i1 | 218 | 52.2802 | 239.817 |
| c70803_g1_i1 | 649 | 141.073 | 217.37 |
| c70815_g1_i1 | 321 | 60.5786 | 188.718 |
| c70866_g1_i1 | 214 | 39.8325 | 186.133 |
| c70910_g1_i1 | 510 | 126.136 | 247.326 |
| c70912_g1_i1 | 247 | 132.775 | 537.551 |
| c70934_g1_i1 | 317 | 64.7278 | 204.189 |
| c70967_g1_i1 | 280 | 390.027 | 1392.95 |
| c71028_g1_i1 | 391 | 170.948 | 437.207 |
| c71143_g1_i1 | 305 | 31.5341 | 103.39 |
| c71165_g1_i1 | 464 | 106.22 | 228.922 |
| c71230_g1_i2 | 819 | 90.453 | 110.443 |
| c71243_g1_i1 | 537 | 263.89 | 491.416 |
| c71243_g2_i1 | 347 | 48.1309 | 138.706 |
| c71515_g1_i1 | 352 | 290.445 | 825.129 |
| c71596_g1_i1 | 285 | 73.0263 | 256.232 |
| c71669_g1_i1 | 322 | 43.1519 | 134.012 |
| c71729_g1_i1 | 408 | 107.88 | 264.411 |
| c71729_g2_i1 | 334 | 168.458 | 504.366 |
| c71743_g1_i1 | 217 | 36.5131 | 168.263 |
| c71791_g1_i1 | 240 | 30.7042 | 127.934 |
| c71863_g1_i1 | 1697 | 432.349 | 254.772 |
| c71878_g1_i1 | 530 | 70.5367 | 133.088 |
| c71878_g2_i1 | 348 | 36.5131 | 104.923 |
| c71977_g1_i1 | 410 | 130.285 | 317.769 |
| c72061_g1_i1 | 364 | 73.0263 | 200.622 |
| c72138_g1_i1 | 366 | 59.7488 | 163.248 |
| c72210_g1_i1 | 206 | 70.5367 | 342.411 |
| c72224_g1_i1 | 321 | 197.503 | 615.274 |
| c72243_g1_i1 | 387 | 118.668 | 306.635 |
| c72243_g2_i1 | 392 | 146.053 | 372.583 |
| c72245_g2_i1 | 306 | 74.6859 | 244.072 |
| c72273_g1_i1 | 621 | 82.9844 | 133.63 |
| c72300_g1_i1 | 458 | 75.5158 | 164.882 |
| c72386_g1_i1 | 395 | 127.796 | 323.534 |
| c72394_g1_i1 | 758 | 95.432 | 125.9 |
| c72429_g1_i1 | 296 | 75.5158 | 255.121 |
| c72435_g2_i2 | 432 | 43.9817 | 101.81 |
| c72436_g1_i1 | 401 | 321.15 | 800.872 |
| c72450_g1_i1 | 399 | 71.3666 | 178.864 |
| c72459_g1_i1 | 426 | 43.1519 | 101.295 |
| c72727_g1_i1 | 987 | 145.223 | 147.135 |
| c72749_g1_i1 | 277 | 47.3011 | 170.762 |
| c72783_g2_i1 | 341 | 41.4922 | 121.678 |
| c72823_g2_i1 | 384 | 363.472 | 946.541 |
| c72896_g1_i1 | 300 | 192.524 | 641.746 |
| c72896_g2_i2 | 347 | 53.9398 | 155.446 |
| c72923_g1_i1 | 250 | 109.539 | 438.158 |
| c72923_g2_i1 | 355 | 156.011 | 439.467 |
| c72986_g1_i1 | 439 | 61.4084 | 139.883 |
| c73116_g1_i1 | 217 | 43.1519 | 198.857 |
| c73116_g1_i2 | 239 | 101.241 | 423.602 |
| c73129_g2_i1 | 434 | 53.11 | 122.373 |
| c73242_g1_i1 | 221 | 97.0917 | 439.329 |
| c73250_g1_i1 | 591 | 106.22 | 179.729 |
| c73263_g1_i1 | 674 | 146.053 | 216.695 |
| c73264_g1_i2 | 1227 | 354.343 | 288.788 |
| c73352_g1_i1 | 909 | 121.157 | 133.286 |
| c73386_g1_i1 | 221 | 63.898 | 289.131 |
| c73442_g2_i1 | 361 | 65.5577 | 181.6 |
| c73490_g1_i1 | 351 | 42.322 | 120.576 |
| c73502_g1_i1 | 284 | 219.909 | 774.326 |
| c73502_g2_i1 | 384 | 282.977 | 736.919 |
| c73518_g1_i1 | 223 | 57.2592 | 256.768 |
| c73519_g1_i1 | 279 | 36.5131 | 130.871 |
| c73547_g1_i1 | 600 | 115.348 | 192.247 |
| c73570_g1_i1 | 613 | 448.116 | 731.021 |
| c73685_g1_i1 | 278 | 126.966 | 456.713 |
| c73690_g2_i1 | 243 | 39.8325 | 163.92 |
| c73712_g1_i1 | 579 | 291.275 | 503.066 |
| c73712_g2_i1 | 365 | 293.765 | 804.835 |
| c73759_g1_i1 | 312 | 42.322 | 135.648 |
| c73781_g1_i1 | 267 | 53.11 | 198.914 |
| c73788_g1_i1 | 497 | 121.157 | 243.777 |
| c73795_g1_i1 | 644 | 401.644 | 623.671 |
| c73805_g1_i1 | 357 | 131.945 | 369.594 |
| c73833_g1_i1 | 420 | 91.2828 | 217.34 |
| c73833_g2_i1 | 475 | 159.33 | 335.432 |
| c73984_g1_i2 | 306 | 33.1938 | 108.476 |
| c74057_g1_i1 | 236 | 189.204 | 801.714 |
| c74075_g1_i1 | 316 | 255.592 | 808.835 |
| c74086_g1_i1 | 223 | 38.1728 | 171.179 |
| c74086_g2_i1 | 273 | 43.9817 | 161.105 |
| c74156_g1_i1 | 224 | 37.343 | 166.71 |
| c74156_g1_i2 | 1100 | 297.084 | 270.076 |
| c74156_g2_i1 | 368 | 143.563 | 390.117 |
| c74158_g1_i1 | 406 | 211.61 | 521.207 |
| c74163_g1_i1 | 318 | 133.605 | 420.141 |
| c74177_g1_i1 | 202 | 49.7906 | 246.488 |
| c74196_g1_i1 | 730 | 176.757 | 242.133 |
| c74246_g1_i1 | 216 | 34.8534 | 161.359 |
| c74253_g1_i1 | 524 | 54.7697 | 104.522 |
| c74332_g1_i1 | 210 | 26.555 | 126.452 |
| c74360_g1_i1 | 1216 | 100.411 | 82.5749 |
| c74397_g1_i1 | 213 | 24.8953 | 116.879 |
| c74423_g1_i1 | 557 | 131.115 | 235.396 |
| c74434_g1_i1 | 374 | 212.44 | 568.021 |
| c74438_g1_i1 | 485 | 69.7069 | 143.726 |
| c74535_g1_i1 | 1227 | 479.65 | 390.913 |
| c74561_g1_i1 | 407 | 143.563 | 352.735 |
| c74618_g1_i1 | 253 | 94.6022 | 373.922 |
| c74618_g3_i1 | 204 | 75.5158 | 370.175 |
| c74622_g1_i1 | 422 | 334.427 | 792.481 |
| c74636_g1_i1 | 927 | 310.362 | 334.802 |
| c74690_g2_i1 | 451 | 124.477 | 276.001 |
| c74691_g1_i1 | 285 | 98.7514 | 346.496 |
| c74701_g1_i1 | 331 | 164.309 | 496.402 |
| c74747_g1_i1 | 758 | 125.306 | 165.312 |
| c74949_g1_i1 | 348 | 143.563 | 412.537 |
| c74949_g3_i1 | 234 | 87.1336 | 372.366 |
| c75061_g2_i1 | 453 | 172.608 | 381.032 |
| c75088_g2_i1 | 482 | 87.9634 | 182.497 |
| c75102_g2_i1 | 249 | 145.223 | 583.224 |
| c75129_g1_i1 | 353 | 234.016 | 662.935 |
| c75158_g1_i1 | 307 | 34.0236 | 110.826 |
| c75158_g2_i1 | 223 | 31.5341 | 141.408 |
| c75210_g1_i1 | 394 | 112.029 | 284.337 |
| c75210_g2_i1 | 487 | 119.498 | 245.375 |
| c75376_g1_i1 | 312 | 87.1336 | 279.274 |
| c75392_g1_i1 | 221 | 101.241 | 458.104 |
| c75418_g1_i1 | 215 | 23.2356 | 108.073 |
| c75434_g1_i3 | 305 | 77.1755 | 253.034 |
| c75599_g1_i1 | 308 | 35.6833 | 115.855 |
| c75672_g1_i2 | 516 | 238.995 | 463.169 |
| c75710_g1_i1 | 791 | 170.118 | 215.067 |
| c75837_g1_i1 | 309 | 248.123 | 802.988 |
| c75855_g1_i1 | 392 | 39.8325 | 101.614 |
| c75913_g1_i1 | 448 | 46.4713 | 103.73 |
| c75913_g1_i2 | 371 | 39.0027 | 105.128 |
| c75961_g1_i1 | 440 | 92.9425 | 211.233 |
| c75961_g2_i1 | 449 | 110.369 | 245.811 |
| c76009_g1_i1 | 996 | 804.949 | 808.181 |
| c76056_g1_i1 | 709 | 108.71 | 153.328 |
| c76099_g1_i1 | 326 | 165.969 | 509.107 |
| c76099_g2_i1 | 444 | 183.395 | 413.053 |
| c76177_g1_i1 | 339 | 36.5131 | 107.708 |
| c76181_g1_i1 | 281 | 45.6414 | 162.425 |
| c76253_g1_i1 | 268 | 155.181 | 579.033 |
| c76349_g1_i1 | 231 | 36.5131 | 158.065 |
| c76349_g2_i1 | 201 | 106.22 | 528.458 |
| c76349_g2_i2 | 213 | 38.1728 | 179.215 |
| c76433_g1_i1 | 486 | 463.883 | 954.491 |
| c76440_g1_i1 | 293 | 294.595 | 1005.44 |
| c76674_g1_i1 | 840 | 210.78 | 250.929 |
| c76674_g2_i1 | 375 | 107.05 | 285.466 |
| c76858_g1_i1 | 261 | 195.843 | 750.357 |
| c76861_g2_i1 | 246 | 27.3848 | 111.321 |
| c76957_g1_i1 | 210 | 104.56 | 497.906 |
| c76957_g1_i3 | 263 | 142.733 | 542.712 |
| c76961_g1_i1 | 422 | 147.712 | 350.029 |
| c76961_g2_i1 | 409 | 60.5786 | 148.114 |
| c76973_g2_i1 | 391 | 59.7488 | 152.81 |
| c76973_g3_i1 | 218 | 75.5158 | 346.403 |
| c76973_g4_i1 | 319 | 35.6833 | 111.86 |
| c77000_g1_i1 | 668 | 142.733 | 213.672 |
| c77000_g1_i2 | 435 | 448.116 | 1030.15 |
| c77000_g2_i2 | 310 | 92.9425 | 299.815 |
| c77023_g1_i1 | 369 | 852.25 | 2309.62 |
| c77023_g2_i1 | 452 | 299.574 | 662.774 |
| c77030_g1_i1 | 204 | 124.477 | 610.179 |
| c77030_g3_i1 | 202 | 73.0263 | 361.516 |
| c77061_g1_i1 | 1056 | 119.498 | 113.161 |
| c77061_g2_i1 | 763 | 299.574 | 392.626 |
| c77061_g3_i1 | 750 | 77.1755 | 102.901 |
| c77078_g1_i1 | 403 | 229.867 | 570.389 |
| c77099_g2_i1 | 249 | 105.39 | 423.254 |
| c77119_g1_i1 | 432 | 105.39 | 243.959 |
| c77129_g1_i1 | 260 | 32.3639 | 124.477 |
| c77133_g1_i1 | 391 | 41.4922 | 106.118 |
| c77133_g2_i1 | 284 | 45.6414 | 160.709 |
| c77159_g1_i1 | 446 | 240.655 | 539.585 |
| c77183_g1_i1 | 560 | 238.165 | 425.295 |
| c77247_g1_i1 | 291 | 296.254 | 1018.06 |
| c77247_g2_i1 | 257 | 200.822 | 781.409 |
| c77281_g1_i1 | 762 | 225.718 | 296.217 |
| c77318_g1_i1 | 324 | 86.3038 | 266.37 |
| c77362_g2_i1 | 861 | 189.204 | 219.75 |
| c77362_g3_i1 | 553 | 95.432 | 172.572 |
| c77381_g1_i1 | 222 | 46.4713 | 209.33 |
| c77381_g2_i2 | 500 | 65.5577 | 131.115 |
| c77385_g3_i1 | 222 | 143.563 | 646.68 |
| c77438_g2_i1 | 393 | 230.697 | 587.014 |
| c77519_g1_i1 | 395 | 55.5995 | 140.758 |
| c77535_g1_i1 | 431 | 120.327 | 279.182 |
| c77539_g1_i1 | 609 | 70.5367 | 115.824 |
| c77559_g1_i1 | 521 | 68.0472 | 130.609 |
| c77574_g1_i1 | 683 | 113.689 | 166.455 |
| c77680_g2_i1 | 329 | 55.5995 | 168.996 |
| c77680_g3_i1 | 407 | 199.163 | 489.343 |
| c77777_g1_i1 | 385 | 43.1519 | 112.083 |
| c77825_g1_i1 | 249 | 85.4739 | 343.269 |
| c77825_g2_i1 | 712 | 216.589 | 304.198 |
| c77914_g1_i1 | 462 | 53.9398 | 116.753 |
| c77954_g1_i1 | 496 | 224.888 | 453.403 |
| c77954_g2_i1 | 338 | 140.244 | 414.922 |
| c77982_g2_i1 | 806 | 487.948 | 605.395 |
| c78086_g2_i1 | 616 | 111.199 | 180.518 |
| c78086_g3_i1 | 502 | 87.9634 | 175.226 |
| c78087_g1_i1 | 1023 | 113.689 | 111.133 |
| c78091_g1_i2 | 252 | 36.5131 | 144.893 |
| c78135_g1_i1 | 303 | 263.89 | 870.925 |
| c78205_g2_i1 | 244 | 68.0472 | 278.882 |
| c78240_g1_i1 | 322 | 56.4294 | 175.247 |
| c78267_g1_i1 | 289 | 43.9817 | 152.186 |
| c78277_g2_i1 | 448 | 65.5577 | 146.334 |
| c78292_g1_i1 | 520 | 264.72 | 509.077 |
| c78395_g1_i1 | 208 | 70.5367 | 339.119 |
| c78455_g1_i1 | 423 | 225.718 | 533.611 |
| c78482_g1_i1 | 207 | 51.4503 | 248.552 |
| c78550_g1_i2 | 317 | 108.71 | 342.932 |
| c78606_g1_i1 | 361 | 95.432 | 264.355 |
| c78740_g1_i1 | 236 | 84.6441 | 358.661 |
| c78766_g1_i1 | 458 | 61.4084 | 134.08 |
| c78957_g1_i1 | 204 | 68.0472 | 333.565 |
| c78957_g2_i1 | 398 | 121.157 | 304.415 |
| c78957_g3_i1 | 522 | 92.1127 | 176.461 |
| c79055_g1_i1 | 343 | 34.8534 | 101.614 |
| c79055_g1_i2 | 219 | 122.817 | 560.808 |
| c79113_g1_i2 | 259 | 48.9608 | 189.038 |
| c79115_g1_i1 | 335 | 54.7697 | 163.492 |
| c79309_g1_i3 | 1352 | 314.511 | 232.626 |
| c79309_g2_i1 | 675 | 202.482 | 299.973 |
| c79325_g1_i1 | 401 | 61.4084 | 153.138 |
| c79375_g1_i1 | 275 | 52.2802 | 190.11 |
| c79375_g1_i2 | 279 | 41.4922 | 148.718 |
| c79375_g1_i3 | 327 | 105.39 | 322.294 |
| c79388_g1_i1 | 558 | 58.0891 | 104.102 |
| c79402_g1_i1 | 459 | 72.1964 | 157.291 |
| c79410_g1_i1 | 875 | 94.6022 | 108.117 |
| c79410_g2_i2 | 540 | 117.838 | 218.218 |
| c79438_g1_i1 | 433 | 70.5367 | 162.902 |
| c79438_g2_i1 | 248 | 35.6833 | 143.884 |
| c79469_g1_i2 | 328 | 34.8534 | 106.26 |
| c79483_g1_i1 | 279 | 29.0445 | 104.102 |
| c79494_g1_i1 | 347 | 87.1336 | 251.105 |
| c79622_g1_i2 | 278 | 63.0681 | 226.864 |
| c79716_g1_i1 | 454 | 424.05 | 934.031 |
| c79768_g1_i2 | 1394 | 138.584 | 99.4146 |
| c79804_g1_i2 | 1022 | 120.327 | 117.737 |
| c79812_g1_i1 | 209 | 37.343 | 178.675 |
| c79812_g1_i2 | 227 | 43.1519 | 190.096 |
| c79812_g2_i1 | 271 | 37.343 | 137.797 |
| c79835_g1_i1 | 694 | 88.7933 | 127.944 |
| c79840_g1_i1 | 325 | 53.11 | 163.415 |
| c79870_g1_i1 | 407 | 48.1309 | 118.258 |
| c79882_g1_i1 | 421 | 58.9189 | 139.95 |
| c79897_g1_i1 | 700 | 146.053 | 208.646 |
| c79912_g1_i1 | 424 | 130.285 | 307.277 |
| c79924_g1_i2 | 401 | 44.8116 | 111.75 |
| c80039_g1_i1 | 381 | 1052.24 | 2761.79 |
| c80058_g1_i1 | 202 | 35.6833 | 176.65 |
| c80067_g1_i1 | 657 | 90.453 | 137.676 |
| c80096_g1_i1 | 580 | 74.6859 | 128.769 |
| c80128_g1_i1 | 327 | 91.2828 | 279.152 |
| c80128_g2_i1 | 393 | 142.733 | 363.189 |
| c80161_g1_i1 | 740 | 145.223 | 196.247 |
| c80275_g1_i1 | 286 | 37.343 | 130.57 |
| c80304_g1_i1 | 580 | 584.21 | 1007.26 |
| c80304_g2_i1 | 208 | 71.3666 | 343.109 |
| c80366_g3_i1 | 362 | 60.5786 | 167.344 |
| c80366_g3_i2 | 353 | 78.0053 | 220.978 |
| c80395_g1_i1 | 419 | 47.3011 | 112.89 |
| c80453_g1_i1 | 214 | 40.6623 | 190.011 |
| c80519_g2_i1 | 566 | 63.898 | 112.894 |
| c80628_g1_i1 | 423 | 249.783 | 590.504 |
| c80668_g1_i1 | 609 | 1184.19 | 1944.48 |
| c80684_g1_i1 | 385 | 42.322 | 109.927 |
| c80684_g2_i1 | 205 | 28.2147 | 137.633 |
| c80708_g1_i1 | 212 | 33.1938 | 156.574 |
| c80792_g1_i1 | 386 | 46.4713 | 120.392 |
| c80813_g1_i1 | 781 | 390.027 | 499.394 |
| c80825_g1_i1 | 854 | 307.042 | 359.534 |
| c80875_g1_i1 | 276 | 49.7906 | 180.401 |
| c80901_g2_i1 | 244 | 37.343 | 153.045 |
| c80927_g1_i1 | 588 | 177.587 | 302.018 |
| c81031_g1_i1 | 554 | 136.094 | 245.658 |
| c81031_g2_i1 | 347 | 89.6231 | 258.28 |
| c81052_g6_i1 | 548 | 55.5995 | 101.459 |
| c81066_g1_i1 | 434 | 165.139 | 380.504 |
| c81066_g1_i2 | 219 | 298.744 | 1364.13 |
| c81156_g1_i1 | 596 | 221.568 | 371.759 |
| c81191_g1_i1 | 1661 | 612.425 | 368.708 |
| c81258_g2_i1 | 250 | 48.1309 | 192.524 |
| c81258_g3_i1 | 284 | 141.903 | 499.659 |
| c81327_g3_i1 | 266 | 43.1519 | 162.225 |
| c81412_g1_i1 | 828 | 102.901 | 124.276 |
| c81441_g1_i1 | 332 | 81.3247 | 244.954 |
| c81463_g1_i1 | 1668 | 132.775 | 79.6013 |
| c81487_g2_i1 | 432 | 480.48 | 1112.22 |
| c81487_g3_i1 | 381 | 84.6441 | 222.163 |
| c81487_g4_i1 | 368 | 53.9398 | 146.576 |
| c81538_g2_i1 | 310 | 36.5131 | 117.784 |
| c81581_g1_i1 | 769 | 100.411 | 130.574 |
| c81581_g2_i1 | 287 | 99.5813 | 346.973 |
| c81606_g1_i1 | 232 | 26.555 | 114.461 |
| c81630_g2_i1 | 314 | 38.1728 | 121.569 |
| c81630_g4_i1 | 948 | 170.118 | 179.449 |
| c81726_g1_i1 | 236 | 77.1755 | 327.015 |
| c81800_g1_i2 | 511 | 70.5367 | 138.037 |
| c81800_g1_i3 | 374 | 116.178 | 310.637 |
| c81882_g1_i1 | 367 | 108.71 | 296.211 |
| c81882_g2_i1 | 746 | 74.6859 | 100.115 |
| c81884_g1_i1 | 461 | 128.626 | 279.015 |
| c81941_g2_i1 | 273 | 68.0472 | 249.257 |
| c81963_g1_i1 | 216 | 24.0655 | 111.414 |
| c81963_g2_i1 | 265 | 37.343 | 140.917 |
| c82027_g1_i1 | 404 | 63.898 | 158.163 |
| c82059_g1_i1 | 518 | 346.045 | 668.04 |
| c82084_g1_i2 | 498 | 88.7933 | 178.3 |
| c82139_g1_i1 | 483 | 78.0053 | 161.502 |
| c82189_g1_i1 | 440 | 185.055 | 420.58 |
| c82189_g2_i1 | 344 | 135.265 | 393.211 |
| c82205_g1_i1 | 295 | 53.11 | 180.034 |
| c82205_g2_i2 | 453 | 92.1127 | 203.339 |
| c82346_g1_i1 | 754 | 104.56 | 138.674 |
| c82388_g1_i1 | 201 | 75.5158 | 375.7 |
| c82448_g1_i1 | 612 | 93.7724 | 153.223 |
| c82470_g1_i1 | 610 | 69.7069 | 114.274 |
| c82540_g1_i1 | 401 | 84.6441 | 211.082 |
| c82650_g2_i1 | 239 | 29.0445 | 121.525 |
| c82701_g3_i2 | 476 | 83.8142 | 176.08 |
| c82732_g1_i1 | 296 | 250.613 | 846.665 |
| c82732_g3_i1 | 383 | 390.027 | 1018.35 |
| c82732_g4_i1 | 201 | 363.472 | 1808.32 |
| c82835_g1_i1 | 775 | 80.4949 | 103.864 |
| c82835_g2_i2 | 395 | 41.4922 | 105.044 |
| c82869_g1_i1 | 241 | 55.5995 | 230.703 |
| c82903_g1_i1 | 297 | 132.775 | 447.054 |
| c83005_g1_i1 | 290 | 43.1519 | 148.8 |
| c83048_g1_i1 | 422 | 58.0891 | 137.652 |
| c83050_g3_i1 | 213 | 59.7488 | 280.511 |
| c83107_g1_i1 | 2309 | 530.27 | 229.654 |
| c83214_g1_i1 | 511 | 187.545 | 367.015 |
| c83229_g1_i1 | 493 | 82.1545 | 166.642 |
| c83272_g1_i1 | 279 | 238.165 | 853.639 |
| c83272_g2_i1 | 213 | 29.0445 | 136.359 |
| c83272_g5_i1 | 217 | 73.0263 | 336.527 |
| c83272_g3_i2 | 886 | 165.969 | 187.324 |
| c83279_g1_i3 | 235 | 45.6414 | 194.219 |
| c83279_g3_i1 | 274 | 91.2828 | 333.149 |
| c83409_g3_i1 | 201 | 1200.78 | 5974.05 |
| c83622_g1_i1 | 551 | 87.1336 | 158.137 |
| c83623_g1_i1 | 296 | 56.4294 | 190.64 |
| c83648_g1_i2 | 1084 | 1120.29 | 1033.48 |
| c83744_g1_i2 | 459 | 168.458 | 367.012 |
| c83744_g2_i1 | 788 | 147.712 | 187.452 |
| c83867_g1_i1 | 463 | 130.285 | 281.394 |
| c83880_g1_i1 | 415 | 48.9608 | 117.978 |
| c83922_g3_i1 | 930 | 127.796 | 137.415 |
| c83940_g1_i1 | 314 | 53.11 | 169.14 |
| c83942_g1_i1 | 516 | 64.7278 | 125.442 |
| c83973_g1_i1 | 322 | 727.773 | 2260.16 |
| c83973_g1_i2 | 530 | 385.048 | 726.505 |
| c84061_g1_i1 | 461 | 97.0917 | 210.611 |
| c84081_g1_i1 | 987 | 1463.84 | 1483.13 |
| c84081_g1_i2 | 257 | 42.322 | 164.677 |
| c84081_g1_i3 | 223 | 33.1938 | 148.851 |
| c84347_g1_i1 | 384 | 61.4084 | 159.918 |
| c84414_g1_i1 | 333 | 43.1519 | 129.585 |
| c84451_g2_i1 | 918 | 721.964 | 786.453 |
| c84484_g1_i1 | 734 | 117.008 | 159.411 |
| c84499_g1_i1 | 905 | 134.435 | 148.547 |
| c84499_g3_i1 | 412 | 49.7906 | 120.851 |
| c84611_g1_i1 | 364 | 40.6623 | 111.71 |
| c84611_g2_i1 | 319 | 242.314 | 759.606 |
| c84611_g3_i1 | 674 | 150.202 | 222.851 |
| c84611_g5_i1 | 221 | 123.647 | 559.487 |
| c84622_g2_i1 | 329 | 36.5131 | 110.982 |
| c84661_g1_i1 | 281 | 83.8142 | 298.271 |
| c84835_g1_i2 | 619 | 68.0472 | 109.931 |
| c84835_g1_i3 | 486 | 307.042 | 631.774 |
| c84877_g2_i1 | 294 | 54.7697 | 186.291 |
| c84905_g1_i1 | 922 | 723.624 | 784.841 |
| c84938_g2_i1 | 251 | 62.2383 | 247.961 |
| c84949_g1_i1 | 412 | 49.7906 | 120.851 |
| c85031_g1_i1 | 1072 | 263.89 | 246.166 |
| c85046_g2_i1 | 613 | 93.7724 | 152.973 |
| c85123_g1_i3 | 510 | 111.199 | 218.037 |
| c85211_g1_i1 | 271 | 28.2147 | 104.113 |
| c85211_g1_i2 | 271 | 61.4084 | 226.599 |
| c85290_g1_i1 | 926 | 118.668 | 128.151 |
| c85297_g1_i1 | 250 | 89.6231 | 358.493 |
| c85304_g4_i1 | 281 | 211.61 | 753.061 |
| c85309_g1_i1 | 244 | 58.9189 | 241.471 |
| c85321_g1_i2 | 251 | 30.7042 | 122.328 |
| c85424_g1_i1 | 441 | 386.707 | 876.887 |
| c85492_g1_i2 | 1344 | 178.416 | 132.75 |
| c85649_g4_i1 | 418 | 68.0472 | 162.792 |
| c85699_g3_i1 | 216 | 68.0472 | 315.033 |
| c85705_g2_i1 | 567 | 62.2383 | 109.768 |
| c85773_g1_i1 | 203 | 55.5995 | 273.889 |
| c85789_g3_i1 | 280 | 43.1519 | 154.114 |
| c85806_g1_i1 | 1146 | 107.88 | 94.1359 |
| c86020_g2_i1 | 316 | 34.8534 | 110.296 |
| c86026_g1_i1 | 437 | 235.676 | 539.304 |
| c86026_g2_i1 | 312 | 70.5367 | 226.079 |
| c86029_g1_i1 | 633 | 211.61 | 334.297 |
| c86029_g2_i1 | 267 | 53.9398 | 202.022 |
| c86046_g3_i1 | 418 | 235.676 | 563.817 |
| c86141_g1_i1 | 253 | 28.2147 | 111.521 |
| c86154_g2_i1 | 530 | 75.5158 | 142.483 |
| c86242_g1_i3 | 411 | 73.0263 | 177.679 |
| c86242_g2_i1 | 360 | 127.796 | 354.989 |
| c86242_g2_i2 | 330 | 181.736 | 550.715 |
| c86389_g1_i1 | 499 | 250.613 | 502.23 |
| c86395_g1_i1 | 355 | 58.9189 | 165.969 |
| c86398_g2_i1 | 354 | 36.5131 | 103.144 |
| c86420_g1_i2 | 630 | 182.566 | 289.787 |
| c86469_g2_i1 | 669 | 99.5813 | 148.851 |
| c86485_g2_i1 | 412 | 259.741 | 630.44 |
| c86485_g3_i3 | 354 | 107.88 | 304.745 |
| c86485_g4_i1 | 229 | 253.102 | 1105.25 |
| c86511_g1_i1 | 226 | 29.0445 | 128.516 |
| c86557_g2_i2 | 384 | 59.7488 | 155.596 |
| c86571_g3_i1 | 470 | 64.7278 | 137.719 |
| c86575_g1_i1 | 309 | 65.5577 | 212.161 |
| c86610_g1_i1 | 209 | 63.898 | 305.732 |
| c86610_g4_i1 | 303 | 40.6623 | 134.199 |
| c86655_g1_i1 | 418 | 52.2802 | 125.072 |
| c86726_g1_i2 | 276 | 91.2828 | 330.735 |
| c86740_g1_i1 | 397 | 74.6859 | 188.126 |
| c86809_g1_i1 | 338 | 63.898 | 189.047 |
| c86809_g2_i1 | 513 | 52.2802 | 101.911 |
| c86809_g3_i1 | 337 | 68.0472 | 201.92 |
| c86860_g1_i1 | 2240 | 170.118 | 75.9455 |
| c86895_g2_i1 | 355 | 48.9608 | 137.918 |
| c86997_g3_i1 | 580 | 111.199 | 191.723 |
| c87085_g1_i1 | 235 | 31.5341 | 134.188 |
| c87085_g3_i1 | 326 | 39.8325 | 122.186 |
| c87098_g1_i1 | 646 | 97.0917 | 150.297 |
| c87115_g1_i1 | 460 | 46.4713 | 101.024 |
| c87115_g2_i1 | 718 | 361.812 | 503.916 |
| c87119_g1_i2 | 601 | 197.503 | 328.624 |
| c87249_g2_i1 | 271 | 28.2147 | 104.113 |
| c87289_g1_i1 | 730 | 240.655 | 329.664 |
| c87289_g2_i1 | 317 | 122.817 | 387.435 |
| c87289_g3_i1 | 922 | 331.938 | 360.019 |
| c87289_g3_i2 | 617 | 101.241 | 164.086 |
| c87289_g3_i3 | 1268 | 340.236 | 268.325 |
| c87294_g1_i1 | 1409 | 151.032 | 107.191 |
| c87356_g1_i1 | 684 | 83.8142 | 122.535 |
| c87437_g1_i1 | 305 | 102.901 | 337.379 |
| c87505_g3_i1 | 227 | 24.8953 | 109.671 |
| c87640_g2_i1 | 1239 | 133.605 | 107.833 |
| c87660_g1_i2 | 679 | 285.466 | 420.422 |
| c87712_g1_i2 | 852 | 90.453 | 106.165 |
| c87725_g2_i1 | 265 | 35.6833 | 134.654 |
| c87733_g2_i1 | 534 | 86.3038 | 161.618 |
| c87911_g1_i2 | 326 | 87.9634 | 269.827 |
| c87911_g2_i1 | 366 | 41.4922 | 113.367 |
| c87911_g2_i2 | 354 | 41.4922 | 117.21 |
| c87947_g1_i1 | 532 | 160.99 | 302.612 |
| c87947_g2_i1 | 534 | 326.958 | 612.282 |
| c87954_g1_i1 | 212 | 46.4713 | 219.204 |
| c87954_g2_i1 | 245 | 107.88 | 440.325 |
| c87998_g1_i1 | 477 | 48.1309 | 100.903 |
| c88127_g1_i1 | 731 | 268.04 | 366.675 |
| c88127_g3_i1 | 336 | 34.8534 | 103.73 |
| c88256_g1_i1 | 1463 | 187.545 | 128.192 |
| c88299_g1_i1 | 563 | 105.39 | 187.194 |
| c88314_g1_i1 | 1773 | 186.715 | 105.31 |
| c88314_g3_i1 | 215 | 71.3666 | 331.938 |
| c88473_g2_i1 | 247 | 81.3247 | 329.25 |
| c88473_g4_i1 | 248 | 32.3639 | 130.5 |
| c88507_g2_i1 | 275 | 43.9817 | 159.934 |
| c88508_g1_i1 | 299 | 37.343 | 124.893 |
| c88508_g3_i1 | 447 | 72.1964 | 161.513 |
| c88521_g1_i2 | 295 | 101.241 | 343.19 |
| c88549_g1_i1 | 213 | 38.1728 | 179.215 |
| c88712_g1_i1 | 273 | 112.029 | 410.362 |
| c88750_g1_i1 | 211 | 32.3639 | 153.383 |
| c88757_g1_i1 | 1956 | 203.312 | 103.943 |
| c88814_g1_i1 | 272 | 226.547 | 832.895 |
| c89020_g1_i1 | 211 | 25.7252 | 121.92 |
| c89131_g1_i1 | 278 | 112.029 | 402.982 |
| c89131_g2_i1 | 209 | 40.6623 | 194.557 |
| c89131_g5_i3 | 478 | 68.0472 | 142.358 |
| c89131_g3_i2 | 795 | 714.496 | 898.737 |
| c89147_g1_i1 | 385 | 82.9844 | 215.544 |
| c89147_g2_i1 | 296 | 52.2802 | 176.622 |
| c89255_g1_i1 | 318 | 69.7069 | 219.204 |
| c89264_g1_i2 | 473 | 125.306 | 264.918 |
| c89345_g1_i1 | 864 | 570.103 | 659.841 |
| c89517_g1_i1 | 364 | 51.4503 | 141.347 |
| c89769_g1_i1 | 351 | 35.6833 | 101.662 |
| c89769_g6_i1 | 684 | 89.6231 | 131.028 |
| c89769_g14_i1 | 539 | 99.5813 | 184.752 |
| c89929_g1_i1 | 747 | 78.8352 | 105.536 |
| c89938_g2_i1 | 403 | 44.8116 | 111.195 |
| c89938_g1_i2 | 856 | 351.024 | 410.075 |
| c89938_g3_i1 | 221 | 448.946 | 2031.43 |
| c89956_g1_i2 | 292 | 37.343 | 127.887 |
| c90010_g2_i1 | 317 | 38.1728 | 120.419 |
| c90016_g2_i3 | 1132 | 224.888 | 198.664 |
| c90034_g1_i1 | 340 | 39.8325 | 117.154 |
| c90057_g1_i1 | 299 | 40.6623 | 135.994 |
| c90057_g3_i2 | 596 | 59.7488 | 100.25 |
| c90057_g3_i3 | 999 | 249.783 | 250.033 |
| c90070_g2_i1 | 318 | 50.6205 | 159.184 |
| c90070_g3_i1 | 415 | 100.411 | 241.954 |
| c90070_g4_i1 | 276 | 53.11 | 192.428 |
| c90138_g2_i1 | 1240 | 229.867 | 185.376 |
| c90143_g1_i1 | 737 | 408.283 | 553.98 |
| c90359_g1_i1 | 682 | 327.788 | 480.628 |
| c90413_g1_i1 | 594 | 112.859 | 189.998 |
| c90434_g1_i1 | 580 | 116.178 | 200.307 |
| c90459_g1_i1 | 298 | 31.5341 | 105.819 |
| c90479_g3_i1 | 693 | 112.029 | 161.658 |
| c90479_g4_i1 | 204 | 26.555 | 130.172 |
| c90491_g3_i1 | 312 | 39.8325 | 127.668 |
| c90549_g1_i2 | 947 | 987.514 | 1042.78 |
| c90549_g1_i3 | 393 | 224.058 | 570.122 |
| c90549_g1_i4 | 466 | 91.2828 | 195.886 |
| c90559_g1_i1 | 307 | 94.6022 | 308.15 |
| c90642_g1_i1 | 512 | 406.623 | 794.186 |
| c90642_g2_i1 | 245 | 266.38 | 1087.26 |
| c90642_g3_i1 | 294 | 285.466 | 970.974 |
| c90708_g1_i1 | 436 | 54.7697 | 125.619 |
| c90787_g1_i1 | 587 | 154.351 | 262.949 |
| c90787_g1_i2 | 568 | 229.867 | 404.695 |
| c90787_g2_i1 | 592 | 205.801 | 347.637 |
| c90870_g2_i1 | 298 | 49.7906 | 167.083 |
| c90957_g5_i1 | 214 | 72.1964 | 337.366 |
| c91014_g4_i1 | 362 | 82.9844 | 229.239 |
| c91050_g4_i1 | 500 | 54.7697 | 109.539 |
| c91084_g1_i1 | 629 | 80.4949 | 127.973 |
| c91095_g1_i1 | 231 | 32.3639 | 140.104 |
| c91095_g2_i1 | 530 | 366.791 | 692.058 |
| c91095_g3_i1 | 1262 | 247.293 | 195.954 |
| c91096_g2_i2 | 285 | 31.5341 | 110.646 |
| c91118_g1_i1 | 437 | 73.0263 | 167.108 |
| c91139_g1_i1 | 1104 | 289.616 | 262.333 |
| c91166_g1_i1 | 891 | 182.566 | 204.9 |
| c91221_g1_i1 | 292 | 181.736 | 622.383 |
| c91221_g2_i1 | 233 | 53.11 | 227.94 |
| c91224_g8_i1 | 786 | 190.034 | 241.774 |
| c91274_g1_i1 | 365 | 36.5131 | 100.036 |
| c91387_g1_i1 | 203 | 49.7906 | 245.274 |
| c91451_g1_i1 | 225 | 24.0655 | 106.958 |
| c91451_g3_i1 | 340 | 44.8116 | 131.799 |
| c91487_g1_i1 | 607 | 92.1127 | 151.751 |
| c91487_g1_i2 | 706 | 144.393 | 204.522 |
| c91513_g2_i1 | 851 | 185.885 | 218.431 |
| c91712_g1_i3 | 479 | 52.2802 | 109.144 |
| c91712_g2_i1 | 375 | 49.7906 | 132.775 |
| c91760_g1_i1 | 420 | 359.322 | 855.529 |
| c91760_g2_i1 | 785 | 841.462 | 1071.93 |
| c91810_g1_i1 | 362 | 155.181 | 428.676 |
| c91810_g3_i1 | 396 | 248.953 | 628.67 |
| c91810_g4_i1 | 409 | 221.568 | 541.732 |
| c91810_g5_i1 | 304 | 138.584 | 455.868 |
| c91836_g1_i1 | 344 | 379.239 | 1102.44 |
| c91836_g3_i1 | 393 | 1019.88 | 2595.11 |
| c91846_g1_i1 | 1528 | 418.241 | 273.718 |
| c91846_g2_i1 | 1333 | 302.893 | 227.227 |
| c91846_g3_i1 | 539 | 76.3456 | 141.643 |
| c91905_g2_i1 | 500 | 131.115 | 262.231 |
| c91954_g1_i3 | 256 | 30.7042 | 119.938 |
| c91994_g1_i1 | 246 | 68.0472 | 276.615 |
| c92087_g1_i1 | 789 | 435.668 | 552.177 |
| c92110_g1_i1 | 206 | 49.7906 | 241.702 |
| c92110_g2_i1 | 449 | 246.464 | 548.917 |
| c92110_g3_i1 | 393 | 302.063 | 768.609 |
| c92110_g4_i1 | 721 | 343.555 | 476.498 |
| c92133_g2_i2 | 1085 | 118.668 | 109.371 |
| c92158_g2_i1 | 204 | 27.3848 | 134.239 |
| c92158_g7_i1 | 657 | 321.979 | 490.075 |
| c92171_g2_i1 | 1709 | 131.115 | 76.7205 |
| c92210_g1_i1 | 226 | 72.1964 | 319.453 |
| c92210_g2_i1 | 278 | 226.547 | 814.919 |
| c92210_g3_i1 | 383 | 204.971 | 535.173 |
| c92260_g1_i1 | 277 | 340.236 | 1228.29 |
| c92260_g2_i1 | 282 | 184.225 | 653.281 |
| c92260_g3_i1 | 312 | 301.233 | 965.491 |
| c92260_g4_i2 | 839 | 107.05 | 127.592 |
| c92260_g5_i1 | 508 | 165.969 | 326.71 |
| c92260_g5_i2 | 1374 | 1645.58 | 1197.66 |
| c92277_g1_i1 | 871 | 94.6022 | 108.613 |
| c92315_g2_i1 | 276 | 82.1545 | 297.661 |
| c92315_g3_i1 | 205 | 39.8325 | 194.305 |
| c92315_g3_i2 | 549 | 204.142 | 371.843 |
| c92350_g1_i1 | 229 | 40.6623 | 177.565 |
| c92470_g6_i1 | 299 | 43.9817 | 147.096 |
| c92502_g1_i1 | 1346 | 145.223 | 107.892 |
| c92566_g3_i1 | 223 | 24.0655 | 107.917 |
| c92621_g1_i1 | 246 | 58.0891 | 236.134 |
| c92621_g3_i2 | 230 | 28.2147 | 122.673 |
| c92626_g4_i1 | 238 | 75.5158 | 317.293 |
| c92626_g2_i2 | 441 | 50.6205 | 114.786 |
| c92637_g1_i4 | 407 | 68.0472 | 167.192 |
| c92637_g2_i1 | 392 | 155.181 | 395.869 |
| c92637_g1_i5 | 293 | 52.2802 | 178.431 |
| c92657_g3_i1 | 248 | 29.8744 | 120.461 |
| c92848_g1_i1 | 326 | 72.1964 | 221.461 |
| c92901_g1_i1 | 1273 | 197.503 | 155.148 |
| c92975_g2_i1 | 1206 | 298.744 | 247.715 |
| c93050_g1_i7 | 225 | 55.5995 | 247.109 |
| c93080_g1_i1 | 323 | 122.817 | 380.238 |
| c93080_g2_i1 | 270 | 150.202 | 556.303 |
| c93080_g3_i1 | 242 | 98.7514 | 408.064 |
| c93080_g5_i1 | 609 | 76.3456 | 125.362 |
| c93080_g6_i3 | 218 | 34.8534 | 159.878 |
| c93080_g8_i1 | 333 | 133.605 | 401.216 |
| c93080_g10_i1 | 468 | 87.1336 | 186.183 |
| c93125_g1_i1 | 413 | 63.0681 | 152.707 |
| c93125_g3_i1 | 570 | 63.898 | 112.102 |
| c93140_g2_i1 | 920 | 114.518 | 124.477 |
| c93140_g3_i1 | 1192 | 121.987 | 102.338 |
| c93212_g1_i1 | 373 | 160.16 | 429.383 |
| c93223_g2_i1 | 1695 | 186.715 | 110.156 |
| c93226_g3_i1 | 597 | 217.419 | 364.186 |
| c93226_g2_i2 | 445 | 112.859 | 253.615 |
| c93255_g2_i1 | 237 | 34.8534 | 147.061 |
| c93301_g5_i1 | 258 | 137.754 | 533.931 |
| c93301_g6_i1 | 322 | 92.1127 | 286.064 |
| c93307_g1_i1 | 280 | 366.791 | 1309.97 |
| c93307_g2_i1 | 1023 | 2442.23 | 2387.32 |
| c93307_g3_i1 | 253 | 419.071 | 1656.41 |
| c93313_g4_i1 | 207 | 69.7069 | 336.748 |
| c93313_g6_i1 | 201 | 65.5577 | 326.158 |
| c93313_g7_i1 | 227 | 33.1938 | 146.228 |
| c93313_g6_i4 | 426 | 819.056 | 1922.67 |
| c93313_g6_i5 | 502 | 78.0053 | 155.389 |
| c93313_g8_i1 | 552 | 65.5577 | 118.764 |
| c93313_g9_i1 | 477 | 1130.25 | 2369.49 |
| c93364_g2_i1 | 594 | 116.178 | 195.586 |
| c93411_g3_i6 | 1325 | 157.67 | 118.996 |
| c93453_g1_i1 | 373 | 55.5995 | 149.06 |
| c93487_g1_i1 | 382 | 277.998 | 727.743 |
| c93595_g1_i1 | 483 | 105.39 | 218.199 |
| c93598_g3_i1 | 280 | 45.6414 | 163.005 |
| c93627_g2_i1 | 2873 | 132.775 | 46.2148 |
| c93640_g2_i1 | 841 | 253.932 | 301.941 |
| c93644_g2_i1 | 1045 | 125.306 | 119.91 |
| c93644_g3_i1 | 445 | 53.11 | 119.348 |
| c93656_g1_i2 | 287 | 56.4294 | 196.618 |
| c93716_g1_i1 | 589 | 340.236 | 577.65 |
| c93753_g1_i1 | 398 | 180.076 | 452.453 |
| c93827_g1_i3 | 690 | 86.3038 | 125.078 |
| c93827_g2_i1 | 418 | 174.267 | 416.907 |
| c93831_g2_i1 | 223 | 35.6833 | 160.015 |
| c93927_g3_i1 | 604 | 152.691 | 252.8 |
| c93927_g7_i1 | 437 | 125.306 | 286.742 |
| c93927_g12_i1 | 511 | 220.738 | 431.974 |
| c93928_g1_i1 | 368 | 74.6859 | 202.951 |
| c93928_g1_i2 | 989 | 166.799 | 168.654 |
| c94002_g2_i1 | 613 | 141.903 | 231.49 |
| c94014_g3_i2 | 1123 | 117.838 | 104.931 |
| c94082_g1_i1 | 270 | 43.9817 | 162.895 |
| c94082_g1_i2 | 315 | 76.3456 | 242.367 |
| c94129_g1_i1 | 247 | 349.364 | 1414.43 |
| c94172_g1_i3 | 1975 | 128.626 | 65.127 |
| c94215_g1_i1 | 2146 | 1696.2 | 790.401 |
| c94215_g2_i2 | 691 | 269.699 | 390.303 |
| c94215_g3_i1 | 769 | 208.291 | 270.859 |
| c94215_g3_i2 | 969 | 321.979 | 332.28 |
| c94244_g1_i1 | 1579 | 194.183 | 122.979 |
| c94305_g2_i1 | 266 | 48.9608 | 184.063 |
| c94360_g6_i1 | 763 | 251.443 | 329.545 |
| c94360_g11_i1 | 241 | 50.6205 | 210.043 |
| c94459_g1_i2 | 335 | 138.584 | 413.683 |
| c94459_g1_i3 | 240 | 83.8142 | 349.226 |
| c94526_g1_i2 | 319 | 87.9634 | 275.747 |
| c94526_g1_i4 | 631 | 67.2174 | 106.525 |
| c94584_g12_i1 | 453 | 58.9189 | 130.064 |
| c94604_g1_i1 | 372 | 77.1755 | 207.461 |
| c94604_g3_i1 | 311 | 60.5786 | 194.786 |
| c94715_g1_i5 | 207 | 40.6623 | 196.436 |
| c94761_g2_i2 | 210 | 29.8744 | 142.259 |
| c94788_g2_i1 | 615 | 82.1545 | 133.585 |
| c94796_g5_i1 | 662 | 215.759 | 325.921 |
| c94830_g1_i1 | 285 | 41.4922 | 145.587 |
| c94850_g1_i1 | 629 | 85.4739 | 135.889 |
| c94850_g3_i1 | 307 | 53.9398 | 175.7 |
| c94850_g1_i2 | 1096 | 829.014 | 756.4 |
| c94850_g4_i1 | 479 | 134.435 | 280.657 |
| c94883_g1_i2 | 1645 | 198.333 | 120.567 |
| c94896_g2_i1 | 221 | 27.3848 | 123.913 |
| c94896_g7_i1 | 273 | 65.5577 | 240.138 |
| c94938_g2_i3 | 2442 | 102.071 | 41.798 |
| c94949_g6_i1 | 412 | 64.7278 | 157.106 |
| c94949_g5_i4 | 220 | 218.249 | 992.041 |
| c94949_g8_i1 | 500 | 383.388 | 766.776 |
| c95017_g2_i2 | 662 | 98.7514 | 149.171 |
| c95038_g1_i1 | 703 | 171.778 | 244.349 |
| c95045_g11_i1 | 306 | 92.9425 | 303.734 |
| c95103_g1_i1 | 646 | 237.335 | 367.392 |
| c95133_g1_i1 | 287 | 36.5131 | 127.223 |
| c95216_g2_i1 | 258 | 188.375 | 730.134 |
| c95250_g1_i1 | 464 | 73.8561 | 159.173 |
| c95307_g3_i1 | 638 | 1932.71 | 3029.32 |
| c95307_g4_i1 | 523 | 60.5786 | 115.829 |
| c95307_g6_i1 | 320 | 134.435 | 420.108 |
| c95307_g2_i3 | 1105 | 309.532 | 280.119 |
| c95307_g7_i1 | 210 | 241.485 | 1149.93 |
| c95307_g8_i1 | 225 | 54.7697 | 243.421 |
| c95307_g12_i1 | 351 | 784.202 | 2234.19 |
| c95352_g2_i1 | 1253 | 255.592 | 203.984 |
| c95358_g2_i1 | 454 | 80.4949 | 177.301 |
| c95417_g2_i3 | 716 | 77.1755 | 107.787 |
| c95423_g2_i1 | 601 | 336.917 | 560.593 |
| c95520_g3_i2 | 236 | 26.555 | 112.521 |
| c95524_g2_i1 | 440 | 204.971 | 465.844 |
| c95524_g1_i3 | 218 | 92.1127 | 422.535 |
| c95659_g1_i1 | 377 | 151.032 | 400.614 |
| c95696_g3_i1 | 275 | 30.7042 | 111.652 |
| c95739_g1_i4 | 551 | 64.7278 | 117.473 |
| c95739_g1_i5 | 339 | 57.2592 | 168.906 |
| c95739_g2_i1 | 1179 | 1555.13 | 1319.02 |
| c95739_g3_i3 | 237 | 72.1964 | 304.626 |
| c95739_g3_i5 | 1071 | 185.885 | 173.562 |
| c95739_g3_i6 | 288 | 73.0263 | 253.563 |
| c95747_g2_i1 | 2563 | 143.563 | 56.0136 |
| c95893_g2_i1 | 3192 | 111.199 | 34.8368 |
| c95933_g1_i1 | 226 | 35.6833 | 157.891 |
| c95933_g2_i2 | 332 | 105.39 | 317.44 |
| c95933_g4_i1 | 258 | 54.7697 | 212.286 |
| c95947_g2_i1 | 357 | 55.5995 | 155.741 |
| c95947_g3_i2 | 569 | 82.1545 | 144.384 |
| c96034_g1_i3 | 463 | 142.733 | 308.279 |
| c96034_g2_i1 | 483 | 66.3875 | 137.448 |
| c96042_g1_i1 | 409 | 145.223 | 355.068 |
| c96042_g2_i1 | 341 | 152.691 | 447.775 |
| c96042_g3_i1 | 244 | 36.5131 | 149.644 |
| c96042_g2_i2 | 711 | 886.273 | 1246.52 |
| c96042_g2_i3 | 238 | 51.4503 | 216.178 |
| c96042_g3_i2 | 578 | 290.445 | 502.501 |
| c96074_g1_i6 | 209 | 101.241 | 484.406 |
| c96087_g1_i1 | 1184 | 568.443 | 480.104 |
| c96088_g2_i1 | 292 | 32.3639 | 110.835 |
| c96100_g2_i1 | 261 | 40.6623 | 155.794 |
| c96169_g3_i1 | 263 | 45.6414 | 173.541 |
| c96207_g2_i1 | 214 | 38.1728 | 178.378 |
| c96213_g1_i1 | 558 | 1109.5 | 1988.35 |
| c96213_g3_i1 | 349 | 143.563 | 411.355 |
| c96213_g2_i3 | 987 | 193.354 | 195.9 |
| c96213_g4_i1 | 273 | 73.0263 | 267.495 |
| c96213_g5_i1 | 450 | 832.333 | 1849.63 |
| c96213_g6_i2 | 487 | 53.11 | 109.055 |
| c96213_g6_i3 | 323 | 39.8325 | 123.32 |
| c96218_g1_i2 | 604 | 92.9425 | 153.878 |
| c96218_g1_i3 | 233 | 26.555 | 113.97 |
| c96282_g1_i1 | 457 | 128.626 | 281.457 |
| c96282_g2_i1 | 226 | 32.3639 | 143.203 |
| c96282_g3_i1 | 1148 | 369.281 | 321.673 |
| c96282_g4_i1 | 370 | 48.9608 | 132.326 |
| c96282_g5_i1 | 792 | 112.029 | 141.451 |
| c96282_g8_i1 | 205 | 96.2619 | 469.57 |
| c96360_g2_i1 | 208 | 64.7278 | 311.191 |
| c96360_g2_i3 | 360 | 38.1728 | 106.036 |
| c96401_g1_i1 | 667 | 102.071 | 153.03 |
| c96413_g4_i1 | 218 | 26.555 | 121.812 |
| c96420_g1_i1 | 236 | 39.0027 | 165.266 |
| c96435_g1_i1 | 248 | 90.453 | 364.73 |
| c96435_g2_i1 | 293 | 148.542 | 506.969 |
| c96435_g1_i2 | 499 | 263.89 | 528.838 |
| c96500_g1_i6 | 379 | 113.689 | 299.97 |
| c96500_g4_i4 | 329 | 223.228 | 678.505 |
| c96500_g4_i7 | 871 | 130.285 | 149.581 |
| c96559_g2_i1 | 210 | 37.343 | 177.824 |
| c96567_g1_i2 | 246 | 126.966 | 516.122 |
| c96567_g1_i3 | 715 | 76.3456 | 106.777 |
| c96606_g4_i1 | 227 | 28.2147 | 124.294 |
| c96608_g1_i1 | 286 | 39.0027 | 136.373 |
| c96633_g1_i4 | 744 | 170.118 | 228.653 |
| c96633_g3_i2 | 586 | 83.8142 | 143.028 |
| c96633_g6_i1 | 244 | 38.1728 | 156.446 |
| c96665_g1_i1 | 317 | 72.1964 | 227.749 |
| c96665_g2_i1 | 957 | 530.27 | 554.096 |
| c96665_g3_i2 | 405 | 43.9817 | 108.597 |
| c96665_g6_i1 | 624 | 243.974 | 390.984 |
| c96665_g6_i2 | 433 | 121.157 | 279.809 |
| c96665_g7_i1 | 509 | 843.951 | 1658.06 |
| c96686_g4_i1 | 230 | 220.738 | 959.732 |
| c96714_g2_i2 | 648 | 200.822 | 309.911 |
| c96749_g6_i1 | 1126 | 100.411 | 89.175 |
| c96749_g13_i1 | 389 | 43.1519 | 110.93 |
| c96763_g1_i1 | 599 | 353.513 | 590.173 |
| c96763_g1_i2 | 222 | 33.1938 | 149.521 |
| c96763_g2_i1 | 563 | 326.958 | 580.743 |
| c96789_g1_i1 | 271 | 53.9398 | 199.04 |
| c96789_g1_i2 | 667 | 218.249 | 327.21 |
| c96900_g1_i1 | 220 | 46.4713 | 211.233 |
| c96900_g2_i1 | 258 | 324.469 | 1257.63 |
| c96900_g3_i1 | 498 | 245.634 | 493.241 |
| c96969_g2_i2 | 427 | 105.39 | 246.815 |
| c97016_g2_i1 | 243 | 43.9817 | 180.995 |
| c97016_g3_i1 | 234 | 25.7252 | 109.937 |
| c97025_g1_i2 | 993 | 446.456 | 449.603 |
| c97025_g2_i1 | 295 | 33.1938 | 112.521 |
| c97064_g2_i1 | 329 | 82.1545 | 249.71 |
| c97064_g4_i1 | 1388 | 238.995 | 172.187 |
| c97068_g3_i1 | 292 | 73.8561 | 252.932 |
| c97068_g4_i1 | 292 | 99.5813 | 341.032 |
| c97068_g3_i2 | 221 | 64.7278 | 292.886 |
| c97068_g5_i1 | 514 | 220.738 | 429.452 |
| c97079_g1_i1 | 1737 | 238.165 | 137.113 |
| c97079_g2_i1 | 476 | 303.723 | 638.073 |
| c97079_g8_i1 | 229 | 31.5341 | 137.703 |
| c97079_g8_i2 | 889 | 492.927 | 554.474 |
| c97079_g11_i1 | 273 | 198.333 | 726.493 |
| c97087_g1_i1 | 489 | 58.9189 | 120.489 |
| c97087_g2_i2 | 1243 | 104.56 | 84.1193 |
| c97135_g1_i1 | 901 | 1505.34 | 1670.74 |
| c97135_g1_i2 | 661 | 384.218 | 581.267 |
| c97135_g2_i1 | 239 | 77.1755 | 322.91 |
| c97159_g1_i1 | 2176 | 320.32 | 147.206 |
| c97160_g1_i1 | 752 | 520.312 | 691.904 |
| c97160_g2_i1 | 357 | 473.841 | 1327.29 |
| c97160_g4_i4 | 2034 | 208.291 | 102.405 |
| c97160_g5_i1 | 622 | 1062.2 | 1707.72 |
| c97160_g4_i6 | 549 | 118.668 | 216.152 |
| c97160_g6_i1 | 456 | 74.6859 | 163.785 |
| c97187_g1_i1 | 1041 | 590.849 | 567.578 |
| c97187_g1_i2 | 905 | 126.136 | 139.377 |
| c97187_g1_i3 | 1002 | 484.629 | 483.661 |
| c97197_g2_i1 | 377 | 106.22 | 281.751 |
| c97200_g1_i1 | 308 | 34.8534 | 113.161 |
| c97200_g3_i10 | 615 | 64.7278 | 105.248 |
| c97208_g2_i5 | 289 | 48.9608 | 169.414 |
| c97215_g9_i1 | 256 | 29.8744 | 116.697 |
| c97244_g1_i1 | 553 | 105.39 | 190.579 |
| c97244_g8_i3 | 1360 | 106.22 | 78.1029 |
| c97256_g1_i2 | 976 | 433.178 | 443.83 |
| c97256_g3_i1 | 384 | 42.322 | 110.214 |
| c97269_g2_i2 | 204 | 62.2383 | 305.09 |
| c97269_g1_i4 | 428 | 91.2828 | 213.278 |
| c97269_g5_i1 | 357 | 87.1336 | 244.072 |
| c97283_g2_i1 | 260 | 55.5995 | 213.844 |
| c97283_g1_i4 | 220 | 47.3011 | 215.005 |
| c97283_g2_i2 | 1007 | 487.118 | 483.732 |
| c97306_g2_i1 | 405 | 136.924 | 338.085 |
| c97320_g1_i1 | 354 | 187.545 | 529.787 |
| c97320_g3_i1 | 523 | 91.2828 | 174.537 |
| c97320_g1_i4 | 353 | 39.0027 | 110.489 |
| c97320_g3_i2 | 457 | 344.385 | 753.578 |
| c97321_g1_i6 | 248 | 27.3848 | 110.423 |
| c97324_g1_i2 | 231 | 27.3848 | 118.549 |
| c97324_g3_i1 | 259 | 129.456 | 499.829 |
| c97324_g3_i2 | 531 | 111.199 | 209.414 |
| c97324_g3_i3 | 459 | 697.069 | 1518.67 |
| c97324_g2_i2 | 747 | 216.589 | 289.945 |
| c97324_g3_i4 | 364 | 78.0053 | 214.3 |
| c97337_g2_i1 | 393 | 56.4294 | 143.586 |
| c97337_g3_i1 | 293 | 32.3639 | 110.457 |
| c97352_g3_i1 | 553 | 145.223 | 262.609 |
| c97352_g5_i1 | 368 | 55.5995 | 151.086 |
| c97352_g5_i3 | 1540 | 643.129 | 417.616 |
| c97352_g7_i1 | 452 | 357.663 | 791.289 |
| c97352_g8_i1 | 286 | 294.595 | 1030.05 |
| c97352_g10_i3 | 441 | 158.5 | 359.411 |
| c97394_g3_i1 | 322 | 35.6833 | 110.818 |
| c97394_g4_i1 | 230 | 79.665 | 346.37 |
| c97394_g4_i2 | 212 | 90.453 | 426.665 |
| c97394_g3_i3 | 891 | 666.365 | 747.884 |
| c97394_g4_i4 | 1979 | 1603.26 | 810.136 |
| c97405_g1_i1 | 422 | 316.171 | 749.219 |
| c97405_g2_i1 | 373 | 353.513 | 947.757 |
| c97405_g3_i1 | 838 | 414.922 | 495.134 |
| c97405_g4_i1 | 201 | 258.081 | 1283.99 |
| c97405_g5_i1 | 1014 | 345.215 | 340.449 |
| c97405_g6_i1 | 496 | 140.244 | 282.749 |
| c97405_g7_i1 | 396 | 124.477 | 314.335 |
| c97405_g8_i1 | 260 | 26.555 | 102.135 |
| c97405_g10_i1 | 373 | 190.034 | 509.475 |
| c97405_g9_i2 | 670 | 270.529 | 403.775 |
| c97406_g2_i1 | 248 | 78.0053 | 314.538 |
| c97406_g3_i1 | 1092 | 162.649 | 148.946 |
| c97406_g4_i1 | 966 | 150.202 | 155.488 |
| c97406_g5_i1 | 395 | 117.838 | 298.324 |
| c97406_g6_i1 | 451 | 98.7514 | 218.961 |
| c97446_g3_i1 | 1189 | 133.605 | 112.367 |
| c97463_g6_i1 | 202 | 47.3011 | 234.164 |
| c97464_g2_i1 | 285 | 39.0027 | 136.851 |
| c97479_g1_i1 | 385 | 165.139 | 428.932 |
| c97538_g3_i2 | 318 | 32.3639 | 101.773 |
| c97581_g1_i1 | 324 | 195.843 | 604.454 |
| c97581_g2_i1 | 389 | 266.38 | 684.781 |
| c97587_g1_i1 | 352 | 190.864 | 542.228 |
| c97587_g2_i1 | 565 | 329.448 | 583.094 |
| c97587_g3_i1 | 537 | 71.3666 | 132.899 |
| c97587_g3_i2 | 333 | 222.398 | 667.862 |
| c97613_g2_i1 | 278 | 29.0445 | 104.477 |
| c97629_g1_i2 | 1050 | 141.903 | 135.146 |
| c97639_g2_i1 | 329 | 182.566 | 554.911 |
| c97639_g3_i1 | 417 | 52.2802 | 125.372 |
| c97639_g4_i1 | 1289 | 194.183 | 150.647 |
| c97650_g1_i1 | 345 | 157.67 | 457.015 |
| c97650_g2_i1 | 2279 | 199.163 | 87.3903 |
| c97679_g6_i1 | 227 | 29.0445 | 127.949 |
| c97682_g6_i1 | 201 | 24.0655 | 119.729 |
| c97715_g3_i1 | 247 | 71.3666 | 288.933 |
| c97715_g2_i4 | 278 | 28.2147 | 101.492 |
| c97715_g2_i5 | 772 | 323.639 | 419.222 |
| c97715_g6_i1 | 300 | 83.8142 | 279.381 |
| c97715_g6_i3 | 784 | 127.796 | 163.005 |
| c97715_g6_i4 | 249 | 121.157 | 486.575 |
| c97727_g2_i1 | 370 | 83.8142 | 226.525 |
| c97738_g2_i3 | 289 | 58.9189 | 203.872 |
| c97740_g2_i11 | 665 | 132.775 | 199.662 |
| c97740_g2_i12 | 267 | 54.7697 | 205.13 |
| c97740_g5_i1 | 256 | 62.2383 | 243.118 |
| c97748_g4_i1 | 259 | 43.1519 | 166.61 |
| c97750_g1_i1 | 3354 | 258.911 | 77.1948 |
| c97750_g2_i1 | 1179 | 224.888 | 190.744 |
| c97750_g2_i2 | 814 | 580.891 | 713.625 |
| c97750_g2_i3 | 201 | 24.0655 | 119.729 |
| c97775_g2_i1 | 539 | 68.877 | 127.787 |
| c97780_g2_i1 | 731 | 101.241 | 138.497 |
| c97798_g1_i1 | 1933 | 566.783 | 293.214 |
| c97798_g4_i1 | 722 | 207.461 | 287.342 |
| c97798_g5_i1 | 769 | 270.529 | 351.793 |
| c97830_g2_i1 | 285 | 63.898 | 224.203 |
| c97830_g4_i1 | 239 | 39.8325 | 166.663 |
| c97831_g1_i1 | 201 | 96.2619 | 478.915 |
| c97872_g4_i2 | 875 | 386.707 | 441.951 |
| c97872_g1_i2 | 204 | 44.8116 | 219.665 |
| c97919_g3_i2 | 591 | 279.657 | 473.194 |
| c97919_g4_i1 | 481 | 400.815 | 833.294 |
| c97919_g5_i2 | 812 | 87.1336 | 107.307 |
| c97919_g6_i1 | 204 | 231.526 | 1134.93 |
| c97965_g2_i5 | 899 | 131.945 | 146.769 |
| c97977_g1_i1 | 284 | 85.4739 | 300.964 |
| c98026_g1_i1 | 234 | 34.0236 | 145.4 |
| c98026_g5_i2 | 804 | 145.223 | 180.625 |
| c98048_g1_i1 | 329 | 107.05 | 325.379 |
| c98048_g5_i1 | 336 | 191.694 | 570.518 |
| c98074_g5_i4 | 267 | 34.8534 | 130.537 |
| c98100_g2_i2 | 303 | 199.163 | 657.302 |
| c98100_g4_i1 | 209 | 175.097 | 837.785 |
| c98100_g5_i1 | 655 | 801.629 | 1223.86 |
| c98100_g3_i2 | 485 | 107.88 | 222.432 |
| c98100_g6_i4 | 396 | 84.6441 | 213.748 |
| c98115_g3_i2 | 353 | 43.1519 | 122.243 |
| c98129_g6_i3 | 331 | 36.5131 | 110.312 |
| c98135_g3_i1 | 553 | 72.1964 | 130.554 |
| c98139_g3_i3 | 465 | 53.11 | 114.215 |
| c98176_g1_i2 | 4475 | 108.71 | 24.2926 |
| c98192_g3_i1 | 480 | 55.5995 | 115.832 |
| c98224_g2_i1 | 353 | 38.1728 | 108.138 |
| c98224_g6_i1 | 269 | 53.9398 | 200.52 |
| c98232_g4_i2 | 4806 | 225.718 | 46.9658 |
| c98306_g1_i3 | 873 | 92.1127 | 105.513 |
| c98336_g1_i1 | 325 | 56.4294 | 173.629 |
| c98336_g2_i3 | 415 | 92.9425 | 223.958 |
| c98369_g1_i1 | 239 | 68.0472 | 284.716 |
| c98369_g2_i1 | 314 | 71.3666 | 227.282 |
| c98369_g6_i1 | 543 | 79.665 | 146.713 |
| c98369_g2_i2 | 337 | 34.8534 | 103.423 |
| c98369_g9_i1 | 350 | 94.6022 | 270.292 |
| c98383_g7_i1 | 732 | 129.456 | 176.852 |
| c98405_g3_i1 | 208 | 54.7697 | 263.316 |
| c98405_g2_i5 | 252 | 62.2383 | 246.977 |
| c98424_g1_i1 | 2129 | 629.022 | 295.454 |
| c98431_g6_i1 | 271 | 92.9425 | 342.961 |
| c98441_g1_i1 | 365 | 124.477 | 341.032 |
| c98441_g2_i1 | 656 | 72.1964 | 110.056 |
| c98441_g2_i2 | 1699 | 419.901 | 247.146 |
| c98517_g2_i1 | 390 | 155.181 | 397.899 |
| c98557_g2_i1 | 1425 | 112.859 | 79.1991 |
| c98562_g1_i2 | 958 | 97.9216 | 102.215 |
| c98562_g3_i1 | 207 | 134.435 | 649.443 |
| c98569_g1_i2 | 240 | 41.4922 | 172.884 |
| c98569_g2_i1 | 461 | 58.0891 | 126.007 |
| c98579_g3_i1 | 382 | 46.4713 | 121.653 |
| c98596_g1_i1 | 802 | 147.712 | 184.18 |
| c98616_g1_i1 | 272 | 34.8534 | 128.138 |
| c98617_g3_i1 | 1090 | 136.924 | 125.619 |
| c98627_g5_i1 | 392 | 63.898 | 163.005 |
| c98632_g3_i1 | 302 | 121.157 | 401.183 |
| c98636_g1_i1 | 645 | 336.917 | 522.351 |
| c98636_g3_i1 | 362 | 2272.94 | 6278.85 |
| c98636_g4_i1 | 372 | 53.11 | 142.769 |
| c98636_g5_i1 | 386 | 796.65 | 2063.86 |
| c98636_g6_i1 | 428 | 290.445 | 678.611 |
| c98636_g6_i2 | 399 | 220.738 | 553.229 |
| c98636_g8_i1 | 419 | 659.726 | 1574.52 |
| c98636_g8_i2 | 367 | 429.859 | 1171.28 |
| c98636_g7_i2 | 595 | 1355.96 | 2278.93 |
| c98636_g9_i2 | 386 | 458.904 | 1188.87 |
| c98636_g10_i1 | 443 | 158.5 | 357.788 |
| c98650_g6_i1 | 363 | 82.9844 | 228.607 |
| c98662_g1_i4 | 307 | 99.5813 | 324.369 |
| c98671_g1_i1 | 226 | 246.464 | 1090.55 |
| c98671_g2_i1 | 685 | 619.064 | 903.742 |
| c98671_g3_i1 | 424 | 393.346 | 927.703 |
| c98671_g2_i2 | 369 | 108.71 | 294.606 |
| c98671_g5_i1 | 430 | 673.833 | 1567.05 |
| c98671_g4_i2 | 565 | 103.73 | 183.594 |
| c98671_g6_i1 | 221 | 494.587 | 2237.95 |
| c98671_g3_i2 | 212 | 32.3639 | 152.66 |
| c98671_g7_i1 | 292 | 1106.18 | 3788.29 |
| c98671_g8_i1 | 271 | 30.7042 | 113.3 |
| c98673_g2_i2 | 391 | 53.9398 | 137.954 |
| c98674_g2_i1 | 837 | 286.296 | 342.05 |
| c98674_g2_i6 | 1594 | 1082.95 | 679.389 |
| c98699_g2_i1 | 307 | 46.4713 | 151.372 |
| c98717_g2_i1 | 471 | 129.456 | 274.853 |
| c98717_g3_i1 | 309 | 34.8534 | 112.794 |
| c98741_g1_i1 | 423 | 102.071 | 241.302 |
| c98741_g2_i1 | 284 | 39.8325 | 140.255 |
| c98741_g1_i2 | 496 | 197.503 | 398.191 |
| c98741_g4_i1 | 211 | 58.0891 | 275.304 |
| c98741_g4_i2 | 208 | 26.555 | 127.668 |
| c98761_g3_i1 | 467 | 63.898 | 136.827 |
| c98765_g2_i2 | 469 | 78.0053 | 166.323 |
| c98765_g2_i3 | 283 | 40.6623 | 143.683 |
| c98765_g2_i4 | 597 | 107.88 | 180.703 |
| c98765_g4_i1 | 603 | 770.095 | 1277.11 |
| c98765_g5_i1 | 216 | 121.157 | 560.913 |
| c98778_g2_i1 | 254 | 187.545 | 738.365 |
| c98778_g3_i1 | 1551 | 858.888 | 553.764 |
| c98778_g4_i2 | 251 | 27.3848 | 109.103 |
| c98814_g1_i1 | 242 | 223.228 | 922.43 |
| c98814_g2_i1 | 592 | 92.1127 | 155.596 |
| c98814_g2_i2 | 223 | 99.5813 | 446.553 |
| c98832_g1_i1 | 339 | 235.676 | 695.208 |
| c98832_g4_i2 | 1133 | 176.757 | 156.008 |
| c98832_g4_i3 | 1322 | 110.369 | 83.4866 |
| c98832_g5_i1 | 359 | 138.584 | 386.028 |
| c98847_g2_i1 | 692 | 118.668 | 171.485 |
| c98847_g2_i2 | 246 | 31.5341 | 128.187 |
| c98871_g1_i1 | 204 | 28.2147 | 138.307 |
| c98884_g2_i1 | 1064 | 197.503 | 185.623 |
| c98889_g1_i2 | 1813 | 267.21 | 147.385 |
| c98910_g8_i1 | 438 | 62.2383 | 142.097 |
| c98929_g5_i1 | 579 | 370.11 | 639.223 |
| c98929_g5_i4 | 417 | 258.081 | 618.9 |
| c98949_g11_i1 | 213 | 25.7252 | 120.775 |
| c99024_g2_i2 | 516 | 52.2802 | 101.318 |
| c99024_g3_i1 | 286 | 86.3038 | 301.761 |
| c99024_g5_i1 | 218 | 24.0655 | 110.392 |
| c99051_g6_i1 | 253 | 325.299 | 1285.77 |
| c99051_g6_i2 | 288 | 38.1728 | 132.545 |
| c99053_g3_i1 | 214 | 43.9817 | 205.522 |
| c99064_g4_i1 | 449 | 269.699 | 600.666 |
| c99064_g5_i1 | 228 | 39.0027 | 171.064 |
| c99068_g3_i3 | 1359 | 224.888 | 165.48 |
| c99079_g5_i1 | 241 | 45.6414 | 189.383 |
| c99129_g2_i1 | 325 | 359.322 | 1105.61 |
| c99129_g3_i5 | 1183 | 170.118 | 143.802 |
| c99129_g8_i1 | 397 | 146.882 | 369.981 |
| c99138_g3_i2 | 219 | 107.05 | 488.812 |
| c99138_g6_i3 | 648 | 258.081 | 398.274 |
| c99144_g1_i1 | 902 | 165.969 | 184.001 |
| c99149_g1_i1 | 497 | 63.898 | 128.567 |
| c99149_g2_i1 | 284 | 30.7042 | 108.113 |
| c99165_g6_i1 | 235 | 44.8116 | 190.688 |
| c99165_g7_i1 | 365 | 91.2828 | 250.09 |
| c99176_g1_i1 | 533 | 64.7278 | 121.441 |
| c99176_g2_i1 | 239 | 25.7252 | 107.637 |
| c99176_g3_i1 | 515 | 404.964 | 786.337 |
| c99190_g2_i3 | 858 | 448.116 | 522.279 |
| c99238_g1_i2 | 1943 | 140.244 | 72.1789 |
| c99254_g3_i1 | 742 | 93.7724 | 126.378 |
| c99258_g1_i1 | 419 | 268.04 | 639.713 |
| c99258_g2_i3 | 363 | 62.2383 | 171.455 |
| c99258_g2_i4 | 413 | 90.453 | 219.014 |
| c99303_g3_i1 | 261 | 44.8116 | 171.692 |
| c99362_g3_i1 | 2587 | 123.647 | 47.7954 |
| c99364_g3_i1 | 677 | 249.783 | 368.956 |
| c99368_g1_i4 | 293 | 170.948 | 583.44 |
| c99368_g1_i6 | 945 | 710.346 | 751.689 |
| c99376_g1_i2 | 441 | 102.901 | 233.335 |
| c99380_g1_i1 | 3854 | 198.333 | 51.4615 |
| c99381_g1_i1 | 256 | 35.6833 | 139.388 |
| c99406_g2_i3 | 2142 | 247.293 | 115.45 |
| c99414_g1_i1 | 686 | 122.817 | 179.033 |
| c99414_g4_i1 | 1045 | 152.691 | 146.116 |
| c99418_g1_i1 | 420 | 56.4294 | 134.356 |
| c99418_g1_i2 | 425 | 47.3011 | 111.297 |
| c99418_g2_i1 | 307 | 138.584 | 451.413 |
| c99458_g1_i3 | 237 | 27.3848 | 115.548 |
| c99458_g3_i1 | 291 | 78.0053 | 268.06 |
| c99488_g5_i1 | 279 | 42.322 | 151.692 |
| c99496_g5_i1 | 484 | 132.775 | 274.329 |
| c99498_g1_i3 | 919 | 112.859 | 122.806 |
| c99507_g2_i2 | 394 | 68.0472 | 172.709 |
| c99523_g2_i2 | 242 | 39.0027 | 161.168 |
| c99564_g1_i1 | 369 | 307.872 | 834.342 |
| c99564_g4_i1 | 254 | 30.7042 | 120.883 |
| c99581_g4_i1 | 720 | 101.241 | 140.612 |
| c99581_g7_i1 | 874 | 297.914 | 340.863 |
| c99585_g1_i2 | 858 | 171.778 | 200.207 |
| c99601_g2_i3 | 243 | 35.6833 | 146.845 |
| c99639_g1_i1 | 206 | 58.9189 | 286.014 |
| c99670_g3_i1 | 440 | 185.055 | 420.58 |
| c99675_g3_i1 | 1179 | 112.029 | 95.0203 |
| c99675_g3_i2 | 961 | 105.39 | 109.667 |
| c99685_g3_i1 | 349 | 62.2383 | 178.333 |
| c99707_g3_i2 | 272 | 35.6833 | 131.189 |
| c99719_g2_i1 | 206 | 26.555 | 128.908 |
| c99721_g5_i5 | 272 | 28.2147 | 103.73 |
| c99760_g2_i1 | 292 | 60.5786 | 207.461 |
| c99775_g2_i1 | 2285 | 395.006 | 172.869 |
| c99775_g2_i2 | 643 | 78.8352 | 122.605 |
| c99775_g4_i1 | 287 | 110.369 | 384.562 |
| c99775_g5_i1 | 338 | 36.5131 | 108.027 |
| c99788_g3_i1 | 552 | 94.6022 | 171.381 |
| c99788_g5_i1 | 1600 | 173.437 | 108.398 |
| c99792_g1_i1 | 215 | 121.987 | 567.382 |
| c99792_g2_i1 | 252 | 388.367 | 1541.14 |
| c99792_g4_i1 | 354 | 513.673 | 1451.05 |
| c99802_g1_i1 | 302 | 816.566 | 2703.86 |
| c99802_g2_i1 | 366 | 110.369 | 301.555 |
| c99802_g2_i2 | 1375 | 503.715 | 366.338 |
| c99802_g2_i3 | 2178 | 669.684 | 307.477 |
| c99802_g2_i5 | 1188 | 1082.95 | 911.571 |
| c99815_g1_i1 | 1243 | 144.393 | 116.165 |
| c99832_g2_i1 | 698 | 190.864 | 273.444 |
| c99832_g2_i2 | 393 | 409.943 | 1043.11 |
| c99832_g4_i1 | 316 | 424.88 | 1344.56 |
| c99839_g3_i1 | 1032 | 155.181 | 150.369 |
| c99885_g1_i1 | 661 | 251.443 | 380.397 |
| c99895_g3_i1 | 238 | 47.3011 | 198.744 |
| c99895_g5_i1 | 447 | 78.0053 | 174.509 |
| c99911_g5_i1 | 221 | 124.477 | 563.242 |
| c99920_g4_i1 | 312 | 96.2619 | 308.532 |
| c99959_g1_i1 | 333 | 55.5995 | 166.966 |
| c99959_g7_i1 | 246 | 32.3639 | 131.561 |
| c99959_g6_i3 | 2299 | 167.628 | 72.9136 |
| c99959_g6_i4 | 1789 | 126.966 | 70.9704 |
| c99959_g8_i1 | 248 | 68.877 | 277.73 |
| c99962_g1_i1 | 208 | 23.2356 | 111.71 |
| c99962_g3_i1 | 1521 | 164.309 | 108.027 |
| c99962_g4_i1 | 1264 | 109.539 | 86.6609 |
| c99983_g5_i1 | 643 | 67.2174 | 104.537 |
| c99996_g2_i1 | 278 | 186.715 | 671.636 |
| c100001_g5_i1 | 298 | 54.7697 | 183.791 |
| c100008_g4_i1 | 356 | 50.6205 | 142.192 |
| c100036_g2_i2 | 545 | 81.3247 | 149.22 |
| c100073_g3_i1 | 228 | 119.498 | 524.112 |
| c100073_g4_i1 | 572 | 109.539 | 191.502 |
| c100073_g2_i4 | 567 | 155.181 | 273.687 |
| c100073_g5_i1 | 205 | 58.0891 | 283.361 |
| c100073_g5_i2 | 397 | 171.778 | 432.689 |
| c100073_g6_i1 | 229 | 52.2802 | 228.298 |
| c100073_g7_i1 | 378 | 171.778 | 454.438 |
| c100109_g1_i2 | 715 | 113.689 | 159.005 |
| c100109_g3_i1 | 211 | 175.097 | 829.844 |
| c100110_g2_i5 | 453 | 70.5367 | 155.71 |
| c100124_g2_i1 | 290 | 72.1964 | 248.953 |
| c100129_g1_i2 | 330 | 695.409 | 2107.3 |
| c100129_g2_i1 | 363 | 317 | 873.279 |
| c100129_g3_i1 | 358 | 166.799 | 465.918 |
| c100129_g3_i2 | 318 | 85.4739 | 268.786 |
| c100129_g4_i1 | 921 | 1033.99 | 1122.68 |
| c100129_g5_i1 | 387 | 258.081 | 666.877 |
| c100132_g1_i1 | 369 | 317.83 | 861.328 |
| c100143_g1_i2 | 4113 | 136.924 | 33.2906 |
| c100143_g2_i1 | 409 | 46.4713 | 113.622 |
| c100143_g3_i1 | 690 | 115.348 | 167.171 |
| c100149_g4_i1 | 318 | 34.8534 | 109.602 |
| c100156_g3_i1 | 485 | 84.6441 | 174.524 |
| c100158_g2_i1 | 2238 | 102.071 | 45.608 |
| c100158_g2_i2 | 681 | 81.3247 | 119.42 |
| c100158_g3_i1 | 413 | 126.136 | 305.415 |
| c100158_g6_i1 | 285 | 35.6833 | 125.205 |
| c100158_g7_i1 | 360 | 85.4739 | 237.428 |
| c100172_g3_i1 | 223 | 29.0445 | 130.245 |
| c100172_g6_i1 | 582 | 87.9634 | 151.14 |
| c100179_g1_i7 | 960 | 161.82 | 168.562 |
| c100184_g3_i1 | 224 | 34.8534 | 155.596 |
| c100226_g1_i2 | 223 | 101.241 | 453.995 |
| c100226_g2_i1 | 276 | 206.631 | 748.663 |
| c100226_g3_i1 | 489 | 77.1755 | 157.823 |
| c100226_g1_i4 | 261 | 136.094 | 521.434 |
| c100226_g1_i5 | 375 | 197.503 | 526.674 |
| c100226_g4_i1 | 1076 | 531.93 | 494.359 |
| c100226_g6_i1 | 207 | 122.817 | 593.318 |
| c100228_g2_i1 | 1119 | 165.969 | 148.319 |
| c100228_g2_i3 | 486 | 97.0917 | 199.777 |
| c100230_g2_i1 | 334 | 83.8142 | 250.941 |
| c100230_g1_i2 | 309 | 34.0236 | 110.109 |
| c100238_g4_i1 | 437 | 48.9608 | 112.038 |
| c100242_g2_i2 | 678 | 73.0263 | 107.708 |
| c100253_g2_i2 | 225 | 48.9608 | 217.603 |
| c100253_g3_i1 | 249 | 77.1755 | 309.942 |
| c100253_g2_i5 | 251 | 39.8325 | 158.695 |
| c100253_g2_i6 | 416 | 43.9817 | 105.725 |
| c100255_g1_i1 | 251 | 58.0891 | 231.431 |
| c100255_g4_i1 | 1264 | 219.079 | 173.322 |
| c100255_g10_i1 | 242 | 25.7252 | 106.302 |
| c100261_g2_i1 | 1036 | 121.987 | 117.748 |
| c100261_g2_i2 | 230 | 58.0891 | 252.561 |
| c100261_g2_i3 | 592 | 105.39 | 178.024 |
| c100261_g3_i1 | 232 | 29.0445 | 125.192 |
| c100261_g4_i1 | 228 | 52.2802 | 229.299 |
| c100261_g3_i2 | 212 | 59.7488 | 281.834 |
| c100271_g2_i1 | 218 | 22.4058 | 102.779 |
| c100284_g3_i2 | 1385 | 1114.48 | 804.679 |
| c100320_g4_i1 | 1597 | 135.265 | 84.6992 |
| c100326_g1_i1 | 317 | 77.1755 | 243.456 |
| c100326_g2_i1 | 307 | 129.456 | 421.68 |
| c100326_g3_i1 | 223 | 28.2147 | 126.523 |
| c100326_g4_i1 | 261 | 38.1728 | 146.256 |
| c100326_g6_i1 | 585 | 71.3666 | 121.994 |
| c100326_g6_i2 | 379 | 160.99 | 424.775 |
| c100326_g3_i2 | 244 | 36.5131 | 149.644 |
| c100326_g9_i1 | 504 | 83.8142 | 166.298 |
| c100327_g3_i2 | 343 | 47.3011 | 137.904 |
| c100327_g4_i1 | 535 | 570.103 | 1065.61 |
| c100327_g2_i3 | 649 | 82.9844 | 127.865 |
| c100327_g5_i1 | 285 | 295.424 | 1036.58 |
| c100335_g2_i1 | 457 | 194.183 | 424.909 |
| c100342_g2_i1 | 266 | 161.82 | 608.344 |
| c100363_g9_i1 | 1127 | 183.395 | 162.729 |
| c100364_g1_i1 | 366 | 429.859 | 1174.48 |
| c100364_g2_i1 | 1025 | 424.88 | 414.517 |
| c100364_g3_i1 | 279 | 614.914 | 2203.99 |
| c100364_g4_i1 | 243 | 518.652 | 2134.37 |
| c100364_g2_i3 | 936 | 100.411 | 107.277 |
| c100364_g5_i1 | 382 | 732.752 | 1918.2 |
| c100364_g6_i1 | 364 | 139.414 | 383.005 |
| c100365_g1_i1 | 434 | 61.4084 | 141.494 |
| c100365_g1_i3 | 464 | 58.0891 | 125.192 |
| c100385_g1_i2 | 1422 | 180.906 | 127.219 |
| c100394_g1_i9 | 8281 | 103.73 | 12.5263 |
| c100403_g2_i1 | 1365 | 455.584 | 333.761 |
| c100403_g2_i2 | 945 | 1210.74 | 1281.21 |
| c100403_g7_i1 | 285 | 121.157 | 425.113 |
| c100434_g2_i1 | 1485 | 421.561 | 283.879 |
| c100434_g3_i1 | 814 | 107.88 | 132.53 |
| c100436_g5_i1 | 253 | 48.9608 | 193.521 |
| c100442_g2_i3 | 385 | 78.0053 | 202.611 |
| c100443_g1_i4 | 205 | 79.665 | 388.61 |
| c100449_g3_i2 | 248 | 170.118 | 685.96 |
| c100455_g1_i1 | 257 | 96.2619 | 374.56 |
| c100455_g2_i2 | 879 | 171.778 | 195.424 |
| c100455_g3_i1 | 223 | 105.39 | 472.602 |
| c100458_g6_i1 | 206 | 39.0027 | 189.333 |
| c100474_g1_i1 | 924 | 165.969 | 179.62 |
| c100474_g3_i1 | 242 | 32.3639 | 133.735 |
| c100475_g3_i1 | 1670 | 235.676 | 141.123 |
| c100475_g3_i2 | 679 | 126.966 | 186.99 |
| c100485_g1_i1 | 361 | 70.5367 | 195.393 |
| c100485_g1_i2 | 1011 | 365.961 | 361.979 |
| c100485_g1_i3 | 357 | 41.4922 | 116.225 |
| c100485_g1_i4 | 265 | 39.0027 | 147.18 |
| c100492_g2_i1 | 282 | 31.5341 | 111.823 |
| c100499_g1_i1 | 236 | 28.2147 | 119.554 |
| c100499_g2_i1 | 428 | 235.676 | 550.644 |
| c100499_g3_i1 | 1536 | 145.223 | 94.546 |
| c100499_g2_i3 | 967 | 1789.97 | 1851.06 |
| c100499_g2_i5 | 842 | 293.765 | 348.889 |
| c100499_g4_i1 | 622 | 196.673 | 316.195 |
| c100501_g1_i1 | 3507 | 4386.55 | 1250.8 |
| c100502_g1_i1 | 2664 | 284.636 | 106.846 |
| c100505_g4_i1 | 5420 | 136.924 | 25.2628 |
| c100508_g1_i1 | 268 | 63.0681 | 235.329 |
| c100508_g2_i1 | 625 | 64.7278 | 103.565 |
| c100508_g4_i1 | 604 | 153.521 | 254.174 |
| c100513_g1_i1 | 739 | 442.307 | 598.521 |
| c100522_g1_i4 | 2561 | 129.456 | 50.5489 |
| c100527_g2_i1 | 345 | 69.7069 | 202.049 |
| c100527_g3_i1 | 234 | 157.67 | 673.805 |
| c100528_g2_i1 | 235 | 24.0655 | 102.406 |
| c100528_g5_i1 | 293 | 117.838 | 402.177 |
| c100529_g1_i2 | 278 | 72.1964 | 259.699 |
| c100529_g2_i1 | 1580 | 346.875 | 219.541 |
| c100529_g1_i4 | 7798 | 216.589 | 27.775 |
| c100531_g1_i2 | 291 | 58.9189 | 202.47 |
| c100532_g5_i1 | 238 | 260.571 | 1094.84 |
| c100536_g1_i2 | 320 | 71.3666 | 223.021 |
| c100543_g1_i2 | 6008 | 575.912 | 95.8575 |
| c100550_g1_i1 | 6285 | 20331.2 | 3234.87 |
| c100550_g1_i3 | 293 | 53.9398 | 184.095 |
| c100551_g2_i1 | 244 | 93.7724 | 384.313 |
| c100551_g3_i2 | 410 | 42.322 | 103.224 |
| c100553_g2_i1 | 348 | 68.877 | 197.923 |
| c100563_g1_i1 | 254 | 168.458 | 663.222 |
| c100582_g1_i1 | 308 | 116.178 | 377.202 |
| c100585_g1_i1 | 372 | 868.017 | 2333.38 |
| c100597_g1_i1 | 258 | 91.2828 | 353.809 |
| c100641_g1_i1 | 242 | 28.2147 | 116.59 |
| c100664_g1_i1 | 372 | 37.343 | 100.384 |
| c100704_g1_i1 | 232 | 23.2356 | 100.154 |
| c100711_g1_i1 | 253 | 29.8744 | 118.081 |
| c100765_g1_i1 | 287 | 56.4294 | 196.618 |
| c100784_g1_i1 | 365 | 138.584 | 379.682 |
| c100844_g1_i1 | 204 | 29.8744 | 146.443 |
| c100867_g1_i1 | 208 | 28.2147 | 135.648 |
| c100870_g1_i1 | 267 | 52.2802 | 195.806 |
| c100873_g1_i1 | 305 | 358.493 | 1175.39 |
| c100887_g1_i1 | 317 | 123.647 | 390.053 |
| c100968_g1_i1 | 349 | 40.6623 | 116.511 |
| c101033_g1_i1 | 234 | 34.8534 | 148.946 |
| c101059_g1_i1 | 224 | 53.9398 | 240.803 |
| c101098_g1_i1 | 208 | 91.2828 | 438.86 |
| c101108_g1_i1 | 367 | 72.1964 | 196.72 |
| c101140_g1_i1 | 300 | 67.2174 | 224.058 |
| c101143_g1_i1 | 235 | 64.7278 | 275.438 |
| c101222_g1_i1 | 327 | 38.1728 | 116.736 |
| c101232_g1_i1 | 241 | 189.204 | 785.08 |
| c101249_g1_i1 | 277 | 63.898 | 230.679 |
| c101293_g1_i1 | 205 | 119.498 | 582.915 |
| c101349_g1_i1 | 263 | 81.3247 | 309.219 |
| c101356_g1_i1 | 545 | 97.0917 | 178.15 |
| c101450_g1_i1 | 203 | 24.0655 | 118.549 |
| c101456_g1_i1 | 501 | 52.2802 | 104.352 |
| c101493_g1_i1 | 269 | 62.2383 | 231.369 |
| c101522_g1_i1 | 237 | 34.8534 | 147.061 |
| c101523_g1_i1 | 205 | 33.1938 | 161.921 |
| c101530_g1_i1 | 371 | 72.1964 | 194.599 |
| c101540_g1_i1 | 240 | 39.0027 | 162.511 |
| c101576_g1_i1 | 207 | 40.6623 | 196.436 |
| c101640_g1_i1 | 265 | 40.6623 | 153.443 |
| c101682_g1_i1 | 534 | 92.1127 | 172.496 |
| c101762_g1_i1 | 246 | 78.8352 | 320.468 |
| c101765_g1_i1 | 385 | 83.8142 | 217.699 |
| c101827_g1_i1 | 264 | 60.5786 | 229.464 |
| c101834_g1_i1 | 204 | 204.971 | 1004.76 |
| c101888_g1_i1 | 219 | 62.2383 | 284.193 |
| c101903_g1_i1 | 224 | 26.555 | 118.549 |
| c101946_g1_i1 | 273 | 32.3639 | 118.549 |
| c101954_g1_i1 | 351 | 63.0681 | 179.681 |
| c101960_g1_i1 | 248 | 73.8561 | 297.807 |
| c102053_g1_i1 | 201 | 48.9608 | 243.586 |
| c102114_g1_i1 | 543 | 70.5367 | 129.902 |
| c102171_g1_i1 | 222 | 49.7906 | 224.282 |
| c102194_g1_i1 | 219 | 39.8325 | 181.884 |
| c102204_g1_i1 | 255 | 102.901 | 403.532 |
| c102205_g1_i1 | 202 | 44.8116 | 221.839 |
| c102222_g1_i1 | 217 | 24.8953 | 114.725 |
| c102253_g1_i1 | 213 | 21.5759 | 101.295 |
| c102290_g1_i1 | 209 | 21.5759 | 103.234 |
| c102323_g1_i1 | 252 | 82.9844 | 329.303 |
| c102339_g1_i1 | 224 | 31.5341 | 140.777 |
| c102340_g1_i1 | 306 | 43.1519 | 141.019 |
| c102395_g1_i1 | 222 | 31.5341 | 142.045 |
| c102427_g1_i1 | 243 | 31.5341 | 129.77 |
| c102504_g1_i1 | 208 | 34.0236 | 163.575 |
| c102521_g1_i1 | 212 | 80.4949 | 379.693 |
| c102529_g1_i1 | 225 | 31.5341 | 140.151 |
| c102539_g1_i1 | 279 | 39.0027 | 139.794 |
| c102573_g1_i1 | 415 | 168.458 | 405.924 |
| c102642_g1_i1 | 382 | 138.584 | 362.785 |
| c102660_g1_i1 | 202 | 20.7461 | 102.703 |
| c102668_g1_i1 | 270 | 39.0027 | 144.454 |
| c102778_g1_i1 | 257 | 59.7488 | 232.485 |
| c102854_g1_i1 | 229 | 88.7933 | 387.744 |
| c102873_g1_i1 | 221 | 27.3848 | 123.913 |
| c102887_g1_i1 | 212 | 87.1336 | 411.008 |
| c102918_g1_i1 | 266 | 40.6623 | 152.866 |
| c102950_g1_i1 | 242 | 40.6623 | 168.026 |
| c103029_g1_i1 | 204 | 30.7042 | 150.511 |
| c103041_g1_i1 | 252 | 75.5158 | 299.666 |
| c103063_g1_i1 | 266 | 44.8116 | 168.465 |
| c103091_g1_i1 | 322 | 238.995 | 742.221 |
| c103147_g1_i1 | 262 | 53.11 | 202.71 |
| c103216_g1_i1 | 226 | 101.241 | 447.969 |
| c103230_g1_i1 | 323 | 54.7697 | 169.566 |
| c103262_g1_i1 | 251 | 58.0891 | 231.431 |
| c103369_g1_i1 | 374 | 96.2619 | 257.385 |
| c103370_g1_i1 | 223 | 175.927 | 788.91 |
| c103393_g1_i1 | 351 | 833.993 | 2376.05 |
| c103416_g1_i1 | 227 | 37.343 | 164.506 |
| c103472_g1_i1 | 210 | 32.3639 | 154.114 |
| c103503_g1_i1 | 204 | 26.555 | 130.172 |
| c103504_g1_i1 | 312 | 53.9398 | 172.884 |
| c103516_g1_i1 | 279 | 94.6022 | 339.076 |
| c103527_g1_i1 | 257 | 121.157 | 471.429 |
| c103543_g1_i1 | 215 | 25.7252 | 119.652 |
| c103562_g1_i1 | 216 | 43.1519 | 199.777 |
| c103579_g1_i1 | 300 | 34.8534 | 116.178 |
| c103585_g1_i1 | 205 | 27.3848 | 133.585 |
| c103649_g1_i1 | 267 | 30.7042 | 114.997 |
| c103690_g1_i1 | 326 | 80.4949 | 246.917 |
| c103700_g1_i1 | 236 | 29.8744 | 126.586 |
| c103703_g1_i1 | 312 | 41.4922 | 132.988 |
| c103705_g1_i1 | 202 | 20.7461 | 102.703 |
| c103712_g1_i1 | 205 | 29.0445 | 141.681 |
| c103723_g1_i1 | 263 | 59.7488 | 227.182 |
| c103731_g1_i1 | 263 | 39.8325 | 151.454 |
| c103788_g1_i1 | 218 | 126.966 | 582.413 |
| c103898_g1_i1 | 330 | 100.411 | 304.276 |
| c103982_g1_i1 | 224 | 24.8953 | 111.14 |
| c104004_g1_i1 | 266 | 42.322 | 159.105 |
| c104013_g1_i1 | 290 | 45.6414 | 157.384 |
| c104073_g1_i1 | 251 | 29.8744 | 119.021 |
| c104099_g1_i1 | 232 | 65.5577 | 282.576 |
| c104105_g1_i1 | 215 | 59.7488 | 277.901 |
| c104220_g1_i1 | 211 | 39.0027 | 184.847 |
| c104270_g1_i1 | 202 | 29.8744 | 147.893 |
| c104378_g1_i1 | 209 | 58.0891 | 277.938 |
| c104445_g1_i1 | 234 | 32.3639 | 138.307 |
| c104536_g1_i1 | 241 | 29.8744 | 123.96 |
| c104621_g1_i1 | 221 | 31.5341 | 142.688 |
| c104670_g1_i1 | 221 | 33.1938 | 150.198 |
| c104686_g1_i1 | 205 | 54.7697 | 267.169 |
| c104810_g1_i1 | 257 | 38.1728 | 148.532 |
| c104816_g1_i1 | 225 | 85.4739 | 379.884 |
| c104907_g1_i1 | 249 | 144.393 | 579.891 |
| c104925_g1_i1 | 428 | 48.1309 | 112.455 |
| c104926_g1_i1 | 322 | 81.3247 | 252.561 |
| c104941_g1_i1 | 325 | 65.5577 | 201.716 |
| c104951_g1_i1 | 396 | 40.6623 | 102.683 |
| c104967_g1_i1 | 297 | 31.5341 | 106.175 |
| c104978_g1_i1 | 314 | 58.0891 | 184.997 |
| c104981_g1_i1 | 241 | 46.4713 | 192.827 |
| c105012_g1_i1 | 258 | 109.539 | 424.571 |
| c105022_g1_i1 | 240 | 27.3848 | 114.104 |
| c105106_g1_i1 | 328 | 135.265 | 412.392 |
| c105120_g1_i1 | 766 | 117.008 | 152.752 |
| c105127_g1_i1 | 203 | 107.88 | 531.427 |
| c105133_g1_i1 | 260 | 36.5131 | 140.435 |
| c105199_g1_i1 | 286 | 71.3666 | 249.533 |
| c105203_g1_i1 | 315 | 87.1336 | 276.615 |
| c105236_g1_i1 | 225 | 42.322 | 188.098 |
| c105299_g1_i1 | 265 | 68.0472 | 256.782 |
| c105307_g1_i1 | 478 | 424.05 | 887.134 |
| c105314_g1_i1 | 261 | 26.555 | 101.743 |
| c105321_g1_i1 | 387 | 73.0263 | 188.698 |
| c105472_g1_i1 | 603 | 73.8561 | 122.481 |
| c105473_g1_i1 | 263 | 55.5995 | 211.405 |
| c105552_g1_i1 | 217 | 22.4058 | 103.252 |
| c105570_g1_i1 | 411 | 165.139 | 401.798 |
| c105578_g1_i1 | 443 | 140.244 | 316.577 |
| c105727_g1_i1 | 246 | 58.0891 | 236.134 |
| c105773_g1_i1 | 201 | 102.071 | 507.815 |
| c105797_g1_i1 | 288 | 76.3456 | 265.089 |
| c105816_g1_i1 | 677 | 256.422 | 378.762 |
| c105899_g1_i1 | 311 | 224.888 | 723.112 |
| c105917_g1_i1 | 281 | 69.7069 | 248.067 |
| c105947_g1_i1 | 249 | 107.88 | 433.252 |
| c105954_g1_i1 | 240 | 24.0655 | 100.273 |
| c105988_g1_i1 | 204 | 63.898 | 313.225 |
| c105998_g1_i1 | 210 | 47.3011 | 225.243 |
| c106026_g1_i1 | 238 | 81.3247 | 341.7 |
| c106073_g1_i1 | 409 | 181.736 | 444.342 |
| c106095_g1_i1 | 361 | 43.9817 | 121.833 |
| c106132_g1_i1 | 253 | 46.4713 | 183.681 |
| c106179_g1_i1 | 215 | 87.9634 | 409.132 |
| c106289_g1_i1 | 330 | 37.343 | 113.161 |
| c106305_g1_i1 | 276 | 29.0445 | 105.234 |
| c106390_g1_i1 | 242 | 172.608 | 713.254 |
| c106393_g1_i1 | 244 | 153.521 | 629.185 |
| c106482_g1_i1 | 210 | 38.1728 | 181.775 |
| c106491_g1_i1 | 322 | 39.8325 | 123.703 |
| c106536_g1_i1 | 302 | 65.5577 | 217.078 |
| c106538_g1_i1 | 730 | 78.0053 | 106.857 |
| c106656_g1_i1 | 211 | 99.5813 | 471.949 |
| c106679_g1_i1 | 229 | 26.555 | 115.961 |
| c106689_g1_i1 | 206 | 63.898 | 310.184 |
| c106704_g1_i1 | 328 | 34.0236 | 103.73 |
| c106716_g1_i1 | 225 | 50.6205 | 224.98 |
| c106755_g1_i1 | 228 | 204.971 | 898.997 |
| c106760_g1_i1 | 214 | 29.0445 | 135.722 |
| c106842_g1_i1 | 316 | 122.817 | 388.661 |
| c106870_g1_i1 | 289 | 139.414 | 482.401 |
| c106935_g1_i1 | 202 | 25.7252 | 127.352 |
| c106939_g1_i1 | 313 | 63.0681 | 201.496 |
| c106957_g1_i1 | 386 | 66.3875 | 171.988 |
| c107107_g1_i1 | 232 | 34.8534 | 150.23 |
| c107112_g1_i1 | 203 | 22.4058 | 110.373 |
| c107148_g1_i1 | 254 | 26.555 | 104.547 |
| c107170_g1_i1 | 246 | 25.7252 | 104.574 |
| c107223_g1_i1 | 220 | 122.817 | 558.259 |
| c107287_g1_i1 | 250 | 107.88 | 431.519 |
| c107292_g1_i1 | 374 | 162.649 | 434.891 |
| c107344_g1_i1 | 463 | 55.5995 | 120.085 |
| c107358_g1_i1 | 287 | 94.6022 | 329.624 |
| c107391_g1_i1 | 303 | 58.0891 | 191.713 |
| c107399_g1_i1 | 213 | 55.5995 | 261.031 |
| c107405_g1_i1 | 207 | 29.8744 | 144.321 |
| c107417_g1_i1 | 249 | 29.8744 | 119.977 |
| c107439_g1_i1 | 207 | 82.1545 | 396.882 |
| c107449_g1_i1 | 229 | 28.2147 | 123.208 |
| c107452_g1_i1 | 216 | 136.094 | 630.067 |
| c107453_g1_i1 | 344 | 43.1519 | 125.442 |
| c107509_g1_i1 | 282 | 100.411 | 356.068 |
| c107513_g1_i1 | 217 | 79.665 | 367.12 |
| c107534_g1_i1 | 318 | 103.73 | 326.196 |
| c107589_g1_i1 | 228 | 24.0655 | 105.55 |
| c107603_g1_i1 | 203 | 22.4058 | 110.373 |
| c107730_g1_i1 | 328 | 124.477 | 379.502 |
| c107745_g1_i1 | 257 | 31.5341 | 122.701 |
| c107758_g1_i1 | 260 | 49.7906 | 191.502 |
| c107796_g1_i1 | 266 | 131.115 | 492.915 |
| c107827_g1_i1 | 212 | 37.343 | 176.146 |
| c107916_g1_i1 | 401 | 104.56 | 260.749 |
| c107941_g1_i1 | 207 | 24.8953 | 120.267 |
| c107943_g1_i1 | 208 | 60.5786 | 291.243 |
| c107965_g1_i1 | 291 | 52.2802 | 179.657 |
| c108013_g1_i1 | 253 | 41.4922 | 164.001 |
| c108074_g1_i1 | 348 | 433.178 | 1244.77 |
| c108162_g1_i1 | 206 | 68.877 | 334.355 |
| c108190_g1_i1 | 240 | 68.877 | 286.988 |
| c108198_g1_i1 | 203 | 20.7461 | 102.198 |
| c108394_g1_i1 | 324 | 36.5131 | 112.695 |
| c108454_g1_i1 | 216 | 80.4949 | 372.661 |
| c108466_g1_i1 | 232 | 68.0472 | 293.307 |
| c108501_g1_i1 | 207 | 108.71 | 525.167 |
| c108520_g1_i1 | 204 | 28.2147 | 138.307 |
| c108533_g1_i1 | 275 | 36.5131 | 132.775 |
| c108623_g1_i1 | 258 | 34.8534 | 135.091 |
| c108625_g1_i1 | 224 | 45.6414 | 203.756 |
| c108627_g1_i1 | 343 | 138.584 | 404.035 |
| c108682_g1_i1 | 270 | 30.7042 | 113.719 |
| c108736_g1_i1 | 275 | 49.7906 | 181.057 |
| c108845_g1_i1 | 238 | 40.6623 | 170.85 |
| c108958_g1_i1 | 237 | 91.2828 | 385.16 |
| c109057_g1_i1 | 567 | 95.432 | 168.31 |
| c109082_g1_i1 | 404 | 192.524 | 476.544 |
| c109097_g1_i1 | 292 | 34.8534 | 119.361 |
| c109108_g1_i1 | 428 | 414.092 | 967.505 |
| c109177_g1_i1 | 287 | 112.029 | 390.345 |
| c109228_g1_i1 | 373 | 58.9189 | 157.96 |
| c109328_g1_i1 | 507 | 69.7069 | 137.489 |
| c109366_g1_i1 | 414 | 47.3011 | 114.254 |
| c109390_g1_i1 | 444 | 238.165 | 536.408 |
| c109453_g1_i1 | 492 | 73.8561 | 150.114 |
| c109555_g1_i1 | 271 | 69.7069 | 257.221 |
| c109594_g1_i1 | 203 | 57.2592 | 282.065 |
| c109603_g1_i1 | 214 | 65.5577 | 306.344 |
| c109622_g1_i1 | 361 | 39.0027 | 108.041 |
| c109645_g1_i1 | 383 | 93.7724 | 244.836 |
| c109677_g1_i1 | 259 | 29.8744 | 115.345 |
| c109700_g1_i1 | 226 | 41.4922 | 183.594 |
| c109708_g1_i1 | 227 | 89.6231 | 394.816 |
| c109710_g1_i1 | 323 | 58.0891 | 179.842 |
| c109784_g1_i1 | 292 | 194.183 | 665.012 |
| c109796_g1_i1 | 226 | 82.1545 | 363.516 |
| c109813_g1_i1 | 206 | 27.3848 | 132.936 |
| c109821_g1_i1 | 487 | 248.953 | 511.197 |
| c109886_g1_i1 | 225 | 57.2592 | 254.485 |
| c109894_g1_i1 | 210 | 89.6231 | 426.777 |
| c109912_g1_i1 | 871 | 385.877 | 443.028 |
| c109939_g1_i1 | 236 | 89.6231 | 379.759 |
| c109971_g1_i1 | 278 | 29.8744 | 107.462 |
| c109983_g1_i1 | 209 | 43.9817 | 210.439 |
| c110017_g1_i1 | 211 | 77.1755 | 365.761 |
| c110139_g1_i1 | 297 | 45.6414 | 153.675 |
| c110146_g1_i1 | 208 | 53.11 | 255.337 |
| c110156_g1_i1 | 210 | 22.4058 | 106.694 |
| c110243_g1_i1 | 357 | 234.016 | 655.507 |
| c110399_g1_i1 | 211 | 165.139 | 782.649 |
| c110408_g1_i1 | 260 | 214.93 | 826.652 |
| c110435_g1_i1 | 323 | 73.0263 | 226.087 |
| c110436_g1_i1 | 211 | 28.2147 | 133.719 |
| c110446_g1_i1 | 276 | 29.0445 | 105.234 |
| c110464_g1_i1 | 496 | 52.2802 | 105.404 |
| c110476_g1_i1 | 239 | 39.8325 | 166.663 |
| c110500_g1_i1 | 222 | 23.2356 | 104.665 |
| c110516_g1_i1 | 236 | 40.6623 | 172.298 |
| c110554_g1_i1 | 345 | 39.0027 | 113.051 |
| c110597_g1_i1 | 218 | 24.0655 | 110.392 |
| c110661_g1_i1 | 328 | 54.7697 | 166.981 |
| c110672_g1_i1 | 359 | 68.0472 | 189.547 |
| c110684_g1_i1 | 373 | 44.8116 | 120.138 |
| c110692_g1_i1 | 206 | 23.2356 | 112.794 |
| c110702_g1_i1 | 262 | 32.3639 | 123.526 |
| c110765_g1_i1 | 313 | 114.518 | 365.874 |
| c110803_g1_i1 | 294 | 41.4922 | 141.13 |
| c110812_g1_i1 | 354 | 82.1545 | 232.075 |
| c110822_g1_i1 | 244 | 68.877 | 282.283 |
| c110849_g1_i1 | 207 | 22.4058 | 108.241 |
| c110866_g1_i1 | 221 | 73.0263 | 330.436 |
| c110873_g1_i1 | 466 | 271.359 | 582.315 |
| c110919_g1_i1 | 387 | 138.584 | 358.098 |
| c110920_g1_i1 | 259 | 33.1938 | 128.161 |
| c110937_g1_i1 | 474 | 69.7069 | 147.061 |
| c110950_g1_i1 | 253 | 69.7069 | 275.521 |
| c111013_g1_i1 | 351 | 43.1519 | 122.94 |
| c111016_g1_i1 | 218 | 40.6623 | 186.525 |
| c111069_g1_i1 | 241 | 90.453 | 375.324 |
| c111086_g1_i1 | 268 | 42.322 | 157.918 |
| c111151_g1_i1 | 233 | 149.372 | 641.081 |
| c111188_g1_i1 | 209 | 63.0681 | 301.761 |
| c111240_g1_i1 | 247 | 273.019 | 1105.34 |
| c111273_g1_i1 | 239 | 34.0236 | 142.358 |
| c111287_g1_i1 | 512 | 66.3875 | 129.663 |
| c111342_g1_i1 | 366 | 374.26 | 1022.57 |
| c111349_g1_i1 | 368 | 65.5577 | 178.146 |
| c111390_g1_i1 | 202 | 39.0027 | 193.082 |
| c111441_g1_i1 | 261 | 34.8534 | 133.538 |
| c111479_g1_i1 | 332 | 117.838 | 354.933 |
| c111493_g1_i1 | 231 | 48.1309 | 208.359 |
| c111548_g1_i1 | 243 | 36.5131 | 150.26 |
| c111590_g1_i1 | 201 | 22.4058 | 111.472 |
| c111611_g1_i1 | 226 | 125.306 | 554.453 |
| c111614_g1_i1 | 497 | 101.241 | 203.704 |
| c111643_g1_i1 | 221 | 50.6205 | 229.052 |
| c111676_g1_i1 | 253 | 41.4922 | 164.001 |
| c111794_g1_i1 | 240 | 38.1728 | 159.053 |
| c111795_g1_i1 | 212 | 22.4058 | 105.688 |
| c111826_g1_i1 | 208 | 102.901 | 494.715 |
| c111904_g1_i1 | 205 | 86.3038 | 420.994 |
| c111929_g1_i1 | 249 | 43.1519 | 173.301 |
| c111934_g1_i1 | 226 | 32.3639 | 143.203 |
| c111952_g1_i1 | 208 | 48.9608 | 235.388 |
| c111974_g1_i1 | 251 | 64.7278 | 257.88 |
| c112006_g1_i1 | 255 | 71.3666 | 279.869 |
| c112059_g1_i1 | 272 | 83.8142 | 308.141 |
| c112097_g1_i1 | 235 | 33.1938 | 141.25 |
| c112160_g1_i1 | 251 | 49.7906 | 198.369 |
| c112239_g1_i1 | 221 | 71.3666 | 322.926 |
| c112266_g1_i1 | 257 | 99.5813 | 387.476 |
| c112296_g1_i1 | 278 | 165.139 | 594.025 |
| c112513_g1_i1 | 205 | 47.3011 | 230.737 |
| c112526_g1_i1 | 291 | 74.6859 | 256.653 |
| c112539_g1_i1 | 202 | 29.8744 | 147.893 |
| c112659_g1_i1 | 238 | 26.555 | 111.576 |
| c112664_g1_i1 | 211 | 79.665 | 377.559 |
| c112684_g1_i1 | 253 | 31.5341 | 124.641 |
| c112819_g1_i1 | 206 | 23.2356 | 112.794 |
| c112829_g1_i1 | 233 | 102.901 | 441.634 |
| c112875_g1_i1 | 235 | 78.8352 | 335.469 |
| c113064_g1_i1 | 204 | 82.9844 | 406.786 |
| c113071_g1_i1 | 207 | 38.1728 | 184.41 |
| c113087_g1_i1 | 202 | 33.1938 | 164.326 |
| c113170_g1_i1 | 285 | 31.5341 | 110.646 |
| c113212_g1_i1 | 507 | 99.5813 | 196.413 |
| c113226_g1_i1 | 280 | 142.733 | 509.761 |
| c113310_g1_i1 | 316 | 33.1938 | 105.044 |
| c113317_g1_i1 | 226 | 26.555 | 117.5 |
| c113344_g1_i1 | 270 | 52.2802 | 193.63 |
| c113367_g1_i1 | 204 | 86.3038 | 423.058 |
| c113377_g1_i1 | 335 | 68.877 | 205.603 |
| c113498_g1_i1 | 252 | 25.7252 | 102.084 |
| c113514_g1_i1 | 227 | 89.6231 | 394.816 |
| c113541_g1_i1 | 240 | 116.178 | 484.076 |
| c113624_g1_i1 | 225 | 24.8953 | 110.646 |
| c113664_g1_i1 | 201 | 115.348 | 573.872 |
| c113709_g1_i1 | 213 | 24.0655 | 112.983 |
| c113711_g1_i1 | 312 | 36.5131 | 117.029 |
| c113882_g1_i1 | 371 | 47.3011 | 127.496 |
| c113910_g1_i1 | 209 | 106.22 | 508.23 |
| c113939_g1_i1 | 219 | 46.4713 | 212.198 |
| c113941_g1_i1 | 216 | 135.265 | 626.225 |
| c113956_g1_i1 | 305 | 112.859 | 370.029 |
| c113965_g1_i1 | 265 | 74.6859 | 281.834 |
| c113985_g1_i1 | 243 | 42.322 | 174.165 |
| c114009_g1_i1 | 297 | 68.877 | 231.909 |
| c114030_g1_i1 | 225 | 24.0655 | 106.958 |
| c114041_g1_i1 | 249 | 111.199 | 446.583 |
| c114047_g1_i1 | 214 | 158.5 | 740.655 |
| c114083_g1_i1 | 287 | 112.859 | 393.236 |
| c114104_g1_i1 | 471 | 92.9425 | 197.33 |
| c114121_g1_i1 | 222 | 47.3011 | 213.068 |
| c114132_g1_i1 | 249 | 29.8744 | 119.977 |
| c114151_g1_i1 | 205 | 470.521 | 2295.23 |
| c114166_g1_i1 | 267 | 43.9817 | 164.726 |
| c114189_g1_i1 | 264 | 32.3639 | 122.591 |
| c114212_g1_i1 | 456 | 78.0053 | 171.064 |
| c114304_g1_i1 | 202 | 49.7906 | 246.488 |
| c114340_g1_i1 | 201 | 64.7278 | 322.029 |
| c114349_g1_i1 | 246 | 53.11 | 215.894 |
| c114384_g1_i1 | 244 | 49.7906 | 204.06 |
| c114412_g1_i1 | 569 | 63.898 | 112.299 |
| c114442_g1_i1 | 211 | 39.0027 | 184.847 |
| c114467_g1_i1 | 238 | 33.1938 | 139.47 |
| c114514_g1_i1 | 287 | 122.817 | 427.933 |
| c114518_g1_i1 | 373 | 41.4922 | 111.239 |
| c114558_g1_i1 | 225 | 193.354 | 859.349 |
| c114659_g1_i1 | 226 | 24.8953 | 110.156 |
| c114745_g1_i1 | 346 | 48.9608 | 141.505 |
| c114786_g1_i1 | 210 | 39.0027 | 185.727 |
| c114807_g1_i1 | 247 | 33.1938 | 134.388 |
| c114850_g1_i1 | 212 | 34.8534 | 164.403 |
| c114944_g1_i1 | 262 | 48.1309 | 183.706 |
| c114980_g1_i1 | 397 | 219.079 | 551.836 |
| c114984_g1_i1 | 290 | 97.9216 | 337.661 |
| c114997_g1_i1 | 236 | 32.3639 | 137.135 |
| c115014_g1_i1 | 259 | 87.9634 | 339.627 |
| c115022_g1_i1 | 443 | 75.5158 | 170.465 |
| c115069_g1_i1 | 205 | 380.068 | 1853.99 |
| c115103_g1_i1 | 475 | 70.5367 | 148.498 |
| c115136_g1_i1 | 238 | 43.9817 | 184.797 |
| c115270_g1_i1 | 251 | 42.322 | 168.614 |
| c115283_g1_i1 | 261 | 43.9817 | 168.512 |
| c115371_g1_i1 | 212 | 21.5759 | 101.773 |
| c115440_g1_i1 | 294 | 289.616 | 985.087 |
| c115448_g1_i1 | 237 | 41.4922 | 175.073 |
| c115552_g1_i1 | 205 | 51.4503 | 250.977 |
| c115554_g1_i1 | 267 | 151.861 | 568.769 |
| c115615_g1_i1 | 274 | 50.6205 | 184.746 |
| c115621_g1_i1 | 331 | 53.9398 | 162.96 |
| c115639_g1_i1 | 207 | 40.6623 | 196.436 |
| c115662_g1_i1 | 286 | 50.6205 | 176.995 |
| c115673_g1_i1 | 272 | 97.9216 | 360.006 |
| c115712_g1_i1 | 236 | 44.8116 | 189.88 |
| c115714_g1_i1 | 244 | 87.1336 | 357.105 |
| c115728_g1_i1 | 311 | 63.898 | 205.46 |
| c115744_g1_i1 | 396 | 53.9398 | 136.212 |
| c115788_g1_i1 | 270 | 34.8534 | 129.087 |
| c115803_g1_i1 | 240 | 38.1728 | 159.053 |
| c115815_g1_i1 | 369 | 68.0472 | 184.41 |
| c115848_g1_i1 | 329 | 72.1964 | 219.442 |
| c115870_g1_i1 | 269 | 48.9608 | 182.01 |
| c115931_g1_i1 | 235 | 55.5995 | 236.594 |
| c115949_g1_i1 | 444 | 170.118 | 383.149 |
| c116010_g1_i1 | 204 | 21.5759 | 105.764 |
| c116012_g1_i1 | 356 | 53.11 | 149.185 |
| c116051_g1_i1 | 250 | 130.285 | 521.142 |
| c116109_g1_i1 | 289 | 143.563 | 496.758 |
| c116141_g1_i1 | 226 | 45.6414 | 201.953 |
| c116189_g1_i1 | 248 | 49.7906 | 200.769 |
| c116203_g1_i1 | 207 | 32.3639 | 156.347 |
| c116262_g1_i1 | 202 | 44.8116 | 221.839 |
| c116318_g1_i1 | 238 | 31.5341 | 132.496 |
| c116353_g1_i1 | 205 | 41.4922 | 202.401 |
| c116426_g1_i1 | 229 | 28.2147 | 123.208 |
| c116459_g1_i1 | 293 | 48.1309 | 164.269 |
| c116499_g1_i1 | 213 | 81.3247 | 381.806 |
| c116514_g1_i1 | 288 | 29.0445 | 100.849 |
| c116527_g1_i1 | 201 | 143.563 | 714.244 |
| c116562_g1_i1 | 322 | 89.6231 | 278.333 |
| c116586_g1_i1 | 209 | 28.2147 | 134.999 |
| c116663_g1_i1 | 286 | 48.9608 | 171.192 |
| c116802_g1_i1 | 209 | 107.88 | 516.171 |
| c116837_g1_i1 | 203 | 22.4058 | 110.373 |
| c116913_g1_i1 | 281 | 37.343 | 132.893 |
| c116950_g1_i1 | 243 | 126.136 | 519.079 |
| c116968_g1_i1 | 218 | 49.7906 | 228.397 |
| c116971_g1_i1 | 201 | 264.72 | 1317.02 |
| c116985_g1_i1 | 220 | 58.0891 | 264.041 |
| c117026_g1_i1 | 255 | 31.5341 | 123.663 |
| c117213_g1_i1 | 246 | 50.6205 | 205.774 |
| c117276_g1_i1 | 393 | 166.799 | 424.424 |
| c117284_g1_i1 | 344 | 37.343 | 108.555 |
| c117301_g1_i1 | 211 | 26.555 | 125.853 |
| c117304_g1_i1 | 673 | 332.767 | 494.454 |
| c117335_g1_i1 | 562 | 102.071 | 181.621 |
| c117374_g1_i1 | 348 | 213.27 | 612.844 |
| c117425_g1_i1 | 282 | 171.778 | 609.141 |
| c117445_g1_i1 | 261 | 26.555 | 101.743 |
| c117471_g1_i1 | 205 | 26.555 | 129.537 |
| c117525_g1_i1 | 365 | 42.322 | 115.951 |
| c117662_g1_i1 | 315 | 141.073 | 447.852 |
| c117672_g1_i1 | 298 | 37.343 | 125.312 |
| c117675_g1_i1 | 208 | 48.1309 | 231.399 |
| c117703_g1_i1 | 290 | 43.9817 | 151.661 |
| c117746_g1_i1 | 215 | 103.73 | 482.467 |
| c117753_g1_i1 | 308 | 63.0681 | 204.767 |
| c117820_g1_i1 | 424 | 43.1519 | 101.773 |
| c117833_g1_i1 | 231 | 24.8953 | 107.772 |
| c117886_g1_i1 | 330 | 138.584 | 419.951 |
| c117912_g1_i1 | 218 | 140.244 | 643.319 |
| c117949_g1_i1 | 252 | 48.9608 | 194.289 |
| c117975_g1_i1 | 204 | 34.0236 | 166.782 |
| c118035_g1_i1 | 218 | 54.7697 | 251.237 |
| c118061_g1_i1 | 239 | 43.9817 | 184.024 |
| c118062_g1_i1 | 203 | 20.7461 | 102.198 |
| c118078_g1_i1 | 241 | 34.8534 | 144.62 |
| c118088_g1_i1 | 310 | 85.4739 | 275.722 |
| c118089_g1_i1 | 248 | 77.1755 | 311.191 |
| c118111_g1_i1 | 392 | 124.477 | 317.542 |
| c118150_g1_i1 | 206 | 27.3848 | 132.936 |
| c118152_g1_i1 | 325 | 47.3011 | 145.542 |
| c118157_g1_i1 | 298 | 40.6623 | 136.451 |
| c118208_g1_i1 | 713 | 165.969 | 232.775 |
| c118235_g1_i1 | 581 | 287.126 | 494.193 |
| c118250_g1_i1 | 328 | 44.8116 | 136.621 |
| c118266_g1_i1 | 214 | 133.605 | 624.322 |
| c118374_g1_i1 | 223 | 50.6205 | 226.998 |
| c118375_g1_i1 | 441 | 236.505 | 536.294 |
| c118444_g1_i1 | 346 | 146.053 | 422.117 |
| c118454_g1_i1 | 209 | 59.7488 | 285.879 |
| c118464_g1_i1 | 256 | 28.2147 | 110.214 |
| c118568_g1_i1 | 224 | 129.456 | 577.927 |
| c118579_g1_i1 | 202 | 43.9817 | 217.731 |
| c118627_g1_i1 | 202 | 154.351 | 764.114 |
| c118632_g1_i1 | 237 | 71.3666 | 301.125 |
| c118669_g1_i1 | 253 | 117.838 | 465.762 |
| c118706_g1_i1 | 298 | 61.4084 | 206.069 |
| c118759_g1_i1 | 267 | 60.5786 | 226.886 |
| c118853_g1_i1 | 377 | 473.841 | 1256.87 |
| c118869_g1_i1 | 243 | 61.4084 | 252.71 |
| c118870_g1_i1 | 276 | 126.136 | 457.015 |
| c118887_g1_i1 | 431 | 87.1336 | 202.166 |
| c118934_g1_i1 | 206 | 48.1309 | 233.645 |
| c118944_g1_i1 | 372 | 59.7488 | 160.615 |
| c118971_g1_i1 | 242 | 37.343 | 154.31 |
| c118976_g1_i1 | 276 | 62.2383 | 225.501 |
| c118985_g1_i1 | 217 | 63.0681 | 290.637 |
| c118993_g1_i1 | 333 | 58.0891 | 174.442 |
| c119030_g1_i1 | 235 | 25.7252 | 109.469 |
| c119040_g1_i1 | 373 | 38.1728 | 102.34 |
| c119056_g1_i1 | 460 | 195.013 | 423.942 |
| c119129_g1_i1 | 352 | 86.3038 | 245.181 |
| c119212_g1_i1 | 309 | 77.1755 | 249.759 |
| c119265_g1_i1 | 202 | 66.3875 | 328.651 |
| c119285_g1_i1 | 202 | 136.094 | 673.735 |
| c119324_g1_i1 | 397 | 93.7724 | 236.202 |
| c119328_g1_i1 | 292 | 29.8744 | 102.31 |
| c119379_g1_i1 | 333 | 151.861 | 456.04 |
| c119415_g1_i1 | 336 | 37.343 | 111.14 |
| c119471_g1_i1 | 578 | 107.88 | 186.643 |
| c119476_g1_i1 | 249 | 45.6414 | 183.299 |
| c119478_g1_i1 | 241 | 180.906 | 750.647 |
| c119531_g1_i1 | 327 | 50.6205 | 154.803 |
| c119547_g1_i1 | 273 | 61.4084 | 224.939 |
| c119570_g1_i1 | 284 | 147.712 | 520.113 |
| c119572_g1_i1 | 293 | 34.8534 | 118.954 |
| c119613_g1_i1 | 283 | 39.0027 | 137.819 |
| c119637_g1_i1 | 243 | 74.6859 | 307.35 |
| c119685_g1_i1 | 251 | 32.3639 | 128.94 |
| c119702_g1_i1 | 248 | 37.343 | 150.577 |
| c119707_g1_i1 | 332 | 173.437 | 522.402 |
| c119725_g1_i1 | 207 | 23.2356 | 112.249 |
| c119739_g1_i1 | 427 | 156.011 | 365.365 |
| c119750_g1_i1 | 310 | 76.3456 | 246.276 |
| c119790_g1_i1 | 290 | 46.4713 | 160.246 |
| c119843_g1_i1 | 215 | 24.8953 | 115.792 |
| c119869_g1_i1 | 347 | 44.8116 | 129.14 |
| c119881_g1_i1 | 248 | 63.0681 | 254.307 |
| c119928_g1_i1 | 298 | 190.864 | 640.484 |
| c119974_g1_i1 | 208 | 43.1519 | 207.461 |
| c120054_g1_i1 | 236 | 83.8142 | 355.145 |
| c120057_g1_i1 | 217 | 29.8744 | 137.67 |
| c120058_g1_i1 | 227 | 29.0445 | 127.949 |
| c120069_g1_i1 | 242 | 48.9608 | 202.317 |
| c120076_g1_i1 | 343 | 36.5131 | 106.452 |
| c120123_g1_i1 | 296 | 41.4922 | 140.176 |
| c120128_g1_i1 | 330 | 37.343 | 113.161 |
| c120158_g1_i1 | 247 | 44.8116 | 181.423 |
| c120222_g1_i1 | 306 | 58.9189 | 192.545 |
| c120238_g1_i1 | 232 | 43.9817 | 189.576 |
| c120312_g1_i1 | 259 | 122.817 | 474.196 |
| c120383_g1_i1 | 228 | 30.7042 | 134.668 |
| c120413_g1_i1 | 296 | 36.5131 | 123.355 |
| c120472_g1_i1 | 266 | 27.3848 | 102.951 |
| c120541_g1_i1 | 265 | 52.2802 | 197.284 |
| c120578_g1_i1 | 221 | 26.555 | 120.158 |
| c120588_g1_i1 | 324 | 44.8116 | 138.307 |
| c120602_g1_i1 | 231 | 187.545 | 811.882 |
| c120686_g1_i1 | 223 | 34.8534 | 156.293 |
| c120702_g1_i1 | 265 | 59.7488 | 225.467 |
| c120729_g1_i1 | 292 | 63.0681 | 215.987 |
| c120753_g1_i1 | 218 | 23.2356 | 106.585 |
| c120756_g1_i1 | 250 | 29.0445 | 116.178 |
| c120775_g1_i1 | 332 | 50.6205 | 152.471 |
| c120789_g1_i1 | 402 | 66.3875 | 165.143 |
| c120810_g1_i1 | 214 | 31.5341 | 147.355 |
| c120831_g1_i1 | 234 | 99.5813 | 425.561 |
| c120967_g1_i1 | 235 | 78.8352 | 335.469 |
| c120991_g1_i1 | 256 | 46.4713 | 181.528 |
| c121002_g1_i1 | 248 | 121.987 | 491.883 |
| c121018_g1_i1 | 226 | 48.1309 | 212.969 |
| c121086_g1_i1 | 205 | 42.322 | 206.449 |
| c121110_g1_i1 | 212 | 62.2383 | 293.577 |
| c121227_g1_i1 | 202 | 111.199 | 550.49 |
| c121405_g1_i1 | 267 | 132.775 | 497.285 |
| c121406_g1_i1 | 212 | 293.765 | 1385.68 |
| c121466_g1_i1 | 203 | 102.901 | 506.9 |
| c121484_g1_i1 | 391 | 49.7906 | 127.342 |
| c121528_g1_i1 | 222 | 82.9844 | 373.804 |
| c121545_g1_i1 | 230 | 43.9817 | 191.225 |
| c121569_g1_i1 | 315 | 330.278 | 1048.5 |
| c121576_g1_i1 | 251 | 47.3011 | 188.451 |
| c121620_g1_i1 | 439 | 102.901 | 234.398 |
| c121621_g1_i1 | 303 | 31.5341 | 104.073 |
| c121628_g1_i1 | 324 | 128.626 | 396.993 |
| c121655_g1_i1 | 245 | 31.5341 | 128.71 |
| c121724_g1_i1 | 241 | 30.7042 | 127.403 |
| c121826_g1_i1 | 232 | 193.354 | 833.421 |
| c121835_g1_i1 | 414 | 85.4739 | 206.459 |
| c121903_g1_i1 | 288 | 37.343 | 129.663 |
| c121904_g1_i1 | 311 | 41.4922 | 133.415 |
| c121924_g1_i1 | 601 | 69.7069 | 115.985 |
| c121965_g1_i1 | 262 | 341.066 | 1301.78 |
| c121966_g1_i1 | 259 | 44.8116 | 173.018 |
| c121983_g1_i1 | 249 | 32.3639 | 129.976 |
| c121986_g1_i1 | 259 | 48.9608 | 189.038 |
| c122032_g1_i1 | 291 | 45.6414 | 156.843 |
| c122065_g1_i1 | 221 | 163.479 | 739.725 |
| c122089_g1_i1 | 261 | 31.5341 | 120.82 |
| c122146_g1_i1 | 224 | 36.5131 | 163.005 |
| c122169_g1_i1 | 296 | 63.0681 | 213.068 |
| c122230_g1_i1 | 202 | 32.3639 | 160.217 |
| c122257_g1_i1 | 265 | 32.3639 | 122.128 |
| c122261_g1_i1 | 226 | 93.7724 | 414.922 |
| c122291_g1_i1 | 258 | 32.3639 | 125.442 |
| c122346_g1_i1 | 327 | 33.1938 | 101.51 |
| c122396_g1_i1 | 232 | 41.4922 | 178.846 |
| c122412_g1_i1 | 290 | 302.893 | 1044.46 |
| c122415_g1_i1 | 215 | 23.2356 | 108.073 |
| c122447_g1_i1 | 204 | 53.11 | 260.343 |
| c122458_g1_i1 | 263 | 60.5786 | 230.337 |
| c122518_g1_i1 | 224 | 34.8534 | 155.596 |
| c122538_g1_i1 | 238 | 107.88 | 453.276 |
| c122552_g1_i1 | 242 | 30.7042 | 126.877 |
| c122637_g1_i1 | 251 | 342.726 | 1365.44 |
| c122640_g1_i1 | 202 | 39.0027 | 193.082 |
| c122652_g1_i1 | 336 | 175.097 | 521.122 |
| c122679_g1_i1 | 215 | 43.9817 | 204.566 |
| c122702_g1_i1 | 217 | 56.4294 | 260.043 |
| c122727_g1_i1 | 245 | 25.7252 | 105.001 |
| c122801_g1_i1 | 373 | 63.898 | 171.308 |
| c122821_g1_i1 | 212 | 23.2356 | 109.602 |
| c122861_g1_i1 | 303 | 33.1938 | 109.55 |
| c122875_g1_i1 | 203 | 59.7488 | 294.329 |
| c122894_g1_i1 | 204 | 23.2356 | 113.9 |
| c122994_g1_i1 | 417 | 60.5786 | 145.272 |
| c123063_g1_i1 | 228 | 26.555 | 116.469 |
| c123090_g1_i1 | 507 | 117.838 | 232.422 |
| c123101_g1_i1 | 213 | 63.0681 | 296.095 |
| c123133_g1_i1 | 205 | 29.8744 | 145.729 |
| c123212_g1_i1 | 218 | 168.458 | 772.744 |
| c123252_g1_i1 | 236 | 34.0236 | 144.168 |
| c123277_g1_i1 | 381 | 42.322 | 111.081 |
| c123284_g1_i1 | 285 | 60.5786 | 212.556 |
| c123285_g1_i1 | 510 | 117.838 | 231.055 |
| c123292_g1_i1 | 213 | 23.2356 | 109.087 |
| c123322_g1_i1 | 225 | 53.9398 | 239.733 |
| c123355_g1_i1 | 302 | 49.7906 | 164.87 |
| c123430_g1_i1 | 242 | 69.7069 | 288.045 |
| c123444_g1_i1 | 227 | 29.0445 | 127.949 |
| c123449_g1_i1 | 217 | 22.4058 | 103.252 |
| c123460_g1_i1 | 207 | 31.5341 | 152.338 |
| c123468_g1_i1 | 225 | 76.3456 | 339.314 |
| c123471_g1_i1 | 461 | 92.1127 | 199.811 |
| c123478_g1_i1 | 227 | 106.22 | 467.93 |
| c123480_g1_i1 | 236 | 32.3639 | 137.135 |
| c123515_g1_i1 | 280 | 214.93 | 767.606 |
| c123559_g1_i1 | 209 | 74.6859 | 357.349 |
| c123605_g1_i1 | 281 | 29.8744 | 106.315 |
| c123670_g1_i1 | 203 | 34.0236 | 167.604 |
| c123680_g1_i1 | 208 | 40.6623 | 195.492 |
| c123693_g1_i1 | 440 | 45.6414 | 103.73 |
| c123708_g1_i1 | 236 | 41.4922 | 175.814 |
| c123717_g1_i1 | 240 | 183.395 | 764.148 |
| c123721_g1_i1 | 204 | 40.6623 | 199.325 |
| c123741_g1_i1 | 269 | 39.8325 | 148.076 |
| c123742_g1_i1 | 241 | 30.7042 | 127.403 |
| c123762_g1_i1 | 365 | 39.0027 | 106.857 |
| c123798_g1_i1 | 239 | 30.7042 | 128.47 |
| c123838_g1_i1 | 202 | 40.6623 | 201.299 |
| c123862_g1_i1 | 206 | 30.7042 | 149.05 |
| c123868_g1_i1 | 273 | 28.2147 | 103.351 |
| c123935_g1_i1 | 450 | 55.5995 | 123.555 |
| c123951_g1_i1 | 272 | 40.6623 | 149.494 |
| c123967_g1_i1 | 206 | 39.0027 | 189.333 |
| c123981_g1_i1 | 217 | 23.2356 | 107.077 |
| c124017_g1_i1 | 243 | 26.555 | 109.28 |
| c124029_g1_i1 | 466 | 124.477 | 267.117 |
| c124033_g1_i1 | 216 | 38.1728 | 176.726 |
| c124087_g1_i1 | 218 | 33.1938 | 152.265 |
| c124104_g1_i1 | 247 | 52.2802 | 211.661 |
| c124129_g1_i1 | 266 | 52.2802 | 196.542 |
| c124375_g1_i1 | 215 | 34.0236 | 158.249 |
| c124379_g1_i1 | 205 | 131.945 | 643.635 |
| c124430_g1_i1 | 273 | 32.3639 | 118.549 |
| c124459_g1_i1 | 305 | 125.306 | 410.841 |
| c124471_g1_i1 | 261 | 86.3038 | 330.666 |
| c124528_g1_i1 | 302 | 39.0027 | 129.148 |
| c124530_g1_i1 | 223 | 48.9608 | 219.555 |
| c124539_g1_i1 | 218 | 69.7069 | 319.756 |
| c124675_g1_i1 | 309 | 237.335 | 768.076 |
| c124777_g1_i1 | 226 | 53.11 | 235 |
| c124790_g1_i1 | 236 | 34.8534 | 147.684 |
| c124822_g1_i1 | 248 | 70.5367 | 284.422 |
| c124838_g1_i1 | 284 | 84.6441 | 298.043 |
| c124854_g1_i1 | 222 | 23.2356 | 104.665 |
| c124868_g1_i1 | 239 | 24.0655 | 100.692 |
| c124904_g1_i1 | 211 | 148.542 | 703.991 |
| c124913_g1_i1 | 279 | 36.5131 | 130.871 |
| c124918_g1_i1 | 227 | 32.3639 | 142.572 |
| c124942_g1_i1 | 261 | 51.4503 | 197.128 |
| c124947_g1_i1 | 229 | 381.728 | 1666.94 |
| c124950_g1_i1 | 246 | 124.477 | 506.002 |
| c124969_g1_i1 | 225 | 29.0445 | 129.087 |
| c125029_g1_i1 | 249 | 27.3848 | 109.979 |
| c125097_g1_i1 | 207 | 49.7906 | 240.534 |
| c125101_g1_i1 | 265 | 79.665 | 300.623 |
| c125116_g1_i1 | 405 | 40.6623 | 100.401 |
| c125121_g1_i1 | 258 | 47.3011 | 183.338 |
| c125143_g1_i1 | 227 | 30.7042 | 135.261 |
| c125153_g1_i1 | 242 | 89.6231 | 370.344 |
| c125170_g1_i1 | 210 | 21.5759 | 102.743 |
| c125275_g1_i1 | 220 | 26.555 | 120.705 |
| c125277_g1_i1 | 268 | 101.241 | 377.765 |
| c125360_g1_i1 | 206 | 101.241 | 491.461 |
| c125439_g1_i1 | 207 | 69.7069 | 336.748 |
| c125509_g1_i1 | 240 | 42.322 | 176.342 |
| c125530_g1_i1 | 216 | 49.7906 | 230.512 |

**Supplementary table 6. Mapping statistics of small RNAs from whole worm and Macpiwi1 IP**

**Total small RNAs**

|  | Whole worm | | | | Macpiwi1 IP | | | |
| --- | --- | --- | --- | --- | --- | --- | --- | --- |
|  | Replicate 1 | | Replicate 2 | | Replicate 1 | | Replicate 2 | |
|  | Abundance | Complexity | Abundance | Complexity | Abundance | Complexity | Abundance | Complexity |
| Total reads | 14193547 | 1580600 | 15092856 | 1089039 | 8411889 | 673929 | 13727400 | 509896 |
| All mappers | 10207564 | 1008705 | 10986209 | 678302 | 4814242 | 350851 | 7042584 | 239011 |
| Unique mappers | 2939838 | 335427 | 3016291 | 214978 | 1358671 | 142882 | 2056067 | 102536 |

**piRNAs (28-32nt)**

|  | Whole worm | | | | Macpiwi1 IP | | | |
| --- | --- | --- | --- | --- | --- | --- | --- | --- |
|  | Replicate 1 | | Replicate 2 | | Replicate 1 | | Replicate 2 | |
|  | Abundance | Complexity | Abundance | Complexity | Abundance | Complexity | Abundance | Complexity |
| All mappers | 3510975 | 670484 | 3198136 | 414195 | 3180859 | 266321 | 4912910 | 190265 |
| Unique mappers | 902418 | 253361 | 764398 | 154113 | 1023860 | 119031 | 1558519 | 85384 |
| Unique mappers % | 25.70 | 37.79 | 23.90 | 37.21 | 32.19 | 44.69 | 31.72 | 44.88 |
